# Asymmetric Full Saturation of Vinylarenes with Cooperative Homogeneous and Heterogeneous Rhodium Catalysis

**DOI:** 10.1021/jacs.1c09975

**Published:** 2021-11-22

**Authors:** Haibo Wu, Jianping Yang, Bram B. C. Peters, Luca Massaro, Jia Zheng, Pher G. Andersson

**Affiliations:** † Department of Organic Chemistry, Stockholm University, Svante Arrhenius väg 16C, SE-10691 Stockholm, Sweden; ‡ School of Chemistry and Physics, University of Kwazulu-Natal, Private Bag X54001, Durban 4000, South Africa

## Abstract

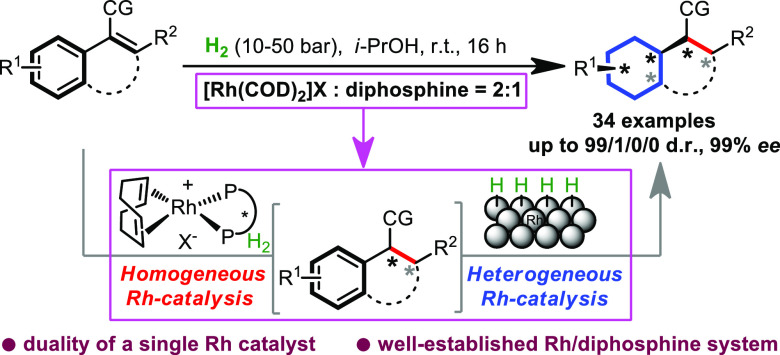

Homogeneous and heterogeneous catalyzed reactions can seldom operate synergistically under the same conditions. Here we communicate the use of a single rhodium precursor that acts in both the homogeneous and heterogeneous phases for the asymmetric full saturation of vinylarenes that, to date, constitute an unmet bottleneck in the field. A simple asymmetric hydrogenation of a styrenic olefin, enabled by a ligand accelerated effect, accounted for the facial selectivity in the consecutive arene hydrogenation. Tuning the ratio between the phosphine ligand and the rhodium precursor controlled the formation of homogeneous and heterogeneous catalytic species that operate without interference from each other. The system is flexible in terms of both the chiral ligand and the nature of the external olefin. We anticipate that our findings will promote the development of asymmetric arene hydrogenations.

## Introduction

**Figure 1:**
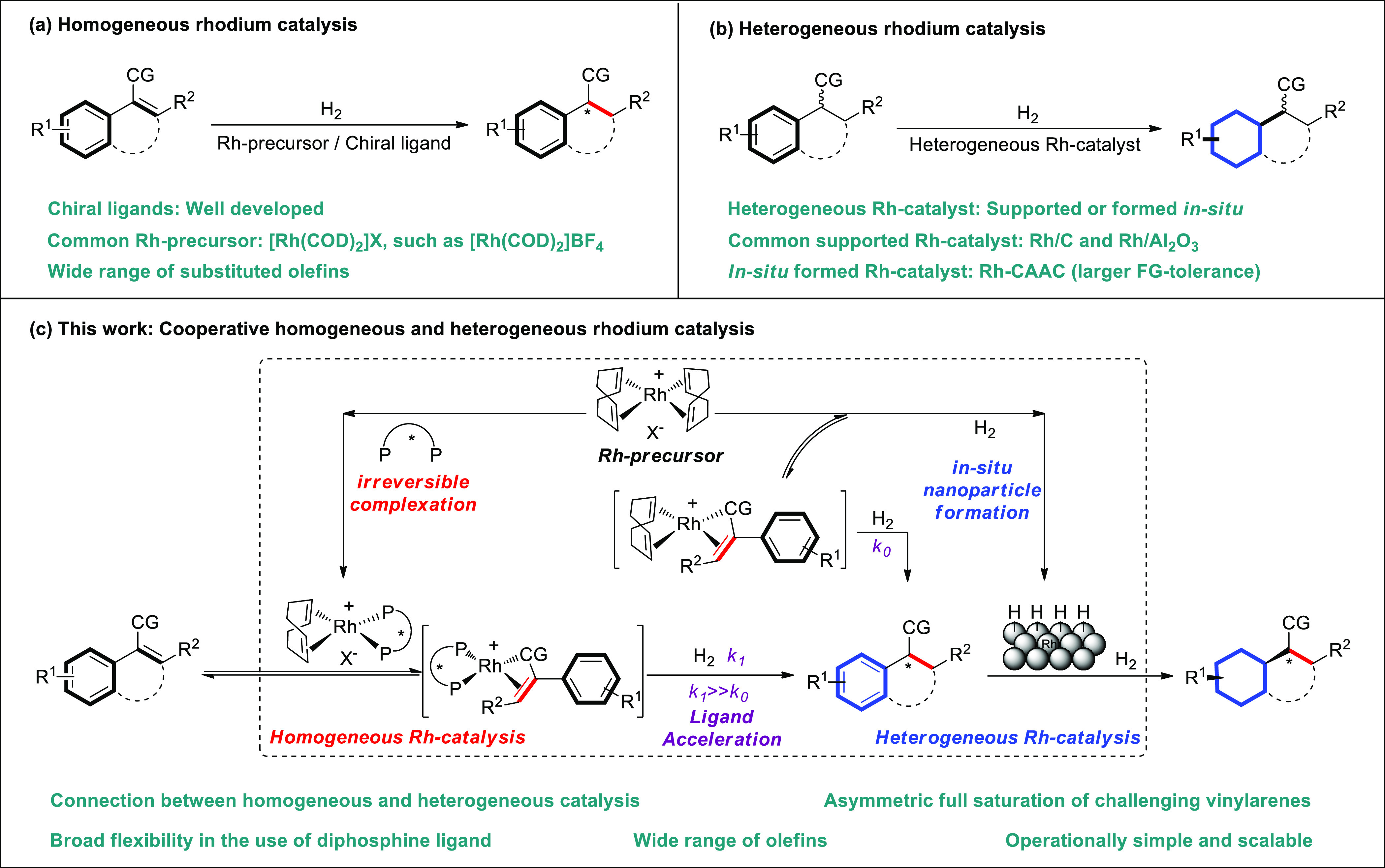
Rhodium-catalyzed hydrogenation of olefins and aromatic rings. (a) Homogeneous rhodium-catalyzed asymmetric hydrogenation of olefins. (b) Heterogeneous rhodium-catalyzed hydrogenation of aromatic rings. (c) This work: Asymmetric dearomative hydrogenation with cooperative homogeneous and heterogeneous rhodium catalysis.

Catalysis plays a fundamental and key role in organic synthesis and is used as a tool for the production of numerous pharmaceuticals, natural products, agrochemicals, and fine chemicals.^1,2^ Homogeneous and heterogeneous catalyzed hydrogenation constitute two well-developed areas in both industry and academia and have independently been awarded shares of Nobel prizes (1912, Sabatier; 2001, Noyori and Knowles).^3−5^ Over the past decades, rhodium has emerged as a robust metal for homogeneous as well as heterogeneous catalyzed hydrogenation. The development of chiral ligands (mainly diphosphines) that in many cases are commercially available presently has driven and expanded homogeneous rhodium-catalyzed hydrogenation of diversely substituted olefins largely to become a powerful strategy for the production of optically active compounds (Figure 1a).^6,7^ On the other hand, heterogeneous rhodium catalysts have been found to be reactive toward the hydrogenation of aromatic unsaturated bonds and applied therein (Figure. 1b).^8^ However, asymmetric hydrogenation of aromatic compounds is restricted to heteroaromatic rings and fused arenes, whereas the asymmetric hydrogenation of simple substituted benzenes remains a formidable goal.^9−12^ This is mainly attributed to the relatively high resonance stability of all-carbon aromatic rings compared to the hetero- or fused- aromatic ring systems. Despite the reduction of arenes having been known for many decades, no chiral catalyst is known for the asymmetric hydrogenation of simple substituted arenes; yet, it is highly sought after. Traditionally, the hydrogenation of arenes is achieved by using solid supported heterogeneous rhodium catalysts.^13^ In recent years, homogeneous precursors are more frequently used as an alternative that can form nanoparticles under the reaction conditions.^14−21^ These nanoparticles showed higher reactivity and a larger functional group tolerance compared to solid supported metal catalysts. To date, a clear separation between homogeneous and heterogeneous rhodium-catalyzed hydrogenation exists. Ideally, the advances in both fields of catalysis combined could tackle the asymmetric hydrogenation of arenes that remains a largely unsolved bottleneck in state-of-the-art hydrogenation.

Dearomative transformations, reactions able to break and functionalize the π-system in aromatic compounds, are of high interest.^22,23^ In particular the pharmaceutical industry stands to benefit since it allows late-stage introduction of complex structural diversity into lead compounds.^24^ Most importantly, aromatic precursors are readily accessible, and various methodologies targeting aromatic sp^2^-hybridized carbons have been developed.^25^ Therefore, dearomatization offers a direct opportunity to escape flat land out of relatively simple and abundant starting materials. A convenient and atom efficient strategy would be to use molecular hydrogen for this purpose. This hydrogenative strategy would largely increase in value if used in an asymmetric fashion since it is known that stereoisomers often exhibit different pharmacological properties.^26^ Here we communicate the use of diphosphine ligands in combination with common rhodium precursors for the asymmetric full saturation of vinylarenes that operates via cooperative homogeneous and heterogeneous catalysis (Figure 1c). This protocol offers a very flexible and conceptionally novel approach that relies on a dual system: an irreversible binding between diphosphine and rhodium, a very efficient ligand accelerated asymmetric hydrogenation of an olefin, and the *in situ* aggregation of nonligated rhodium into a very active nanoparticle for the hydrogenation of the aromatic ring that results in the facile construction of chiral cyclohexane motifs.

## Results and Discussion

### Discovery of the Duality of [Rh(COD)_2_]BF_4_

**Figure 2:**
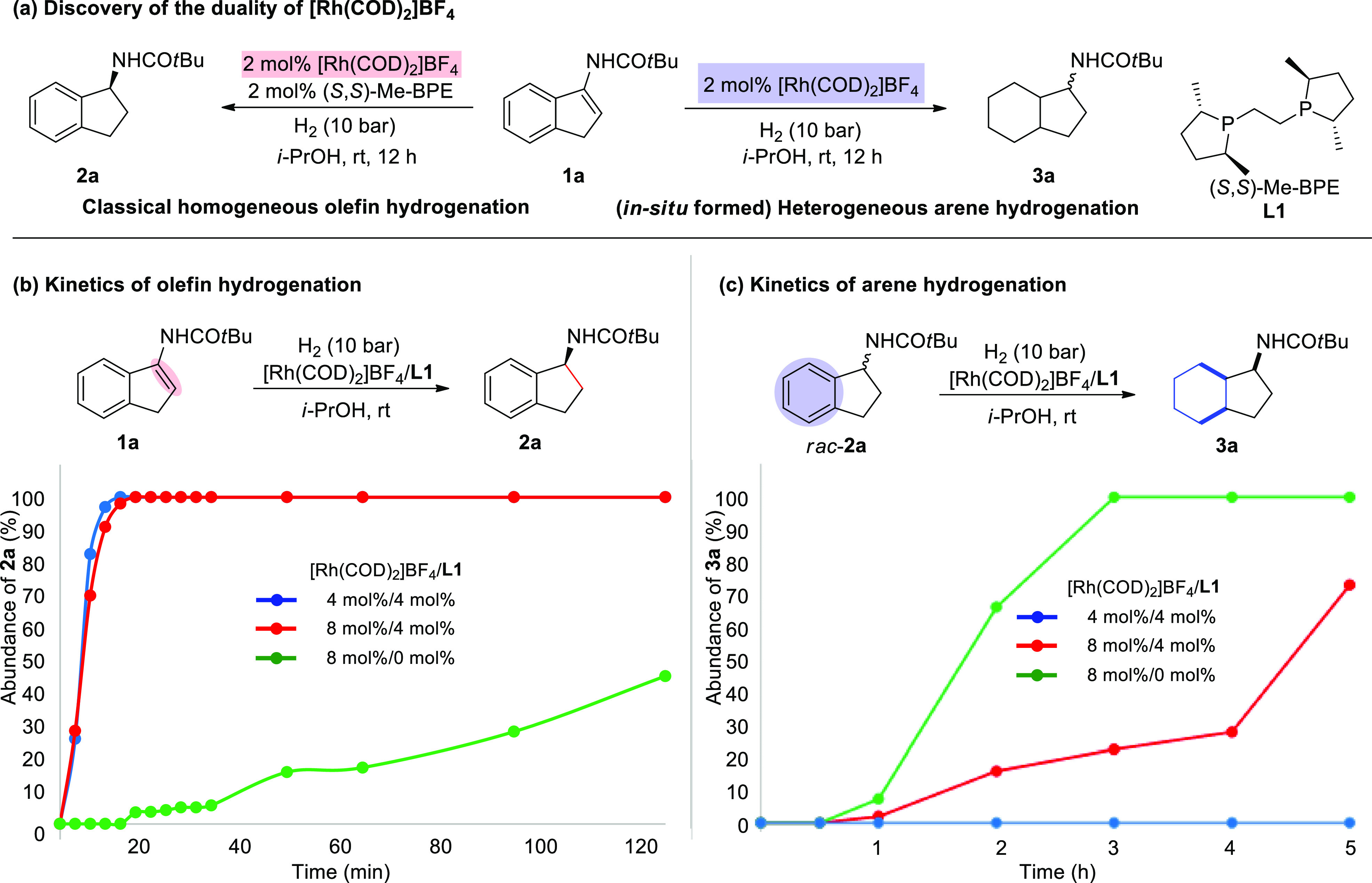
Development of arene hydrogenation based on classical Rh/diphosphine system. (a) Discovery of the duality of [Rh(COD)_2_]BF_4_ as the active catalyst for olefin/arene hydrogenation. (b) Kinetics of olefin hydrogenation. The green line refers to the total amount of **2a** and **3a**. (c) Kinetics of arene hydrogenation.

Seeking the development of an asymmetric hydrogenation of arenes, initial studies on the reactivity of rhodium catalysts demonstrated that common rhodium precursors in the form of [Rh(COD)_2_]X (X = anion) aggregate into very reactive nanoparticles for the hydrogenation of substituted benzenes (Figure 2a). We realized that the exact same precursors are frequently used for the homogeneous rhodium-diphosphine-catalyzed asymmetric hydrogenation of olefins, which is one of the most studied types of asymmetric hydrogenation.^6,27^ Notably, although some neutral rhodium complexes (such as [Rh(COD)Cl]_2_^28^ and [Rh(η^5^-C_5_Me_5_)Cl_2_]_2_^29,30^) were found as effective precatalysts for dearomative (transfer) hydrogenations, the duality of commonly used cationic rhodium precursors ([Rh(COD)_2_]X) has not been reported so far. The active catalyst in these homogeneous asymmetric hydrogenations is normally formed *in situ* by mixing a rhodium-precursor and diphosphine ligand. Usually, a slight excess of ligand is used to ensure full complexation of all monomeric rhodium and thus prevent a racemic background reaction by nonligated catalyst. The very strong binding affinity between rhodium and diphosphine ligands makes ligation practically irreversible and prevents the persistence of nonligated rhodium monomers.^31^ We anticipated that a single rhodium precursor can simultaneously form both chiral homogeneous complexes and the heterogeneous nanoparticles.^32^ Our envisioned strategy would use an excess of rhodium precursors to direct the hydrogenation of arenes by first forming a stereogenic center by an enantioselective hydrogenation of styrenic olefins.

### Kinetic Studies

First, kinetic experiments were performed to study the relative reactivity of the catalytic homogeneous and heterogeneous species. The hydrogenation of the olefin was monitored first (Figure 2b). Using [Rh(COD)_2_]BF_4_ as the catalyst, an induction period was observed and the hydrogenation started after a period of 12–15 min (green line). From this point hydrogenation proceeded gradually to reach 45% of **2a** in 2 h. A large rate acceleration of the hydrogenation was observed when [Rh(COD)_2_]BF_4_ and the diphosphine **L1** were used in an equimolar ratio and under these conditions hydrogenation was complete in 15 min and gave rise to the product in high stereoselectivity (blue line, 99% *ee*). Intriguingly, using [Rh(COD)_2_]BF_4_ and **L1** in a 2:1 ratio, full conversion and 99% *ee* was again reached within 15 min (red line). These observations demonstrate that rhodium catalyzed hydrogenation of olefins is a very efficient form of ligand accelerated catalysis^33^ in which the addition of a diphosphine ligand leads to an enormous rate acceleration (*k*_1_ ≫ *k*_0_, Figure 1c). Since no loss in enantioselectivity was observed when using a sub stoichiometric amount of ligand, it was demonstrated that the ligand accelerating effect using **L1** is of a sufficient magnitude to completely outperform the hydrogenation by nonligated rhodium particles that will not interfere with asymmetric olefin hydrogenation. The relatively low reactivity of [Rh(COD)_2_]BF_4_ compared to that of [Rh(COD)_2_]BF_4_ and **L1** (2:1) indicates that the [Rh(COD)(solvent)_2_]BF_4_ complex is stabilized by styrenes and forms nanoparticles more easily once the styrenic olefin has been reduced.

Next, the hydrogenation of the aromatic ring was monitored using *rac*-**2a** as the substrate (Figure 2c). The Rh-**L1** complex did not catalyze the hydrogenation of the aromatic ring (blue line) whereas 4 mol % of [Rh(COD)_2_]BF_4_ alone gave rise to a fast reaction and full conversion in 3 h (green line). A control experiment was carried out doping the reaction with benzothiophene (see the SI) that inhibit the reaction and demonstrated that the arene hydrogenation involves heterogeneous catalysis.^34^ The *in situ* formation of heterogeneous Rh-nanoparticles was further confirmed by HR-TEM images (Figure S3). Interestingly, 8 mol % of [Rh(COD)_2_]BF_4_ together with 4 mol % **L1** hydrogenates arene significantly slower (red line, 73% conversion in 5 h) as compared to 4 mol % of [Rh(COD)_2_]BF_4_ alone (full conversion in 3 h). Many studies demonstrated that the presence of phosphine ligands in solution affects the nanoparticle properties and thus can alter the reactivity toward arene hydrogenation.^35−38^ The kinetic data suggests that both the olefin and the arene hydrogenation are two separate processes that can cooperate as a result of ligand binding and ligand accelerated catalysis.

### Evaluation of Rh-Precursors and the Generality of Diphosphine Ligands

Based on the understanding of the kinetics of both olefin and arene hydrogenation processes, we then proceeded to explore the potential of the well-established Rh/diphosphine system for the asymmetric dearomative hydrogenation. First, a series of commonly used rhodium precursors were tested for the hydrogenation of compound **4a** that is normally used as a benchmark substrate for the rhodium-catalyzed asymmetric hydrogenation of olefins (Table 1). This olefin was completely hydrogenated in all cases and in addition, the aromatic ring was reduced to a varying extent (entries 1–6, 23–86% of **6a**). [Rh(COD)_2_]BF_4_ and [Rh(COD)_2_]SbF_6_ were found to be most reactive catalysts and formed **6a** in 85% and 86% yield respectively (entries 2 and 4). Addition of a slight excess of **L2** (1.1 equiv) to the hydrogenation produced exclusively **5a** in 99% *ee*, as anticipated (entry 7). Intriguingly, we found that **6a** could be formed in the exact same enantioselectivity as **5a** is formed by using a well-chosen 2:1 ratio between the rhodium precursor and **L2** (entries 8–13). To our delight, full conversion toward **6a** in 99% *ee* was obtained when [Rh(COD)_2_]BF_4_ and [Rh(COD)_2_]SbF_6_ were used as rhodium precursors (entries 9 and 11). Thus, nonligated rhodium can be present in solution without interference in the olefin hydrogenation. Most important, the same precursor also forms a reactive catalyst for the hydrogenation of arenes that allows the formation of optically pure saturated cyclohexanes. In a control experiment (entry 14), Rh on carbon was used instead of the *in situ* generated Rh-nanoparticles, a significant decrease of enantioselectvity (79% *ee*) was observed.

**Table 1:**
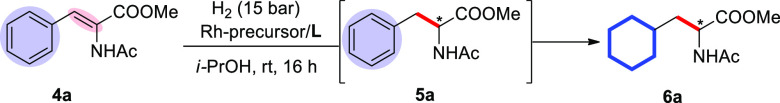

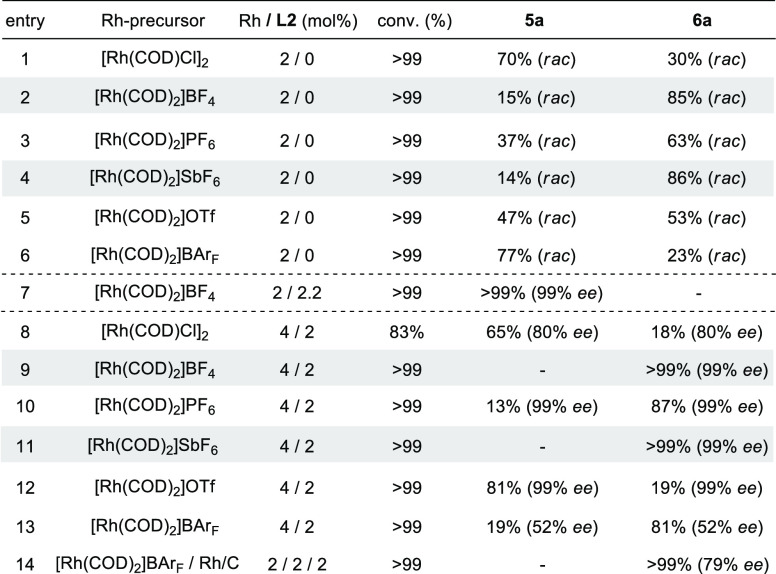
Evaluation of Rh Precursors^a^

a The reactions were carried out with **4a** (0.05 mmol) in 1.0 mL of *i*-PrOH at room temperature. Conversions (conv.) were determined by ^1^H NMR spectroscopy. Enantiomeric excesses (*ee*) were determined by GC analysis using chiral stationary phase.

In principle, any Rh/diphosphine catalyzed hydrogenation of olefins can be followed up with the consecutive hydrogenation of the arene using this concept. The generality in terms of chiral ligand was demonstrated using structurally diverse diphosphine ligands^39^ (Table 2). When **L2** was used in a 1:1 ratio with [Rh(COD)_2_]BF_4_, **4a** was fully converted to **5a** in 99% *ee* (entry 1). The arene was also hydrogenated when the Rh:**L2** ratio was increased to 2:1 and product **6a** was obtained in a clean manner (entry 2, 99% *ee*). The hydrogenation of **4a** to either of the products proceeded also in 99% *ee* when **L3** and **L4** were used (entries 3–6). Even though **L5** and **L6** produced **6a** in a lower enantiomeric excess compared to **L2**-**4** (entries 7–10), using the conditions developed herein, still produced **6a** in the same range of *ee* as **5a**.

**Table 2:**
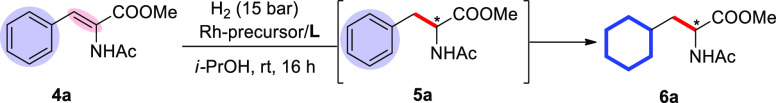

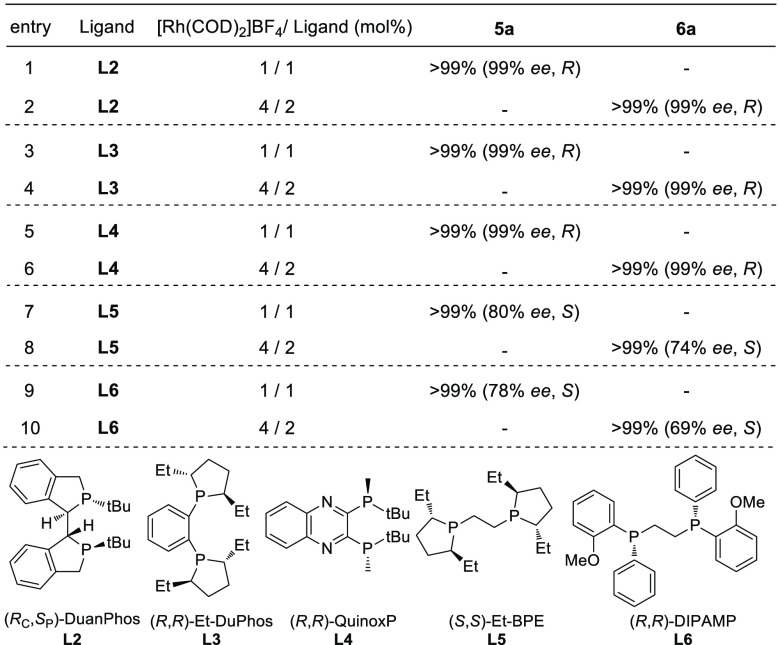
Generality of Diphosphine Ligands^a^

a The reactions were carried out with **4a** (0.05 mmol) in 1.0 mL of *i*-PrOH at room temperature. Conversions were determined by ^1^H NMR spectroscopy. Enantiomeric excesses (*ee*) were determined by GC analysis using chiral stationary phase.

### Evaluation of Substrate Classes

**Scheme 1:**
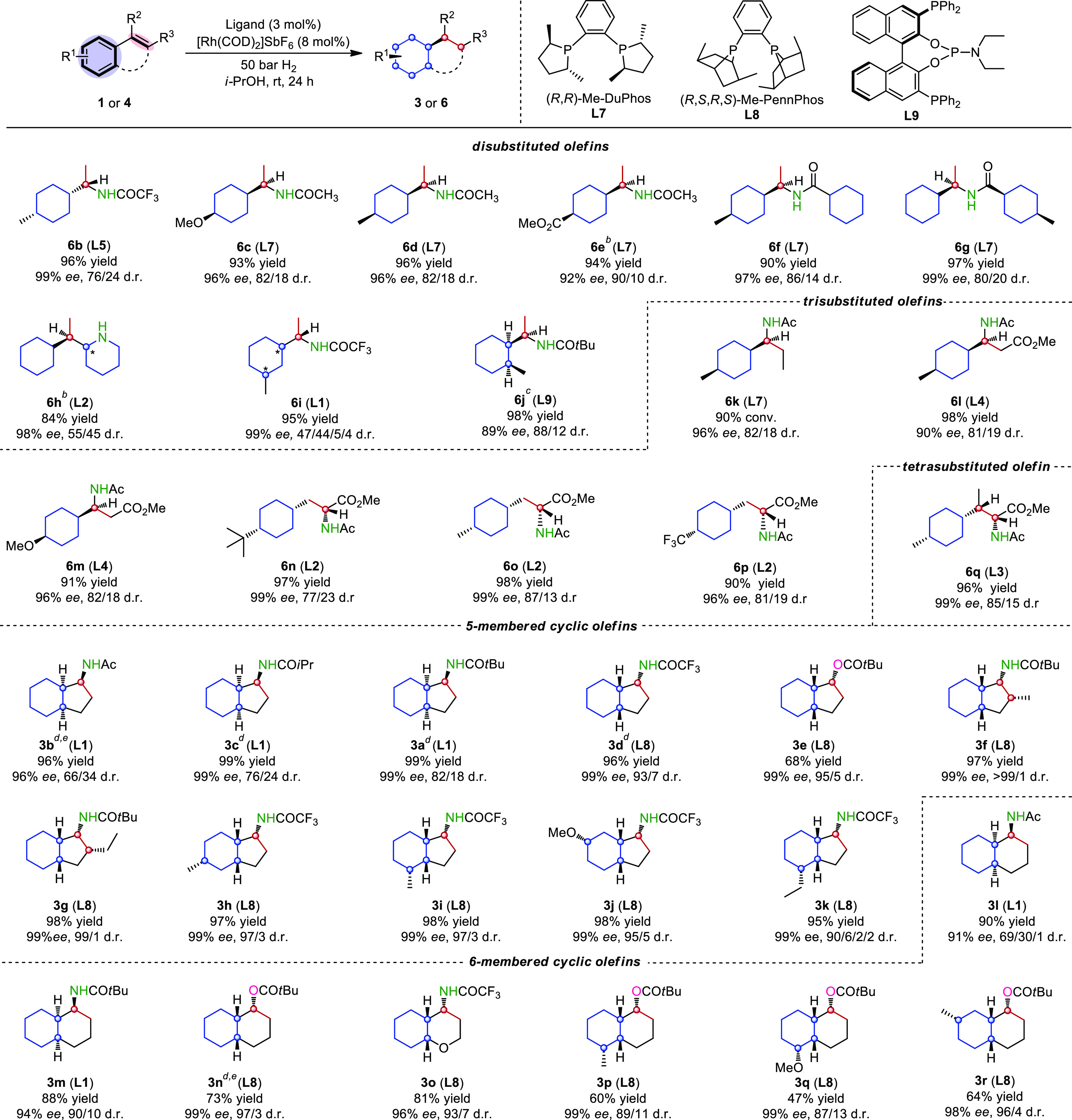
Evaluation of Substrate Classes Reaction conditions: substrate (0.2 mmol), 3 mol % ligand and 8 mol % [Rh(COD)_2_]SbF_6_ in *i*-PrOH (2.0 mL) under 50 bar H_2_ at room temperature for 24 h. Isolated yield. Enantiomeric excesses and diastereomeric ratios were determined by GC analysis using chiral stationary phase. Additional [Rh(COD)_2_]SbF_6_ (5 mol %) was added after 12 h, then 50 bar H_2_ for 12 h. 2 mol % ligand and 2 mol % [Rh(COD)_2_]SbF_6_ were used in CF_3_CH_2_OH (2.0 mL) under 10 bar H_2_ for 12 h, then 5 mol % [Rh(COD)_2_]SbF_6_ was added under 50 bar H_2_ for 12 h. 10 bar H_2_. [Rh(COD)_2_]BF_4_ was used instead of [Rh(COD)_2_]SbF_6_.

With the proof of concept established, various acyclic and cyclic substituted benzene substrate classes including di-, tri-, and tetra-substituted olefins were evaluated (Scheme 1). Starting with acyclic terminal olefins, electron-donating groups and electron-withdrawing groups in the *para*-position were found to be compatible and the corresponding fully saturated acetamides (**6b**-**e**) were formed in excellent yield with 92–99% *ee* and 76/24–90/10 d.r.. Both arenes were reduced with excellent enantioselectivity when benzamide derived enamides **4f**–**g** (80/20–86/14 d.r.) or pyridine-substituted olefin **4h** (55/45 d.r.) were hydrogenated. The *meta*-substituted substrate **4i** was fully reduced in 99% *ee* but with a poor diastereoselectivity. The *ortho*-substituted terminal enamide **4j** was also evaluated, however, this substrate was found to be much slower in the olefin hydrogenation step. It was known^40^ that the enantiocontrol in the hydrogenation of *ortho*-substituted terminal enamides is challenging and requires specialized ligands, 89% *ee* and 88/12 d.r. were achieved by using a bidentate phosphine-phosphoramidite ligand **L9**(41) with a two-step procedure. Notably, this strategy might be further applied as a possible alternative to the chiral auxiliary approaches.^42−44^ Then, numerous trisubstituted olefins that bear the prochiral center either in the benzylic or homobenzylic position were hydrogenated and formed the desired products in high stereoselectivities and yields (**6k**-**p**). To our delight, tetrasubstituted enamide **4q** was also hydrogenated in high enantioselectivity. The diastereoselectivity in the heterogeneous dearomative hydrogenation is highly substrate dependent. Encouraged by the excellent enantioselectivities obtained, we proceeded in evaluating substrates bearing cyclic olefins that have been reduced in higher diastereoselectivity in previous studies.^45,46^ Different nitrogen- and oxygen 3-substituted 1*H*-indenes were well tolerated (**1a**-**e**). The facial selectivity in the hydrogenation of the cyclic arene is sterically controlled, and as a result more sterically demanding groups in the benzylic position ensured higher diastereomeric ratio (**3a**-**c**) (from 66/34 to 82/18). Interestingly, trifluoromethyl-enamides **3d** were found to induce even higher diastereoselecitvities (93/7). Indenes bearing a tetrasubstituted olefins (**1f**-**g**) as well as a substitution in the 4-, 5- or 6-position (**1h**-**k**) were also smoothly hydrogenated and produced products with 4 stereogenic centers in excellent stereocontrol (up to 99/1 d.r. and 99% *ee*). Diverse six-membered fused arenes **1i**-**r** yielded the desired product in high isolated yields and good stereoselectivities. Carbon–oxygen bond cleavage was observed during the hydrogenation of cyclic enol-esters that accounted for the decreased yields.

### Scale-up Asymmetric Full Saturation of Vinylarene and Applications

**Scheme 2:**
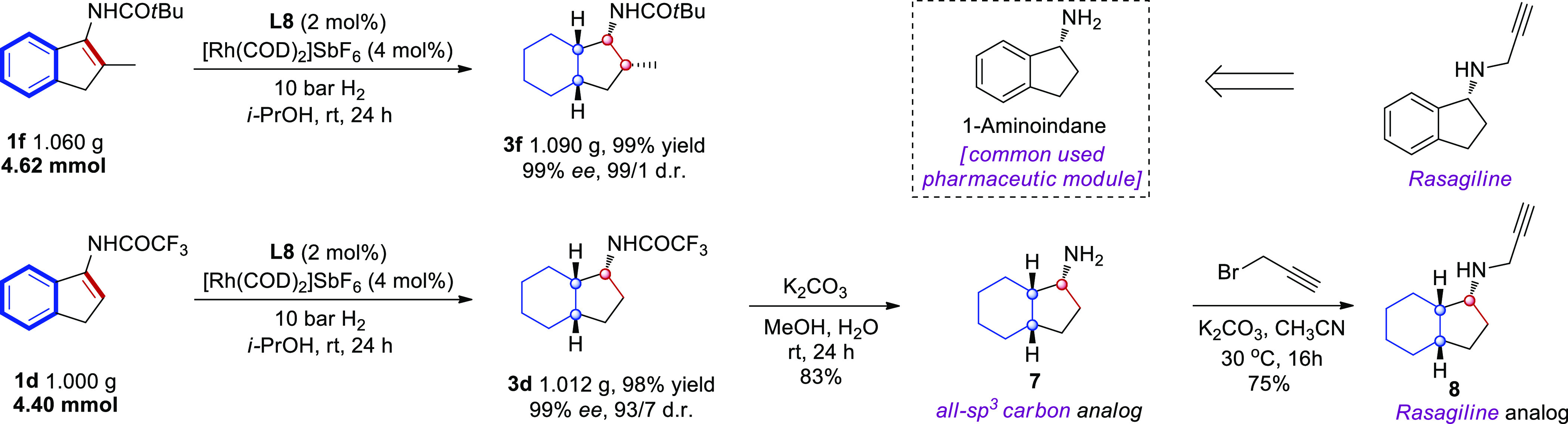
Gram Scale Reaction and Applications

The hydrogenation of **1f** was performed on a gram-scale to produce **3f** in high yield as a single stereoisomer, showing the scalability of this protocol (Scheme 2). Compound **1d** could also be hydrogenated on a gram-scale and consecutive cleavage of the amide group provided **7** as an all-sp^3^ carbon analogue to the widely used 1-aminoindane motif in pharmaceuticals.^47^ The reaction of **7** with propargyl bromide yielded the arene saturated analogue **8** to the *anti*-Parkinson’s therapeutic Rasagiline^48^ in good yield. Analysis of large data sets on medicinal bioisosters in previous literature correlates both increased sp^3^-content and the number of stereogenic centers with the enhanced likelihood that lead compounds proceed to clinical trials.^49,50^ Given this knowledge, the methodology presented herein can grant access to chiral cyclohexane scaffolds and thus positively influence both factors in an efficient and atom-economical way.^24,51^

## Conclusions

In summary, the results presented herein suggest that the developed protocol shows potential for application of the well-established classical homogeneous rhodium catalytic system to the formidable asymmetric arene hydrogenation and demonstrate the power of merging homogeneous and heterogeneous catalysis in organic synthesis. It is also anticipated that this practical dearomative hydrogenation will be of interest to the pharmaceutical industry for the rapid buildup of the complexity in the lead compounds from abundant aromatic feedstocks.

## Supporting Information Available

Materials, experimental procedures, characterization data for all new compounds, assignment of the absolute configurations of complete hydrogenated products, additional experiments, HR-TEM images of *in situ* generated Rh-nanoparticles, and NMR and GC spectra (PDF)
