# Supplementary material for: Asymmetric Full Saturation of Vinylarenes with Cooperative Homogeneous and Heterogeneous Rhodium Catalysis
Source: J Am Chem Soc. 2021 Nov 22;143(48):20377–83. doi: 10.1021/jacs.1c09975 (PMC8662739; doi:10.1021/jacs.1c09975)
Supplement: Supplementary file 1 — ja1c09975_si_001.pdf [file ja1c09975_si_001.pdf]

# Asymmetric Full Saturation of Vinylarenes with Cooperative Homogeneous and Heterogeneous Rhodium Catalysis

Haibo Wu,[1 §] Jianping Yang,[1 §] Bram B. C. Peters,[1] Luca Massaro,[1] Jia Zheng[1] and Pher G. Andersson[* 1,2]

[1]Department of Organic Chemistry, Stockholm University, Svante Arrhenius väg 16C, SE-10691 Stockholm, Sweden.

[2]School of Chemistry and Physics, University of Kwazulu-Natal, Private Bag X54001, Durban, 4000, South Africa.

§These authors contributed equally to this work.

*Corresponding author. Email: pher.andersson@su.se.

Contents

1. General methods ........................................................................................ S2

2. Preparation of vinylarenes ........................................................................ S3

3. Development of arene hydrogenation based on Rh/diphosphine system and kinetic studies ........................................................................................................ S10

4. Evaluation of Rh-precursors and the generality of diphosphine ligand............... S11

5. General procedure for asymmetric hydrogenation of arenes ............................... S12

6. Assignment of the absolute configurations of hydrogenated products. ................ S29

7. Scale-up asymmetric hydrogenations and applications ....................................... S30

8. Additional experiments and HR-TEM images of *in-situ* generated Rh-nanoparticles. ........................................................................................ S32

9. $^{1}$H, $^{13}$C and $^{1}$F NMR spectra for new compounds ............................................... S35

10. GC Chromatograms for hydrogenated products .............................................. S110

11. References.................................................................................................. S121

## 1. General methods

Unless otherwise noted, all reactions were conducted under dry nitrogen or argon atmosphere using magnetic stirring. *i*PrOH was freshly distilled from magnesium under nitrogen $CH_2Cl_2$ was freshly distilled from magnesium under nitrogen; THF was freshly distilled from Na and benzophenone under argon; All other solvents and reagents were purchased from commercial suppliers and used without further purification. Chromatographic separations were performed on Kiesel gel 60 H silica gel (particle) size: 0.063-0.100 mm). Thin layer chromatography (TLC) was performed on aluminium plates coated with Kieselgel 60 (0.20 mm, UV254) and visualized under ultraviolet light (ν = 254 nm), or by staining with ethanolic phosphomolybdic acid and heating. $^{1}H$ NMR spectra were recorded at 400 MHz in Chloroform-*d* at 25 ℃ and referenced internally to the residual $CHCl_3$ peak (7.26 ppm) or were recorded at 400 MHz in DMSO-$d_6$ at 25 ℃ and referenced internally to the residual DMSO peaks (2.50 ppm). $^{13}C$ NMR spectra were recorded at 100 MHz in Chloroform-*d* at 25 ℃ and referenced to the central peak of Chloroform-*d* (77.16 ppm) or were recorded at 100 MHz in DMSO-$d_6$ at 25 ℃ and referenced to the central peak of DMSO (39.52 ppm). $^{19}F$ NMR spectra were recorded at 377 MHz in Chloroform-*d or* DMSO-$d_6$ at 25 ℃. Chemical shifts are reported in ppm (δ scale). Optical rotations were recorded on a thermostated polarimeter using a sodium lamp (589 nm) and a 1.0 dm cell. Enantiomeric excesses and diastereomeric ratios were determined using GC (30 m columns, Helium gas carrier at 1 mL/min, constant pressure) with a MS detector. Racemic compounds were used for comparison. HRMS was performed on a Bruker microTOF with an ESI source.

Ligands **L1**-**L7** were used as purchased from Strem Chemicals Inc. and Merck-Sigma Aldrich. Ligand **L8** [(*R*,*S*,*R*,*S*)-Me-PennPhos] and **L9** were synthesized according to the literature.[1]

$[Rh(COD)_2]PF_6$ was synthesized according to the literature.[2]

$[Rh(COD)_2]BAr_F$ was synthesized according to the literature.[3]

The other rhodium precursors were used as purchased from Merck-Sigma Aldrich and Strem Chemicals Inc.

## 2. Preparation of vinylarenes

### General procedure for the synthesis of synthesis of enamides

Method A (for acetal-amides):

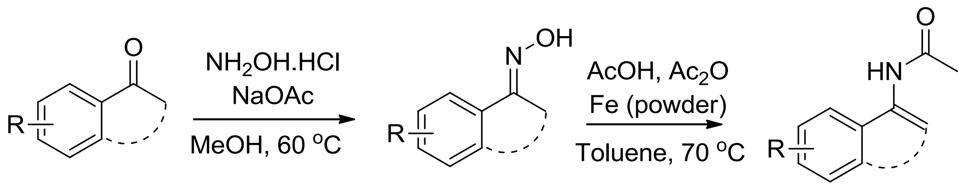


Method B (for the other enamides):

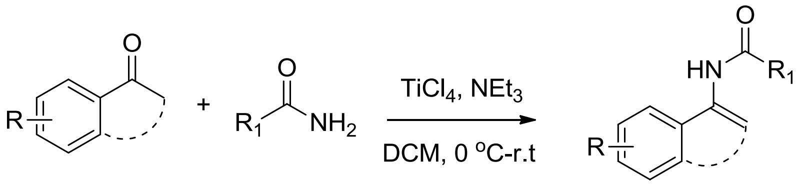


Acetal-enamides **4c**, **4d**, **4k**, **1b**, **1j** were prepared following method A, which was carried out according to the literature.[4] The other enamides **4b**, **4f**, **4g**, **4i**, **4j**, **1a**, **1c**, **1d**, **1e**, **1f**, **1g**, **1l** were prepared following method B, which was carried out according to the literature.[5]

Characterization for the following compounds have been previously reported: **4f**,[6] **4g**,[6] **4k**,[7] **1k**,[8] **1l**.[8]

New compounds:

### 2,2,2-trifluoro-*N*-(1-(*p*-tolyl)vinyl)acetamide

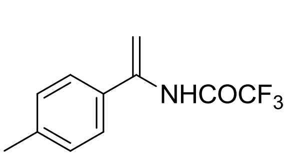


White Solid. $^1$H NMR (400 MHz, DMSO-$d_6$) δ 10.9 (s, *br*, 1H), 7.3 (d, *J* = 8.07 Hz, 2H), 7.2 (d, *J* = 8.03 Hz, 2H), 5.5 (s, 1H), 5.4 (s, 1H), 2.3 (s, 3H). $^{13}$C NMR (100 MHz, DMSO-$d_6$) δ 155.4 (q, *J* = 36.81 Hz), 139.1, 138.3, 133.0, 129.1, 125.6, 115.9 (q, *J* = 288.61 Hz), 108.8, 20.7. $^{19}$F NMR (377 MHz, DMSO-$d_6$) δ -73.95. HRMS-ESI: Found $[M+Na]^+$ = 252.0628; $C_{11}H_{10}F_3NONa$ requires 252.0607.

**2,2,2-trifluoro-*N*-(1-(*m*-tolyl)vinyl)acetamide**

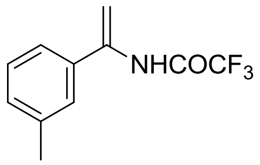


Colorless oil. $^1$H NMR (400 MHz, DMSO-$d_6$) δ 10.87 (s, *br*, 1H), 7.33 – 7.25 (m, 2H), 7.25 – 7.17 (m, 2H), 5.56 (s, 1H), 5.46 (s, 1H), 2.33 (s, 3H). $^{13}$C NMR (100 MHz, DMSO-$d_6$) δ 155.4 (q, *J* = 36.73 Hz), 139.2, 137.7, 135.9, 129.4, 128.4, 126.3, 122.9, 115.6 (q, *J* = 288.98 Hz), 109.3, 21.0. $^{19}$F NMR (377 MHz, DMSO-$d_6$) δ -73.94. **HRMS-ESI**: Found $[M+Na]^+$ = 252.0609; $C_{11}H_{10}F_3NONa$ requires 252.0607.

***N*-(1-(*o*-tolyl)vinyl)pivalamide**

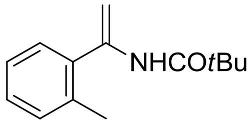


White solid. $^1$H NMR (400 MHz, DMSO-$d_6$) δ 8.79 (s, *br*, 1H), 7.27 – 7.14 (m, 4H), 5.68 (s, 1H), 4.53 (s, 1H), 2.25 (s, 3H), 1.14 (s, 9H). $^{13}$C NMR (100 MHz, DMSO-$d_6$) δ 176.4, 142.4, 139.2, 135.3, 129.9, 128.9, 127.7, 125.5, 103.3, 39.1, 27.1, 19.5. **HRMS-ESI**: Found $[M+Na]^+$ = 240.1379; $C_{14}H_{19}NONa$ requires 240.1359.

**4-methyl-*N*-(1-phenylvinyl)benzamide**

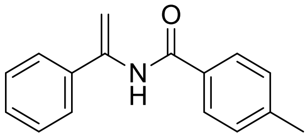


White solid. $^1$H NMR (400 MHz, DMSO-$d_6$) δ 9.82 (s, *br*, 1H), 7.88 (d, *J* = 5.51 Hz, 2H), 7.49 (d, *J* = 4.09 Hz, 2H), 7.42 – 7.27 (m, 5H), 5.52 (d, *J* = 4.94 Hz, 1H), 5.29 (d, *J* = 2.13 Hz, 1H), 2.37 (s, 3H). $^{13}$C NMR (100 MHz, DMSO-$d_6$) δ 165.8, 141.9, 141.5, 138.0, 131.7, 128.9, 128.3, 128.1, 127.8, 126.0, 105.7, 21.0. **HRMS-ESI**: Found $[M+Na]^+$ = 260.1057; $C_{16}H_{15}NONa$ requires 260.1046.

***N*-(1-(*p*-tolyl)vinyl)benzamide**

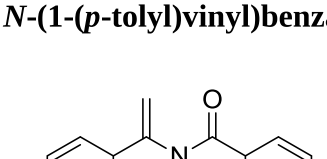


White solid. $^1$H NMR (400 MHz, DMSO-$d_6$) δ 9.82 (s, *br*, 1H), 7.94 (dd, *J* = 7.09, 1.46 Hz, 2H), 7.61 – 7.53 (m, 1H), 7.53 – 7.45 (m, 2H), 7.43 – 7.34 (m, 2H), 7.18 (d, *J* = 7.91 Hz, 2H), 5.46 (s, 1H), 5.27 (s, 1H), 2.31 (s, 3H). $^{13}$C NMR (100 MHz, DMSO-*d*6) δ 165.9, 141.7, 137.5, 135.1, 134.5, 131.5, 128.8, 128.3, 127.7, 125.8, 105.1, 20.7. **HRMS-ESI**: Found $[M+Na]^+$ = 260.1063; $C_{16}H_{15}NONa$ requires 260.1046.

**_N_-(1_H_-inden-3-yl)isobutyramide**

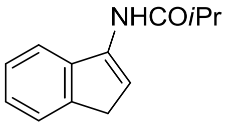


White solid. $^{1}$H NMR (400 MHz, Chloroform-*d*) δ 9.61 (s, *br*, 1H), 7.78 (d, *J* = 7.60 Hz, 1H), 7.46 (d, *J* = 7.32 Hz, 1H), 7.32 (td, *J* = 7.55, 0.98 Hz, 1H), 7.22 (td, *J* = 7.39, 0.94 Hz, 1H), 6.76 (t, *J* = 2.31 Hz, 1H), 3.37 (d, *J* = 2.33 Hz, 2H), 2.81 (hept, *J* = 6.79 Hz, 1H), 1.11 (d, *J* = 6.81 Hz, 6H). $^{13}$C NMR (100 MHz, Chloroform-*d*) δ 175.9, 142.3, 140.1, 136.7, 125.7, 125.0, 123.8, 118.3, 114.1, 35.9, 34.0, 19.6. **HRMS-ESI**: Found $[M+Na]^+$ = 224.1046; $C_{13}H_{15}NONa$ requires 224.1067.

**_N_-(1_H_-inden-3-yl)pivalamide**

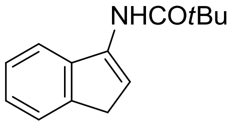


White solid. $^{1}$H NMR (400 MHz, DMSO-$d_6$) δ 8.90 (s, *br*, 1H), 7.67 (d, *J* = 7.49 Hz, 1H), 7.46 (d, *J* = 7.25 Hz, 1H), 7.31 (t, *J* = 7.38 Hz, 1H), 7.26 – 7.15 (m, 1H), 6.65 (t, *J* = 2.37 Hz, 1H), 3.37 (d, *J* = 1.88 Hz, 2H), 1.27 (s, 9H). $^{13}$C NMR (100 MHz, DMSO-$d_6$) δ 176.8, 142.4, 140.4, 136.9, 125.6, 125.0, 123.8, 118.8, 116.6, 39.1, 35.8, 27.3. **HRMS-ESI**: Found $[M+Na]^+$ = 238.1230; $C_{14}H_{17}NONa$ requires 238.1202.

**2,2,2-trifluoro-_N_-(1_H_-inden-3-yl)acetamide**

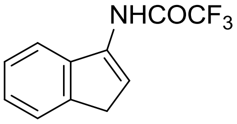


White solid. $^{1}$H NMR (400 MHz, Chloroform-*d*) δ 8.05 (s, *br*, 1H), 7.52 (dd, *J* = 7.39, 2.52 Hz, 1H), 7.40 – 7.31 (m, 2H), 7.30 – 7.25 (m, 1H), 6.98 (t, *J* = 2.47 Hz, 1H), 3.48 (d, *J* = 2.52 Hz, 2H). $^{13}$C NMR (100 MHz, Chloroform-*d*) δ 155.2 (q, *J* = 38.72 Hz), 142.6, 138.5, 133.4, 126.5, 126.3, 124.7, 119.8, 116.2, 115.8 (q, *J* = 261.51 Hz), 36.9. $^{19}$F NMR (377 MHz, Chloroform-*d*) δ -75.54. **HRMS-ESI**: Found $[M+Na]^+$ = 250.0468; $C_{11}H_8F_3NONa$ requires 250.0450.

**_N_-(2-methyl-1_H_-inden-3-yl)pivalamide**

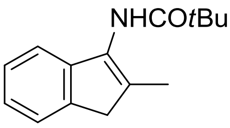


White solid. $^{1}$H NMR (400 MHz, Chloroform-*d*) δ 7.34 (d, *J* = 7.32 Hz, 1H), 7.23 (t, *J* = 7.58 Hz, 1H), 7.13 (td, *J* = 7.43, 1.17 Hz, 1H), 7.08 (d, *J* = 7.51 Hz, 1H), 3.34 (s, 2H), 2.03 (s, 3H), 1.38 (s, 9H). $^{13}$C NMR (100 MHz, Chloroform-*d*) δ 177.1, 142.8, 141.1, 136.1, 131.6, 126.3, 124.5, 123.6, 117.7, 40.9, 39.5, 28.0, 14.2. **HRMS-ESI**: Found $[M+Na]^+$ = 252.1386; $C_{15}H_{19}NONa$ requires 252.1376.

**N-(2-ethyl-1H-inden-3-yl)pivalamide**

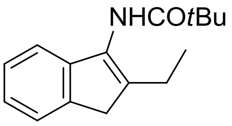


White solid. $^1$H NMR (400 MHz, Chloroform-*d*) δ 7.36 (d, *J* = 7.36 Hz, 1H), 7.23 (d, *J* = 7.53 Hz, 1H), 7.14 (td, *J* = 7.41, 1.20 Hz, 1H), 7.10 (dd, *J* = 7.46, 0.98 Hz, 1H), 3.35 (d, *J* = 2.55 Hz, 2H), 2.44 (q, *J* = 15.26 Hz, 2H), 1.37 (s, 9H), 1.17 (t, *J* = 7.61 Hz, 3H). $^{13}$C NMR (100 MHz, Chloroform-*d*) δ 177.1, 142.8, 141.9, 141.2, 130.8, 126.3, 124.5, 123.7, 117.9, 39.5, 38.2, 28.0, 21.6, 13.4. **HRMS-ESI**: Found $[M+Na]^+$ = 266.1528; $C_{16}H_{21}NONa$ requires 266.1515.

**2,2,2-trifluoro-N-(6-methyl-1H-inden-3-yl)acetamide**

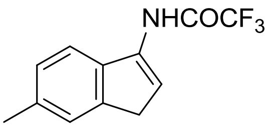


White solid. $^1$H NMR (400 MHz, Chloroform-*d*) δ 7.91 (s, *br*, 1H), 7.34 (s, 1H), 7.16 (q, *J* = 7.81 Hz, 2H), 6.93 (t, *J* = 2.35 Hz, 1H), 3.47 (d, *J* = 2.38 Hz, 2H), 2.43 (s, 3H). $^{13}$C NMR (100 MHz, Chloroform-*d*) δ 148.2 (q, *J* = 51.18 Hz), 142.9, 136.3, 135.9, 133.3, 127.2, 125.7, 118.6, 115.7, 114.5 (q, *J* = 286.89 Hz), 36.7, 21.6. $^{19}$F NMR (377 MHz, Chloroform-*d*) δ -75.54. **HRMS-ESI**: Found $[M+Na]^+$ = 264.0626; $C_{12}H_{10}F_3NONa$ requires 264.0607.

**2,2,2-trifluoro-N-(7-methyl-1H-inden-3-yl)acetamide**

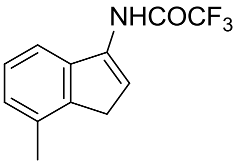


White solid. $^1$H NMR (400 MHz, Chloroform-*d*) δ 7.94 (s, *b*r, 1H), 7.29 (dd, *J* = 15.35, 7.76 Hz, 1H), 7.12 (dd, *J* = 9.99, 7.53 Hz, 2H), 7.01 (t, *J* = 2.49 Hz, 1H), 3.40 (s, 2H), 2.40 (s, 3H). $^{13}$C NMR (100 MHz, Chloroform-*d*) δ 155.1 (q, *J* = 34.80 Hz), 141.2, 138.1, 134.1, 133.6, 127.6, 126.9, 119.2, 115.9 (q, *J* = 288.64 Hz), 113.7, 35.9, 18.5. $^{19}$F NMR (377 MHz, Chloroform-*d*) δ -75.51. **HRMS-ESI**: Found $[M+Na]^+$ = 264.0622; $C_{12}H_{10}F_3NONa$ requires 264.0607.

**2,2,2-trifluoro-N-(5-methoxy-1H-inden-3-yl)acetamide**

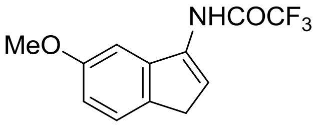


White solid. $^1$H NMR (400 MHz, Chloroform-*d*) δ 7.90 (s, *br*, 1H), 7.39 (d, *J* = 8.20 Hz, 1H), 7.01 (t, *J* = 2.40 Hz, 1H), 6.85 (dd, *J* = 8.24, 2.27 Hz, 1H), 6.78 (d, *J* = 2.24 Hz, 1H), 3.86 (s, 3H), 3.43 (d, *J* = 2.32 Hz, 2H). $^{13}$C NMR (100 MHz, Chloroform-*d*) δ 159.1, 155.1 (q, *J* = 38.27 Hz), 139.8, 134.6, 133.2, 125.2, 121.3, 115.9 (q, *J* = 289.60 Hz),

111.7, 102.6, 55.8, 36.2. $^{19}$F NMR (377 MHz, Chloroform-*d*) δ -75.5. **HRMS-ESI**: Found $[M+Na]^+$ = 280.0569; $C_{12}H_{10}F_3NO_2Na$ requires 280.0556.

***N*-(7-ethyl-1*H*-inden-3-yl)-2,2,2-trifluoroacetamide**

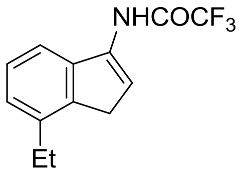


White solid. $^{1}$H NMR (400 MHz, Chloroform-*d*) δ 7.97 (s, *br*, 1H), 7.34 (t, *J* = 7.58 Hz, 1H), 7.18 (d, *J* = 7.56 Hz, 1H), 7.12 (dd, *J* = 7.58, 0.94 Hz, 1H), 7.00 (t, *J* = 2.41 Hz, 1H), 3.44 (d, *J* = 2.43 Hz, 2H), 2.76 (q, *J* = 7.61 Hz, 2H), 1.30 (t, *J* = 7.61 Hz, 3H). $^{13}$C NMR (100 MHz, Chloroform-*d*) δ 155.1 (d, *J* = 37.73 Hz), 140.5, 140.2, 138.3, 133.6, 127.1, 125.8, 119.1, 115.7 (q, *J* = 288.82 Hz), 113.7, 35.5, 25.8, 14.3. $^{19}$F NMR (377 MHz, Chloroform-*d*) δ -75.53. **HRMS-ESI**: Found $[M+Na]^+$ = 278.0784; $C_{13}H_{12}F_3NONa$ requires 278.0763.

***N*-(3,4-dihydronaphthalen-1-yl)pivalamide**

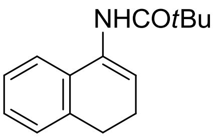


White solid. $^{1}$H NMR (400 MHz, DMSO-$d_6$) δ 8.72 (s, br, 1H), 7.16 (d, *J* = 6.35 Hz, 3H), 7.03 (d, *J* = 6.21 Hz, 1H), 5.94 (t, *J* = 4.28 Hz, 1H), 2.71 (t, *J* = 7.93 Hz, 2H), 2.33 – 2.23 (m, 2H), 1.22 (s, 9H). $^{13}$C NMR (100 MHz, DMSO-$d_6$) δ 176.9, 135.8, 132.9, 132.6, 127.3, 127.0, 126.1, 122.1, 121.9, 38.7, 27.4, 27.2, 21.9. **HRMS-ESI**: Found $[M+Na]^+$ = 252.1367; $C_{15}H_{19}NONa$ requires 252.1359.

***N*-(2*H*-chromen-4-yl)-2,2,2-trifluoroacetamide**

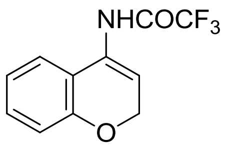


White solid. $^{1}$H NMR (400 MHz, Chloroform-*d*) δ 7.53 (s, *br*, 1H), 7.28 – 7.20 (m, 1H), 7.00 (dtd, *J* = 14.89, 7.66, 1.51 Hz, 2H), 6.91 (dd, *J* = 8.15, 1.12 Hz, 1H), 6.50 (t, *J* = 4.20 Hz, 1H), 4.86 (d, *J* = 4.26 Hz, 2H). $^{13}$C NMR (100 MHz, Chloroform-*d*) δ 155.7 (q, *J* = 37.41 Hz), 155.3, 130.8, 127.0, 121.8, 119.8, 118.9, 117.3, 115.7 (q, *J* = 289.02 Hz), 112.2, 64.7. $^{19}$F NMR (377 MHz, Chloroform-*d*) δ -75.73. **HRMS-ESI**: Found $[M+Na]^+$ = 266.0405; $C_{11}H_8F_3NO_2Na$ requires 266.0399.

***N*-(2*H*-chromen-4-yl)pivalamide**

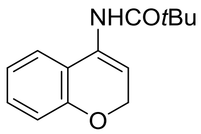


White solid. $^{1}$H NMR (400 MHz, Chloroform-*d*) δ 8.71 (s, *br*, 1H), 7.16 (td, *J* = 7.83, 1.62 Hz, 1H), 7.06 (dd, *J* = 7.67, 1.62 Hz, 1H), 6.92 (td, *J* = 7.51, 1.08 Hz, 1H), 6.81 (dd, *J* = 8.02, 1.04 Hz, 1H),

5.90 (t, *J* = 4.27 Hz, 1H), 4.77 (d, *J* = 3.98 Hz, 2H), 1.22 (s, 9H). $^{13}$C NMR (100 MHz, DMSO-$d_6$) δ 177.1, 154.5, 129.8, 129.2, 122.8, 121.4, 121.0, 115.7, 114.2, 64.6, 39.0, 27.3. **HRMS-ESI**: Found $[M+Na]^+$ = 254.1159; $C_{14}H_{17}NO_2Na$ requires 254.1151.

**General procedure for the synthesis of synthesis of enol-pivalates**

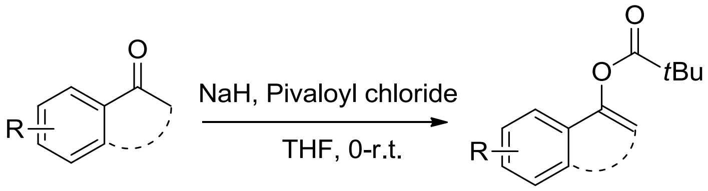


Enol-pivalates **1e**, **1n**, **1p**, **1q**, **1r** were synthesized according to the reported procedures.[9]

New compounds:

**1*H*-inden-3-yl pivalate**

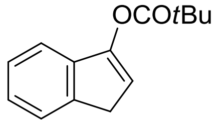


White solid. $^1$H NMR (400 MHz, Chloroform-*d*) δ 7.45 (s, *br*, 1H), 7.36 – 7.28 (m, 2H), 7.30 – 7.21 (m, 1H), 6.33 (t, *J* = 2.32 Hz, 1H), 3.42 (d, *J* = 2.08 Hz, 2H), 1.40 (s, 9H). $^{13}$C NMR (100 MHz, Chloroform-*d*) δ 175.9, 149.3, 141.9, 139.5, 126.4, 125.8, 124.3, 118.0, 115.3, 39.6, 35.2, 27.4. **HRMS-ESI**: Found $[M+Na]^+$ = 239.1062; $C_{14}H_{16}O_2Na$ requires 239.1043.

**3,4-dihydronaphthalen-1-yl pivalate**

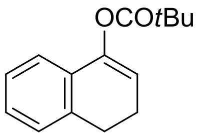


White solid. $^1$H NMR (400 MHz, Chloroform-*d*) δ 7.21 – 7.12 (m, 3H), 7.11 – 7.06 (m, 1H), 5.68 (t, *J* = 4.67 Hz, 1H), 2.87 (t, *J* = 8.10 Hz, 2H), 2.45 (td, *J* = 8.07, 4.67 Hz, 2H), 1.39 (s, 9H). $^{13}$C NMR (100 MHz, Chloroform-*d*) δ 176.9, 145.9, 136.6, 130.9, 127.9, 127.7, 126.5, 120.7, 115.2, 39.3, 27.7, 27.4, 22.2. **HRMS-ESI**: Found $[M+Na]^+$ = 253.1206; $C_{15}H_{18}O_2Na$ requires 253.1199.

**5-methyl-3,4-dihydronaphthalen-1-yl pivalate**

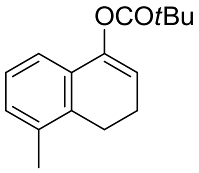


White solid. $^1$H NMR (400 MHz, Chloroform-*d*) δ 7.11 – 7.02 (m, 2H), 7.00 – 6.93 (m, 1H), 5.65 (t, *J* = 4.69 Hz, 1H), 2.81 (t, *J* = 8.20 Hz, 2H), 2.44 (ddd, *J* = 9.13, 7.60, 4.71 Hz, 2H), 2.28 (s, 3H), 1.37 (s, 9H). $^{13}$C NMR (100 MHz, Chloroform-d) δ 177.0, 146.0, 135.2,

134.9, 130.7, 130.1, 125.8, 118.7, 114.6, 39.3, 27.5, 23.6, 21.8, 19.8. **HRMS-ESI**: Found $[M+Na]^+$ = 267.1349; $C_{16}H_{20}O_2Na$ requires 267.1356.

**5-methoxy-3,4-dihydronaphthalen-1-yl pivalate**

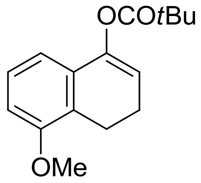


White solid. $^1$H NMR (400 MHz, Chloroform-*d*) δ 7.12 (t, *J* = 7.96, 7.50 Hz, 1H), 6.80 (dd, *J* = 8.31, 1.06 Hz, 1H), 6.74 (d, *J* = 7.82 Hz, 1H), 5.66 (t, *J* = 4.67 Hz, 1H), 3.83 (s, 3H), 2.86 (t, *J* = 8.31 Hz, 2H), 2.42 (ddd, *J* = 9.36, 7.71, 4.69 Hz, 2H), 1.37 (s, 9H). $^{13}$C NMR (100 MHz, Chloroform-d) δ 177.0, 156.3, 145.7, 132.0, 130.7, 126.7, 124.6, 115.4, 113.6, 110.7, 55.7, 39.3, 27.5, 21.7, 19.8. **HRMS-ESI**: Found $[M+Na]^+$ = 283.1305; $C_{16}H_{20}O_3Na$ requires 283.1305.

**7-methyl-3,4-dihydronaphthalen-1-yl pivalate**

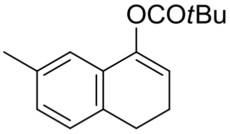


White solid. $^1$H NMR (400 MHz, Chloroform-*d*) δ 7.04 (d, *J* = 7.57 Hz, 1H), 6.98 (ddd, *J* = 7.56, 1.82, 0.82 Hz, 1H), 6.88 (t, *J* = 1.24 Hz, 1H), 5.65 (t, *J* = 4.69 Hz, 1H), 2.82 (t, *J* = 8.09 Hz, 2H), 2.47 – 2.39 (m, 2H), 2.29 (s, 3H), 1.38 (s, 9H). $^{13}$C NMR (100 MHz, Chloroform-d) δ 177.0, 146.0, 135.9, 133.6, 130.7, 128.5, 127.6, 121.4, 115.2, 39.3, 27.5, 27.3, 22.4, 21.5. **HRMS-ESI**: Found $[M+Na]^+$ = 267.1373; $C_{16}H_{20}O_2Na$ requires 267.1356.

**General procedure for the synthesis of the other substrates**

Substrate **4e**,[16] **4h**,[10] **4l**,[11] **4m**,[11] **4o**,[12] **4p**,[12] **4q**[13] were prepared according to the literature procedures, and the characterization data has been included.

Substrate **4n** was prepared according to the literature procedure.[13]

**Methyl (*Z*)-2-acetamido-3-(4-(*tert*-butyl)phenyl)acrylate**

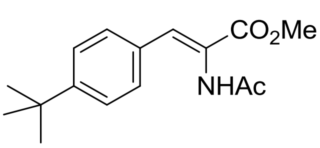


White solid. $^1$H NMR (400 MHz, DMSO-$d_6$) δ 9.59 (s, *br*, 1H), 7.57 (d, *J* = 8.55 Hz, 2H), 7.44 (d, *J* = 8.51 Hz, 2H), 7.16 (s, 1H), 3.70 (s, 3H), 1.99 (s, 3H), 1.28 (s, 9H). $^{13}$C NMR (100 MHz, DMSO-$d_6$) δ 169.3, 165.6, 152.2, 131.1, 130.6, 129.7, 126.0, 125.4, 52.1, 34.5, 30.9, 22.4. **HRMS-ESI**: Found $[M+Na]^+$ = 298.1420; $C_{16}H_{21}NO_3Na$ requires 298.1414.

## 3. Development of arene hydrogenation based on Rh/diphosphine system and kinetic studies

**Asymmetric olefin hydrogenation of 1a with homogeneous Rh catalyst (Rh/L=1:1).**

To a dry 4 mL vial was charged with $[Rh(COD)_2]BF_4$ (2 mol%) and diphosphine ligand (S,S)-Me-BPE (2 mol%, 0.5 mL stock solution in *i*-PrOH) under argon. The mixture was stirred for 0.5 h. The substrate **1a** (0.1 mmol) was dissolved in 0.5 mL *i*-PrOH and then added to the vial under argon. The vial was placed in a high-pressure hydrogenation apparatus. The reactor was purged three times with Ar, and then filled with $H_2$ (10 bar). The reaction was stirred at room temperature for 12 h before the $H_2$ pressure was released and the solvent was removed *in vacuo*. The crude product was filtered through a short plug of silica with 1:1 $Et_2O$/Pentane as the eluent to yield product **2a**.

**Complete hydrogenation of 1a with $[Rh(COD)_2]BF_4$.**

To a dry 4 mL vial was charged with $[Rh(COD)_2]BF_4$ (2 mol%), substrate **1a** (0.1 mmol) and 1 mL *i*-PrOH. The vial was placed in a high-pressure hydrogenation apparatus. The reactor was purged three times with Ar, and then filled with $H_2$ (10 bar). The reaction was stirred at room temperature for 12 h before the $H_2$ pressure was released and the solvent was removed *in vacuo*. The crude product was filtered through a short plug of silica with 1:1 $Et_2O$/Pentane as the eluent to yield the racemic product **3a**.

**Asymmetric full saturation of 1a with homogeneous Rh/diphosphine and in-situ generated heterogeneous Rh-nanoparticles (Rh/L=2:1).**

To a dry 4 mL vial was charged with $[Rh(COD)_2]BF_4$ (4 mol%) and diphosphine ligand (S,S)-Me-BPE (2 mol%, 0.5 mL stock solution in *i*-PrOH) under argon. The mixture was stirred for 0.5 h. The substrate **1a** (0.1 mmol) was dissolved in 0.5 mL *i*-PrOH and then added to the vial under argon. The vial was placed in a high-pressure hydrogenation apparatus. The reactor was purged three times with Ar, and then filled with $H_2$ (10 bar). The reaction was stirred at room temperature for 12 h before the $H_2$ pressure was released and the solvent was removed *in vacuo*. The crude product was

filtered through a short plug of silica with 1:1 $Et_2O$/Pentane as the eluent to yield the enantio-enriched full reduced product **3a**.

**Kinetic studies of olefin hydrogenation of 1a.**

To a set of three 4 mL dry vials was charged with the corresponding amount of $[Rh(COD)_2]BF_4$ and diphosphine ligand (S,S)-Me-BPE (2 mol%, 0.5 mL stock solution in *i*-PrOH) under argon. The mixture was stirred for 0.5 h. The substrate **1a** (0.1 mmol) was dissolved in 0.5 mL *i*-PrOH and then added to the vial under argon. The vial was placed in a high-pressure hydrogenation apparatus. The reactor was purged three times with Ar, and then filled with $H_2$ (10 bar). The reaction was stirred at room temperature for the allocated time before the $H_2$ pressure was released and the solvent was removed *in vacuo*. The distribution of products from the reaction mixture was determined by $^1H$ NMR spectroscopy.

**Kinetic studies of arene hydrogenation of *rac*-2a.**

To a set of three 4 mL dry vials was charged with the corresponding amount of $[Rh(COD)_2]BF_4$ and diphosphine ligand (S,S)-Me-BPE (2 mol%, 0.5 mL stock solution in *i*-PrOH) under argon. The mixture was stirred for 0.5 h. The substrate *rac*-**2a** (0.1 mmol) was dissolved in 0.5 mL *i*-PrOH and then added to the vial under argon. The vial was placed in a high-pressure hydrogenation apparatus. The reactor was purged three times with Ar, and then filled with $H_2$ (10 bar). The reaction was stirred at room temperature for the allocated time before the $H_2$ pressure was released and the solvent was removed *in vacuo*. The distribution of products from the reaction mixture was determined by $^1H$ NMR spectroscopy.

**4. Evaluation of Rh-precursors and the generality of diphosphine ligand.**

To a dry 4 mL vial was charged with the corresponding Rh-precursor and allocated amount of diphosphine ligand under argon. The mixture was stirred for 0.5 h. The substrate **4a** (0.05 mmol) was dissolved in 0.5 mL *i*-PrOH and then added to the vial under argon. The vial was placed in a high-pressure hydrogenation apparatus. The reactor was purged three times with Ar, and then filled with $H_2$ (15 bar). The reaction was stirred at room temperature for 16 h before the $H_2$ pressure was released and the solvent was removed *in vacuo*. The distribution of products from the reaction mixture

was determined by $^{1}H$ NMR spectroscopy. Enantiomeric excess was determined by GC using a chiral stationary phase.

## 5. General procedure for asymmetric hydrogenation of arenes

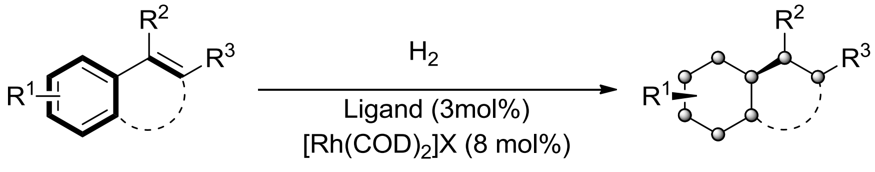


**1** or **4** → **3** or **6**

A vial was charged with Rh precursor (8 mol%) and chiral ligand (3 mol%) under Argon. Degassed dry *i*PrOH (2.0 ml) was added and the vial was stirred for 0.5 h. The substrates (0.2 mmol) were added under Argon (so that the concentration of the substrate was 0.1 M) and the vial was placed in a high-pressure hydrogenation apparatus. The reactor was purged three times with Ar gas, then filled with $H_2$. The reaction was stirred at room temperature for 24 h before the $H_2$ pressure was released and the solvent was removed *in vacuo*. The crude product was filtered through on a short plug of silica. Conversions were determined by $^{1}H$ NMR spectroscopy and *ee* values were determined by GC using a chiral stationary phase.

**Table S1.** Characterization data for fully hydrogenated products**.**

| Entry | Product | *d.r.* | Separation method | Optical rotation | *ee*/ % |
|---|---|---|---|---|---|
| 1 | 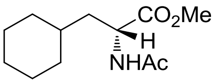  | -- | GC-MS: column Chiraldex β-DM, 50 °C to 175 ℃ at 1 ℃/min. $t_R$ = 89.6 min. | $[\alpha]_D^{29}$ = -16.0 (c = 0.2, $CHCl_3$) | 99 |
| 2 | 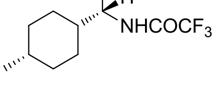  | 76:24 | GC-MS: column Chiraldex β-DM, 60 °C iso 180min. $t_R$ = 82.9 min/ 85.1 min (major) | –– | 99 |
| 3 | 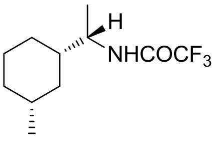  | 47:44:5:4 | GC-MS: column Chiraldex β-DM, 50 °C to 175 ℃ at 1 ℃/min. $t_R$ = 40.4 min/ 41.7 min/42.0 min/42.3 min. | –– | 99 |

| | | | | | |
|---|---|---|---|---|---|
| 4 | 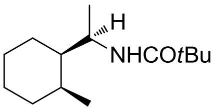 | 88:12 | GC-MS: column Chiraldex β-DM, 50 $^{o}$C to 175 ℃ at 1 ℃/min. $t_R$ = 72.0 min/ 73.2 min/73.6 min/74.7 min. | –– | 89 |
| 5 | 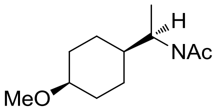 | 86:14 | GC-MS: column Chiraldex β-DM, 50 $^{o}$C to 175 ℃ at 1 ℃/min. $t_R$ = 66.0 min (minor)/ 81.0 min (major) | –– | 99 |
| 6 | 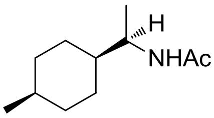 | 82:18 | GC-MS: column Chiraldex β-DM, 50 $^{o}$C to 175 ℃ at 1 ℃/min. $t_R$ = 66.7 min/ 67.8 min/69.0 min | –– | 96 |
| 7 | 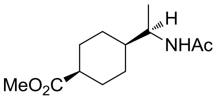 | 90:10 | GC-MS: column Chiraldex β-DM, 120 $^{o}$C iso at 1 ℃/min. $t_R$ = 32.1 min/ 33.2 min/57.0 min | –– | 92 |
| 8 | 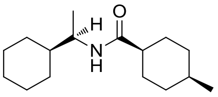 | 80:20 | GC-MS: column Chiraldex β-DM, 50 $^{o}$C to 175 ℃ at 1 ℃/min. $t_R$ = 77.8 min/ 108.2 min. | –– | 99 |
| 9 | 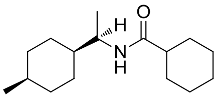 | 84:16 | GC-MS: column Chiraldex β-DM, 50 $^{o}$C to 175 ℃ at 1 ℃/min. $t_R$ = 64.5 min/ 65.1 min/66.9 min/66.3 min. | –– | 98 |
| 10 | 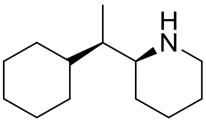 | 55:45 | GC-MS: column Chiraldex β-DM, 50 $^{o}$C to 175 ℃ at 1 ℃/min. $t_R$ = 64.5 min/ 65.1 min/66.9 min/66.3 min. | –– | 98 |
| 11 | 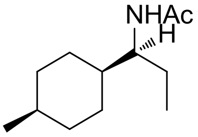 | 82:18 | GC-MS: column Chiraldex β-DM, 50 $^{o}$C to 175 ℃ at 1 ℃/min. $t_R$ = 71.6 min/ 72.8 min/75.1 min. | –– | 96 |

| | | | | | |
|---|---|---|---|---|---|
| 12 | 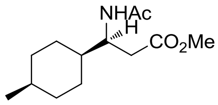 | 81:19 | GC-MS: column Chiraldex β-DM, 50 °C to 175 °C at 1 °C/min. $t_R$ = 94.5 min/ 95.1 min/96.7 min. | —— | 90 |
| 13 | 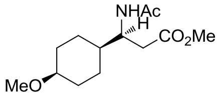 | 82:18 | GC-MS: column Chiraldex β-DM, 120 °C iso at 1 °C/min. $t_R$ = 111.5min/ 118.7 min/ 145.5 min. | —— | 96 |
| 14 | 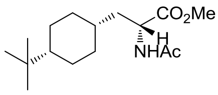 | 73:27 | GC-MS: column Chiraldex β-DM, 50 °C to 175 °C at 1 °C/min. $t_R$ = 114.3 min / 117.4 min. | —— | 99 |
| 15 | 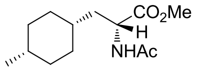 | 83:17 | GC-MS: column Chiraldex β-DM, 50 °C to 175 °C at 1 °C/min. $t_R$ = 92.5 min / 94.6 min. | —— | 99 |
| 16 | 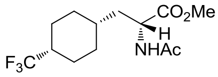 | 81:19 | GC-MS: column Chiraldex β-DM, 50 °C to 175 °C at 1 °C/min. $t_R$ = 92.1 min / 92.4 min /93.6 min. | —— | 96 |
| 17 | 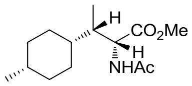 | 85:15 | GC-MS: column Chiraldex β-DM, 50 °C to 175 °C at 1 °C/min. $t_R$ = 94.4 min / 96.1 min. | —— | 99 |
| 18 | 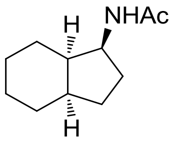 | 66:34 | GC-MS: column Chiraldex β-DM, 50 °C to 175 °C at 1 °C/min. $t_R$ = 81.1 min/82.6 min/83.7 min/85.3 min. | —— | 96 |
| 19 | 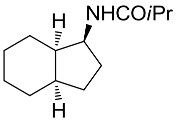 | 76:24 | GC-MS: column Chiraldex β-DM, 50 °C to 175 °C at 1 °C/min. $t_R$ = 59.8 min/ 72.5 min. | —— | 99 |

| | | | | | |
|---|---|---|---|---|---|
| 20 | 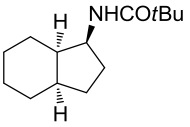 | 82:18 | GC-MS: column Chiraldex β-DM, 50 $^{o}$C to 175 ℃ at 1 ℃/min. $t_R$ = 83.0 min/ 87.9 min. | –– | 99 |
| 21 | 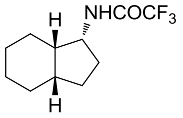 | 93:7 | GC-MS: column Chiraldex β-DM, 50 $^{o}$C to 175 ℃ at 1 ℃/min. $t_R$ = 56.7 min / 58.2 min. | $[\alpha]_D^{29}$ = 76.5 (c = 0.2, $CHCl_3$) | 99 |
| 22 | 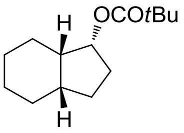 | 95:5 | GC-MS: column IVADEX-1, 50 $^{o}$C to 175 ℃ at 1 ℃/min. $t_R$ = 73.2 min / 79.1 min. | $[\alpha]_D^{29}$ = 17.5 (c = 0.2, $CHCl_3$) | 99 |
| 23 | 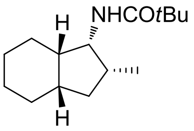 | >99:1 | GC-MS: column Chiraldex β-DM, 50 $^{o}$C to 175 ℃ at 1 ℃/min. $t_R$ = 89.6 min. | $[\alpha]_D^{29}$ = -4.0 (c = 0.2, $CHCl_3$) | 99 |
| 24 | 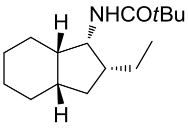 | 99:1 | GC-MS: column Chiraldex β-DM, 50 $^{o}$C to 175 ℃ at 1 ℃/min. $t_R$ = 91.5 min / 95.0 min. | $[\alpha]_D^{29}$ = -10.5 (c = 0.2, $CHCl_3$) | 99 |
| 25 | 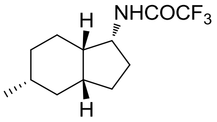 | 97:3 | GC-MS: column Chiraldex β-DM, 50 $^{o}$C to 175 ℃ at 1 ℃/min. $t_R$ = 53.5 min / 55.6 min. | $[\alpha]_D^{29}$ = 60.5 (c = 0.2, $CHCl_3$) | 99 |
| 26 | 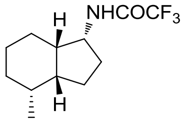 | 97:3 | GC-MS: column Chiraldex β-DM, 50 $^{o}$C to 175 ℃ at 1 ℃/min. $t_R$ = 57.5 min / 60.6 min / 63.5 min. | $[\alpha]_D^{29}$ = 72.5 (c = 0.2, $CHCl_3$) | 99 |
| 27 | 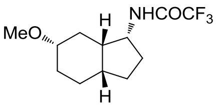 | 95:5 | GC-MS: column Chiraldex β-DM, 50 $^{o}$C to 175 ℃ at 1 ℃/min. $t_R$ = 71.7 min /74.5 min. | $[\alpha]_D^{29}$ = 20.5 (c = 0.2, $CHCl_3$) | 99 |
| 28 | 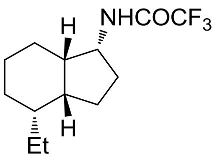 | 90:6:2:2 | GC-MS: column Chiraldex β-DM, 50 $^{o}$C to 175 ℃ at 1 ℃/min. $t_R$ = 65.3 min / 67.5 min / 69.9 min/ 71.0 min | $[\alpha]_D^{29}$ = 68.0 (c = 0.2, $CHCl_3$) | 99 |

| | | | | | |
|---|---|---|---|---|---|
| 29 | 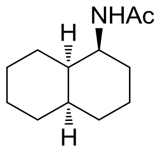 | 69:30:1 | GC-MS: column Chiraldex β-DM, 50 °C to 175 °C at 1 °C/min. $t_R$ = 87.6 min/ 89.5 min/89.8 min/91.7 min/ 92.5 min. | –– | 91 |
| 30 | 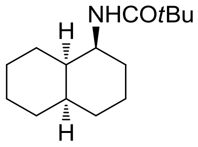 | 90:10 | GC-MS: column Chiraldex β-DM, 120 °C iso 200 min. $t_R$ = 137.5 min/ 176.0 min / 194.4 | $[\alpha]_D^{29}$ = -13.0 (c = 0.2, $CHCl_3$) | 94 |
| 31 | 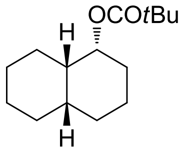 | 97:3 | GC-MS: column Chiraldex β-DM, 50 °C to 175 °C at 1 °C/min. $t_R$ = 68.2 min / 73.4 min. | $[\alpha]_D^{29}$ = 44.0 (c = 0.2, $CHCl_3$) | 99 |
| 32 | 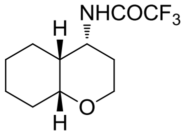 | 93:7 | GC-MS: column Chiraldex β-DM, 50 °C to 175 °C at 1 °C/min. $t_R$ = 62.5 min / 63.1 min /65.2 min. | $[\alpha]_D^{29}$ = 64.5 (c = 0.2, $CHCl_3$) | 96 |
| 33 | 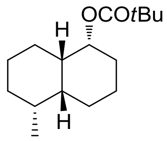 | 89:11 | GC-MS: column Chiraldex β-DM, 50 °C to 175 °C at 1 °C/min. $t_R$ = 76.3 min/ 78.9 min. | $[\alpha]_D^{29}$ = 42.0 (c = 0.2, $CHCl_3$) | 99 |
| 34 | 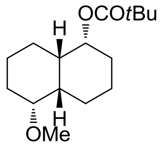 | 87:13 | GC-MS: column Chiraldex β-DM, 50 °C to 175 °C at 1 °C/min. $t_R$ = 86.9 min / 90.4 min. | $[\alpha]_D^{29}$ = 89.5 (c = 0.2, $CHCl_3$) | 99 |
| 35 | 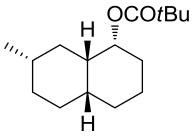 | 96:4 | GC-MS: column IVADEX-1, 50 °C to 175 °C at 1 °C/min. $t_R$ = 90.2 min / 90.7 min / 92.7 min. | $[\alpha]_D^{29}$ = 46.0 (c = 0.2, $CHCl_3$) | 98 |

## Characterization of hydrogenated products

### Methyl (*R*)-2-acetamido-3-cyclohexylpropanoate

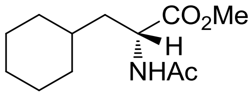


White solid. $^1$H NMR (400 MHz, Chloroform-*d*) δ 5.85 (d, *J* = 8.36 Hz, 1H), 4.65 (td, *J* = 8.69, 5.34 Hz, 1H), 3.73 (s, 3H), 2.02 (s, 3H), 1.78 (dtd, *J* = 13.06, 3.84, 2.26 Hz, 1H), 1.72 – 1.61 (m, 5H), 1.50 (ddd, *J* = 13.90, 8.95, 5.76 Hz, 1H), 1.31 (dddq, *J* = 14.02, 8.19, 5.55, 2.76, 2.27 Hz, 1H), 1.25 – 1.08 (m, 3H), 1.01 – 0.82 (m, 2H). $^{13}$C NMR (100 MHz, Chloroform-*d*) δ 173.9, 169.9, 52.4, 50.3, 40.4, 34.3, 33.6, 32.7, 26.5, 26.3, 26.1, 23.3. $[\alpha]_D^{29}$ = 16.0 (c = 0.2, $CHCl_3$). **HRMS-ESI**: Found $[M+Na]^+$ = 250.1424; $C_{12}H_{21}NO_4Na$ requires 250.1414.

### 2,2,2-trifluoro-*N*-((*S*)-1-(4-methylcyclohexyl)ethyl) acetamide

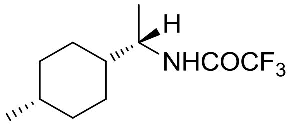


Colorless oil. $^1$H NMR (400 MHz, Chloroform-*d*) major diastereomer: δ 6.11 (s, *br*, 1H), 4.03 (dq, *J* = 13.47, 6.76 Hz, 1H), 1.77 – 1.59 (m, 2H), 1.55 – 1.24 (m, 8H), 1.19 (d, *J* = 6.65 Hz, 3H), 0.91 (d, *J* = 6.98 Hz, 3H). Minor diastereomer: δ 6.11 (s, *br*, 1H), 3.88 (dq, *J* = 13.47, 6.76 Hz, 1H), 1.77 – 1.59 (m, 2H), 1.55 – 1.24 (m, 8H), 1.17 (d, *J* = 6.64 Hz, 3H), 0.87 (d, *J* = 6.53 Hz, 3H). $^{13}$C NMR (100 MHz, Chloroform-*d*) major diastereomer: δ 156.7 (q, *J* = 36.36 Hz, 2C), 116.1 (q, *J* = 288.21 Hz 2C), 48.9, 41.7, 30.8, 30.8, 29.1, 24.8, 24.7, 19.3, 18.2. Minor diastereomer: δ 156.7 (q, *J* = 36.36 Hz, 2C), 116.1 (q, *J* = 288.21 Hz 2C), 50.8, 42.5, 34.8, 34.8, 32.6, 29.0, 28.9, 22.6, 17.8. $^{19}$F NMR (377 MHz, Chloroform-*d*) major diastereomer: δ -75.90. Minor diastereomer: δ -75.91. **HRMS-ESI**: Found $[M+Na]^+$ = 206.1234; $C_{11}H_{18}F_3ONNa$ requires 206.1233.

### 1-(((*R*)-1-((1*s*,4*S*)-4-methoxycyclohexyl)ethyl)-l2-azaneyl) ethan-1-one

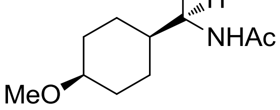


Colorless oil. $^1$H NMR (400 MHz, Chloroform-*d*) major diastereomer: δ 5.63 (s, *br*, 1H), 3.91 (dq, *J* = 13.95, 6.86 Hz, 1H), 3.45-3.40 (m, 1H), 3.28 (s, 3H), 2.15 – 1.71 (m, 5H), 1.52 – 1.21 (m, 6H), 1.20 – 0.99 (m, 1H), 1.09 (d, *J* = 6.80 Hz, 2H). Minor diastereomer: δ 5.52 (s, *br*, 1H), 3.91 (dq, *J* = 13.95, 6.86 Hz, 1H), 3.34 (s, 1H), 3.11-3.02 (m, 1H), 2.15 – 1.71 (m, 5H), 1.52 – 1.21 (m, 6H), 1.20 – 0.99 (m, 1H). $^{13}$C NMR (101 MHz, $CDCl_3$) major diastereomer: δ 169.8, 74.7, 55.6, 53.4, 49.4, 42.1, 31.4, 29.1, 29.0, 22.8, 17.7. Minor diastereomer: δ 169.7, 79.3, 55.7, 49.3, 49.2, 42.5,

27.2, 27.1, 23.3, 23.3, 18.3. **HRMS-ESI**: Found $[M+Na]^+$ = 221.1358; $C_{11}H_{20}NO_2Na$ requires 221.1386.

**1-(((*R*)-1-((1*s*,4*S*)-4-methylcyclohexyl)ethyl)-l2-azaneyl)ethan-1-one**

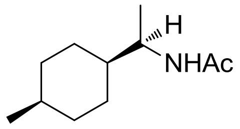


Colorless oil. $^1$H NMR (400 MHz, Chloroform-*d*) major diastereomer: δ 5.25 (s, *br*, 1H), 3.99 (dq, *J* = 13.56, 6.88 Hz, 1H), 1.97 (s, 3H), 1.83 – 1.61 (m, 2H), 1.54 – 1.15 (m, 8H), 1.09 (d, *J* = 6.72 Hz, 3H), 0.91 (d, *J* = 6.98 Hz, 3H). Minor diastereomer: δ 5.25 (s, *br*, 1H), 3.85 (dq, *J* = 13.42, 6.83 Hz, 1H), 1.97 (s, 3H), 1.83 – 1.61 (m, 2H), 1.54 – 1.15 (m, 8H), 1.08 (d, *J* = 6.76 Hz, 3H), 0.86 (d, *J* = 6.54 Hz, 3H). $^{13}$C NMR (100 MHz, Chloroform-*d*) major diastereomer: δ 169.6, 47.8, 41.9, 34.9, 30.9, 30.8, 29.1, 24.8, 24.7, 19.3, 18.5. Minor diastereomer: δ 169.6, 49.7, 42.7, 32.6, 29.7, 29.0, 28.8, 23.4, 23.3, 22.5, 18.0. **HRMS-ESI**: Found $[M+Na]^+$ = 205.1460; $C_{11}H_{20}NONa$ requires 205.1437.

**(*1S,4s*)-methyl 4-((*R*)-1-acetamidoethyl)cyclohexanecarboxylate**

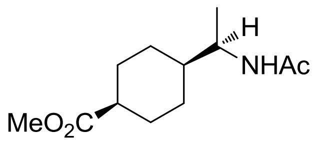


Colorless oil. $^1$H NMR (400 MHz, Chloroform-*d*) major diastereomer: δ 5.33 (d, *J* = 9.6 Hz, 1H), 3.94 – 3.86 (m, 1H), 3.65 (s, 3H), 2.62 – 2.53 (m, 1H), 2.15 – 2.03 (m, 2H), 1.94 (s, 3H), 1.62 – 1.43 (m, 4H), 1.39 – 1.21 (m, 3H), 1.05 (d, *J* = 6.8 Hz, 3H). Minor diastereomer: δ 5.37 (d, *J* = 9.6 Hz, 1H), 3.91 – 3.80 (m, 1H), 3.64 (s, 3H), 2.65 – 2.60 (m, 1H), 2.26 – 2.16 (m, 2H), 1.96 (s, 3H), 1.62 – 1.43 (m, 4H), 1.39 – 1.21 (m, 3H), 1.07 (d, *J* = 6.8 Hz, 3H). $^{13}$C NMR (100 MHz, Chloroform-*d*) major diastereomer: δ 175.6, 169.4, 51.6, 48.4, 42.1, 39.7, 26.8, 26.6, 25.7, 25.4, 23.7, 18.1. Minor diastereomer: δ 176.4, 169.4, 51.7, 49.2, 43.2, 42.5, 28.8, 28.2, 28.2, 26.7, 25.5, 18.2. **HRMS-ESI**: Found $[M+Na]^+$ = 250.1426.; $C_{15}H_{21}NO_2Na$ requires 250.1414.

***N*-((*R*)-1-((1*s*,4*S*)-4-methylcyclohexyl)ethyl)cyclohexane carboxamide**

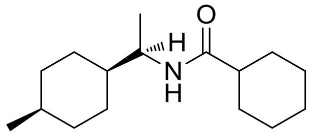


White solid. $^1$H NMR (400 MHz, Chloroform-*d*) major diastereomer: δ 5.37 (s, *br*, 1H), 3.98 (dq, *J* = 14.30, 6.89 Hz, 1H), 3.84 (dq, *J* = 13.55, 6.80 Hz, 1H), 2.07 (ddt, *J* = 11.72, 7.09, 3.39 Hz, 1H), 1.89 – 1.62 (m, 6H), 1.54 – 1.16 (m, 14H), 1.08 (d, *J* = 6.71 Hz, 3H), 0.90 (d, *J* = 6.97 Hz, 3H). Minor diastereomer: δ 5.37 (s, *br*, 1H), 3.84 (dq, *J* = 13.55, 6.80 Hz, 1H), 2.07 (ddt, *J* = 11.72, 7.09, 3.39 Hz, 1H), 1.89 – 1.62 (m,

6H), 1.54 – 1.16 (m, 14H), 1.05 (d, *J* = 6.71 Hz, 3H), 0.85 (d, *J* = 6.53 Hz, 3H). $^{13}$C NMR (100 MHz, Chloroform-*d*) major diastereomer: δ 175.6, 47.2, 46.9, 42.2, 31.1, 31.0, 30.1, 29.7, 29.2, 25.9, 25.9, 25.9, 25.0, 24.8, 19.4, 18.8. Minor diastereomer: δ 175.6, 49.1, 43.0, 35.1, 35.0, 32.8, 30.1, 29.8, 29.1, 29.0, 28.9, 25.9, 25.9, 25.9, 22.7, 18.3. **HRMS-ESI**: Found [M+Na]$^+$ = 274.2153; $C_{16}H_{29}NONa$ requires 274.2141.

**(1*s*,4*S*)-*N*-((*R*)-1-cyclohexylethyl)-4-methylcyclohexane-1-carboxamide**

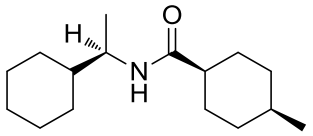


White solid. $^1$H NMR (400 MHz, Chloroform-*d*) major diastereomer: δ 5.31 (s, *br*, 1H), 3.86 (dq, *J* = 10.80, 7.78, 5.43 Hz, 1H), 2.18 (tt, *J* = 8.07, 4.18 Hz, 1H), 1.90 – 1.42 (m, 12H), 1.42 – 1.07 (m, 6H), 1.05 (d, *J* = 6.77 Hz, 3H), 0.93 (d, *J* = 6.91 Hz, 3H), 1.00 – 0.88 (m, 2H). Minor diastereomer: δ 5.28 (s, *br*, 1H), 3.86 (dq, *J* = 10.80, 5.43 Hz, 1H), 1.96 (tt, *J* = 12.13, 3.49 Hz, 1H), 1.90 – 1.42 (m, 12H), 1.42 – 1.07 (m, 6H), 1.04 (d, *J* = 6.76 Hz, 3H), 1.00 – 0.88 (m, 2H), 0.87 (d, *J* = 6.54 Hz, 3H). $^{13}$C NMR (100 MHz, Chloroform-*d*) major diastereomer: δ174.8, 49.0, 43.3, 43.2,31.4, 31.3, 29.3, 29.3, 29.1, 26.6, 26.3, 26.3, 26.1, 25.8, 19.9, 18.2. Minor diastereomer: δ 175.5, 49.0, 45.8, 43.3, 34.7, 34.6, 32.1, 30.1, 29.8, 29.3, 29.1, 26.6, 26.5, 26.3, 22.7, 18.2. **HRMS-ESI**: Found [M+Na]$^+$ = 274.2161; $C_{16}H_{29}NONa$ requires 274.2141.

**(*S*)-2-((*R*)-1-cyclohexylethyl)piperidine**

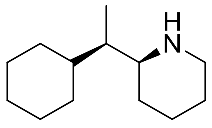


Colorless oil. $^1$H NMR (400 MHz, Chloroform-*d*) major diastereomer: δ 3.58 – 3.50 (m, 1H), 3.03 – 2.93 (m, 1H), 2.94 – 2.74 (m, 1H), 1.94 – 1.60 (m, 8H), 1.57 – 1.37 (m, 4H), 1.33 – 1.04 (m, 6H), 0.93 (d, *J* = 6.95 Hz, 3H). Minor diastereomer: δ 3.58 – 3.50 (m, 1H), 2.94 – 2.74 (m, 2H), 1.94 – 1.60 (m, 8H), 1.57 – 1.37 (m, 4H), 1.33 – 1.04 (m, 6H), 1.00 (d, *J* = 6.89 Hz, 3H). $^{13}$C NMR (100 MHz, Chloroform-*d*) major diastereomer: δ 59.7, 46.6, 40.9, 39.3, 32.1, 31.2, 29.4, 27.4, 26.7, 26.5, 24.8, 23.2, 11.3. Minor diastereomer: δ 60.5, 46.5, 41.5, 35.8, 32.0, 29.8, 28.0, 26.8, 26.4, 25.9, 24.8, 11.3. **HRMS-ESI**: Found [M+Na]$^+$ = 218.1854; $C_{13}H_{25}NNa$ requires 218.1879.

**2,2,2-trifluoro-*N*-((*S*)-1-((1*S*,3*R*)-3-methylcyclohexyl)ethyl) acetamide**

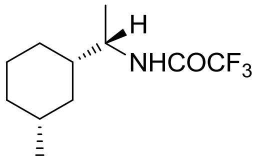


Colorless oil. $^1$H NMR (400 MHz, Chloroform-*d*) major diastereomer: δ 6.09 (s, *br*, 1H), 3.88 (dq, *J* = 15.11, 7.40 Hz, 1H), 1.86 – 1.21 (m, 8H), 1.17 (d, *J* = 6.79 Hz, 3H), 0.90 (d, *J* = 6.58 Hz, 3H), 0.88 – 0.53 (m, 2H). Minor diastereomer: δ 6.09 (s, *br*, 1H), 4.03 (dq, *J* = 15.11, 7.40 Hz, 1H), 1.86 – 1.21 (m, 8H), 1.17 (d, *J* = 6.79 Hz, 3H), 0.90 (d, *J* = 6.58 Hz, 3H), 0.88 – 0.53 (m, 2H). $^{13}$C NMR (100 MHz, Chloroform-*d*) major diastereomers: δ 156.6 (q, *J* = 36.48), 118.3 (q, *J* = 262.60 Hz), 50.8, 42.7, 37.5, 35.0, 32.6, 28.5, 25.9, 22.9, 17.6. Minor diastereomer: δ 156.6 (q, *J* = 36.48), 118.3 (q, *J* = 262.60 Hz), 50.8, 42.8, 37.7, 35.0, 32.6, 28.5, 25.9, 22.9, 17.6. $^{19}$F NMR (377 MHz, Chloroform-*d*) major diastereomer: δ -75.90, -75.91. **HRMS-ESI**: Found $[M+Na]^+$ = 206.1129; $C_{11}H_{18}F_3ONNa$ requires 206.1233.

***N*-((*R*)-1-((1*R*,2*S*)-2-methylcyclohexyl)ethyl)pivalamide**

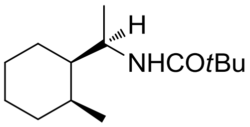


White solid. $^1$H NMR (400 MHz, Chloroform-*d*) major diastereomer: δ 5.29 (s, *br*, 1H), 3.78 (dq, *J* = 13.95, 6.86 Hz, 1H), 2.04 – 1.91 (m, 1H), 1.75 – 1.67 (m, 1H), 1.60 – 1.52 (m, 1H), 1.51 – 1.30 (m, 4H), 1.27 – 1.12 (m, 12H), 1.07 (d, *J* = 6.52 Hz, 3H), 0.86 (d, *J* = 7.15 Hz, 3H). Minor diastereomer: δ 5.36 (s, br, 1H), 4.31 (dq, *J* = 13.95, 6.86 Hz, 1H), 2.04 – 1.91 (m, 1H), 1.75 – 1.67 (m, 1H), 1.60 – 1.52 (m, 1H), 1.51 – 1.30 (m, 4H), 1.27 – 1.12 (m, 12H), 1.07 (d, *J* = 6.52 Hz, 3H), 0.82 (d, *J* = 7.12 Hz, 3H). $^{13}$C NMR (100 MHz, Chloroform-*d*) major diastereomer: δ 177.6, 47.6, 47.1, 38.8, 33.9, 29.4, 27.8, 26.8, 23.4, 20.3, 19.2, 12.0. Minor diastereomer: δ 177.4, 47.0, 46.4, 38.8, 33.7, 29.2, 27.8, 26.7, 24.0, 20.2, 20.1, 12.6. **HRMS-ESI**: Found $[M+Na]^+$ = 248.1990; $C_{14}H_{27}NONa$ requires 248.1985.

***N*-((*S*)-1-((1*s*,4*R*)-4-methylcyclohexyl)propyl) acetamide**

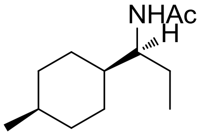


Colorless oil. $^1$H NMR (400 MHz, Chloroform-*d*) major diastereomer: δ 5.36 (s, *br*, 1H), 3.86 (qd, *J* = 9.73, 5.03 Hz, 1H), 2.01 (s, 3H), 1.77 – 1.57 (m, 3H), 1.52 – 1.20 (m, 9H), 0.92 – 0.82 (m, 6H). Minor diastereomer: δ 5.36 (s, *br*, 1H), 3.70 (qd, *J* = 9.73, 5.03 Hz, 1H), 2.00 (s, 3H), 1.77 – 1.57 (m, 3H), 1.52 – 1.20 (m, 9H), 0.92 – 0.82 (m, 6H). $^{13}$C NMR (100 MHz, Chloroform-*d*) major diastereomer: δ 170.2, 53.3, 40.4, 31.2, 31.1, 29.1, 25.2,

25.0, 24.5, 23.5, 19.3, 10.2. Minor diastereomer: δ 170.2, 55.4, 41.4, 35.1, 35.1, 32.8, 29.7, 28.5, 25.1, 23.5, 22.7, 10.6. **HRMS-ESI**: Found $[M+Na]^+$ = 220.1660; $C_{12}H_{23}NONa$ requires 220.1672.

**Methyl (*S*)-3-acetamido-3-((1*s*,4*R*)-4-methylcyclohexyl) propanoate**

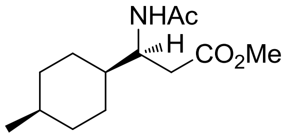


Colorless oil. $^1$H NMR (400 MHz, Chloroform-*d*) major diastereomer: δ 6.13 (s, *br,* 1H), 4.21 (ddt, *J* = 9.76, 8.24, 5.30 Hz, 1H), 3.66 (s, 3H), 2.55 (d, *J* = 5.08 Hz, 2H), 1.97 (s, 3H), 1.82 – 1.51 (m, 3H), 1.51 – 1.19 (m, 7H), 0.88 (d, *J* = 6.93 Hz, 3H). Minor diastereomer: δ 6.11 (s, *br,* 1H), 4.02 (ddt, *J* = 9.76, 8.24, 5.30 Hz, 1H), 3.66 (s, 3H), 2.52 (d, *J* = 4.92 Hz, 2H), 1.96(s, 3H), 1.82 – 1.51 (m, 3H), 1.51 – 1.19 (m, 7H), 0.84 (d, *J* = 6.53 Hz, 3H). $^{13}$C NMR (100 MHz, Chloroform-*d*) major diastereomer: δ 172.9, 169.8, 51.8, 48.3, 38.9, 36.1, 30.7, 30.7, 29.4, 25.6, 25.2, 23.5, 19.6. Minor diastereomer: δ 172.8, 169.8, 51.8, 50.8, 40.7, 36.2, 34.9, 34.8, 32.5, 29.8, 29.5, 29.4, 22.6. **HRMS-ESI**: Found $[M+Na]^+$ = 264.1576; $C_{13}H_{23}NO_3Na$ requires 264.1570.

**Methyl (*S*)-3-acetamido-3-((1*s*,4*R*)-4-methoxycyclohexyl) propanoate**

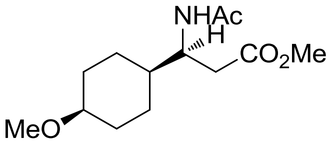


Colorless oil. $^1$H NMR (400 MHz, Chloroform-*d*) major diastereomer: δ 6.09 (d, *J* = 9.27 Hz, 1H), 4.09 (ddt, *J* = 10.90, 9.06, 5.00 Hz, 1H), 3.66 (s, 3H), 3.39 (tt, *J* = 10.48, 3.14 Hz, 1H), 3.25 (s, 3H), 2.60 – 2.46 (m, 2H), 2.13 – 2.02 (m, 1H), 1.96 (s, 3H), 1.94 – 1.69 (m, 2H), 1.60 – 1.25 (m, 5H), 1.20 – 0.91 (m, 1H). Minor diastereomer: δ 6.04 (d, *J* = 9.14 Hz, 1H), 4.02 (ddt, *J* = 11.26, 9.67, 5.00 Hz, 1H), 3.67 (s, 3H), 3.31 (s, 3H), 3.04 (tt, *J* = 10.48, 3.14 Hz, 1H), 2.60 – 2.46 (m, 2H), 2.13 – 2.02 (m, 1H), 1.96 (s, 3H), 1.94 – 1.69 (m, 2H), 1.60 – 1.25 (m, 5H), 1.20 – 0.91 (m, 1H). $^{13}$C NMR (100 MHz, Chloroform-*d*) major diastereomer: δ 172.8, 169.7, 74.5, 55.6, 51.8, 50.3, 40.1, 36.0, 29.2, 28.7, 23.7, 23.5, 23.2. Minor diastereomer: δ 172.7, 169.7, 79.2, 55.8, 51.9, 50.2, 40.3, 36.3, 31.4, 31.3 , 27.9, 27.7, 23.5. **HRMS-ESI**: Found $[M+Na]^+$ = 280.1530; $C_{13}H_{23}NO_4Na$ requires 280.1519.

**Methyl (*R*)-2-acetamido-3-((1*s*,4*S*)-4-(tert-butyl)cyclohexyl) propanoate**

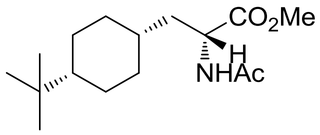


Colorless oil. $^{1}$H NMR (400 MHz, Chloroform-*d*) major diastereomer: δ 5.88 (s, *br*, 1H), 4.59 (td, *J* = 8.62, 5.41 Hz, 1H), 3.74 (s, 3H), 2.01 (s, 3H), 1.92 (ddd, *J* = 13.82, 8.32, 5.44 Hz, 1H), 1.80 – 1.40 (m, 8H), 1.15 – 0.89 (m, 3H), 0.83 (s, 9H). Minor diastereomer: δ 5.88 (s, *br*, 1H), 4.65 (td, *J* = 8.23, 4.96 Hz, 1H), 3.72 (s, 3H), 2.02 (s, 3H), 1.92 (ddd, *J* = 13.82, 8.32, 5.44 Hz, 1H), 1.80 – 1.40 (m, 8H), 1.15 – 0.89 (m, 3H), 0.82 (s, 9H). $^{13}$C NMR (100 MHz, Chloroform-*d*) major diastereomer: δ 173.9, 170.0, 52.4, 51.0, 48.5, 34.4, 32.7, 31.3, 30.0, 28.9, 27.6, 23.3, 21.9, 21.7. Minor diastereomer: δ 173.9, 170.0, 52.4, 50.4, 48.1, 40.4, 34.4, 34.0, 33.2, 32.5, 27.7, 27.6, 27.3, 27.2. **HRMS-ESI**: Found $[M+Na]^+$ = 306.2048; $C_{16}H_{29}NO_3Na$ requires 306.2040.

**Methyl (*R*)-2-acetamido-3-((1*s*,4*S*)-4-methylcyclohexyl) propanoate**

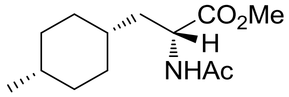


Colorless oil. $^{1}$H NMR (400 MHz, Chloroform-*d*) major diastereomer: δ 5.96 (s, *br*, 1H), 4.62 (td, *J* = 8.44, 5.22 Hz, 1H), 3.72 (s, 3H), 2.01 (s, 3H), 1.87 – 1.75 (m, 1H), 1.70 – 1.41 (m, 7H), 1.41 – 1.28 (m, 2H), 1.28 – 1.14 (m, 2H), 0.88 (d, *J* = 6.86 Hz, 3H). Minor diastereomer: δ 5.95 (s, *br*, 1H), 4.64 (td, *J* = 8.44, 5.22 Hz, 1H), 3.71 (s, 3H), 2.01 (s, 3H), 1.87 – 1.75 (m, 1H), 1.70 – 1.41 (m, 7H), 1.41 – 1.28 (m, 2H), 1.28 – 1.14 (m, 2H), 0.85 (d, *J* = 6.57 Hz, 3H).$^{13}$C NMR (100 MHz, Chloroform-*d*) major diastereomer: δ 174.0, 170.0, 52.4, 50.6, 37.2, 31.5, 30.7, 30.5, 30.2, 29.3, 28.1, 23.3, 20.4. Minor diastereomer: δ 174.0, 169.9, 52.4, 50.3, 40.3, 35.2, 35.0, 34.1, 33.5, 32.7, 32.7, 32.6, 22.7. **HRMS-ESI**: Found $[M+Na]^+$ = 264.1567; $C_{13}H_{23}NO_3Na$ requires 264.1570.

**Methyl (*R*)-2-acetamido -3- ((1*s*,4*S*)-4-(trifluoromethyl) cyclohexyl) propanoate**

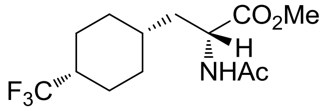


Colorless oil. $^{1}$H NMR (400 MHz, Chloroform-*d*) major diastereomer: δ 5.97 (s, *br*, 1H), 4.63 (td, *J* = 8.73, 5.22 Hz, 1H), 3.74 (s, 3H), 2.02 (s, 3H), 2.00 – 1.81 (m, 2H), 1.78 – 1.47 (m, 8H), 1.37 – 1.20 (m, 1H), 1.06 – 0.88 (m, 1H). Minor diastereomer: 5.95 (s, *br*, 1H), 4.68 (dt, *J* = 5.50, 3.20 Hz, 1H), 3.74 (s, 3H), 2.02 (s, 3H), 2.00 – 1.81 (m, 2H), 1.78 – 1.47 (m, 8H), 1.37 – 1.20 (m, 1H), 1.06 – 0.88 (m, 1H). $^{13}$C NMR (100 MHz, Chloroform-*d*) major diastereomer: δ 173.6, 170.1, 128.06 (q, *J* = 279.07 Hz), 52.6, 50.6, 40.57 (q, *J* =

27.26 Hz), 35.8, 29.6, 29.0, 27.7, 23.3, 20.9, 20.6. Minor diastereomer: δ 173.6, 170.0, 128.06 (q, *J* = 279.07 Hz), 52.6, 50.1, 42.0, 41.8, 40.57 (q, *J* = 27.26 Hz), 33.6, 31.8, 31.0, 24.9, 24.8. $^{19}$F NMR (377 MHz, Chloroform-*d*) major diastereomer: δ -73.83. Minor diastereomer: -72.03. **HRMS-ESI**: Found $[M+Na]^+$ = 318.1281; $C_{13}H_{20}F_3NO_3Na$ requires 318.1287.

**Methyl (2*R*,3*S*)-2-acetamido-3-((1*s*,4*R*)-4-methylcyclohexyl) butanoate**

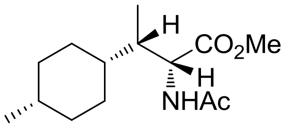


Colorless oil. $^1$H NMR (400 MHz, Chloroform-*d*) major diastereomer: δ 5.80 (d, *J* = 9.55 Hz, 1H), 4.92 (dd, *J* = 9.53, 2.75 Hz, 1H), 3.72 (s, 3H), 2.04 (s, 3H), 1.96 – 1.68 (m, 5H), 1.62 – 1.31 (m, 4H), 1.19 – 1.07 (m, 2H), 0.83 (d, *J* = 7.15 Hz, 3H), 0.78 (d, *J* = 6.86 Hz, 3H). Minor diastereomer: δ 5.96 (d, *J* = 9.10 Hz, 1H), 4.57 (dd, *J* = 9.37 Hz, 1H), 3.63 (s, 3H), 2.98 (dq, *J* = 9.47, 7.02 Hz, 1H), 2.01 (s, 3H), 1.96 – 1.68 (m, 4H), 1.62 – 1.31 (m, 4H), 1.19 – 0,87 (m, 8H). $^{13}$C NMR (100 MHz, Chloroform-*d*) major diastereomer: δ 173.9, 170.4, 53.3, 52.4, 41.9, 37.7, 34.0, 28.7, 26.9, 24.1, 23.4, 20.2, 11.9, 11.8. Minor diastereomer: δ 173.3, 169.9, 55.8, 52.0, 39.3, 32.7, 28.7, 24.3, 23.4, 23.3, 23.1, 20.2, 19.3, 15.2. **HRMS-ESI**: Found $[M+Na]^+$ = 278.1727; $C_{14}H_{25}NO_3Na$ requires 278.1727.

***N*-((1*S*,3a*R*,7a*R*)-octahydro-1*H*-inden-1-yl)acetamide**

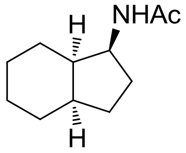


White solid. $^1$H NMR (400 MHz, Chloroform-*d*) major diastereomer: δ 5.54 (s, *br*, 1H), 4.28 – 4.11 (m, 1H), 2.30 – 1.99 (m, 2H), 1.96 (s, 3H), 1.79 – 1.60 (m, 2H), 1.57 – 1.25 (m, 8H), 1.20 – 0.87 (m, 2H). Minor diastereomer: δ 5.58 (s, *br,* 1H), 4.28 – 4.11 (m, 1H), 2.30 – 1.99 (m, 2H), 1.96 (s, 3H), 1.79 – 1.60 (m, 2H), 1.57 – 1.25 (m, 8H), 1.20 – 0.87 (m, 2H). $^{13}$C NMR (100 MHz, Chloroform-*d*) major diastereomer: δ 170.0, 54.0, 41.1, 37.0, 28.9, 26.8, 25.0, 24.0, 23.4, 21.9, 20.9. Minor diastereomer: δ 170.1, 52.5, 46.2, 37.8, 30.9, 28.5, 28.4, 25.1, 24.1, 23.5, 22.4. **HRMS-ESI**: Found $[M+Na]^+$ = 204.1356; $C_{11}H_{19}NONa$ requires 204.1359.

**_N_-((1_S_,3a_R_,7a_R_)-octahydro-1_H_-inden-1-yl)isobutyramide**

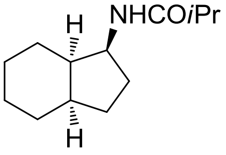


White solid. $^{1}$H NMR (400 MHz, Chloroform-*d*) major diastereomer: δ 5.35 (s, *br*, 1H), 4.21 (dt, *J* = 13.93, 7.48Hz, 1H), 2.40 – 2.21 (m, 1H), 2.20 – 1.87 (m, 3H), 1.81 – 1.22 (m, 10H), 1.14 (d, *J* = 6.86 Hz, 6H), 0.93 (qd, *J* = 12.63, 3.37 Hz, 1H). Minor diastereomer: δ 5.35 (s, *br*, 1H), 4.21 (dt, *J* = 13.93, 7.48Hz, 1H), 2.40 – 2.21 (m, 1H), 2.20 – 1.87 (m, 3H), 1.81 – 1.22 (m, 10H), 1.14 (d, *J* = 6.86 Hz, 6H), 0.93 (qd, *J* = 12.63, 3.37 Hz, 1H). $^{13}$C NMR (100 MHz, Chloroform-*d*) major diastereomer: δ 176.6, 53.5, 40.9, 36.9, 29.0, 26.8, 24.9, 24.0, 21.7, 20.8, 19.9, 19.6. Minor diastereomer: δ 176.8, 52.0, 46.3, 37.8, 35.7, 30.9, 28.4, 28.3, 25.1, 24.0, 22.4, 19.8. **HRMS-ESI**: Found $[M+Na]^+$ = 232.1672; $C_{13}H_{23}NONa$ requires 232.1644.

**_N_-((1_S_,3a_R_,7a_R_)-octahydro-1_H_-inden-1-yl)pivalamide**

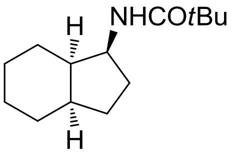


White solid. $^{1}$H NMR (400 MHz, Chloroform-*d*) major diastereomer: δ 5.50 (s, *br*, 1H), 4.25 – 4.09 (m, 1H), 2.37 – 1.90 (m, 3H), 1.81 – 1.22 (m, 10H), 1.17 (s, 9H), 0.93 (qd, *J* = 12.57, 3.47 Hz, 1H). Minor diastereomer: δ 5.44 (s, *br*, 1H), 4.25 – 4.09 (m, 1H), 2.37 – 1.90 (m, 3H), 1.81 – 1.22 (m, 10H), 1.17 (s, 9H), 0.93 (qd, *J* = 12.57, 3.47 Hz, 1H). $^{13}$C NMR (100 MHz, Chloroform-*d*) major diastereomer: δ178.2, 53.7, 41.0, 38.7, 37.1, 29.2, 27.8, 26.9, 25.0, 24.1, 21.8, 21.0. Minor diastereomer: δ 178.3, 52.2, 46.5, 38.6, 37.9, 31.0, 28.9, 28.5, 28.4, 27.8, 25.3, 22.6. **HRMS-ESI**: Found $[M+Na]^+$ = 246.1826; $C_{14}H_{125}NONa$ requires 246.1828.

**2,2,2-trifluoro-_N_-((1_S_,3a_R_,7a_R_)-octahydro-1_H_-inden-1-yl)acetamide**

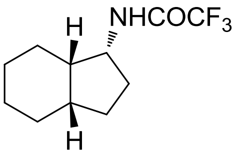


White solid. $^{1}$H NMR (400 MHz, Chloroform-*d*) δ 6.14 (s, *br*, 1H), 4.26 (qd, *J* = 8.99, 6.05 Hz, 1H), 2.38 – 2.00 (m, 3H), 1.86 – 1.26 (m, 9H), 1.17 (qt, *J* = 12.73, 3.25 Hz, 1H), 0.98 (qd, *J* = 12.51, 3.49 Hz, 1H). $^{13}$C NMR (100 MHz, Chloroform-*d*) δ 157.1 (q, *J* = 36.44 Hz), 116.0 (q, *J* = 289.79 Hz), 54.4, 40.9, 37.0, 28.8, 26.7, 24.8, 24.0, 21.7, 20.8. $^{19}$F NMR (377 MHz, Chloroform-*d*) δ -75.93. $[\alpha]_D^{29}$ = 76.5 (c = 0.2, $CHCl_3$). **HRMS-ESI**: Found $[M+Na]^+$ = 258.1086; $C_{11}H_{16}NOF_3Na$ requires 258.1076.

**(1*S*,3a*R*,7a*R*)-octahydro-1*H*-inden-1-yl pivalate**

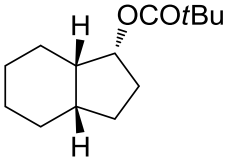


Colorless oil. $^1$H NMR (400 MHz, Chloroform-*d*) δ 5.00 (dt, *J* = 8.67, 6.19 Hz, 1H), 2.15 – 1.97 (m, 3H), 1.74 – 1.58 (m, 3H), 1.59 – 1.35 (m, 7H), 1.22 – 1.12 (m, 10H). $^{13}$C NMR (100 MHz, Chloroform-*d*) δ 178.6, 78.2, 41.3, 38.9, 36.4, 28.9, 27.4, 27.3, 24.9, 24.7, 21.9, 21.6. $[\alpha]_D^{29}$ = 17.5 (c = 0.2, $CHCl_3$). **HRMS-ESI**: Found $[M+Na]^+$ = 247.1676; $C_{14}H_{24}NONa$ requires 247.1669.

***N*-((1*R*,2*R*,3a*S*,7a*S*)-2-methyloctahydro-1*H*-inden-1-yl) pivalamide**

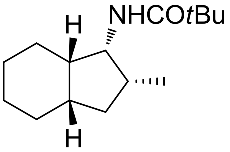


White solid. $^1$H NMR (400 MHz, Chloroform-*d*) δ 5.73 (s, *br*, 1H), 4.33 (td, *J* = 10.15, 6.20 Hz, 1H), 2.34 (dddd, *J* = 17.40, 8.72, 7.27, 2.07 Hz, 1H), 2.09 – 1.96 (m, 2H), 1.79 (dt, *J* = 12.60, 7.79 Hz, 1H), 1.73 – 1.64 (m, 1H), 1.63 – 1.29 (m, 6H), 1.20 (s, 9H), 1.19 – 0.98 (m, 2H), 0.91 (d, *J* = 7.28 Hz, 3H). $^{13}$C NMR (100 MHz, Chloroform-*d*) δ 177.9, 54.1, 41.9, 39.1, 36.2, 35.8, 33.5, 27.9, 27.0, 25.4, 23.3, 21.1, 18.0. $[\alpha]_D^{29}$ = -4.0 (c = 0.2, $CHCl_3$). **HRMS-ESI**: Found $[M+Na]^+$ = 260.1986; $C_{15}H_{27}NONa$ requires 260.1985.

***N*-((1*R*,2*R*,3a*S*,7a*S*)-2-ethyloctahydro-1*H*-inden-1-yl)pivalamide**

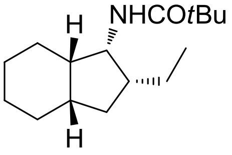


White solid. $^1$H NMR (400 MHz, Chloroform-*d*) δ 5.69 (s, *br*, 1H), 4.40 (td, *J* = 9.69, 5.98 Hz, 1H), 2.16 – 1.92 (m, 3H), 1.83 – 1.72 (m, 1H), 1.62 (ddt, *J* = 24.31, 11.34, 4.27 Hz, 2H), 1.55 – 1.34 (m, 5H), 1.35 – 1.22 (m, 1H), 1.21 (s, 9H), 1.14 – 0.95 (m, 3H), 0.87 (t, *J* = 7.26 Hz, 3H). $^{13}$C NMR (100 MHz, Chloroform-*d*) δ 177.8, 53.9, 41.8, 41.3, 39.1, 36.0, 33.9, 27.9, 27.4, 25.9, 25.2, 23.2, 21.2, 13.0. $[\alpha]_D^{29}$ = -10.5 (c = 0.2, $CHCl_3$). **HRMS-ESI**: Found $[M+Na]^+$ = 274.2142; $C_{16}H_{29}NONa$ requires 274.2141.

**2,2,2-trifluoro-*N*-((1*R*,3a*R*,5*R*,7a*S*)-5-methyloctahydro-1*H*-inden-1-yl)acetamide**

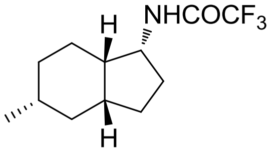


Colorless oil. $^1$H NMR (400 MHz, Chloroform-*d*) δ 6.10 (s, *br*, 1H), 4.43 (td, *J* = 8.73, 6.35 Hz, 1H), 2.23 – 2.13 (m, 2H), 2.06 (dddd, *J* = 14.42, 12.53, 6.44, 2.45 Hz, 1H), 1.84 – 1.34 (m, 8H), 0.91 (d, *J* = 6.63 Hz, 3H), 0.85 – 0.72 (m, 1H), 0.65 (dt, *J* = 13.51, 11.52 Hz, 1H). $^{13}$C NMR (100 MHz, Chloroform-d) δ 156.9 (q, *J* = 36.4 Hz), 116.1 (q, *J* = 288.1 Hz), 53.8, 39.9, 38.5, 38.4, 32.3, 31.8, 30.4, 30.1, 22.9, 22.4. $^{19}$F NMR (377

MHz, Chloroform-*d*) δ -75.97. $[\alpha]_D^{29}$ = 60.5 (c = 0.2, $CHCl_3$). **HRMS-ESI**: Found $[M+Na]^+$ = 272.1232; $C_{12}H_{18}NOF_3Na$ requires 272.1233.

**2,2,2-trifluoro-*N*-((1*R*,3a*R*,4*R*,7a*S*)-4-methyloctahydro-1*H*-inden-1-yl) acetamide**

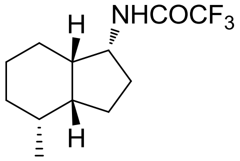


White solid. $^1$H NMR (400 MHz, Chloroform-*d*) δ 6.19 (s, *br*, 1H), 4.25 (qd, *J* = 8.90, 5.91 Hz, 1H), 2.25 – 1.99 (m, 3H), 1.74 (dqd, *J* = 13.75, 6.83, 3.78 Hz, 2H), 1.66 – 1.31 (m, 5H), 1.21 (qt, *J* = 12.84, 3.18 Hz, 1H), 1.07 (qd, *J* = 12.83, 3.22 Hz, 1H), 0.95 – 0.87 (m, 1H), 0.85 (d, *J* = 6.83 Hz, 3H). $^{13}$C NMR (100 MHz, Chloroform-*d*) δ 156.8 (q, *J* = 35.43 Hz), 115.9 (d, *J* = 289.79 Hz), 53.8, 43.1, 41.8, 31.9, 28.6, 27.8, 24.9, 21.1, 19.9, 19.3. $^{19}$F NMR (377 MHz, Chloroform-*d*) δ -75.87. $[\alpha]_D^{29}$ = 72.5 (c = 0.2, $CHCl_3$). **HRMS-ESI**: Found $[M+Na]^+$ = 272.1227; $C_{12}H_{18}NOF_3Na$ requires 272.1233.

**2,2,2-trifluoro-*N*-((1*R*,3a*R*,6*S*7a*S*)6-methoxyoctahydro-1*H*-inden-1-yl) acetamide**

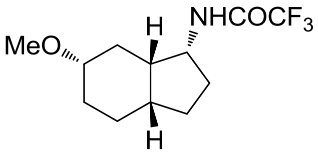


White solid. $^1$H NMR (400 MHz, Chloroform-*d*) δ 8.11 (s, *br*, 1H), 4.20 (td, *J* = 7.61 Hz, 1H), 3.41 – 3.25 (m, 4H), 2.34 – 2.23 (m, 1H), 2.26 – 2.12 (m, 1H), 2.12 – 1.99 (m, 1H), 1.82 – 1.34 (m, 9H). $^{13}$C NMR (100 MHz, Chloroform-d) δ 157.2 (q, *J* = 36.5 Hz), 116.2 (q, *J* = 288.0 Hz), 75.7, 55.8, 53.0, 39.2, 37.5, 30.8, 28.4, 27.8, 26.2, 23.6. $^{19}$F NMR (377 MHz, Chloroform-*d*) δ -75.91. $[\alpha]_D^{29}$ = 20.5 (c = 0.2, $CHCl_3$). **HRMS-ESI**: Found $[M+Na]^+$ = 288.1183; $C_{12}H_{18}NO_2F_3Na$ requires 288.1182.

***N*-((1*R*,3a*R*,4*R*,7a*S*)4-ethyloctahydro-1*H*-inden-1-yl)-2,2,2-trifluoroacetamide**

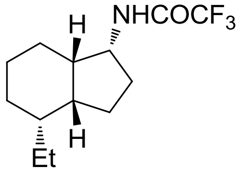


Colorless oil. $^1$H NMR (400 MHz, Chloroform-*d*) δ 6.16 (s, *br*, 1H), 4.25 (dt, *J* = 8.96, 4.35 Hz, 1H), 2.23 – 2.04 (m, 3H), 1.79 (tp, *J* = 13.20, 4.06, 3.38 Hz, 1H), 1.64 – 1.32 (m, 6H), 1.30 – 1.12 (m, 3H), 1.08 – 0.92 (m, 2H), 0.88 (t, *J* = 7.33 Hz, 3H). $^{13}$C NMR (100 MHz, Chloroform-*d*) δ 156.9 (q, *J* = 36.6 Hz), 116.0 (q, *J* = 288.3 Hz), 53.9, 41.9, 41.1, 39.1, 28.0, 27.3, 26.9, 25.0, 21.7, 19.4, 11.8. $^{19}$F NMR (377 MHz, Chloroform-*d*) δ -75.86. $[\alpha]_D^{29}$ = 68.0 (c = 0.2, $CHCl_3$). **HRMS-ESI**: Found $[M+Na]^+$ = 286.1394; $C_{13}H_{20}NOF_3Na$ requires 286.1389.

**$N$-((1$S$,4a$R$,8a$R$)-decahydronaphthalen-1-yl)acetamide**

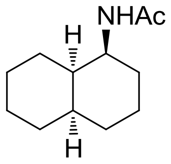


White solid. $^1$H NMR (400 MHz, Chloroform-$d$) major diastereomer: δ 6.01 (s, *br*, 1H), 3.87 (td, $J$ = 8.07, 4.30 Hz, 1H), 2.01 (s, 3H), 1.98 – 1.87 (m, 1H), 1.84 – 1.64 (m, 3H), 1.63 – 1.12 (m, 12H). Minor diastereomer: δ 6.19 (s, *br*, 1H), 4.06 (td, $J$ = 8.07, 4.30 Hz, 1H), 2.05 (s, 3H), 1.98 – 1.87 (m, 1H), 1.84 – 1.64 (m, 3H), 1.63 – 1.12 (m, 12H). $^{13}$C NMR (100 MHz, Chloroform-d) major diastereomer: δ 170.1, 52.4, 39.7, 36.0, 31.9, 26.8, 26.4, 25.2, 24.5, 23.2, 21.3, 19.9. Minor diastereomer: δ 170.7, 41.5, 39.7, 36.0, 31.9, 26.8, 26.4, 25.2, 22.9, 21.3, 20.9, 19.9. **HRMS-ESI**: Found $[M+Na]^+$ = 218.1515; $C_{12}H_{21}NONa$ requires 218.1514.

**$N$-((1$S$,4a$R$,8a$R$)-decahydronaphthalen-1-yl)pivalamide**

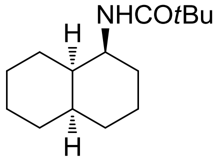


White solid. $^1$H NMR (400 MHz, Chloroform-$d$) δ 5.50 (s, *br*, 1H), 3.87 (ddt, $J$ = 12.53, 8.53, 4.40 Hz, 1H), 2.00 – 1.89 (m, 1H), 1.82 – 1.69 (m, 3H), 1.66 – 1.20 (m, 12H), 1.18 (s, 9H). $^{13}$C NMR (100 MHz, Chloroform-$d$) δ 177.5, 51.4, 36.0, 31.9, 27.8, 27.8, 27.1, 26.5, 25.3, 24.6, 21.4, 20.0. $[\alpha]_D^{29}$ = -13.0 (c = 0.2, $CHCl_3$). **HRMS-ESI**: Found $[M+Na]^+$ = 260.1980; $C_{15}H_{27}NONa$ requires 260.1985.

**(1$R$,4a$S$,8a$S$)-decahydronaphthalen-1-yl pivalate**

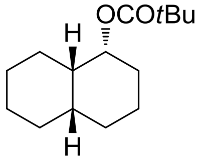


Colorless oil. $^1$H NMR (400 MHz, Chloroform-$d$) δ 4.74 (dt, $J$ = 11.70, 4.85 Hz, 1H), 1.96 (dq, $J$ = 9.31, 4.63 Hz, 1H), 1.75 (tdd, $J$ = 11.36, 5.52, 3.16 Hz, 3H), 1.64 – 1.30 (m, 12H), 1.17 (s, 9H). $^{13}$C NMR (100 MHz, Chloroform-d) δ 178.1, 75.6, 40.1, 39.0, 35.6, 31.8, 27.4, 26.3, 25.9, 24.6, 24.2, 21.5, 20.1. $[\alpha]_D^{29}$ = 44.0 (c = 0.2, $CHCl_3$). **HRMS-ESI**: Found $[M+Na]^+$ = 261.1835; $C_{15}H_{26}NO_2Na$ requires 261.1825.

**2,2,2-trifluoro-$N$-((4$R$,4a$R$,8a$R$)-octahydro-2$H$-chromen-4-yl)acetamide**

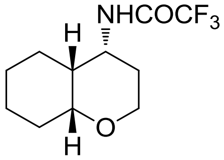


White solid. $^1$H NMR (400 MHz, Chloroform-$d$) δ 6.21 (s, *br*, 1H), 4.15 (ddt, $J$ = 12.85, 8.84, 4.81 Hz, 1H), 4.06 (dd, $J$ = 11.81, 5.10 Hz, 1H), 3.68 – 3.43 (m, 2H), 1.95 – 1.87 (m, 2H), 1.85 – 1.65 (m, 2H), 1.64 – 1.39 (m, 5H), 1.36 – 1.16 (m, 2H). $^{13}$C NMR (100 MHz, Chloroform-$d$) δ 156.5 (q, $J$ = 36.67 Hz), 115.9 (q, $J$ = 288.21 Hz), 75.0, 66.8, 50.5, 38.7, 31.6, 26.9,

25.2, 20.6, 19.8. $^{19}$F NMR (377 MHz, Chloroform-*d*) δ -75.93. $[\alpha]_D^{29}$ = 64.5 (c = 0.2, $CHCl_3$). **HRMS-ESI**: Found $[M+Na]^+$ = 274.1033; $C_{11}H_{16}F_3NO_2Na$ requires 274.1025.

**(1*R*,4a*R*,5*R*,8a*S*)-5-methyldecahydronaphthalen-1-yl pivalate**

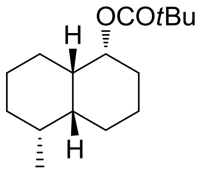


Colorless oil. $^1$H NMR (400 MHz, Chloroform-*d*) δ 4.75 (dt, *J* = 11.90, 4.85 Hz, 1H), 2.00 – 1.90 (m, 1H), 1.85 – 1.72 (m, 2H), 1.66 – 1.21 (m, 12H), 1.18 (s, 9H), 0.86 (d, *J* = 6.75 Hz, 3H). $^{13}$C NMR (100 MHz, Chloroform-*d*) δ 178.1, 75.6, 41.6, 41.5, 39.0, 35.9, 29.6, 27.4, 26.3, 26.2, 24.1, 20.0, 19.4, 18.5. $[\alpha]_D^{29}$ = 42.0 (c = 0.2, $CHCl_3$). **HRMS-ESI**: Found $[M+Na]^+$ = 275.1983; $C_{16}H_{28}NO_2Na$ requires 275.1982.

**(1*R*,4a*S*,5*R*,8a*S*)-5-methoxydecahydronaphthalen-1-yl pivalate**

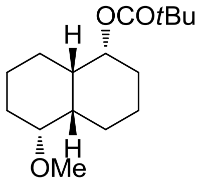


Colorless oil. $^1$H NMR (400 MHz, Chloroform-*d*) δ 4.76 (dt, *J* = 12.02, 4.89 Hz, 1H), 3.31 (s, 3H), 3.16 (dt, *J* = 11.78, 4.61 Hz, 1H), 2.10 (dq, *J* = 12.39, 4.17 Hz, 1H), 1.94 (td, *J* = 9.26, 4.58 Hz, 1H), 1.87 – 1.62 (m, 4H), 1.57 – 1.20 (m, 8H), 1.18 (s, 9H). $^{13}$C NMR (100 MHz, Chloroform-*d*) δ 178.1, 81.7, 75.1, 55.8, 39.6, 39.0, 38.8, 27.4, 26.9, 26.5, 23.9, 23.8, 19.2, 18.2. $[\alpha]_D^{29}$ = 89.5 (c = 0.2, $CHCl_3$). **HRMS-ESI**: Found $[M+Na]^+$ = 291.1937; $C_{16}H_{28}NO_3Na$ requires 291.1931.

**(1*R*,4a*R*,7S,8a*S*)-7-methyldecahydronaphthalen-1-yl pivalate**

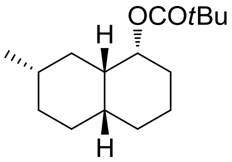


Colorless oil. $^1$H NMR (400 MHz, Chloroform-*d*) δ 4.76 (dt, *J* = 11.79, 4.86 Hz, 1H), 2.01 (dq, *J* = 12.78, 4.13 Hz, 1H), 1.82 – 1.66 (m, 2H), 1.61 – 1.24 (m, 10H), 1.18 (s, 9H), 1.16 – 1.01 (m, 2H), 0.91 (d, *J* = 6.42 Hz, 3H). $^{13}$C NMR (100 MHz, Chloroform-*d*) δ 178.1, 75.5, 40.1, 39.0, 35.1, 32.7, 31.7, 30.2, 28.7, 27.4, 25.8, 24.5, 24.3, 23.2. $[\alpha]_D^{29}$ = 46.0 (c = 0.2, $CHCl_3$). **HRMS-ESI**: Found $[M+Na]^+$ = 275.1983; $C_{16}H_{28}NO_2Na$ requires 275.1982.

## 6. Assignment of the absolute configurations of hydrogenated products.

The absolute configuration of hydrogenated product **3d** was determined as shown in Fig. S1. Firstly, compound **1d** was subjected to hydrogenation to give compound **5d**, which was confirmed as *R* configuration.[13] Compound **1d** was then hydrogenated by same Rh precursor and ligand to yield product **3d**. Finally, deprotection of compound **3d** gave compound **7**, which was reported in literature.[14]

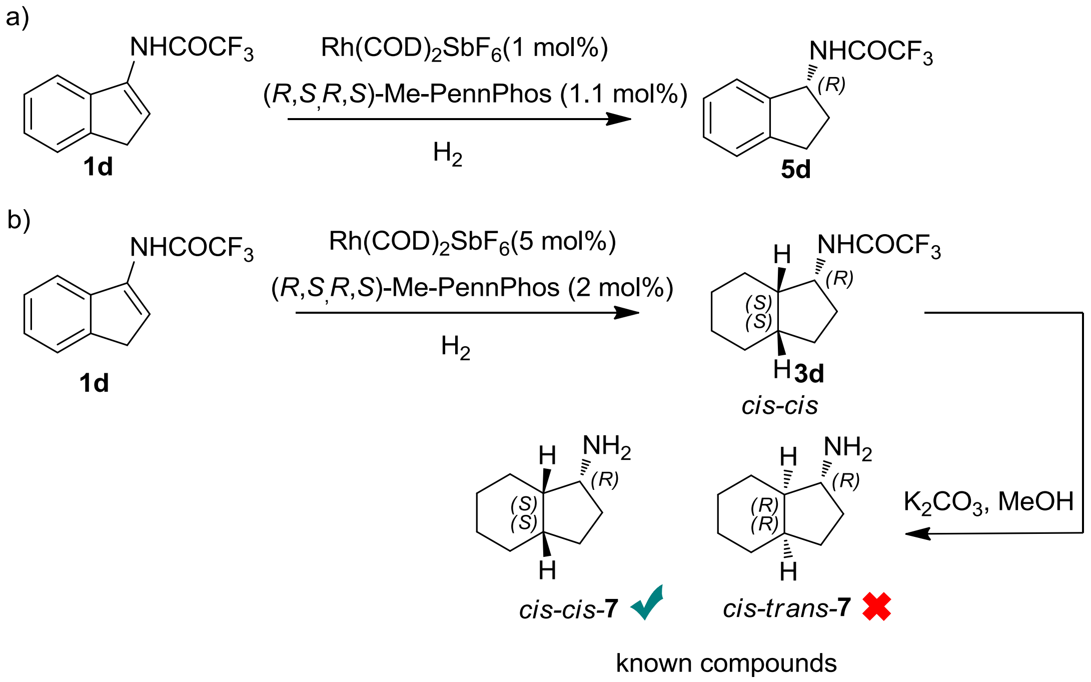


**Figure S1.** Assignment of the absolute configurations of hydrogenated products **3d**.

The absolute configuration of another hydrogenated product **3n** was determined as shown in Figure S2. Firstly, 1-tetralone was fully hydrogenated to give the mixtures of 1-decalols. The diastereomer **9** was then separated from the mixture, and the relative configuration was confirmed by NMR spectra according to literature.[15] Finally, the absolute configuration of **3n** was confirmed by comparison of the NMR data with ester derivative of **9**.

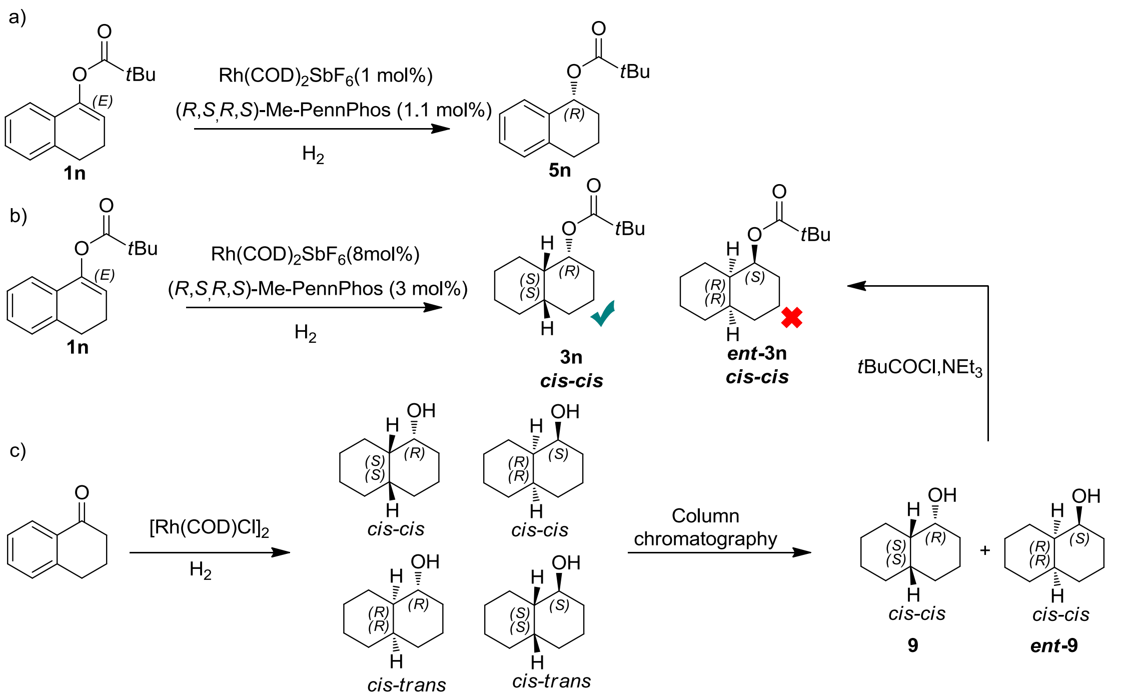


**Figure S2.** Assignment of the absolute configurations of hydrogenated product **3n**.

## 7. Scale-up asymmetric hydrogenations and applications

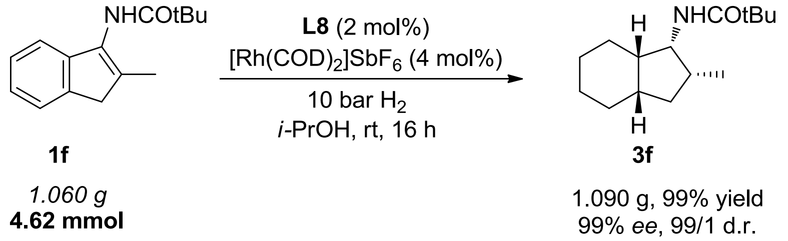


[Rh(COD)$_2$]SbF$_6$ (102 mg, 0.18mmol 4 mol%) was added to a glassed cylinder with a stirring bar. A stock solution of Pennphos (2 mg/ml) in *i*-PrOH (16.5 ml, 33 mg, 2 mol%) was added to the cylinder and stirred for 30 min under argon. Substrate **1f** (1.060g, 4.62 mmol) was dissolved in 10 ml *i*-PrOH and then was added to the cylinder under argon. The cylinder was placed in a high hydrogenation apparatus and then was purged three times with argon and three time with $H_2$ before the indicated pressure (10 bar) was set. The reaction was stirred at room temperature for 16 hours before the $H_2$ pressure was released and the solvent was removed *in vacuo*. The crude product was filtered through on a short plug of silica to yield the product **3f** (1.090 g, 99% yield), d.r and *ee* value was determined by GC using a chiral stationary phase.

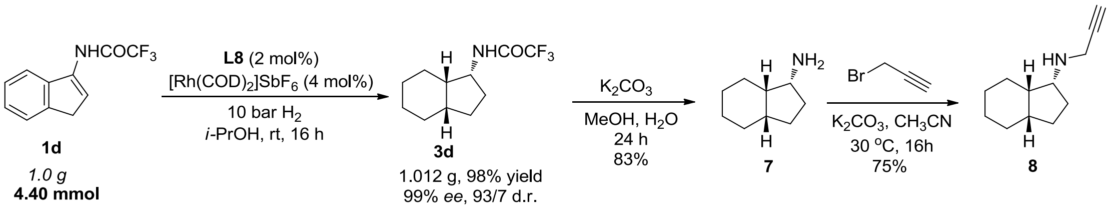


Step 1: Hydrogenation was performed using the same procedure as the above case.

Step 2: Deprotection: To a solution of **3d** (235 mg, 1.0 mmol) in 10 ml MeOH/$H_2O$(1:1) was add $K_2CO_3$ (276 mg, 2.0 mol) and the mixture was stirred at ambient temperature for 24 h before the volatles were removed in vacuo. The residue was partitioned between $CH_2Cl_2$ and saturated aqueous $NaHCO_3$. The organic layer was separated and washed with brine, died over $Na_2SO_4$ and concentrated to give the product **7** as yellowish oil (115 mg, 83% yield).

Step 3: Alkylation: $K_2CO_3$ (120mg, 0.84 mmol, 1.2 eq.) was added to a solution of **7** (100mg, 0.72 mmol) in 5 ml acetonitrile. Propargyl bromide (150 mg, 80% w.t. in toluene, 0.72 mmol) was added to the reaction mixture with stirring at 30 ℃. After stirring at 30 ℃ for 12 h, potassium carbonate was filtered off, whereupon acetonitrile was removed, and the residue was purified by column chromatography (EtOAc: pentane =1:1). The product **8** was obtained as yellowish oil (95 mg, 75% yield).

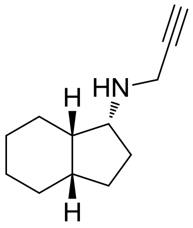


$^1$H NMR (400 MHz, Chloroform-*d*) δ 3.44 (d, J = 2.5 Hz, 2H), 3.29 (td, J = 9.0, 5.6 Hz, 1H), 2.57 (s, 1H), 2.23 (t, J = 2.5 Hz, 1H), 2.14 – 1.87 (m, 3H), 1.76 – 1.60 (m, 2H), 1.60 – 1.42 (m, 6H), 1.41 – 1.28 (m, 1H), 1.20 – 0.97 (m, 2H). $^{13}$C NMR (100 MHz, Chloroform-*d*) δ 81.81, 71.73, 61.94, 41.12, 37.17, 37.12, 28.84, 27.11, 25.16, 24.18, 21.47, 21.00. $[\alpha]_D^{29}$ = 14.0 (c = 0.2, $CHCl_3$). **HRMS-ESI**: Found $[M+Na]^+$ = 200.1417; $C_{16}H_{28}NO_2Na$ requires 200.1415.

## 8. Additional experiments and HR-TEM images of *in-situ* generated Rh-nanoparticles.

### Doping experiment with benzothiophene

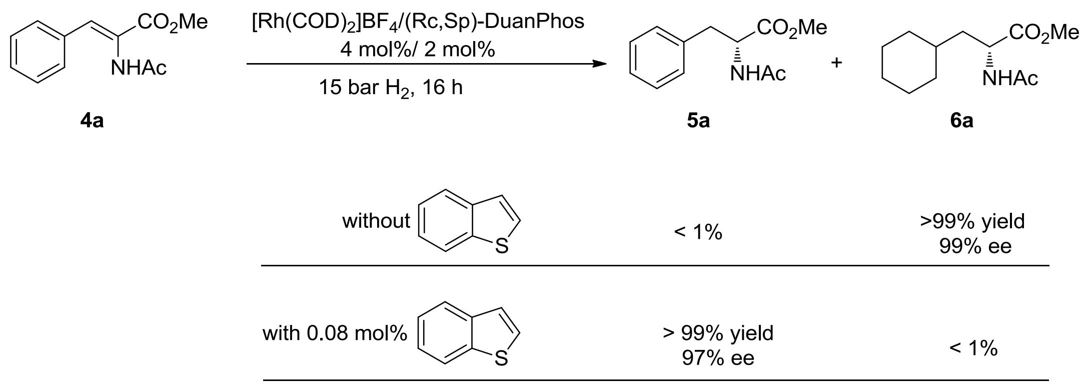


### Investigations concerning the stability of homogeneous Rh/diphosphine catalyst under the hydrogenation conditions.

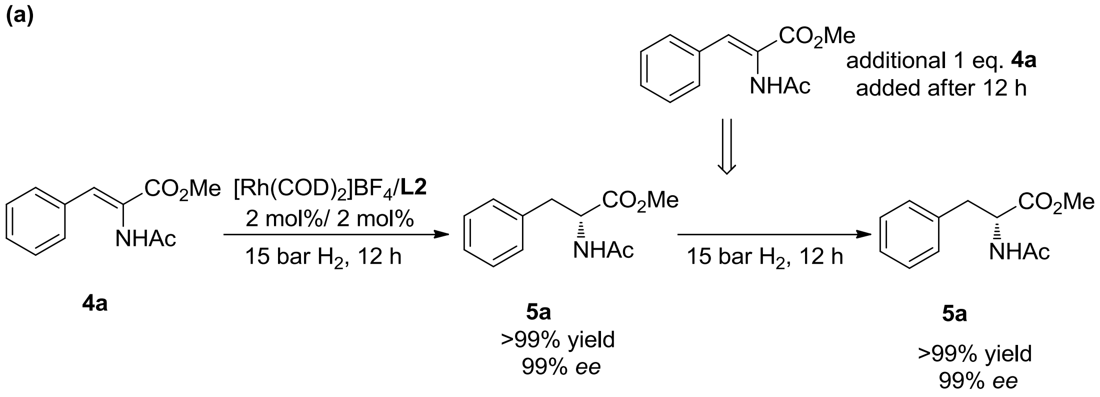


$[Rh(COD)_2]BF_4$ was used in a ratio of 1:1 with diphosphine ligand **L2** to form the homogeneous Rh/diphosphine catalyst for the asymmetric hydrogenation of olefin. After 12 h, the olefin hydrogenation was completed and another 1 equivalent of substrate **4a** was added to the reaction mixture and then subjected to hydrogenation again. No aromatic hydrogenation product was observed, and the enantioselectivity for the olefin hydrogenation was not affected (99% ee). The results indicate that the homogeneous Rh/diphosine catalyst is stable under olefin hydrogenation conditions, and no in-situ formation of Rh-nanoparticles occurred.

(b)

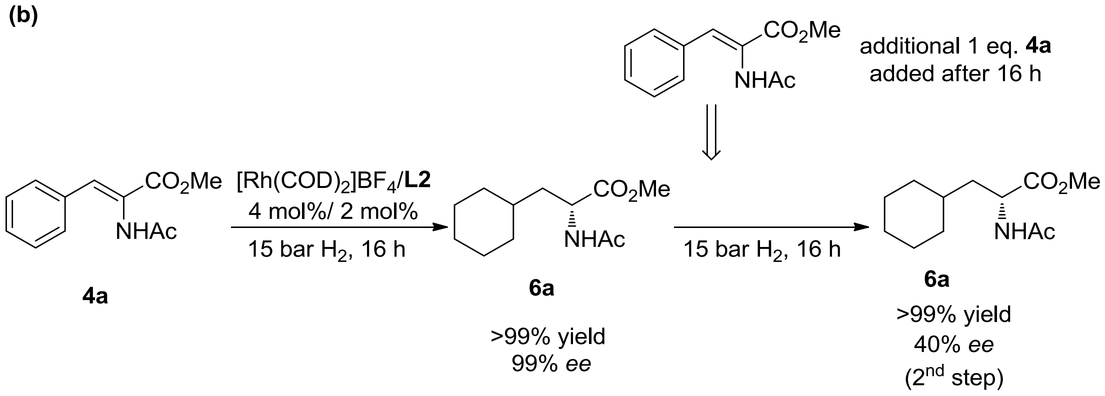


$[Rh(COD)_2]BF_4$ was used in a ratio of 2:1 with diphosphine ligand **L2** for the hydrogenation of both olefin and phenyl ring. After 16 h, a full conversion to the complete hydrogenation product **5a** (99% ee) was obtained. Another 1 equivalent of substrate **4a** was added to the reaction mixture and then subjected to hydrogenation for 16 h. In this case, the complete hydrogenation of the second portion of substrate **4a** was observed. Lower *ee* (40% ee) was obtained for the second hydrogenation might due to the competition hydrogenation of olefin by the in-situ generated Rh-nanoparticles.

(c)

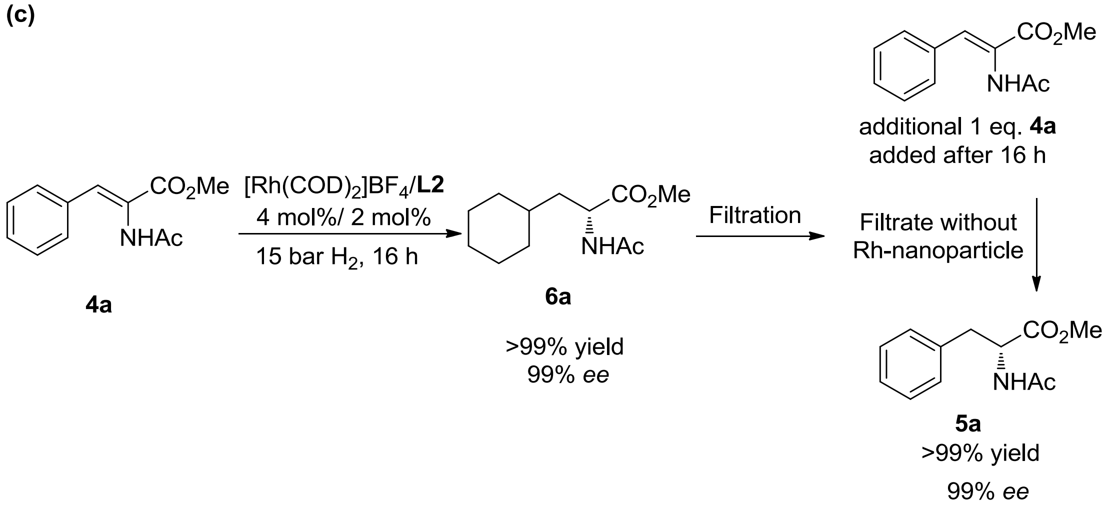


In this experiment, the same conditions as (**b**) were applied for the first step. After 16 h, hydrogen atmosphere was released and the sample was left stand for 1 h under argon (aggregation of nanoparticles). The aggregated Rh-nanoparticles were then removed from the mixture by filtration under argon. Another 1 equivalent of **4a** was added to the filtrate and subjected to the hydrogenation conditions. In this case, only olefin hydrogenation was observed for the second portion of substrate **4a**, and the

enantioselectivity was not affected (99% ee of **5a**). Although not conclusive, the same level of ee (99%) was retained in the second olefin hydrogenation indicates that the in-situ generated Rh-nanoparticles might not influence the stability of homogeneous Rh/diphosphine catalyst.

**HR-TEM images of *in-situ* generated Rh-nanoparticles from complete hydrogenation of 1a.**

**Transmission Electron Microscopy.** TEM images were obtained on a JEOL JEM2100F microscope operated at 200 kV (Cs 1.0 mm, point resolution 0.23 nm) with a Gatan Ultrascan 1000 CCD camera (resolution 2,048 × 2,048 pixels, pixel size 14 μm).

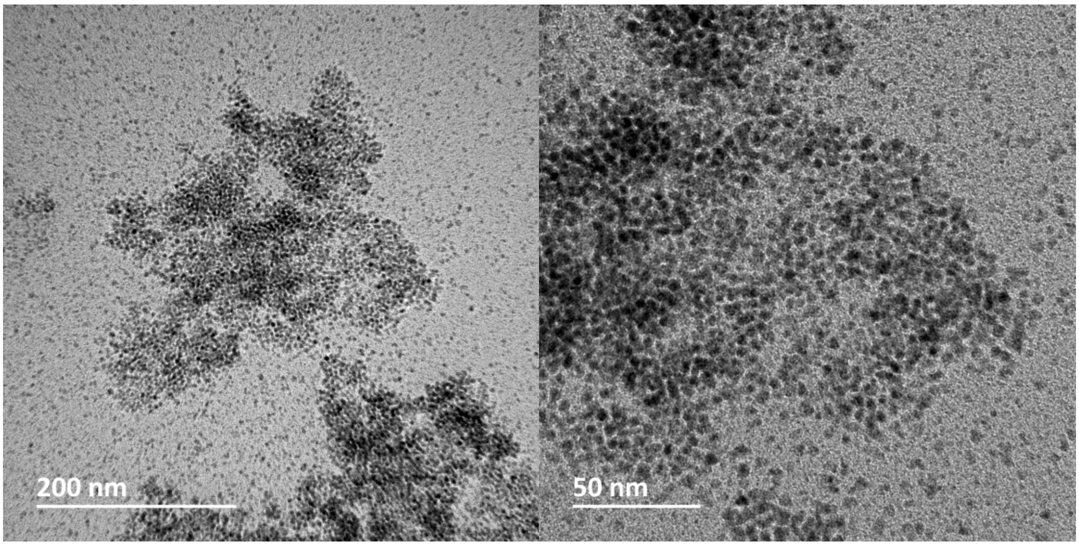


**Figure S3.** TEM images of Rh nanoparticles showing size of 3-5 nm.

## 9. $^{1}$H, $^{13}$C and $^{1}$F NMR spectra for new compounds

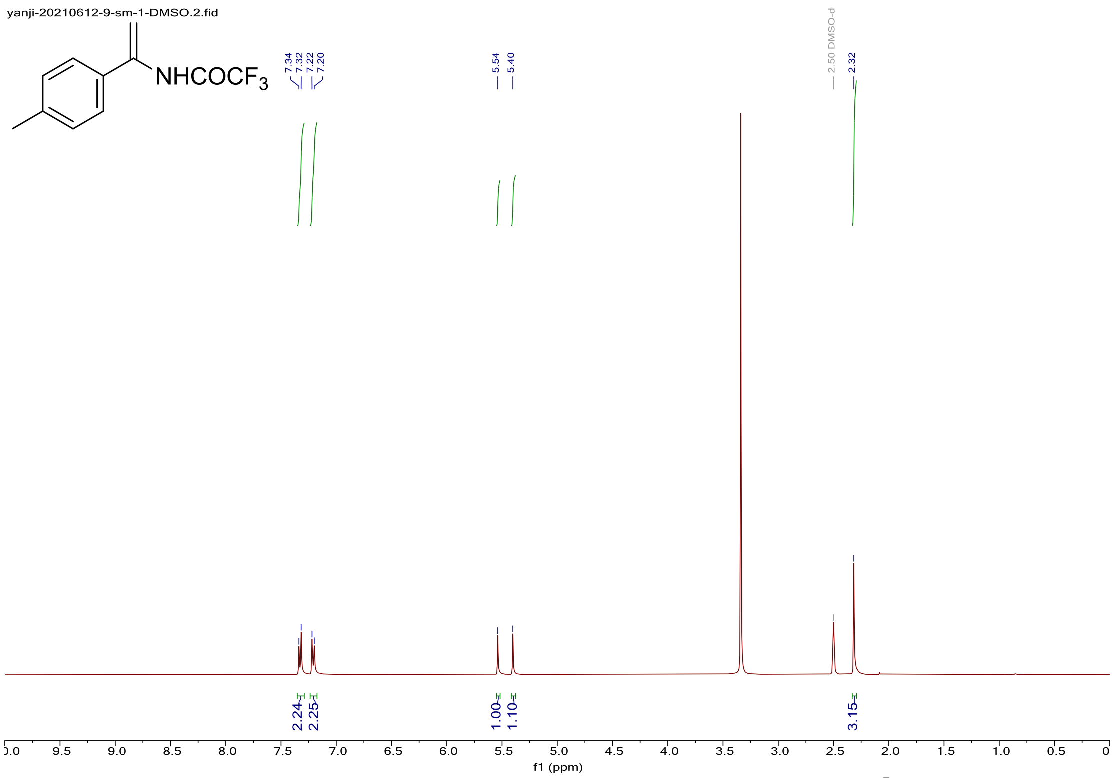


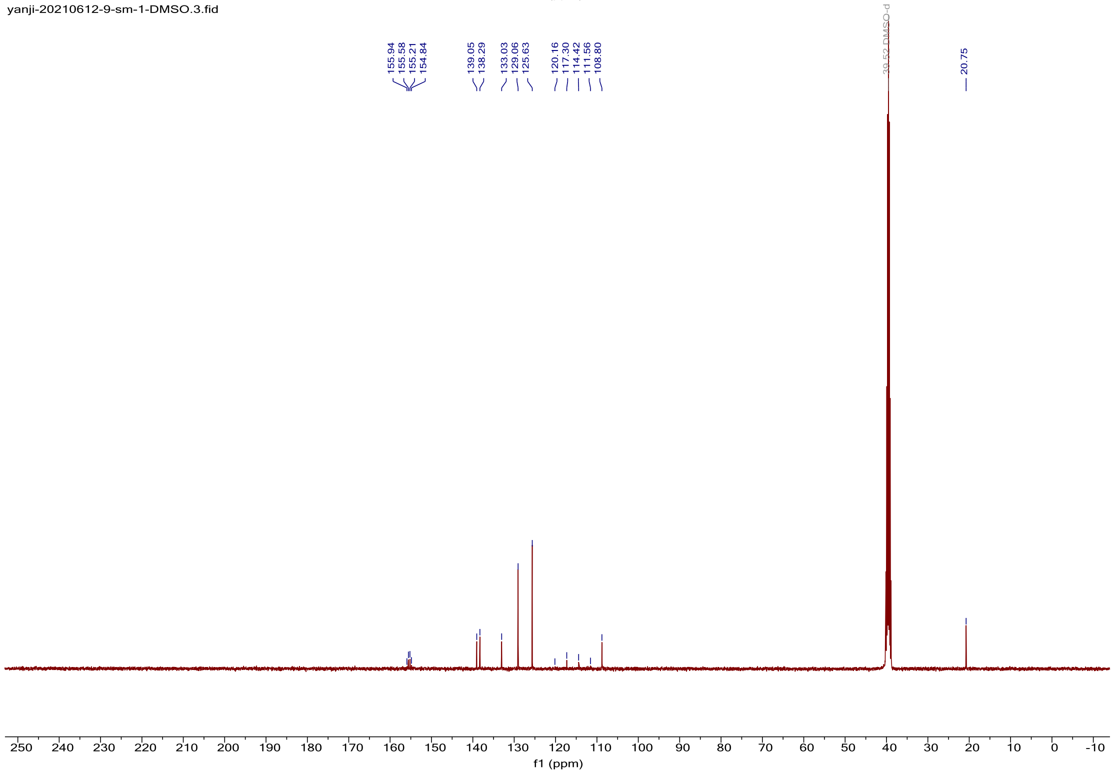

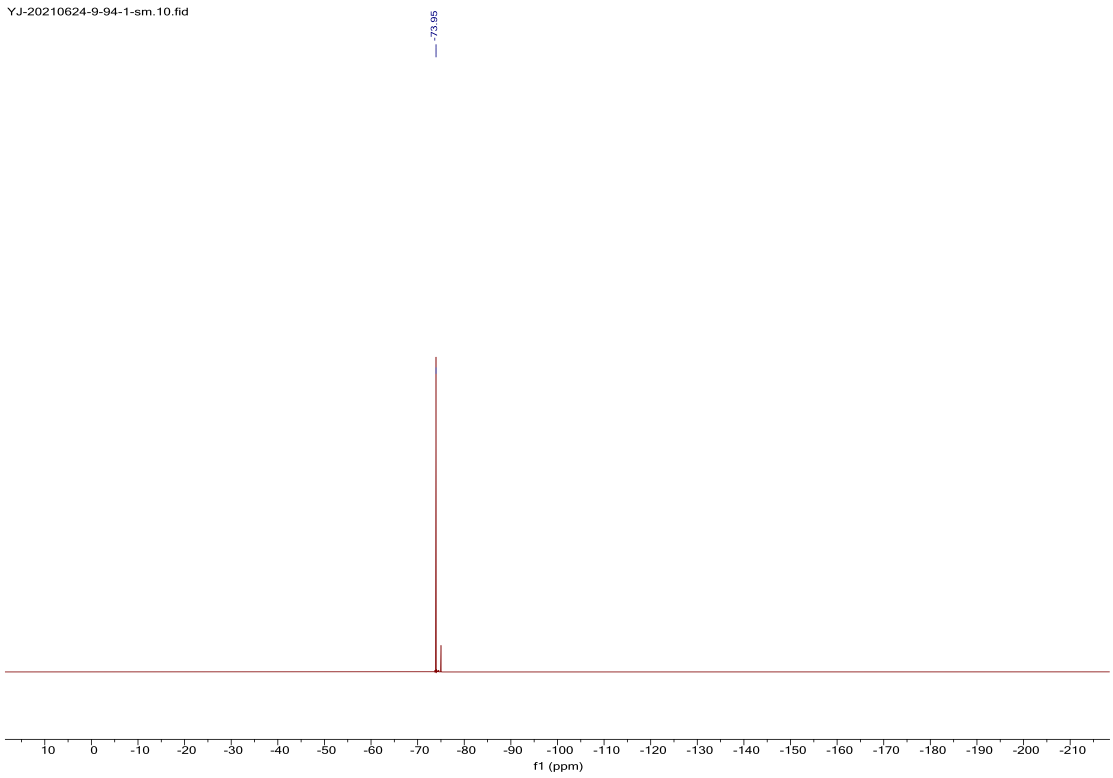
YJ-20210624-9-94-1-sm.10.fid
-73.95
10 0 -10 -20 -30 -40 -50 -60 -70 -80 -90 -100 -110 -120 -130 -140 -150 -160 -170 -180 -190 -200 -210
f1 (ppm)

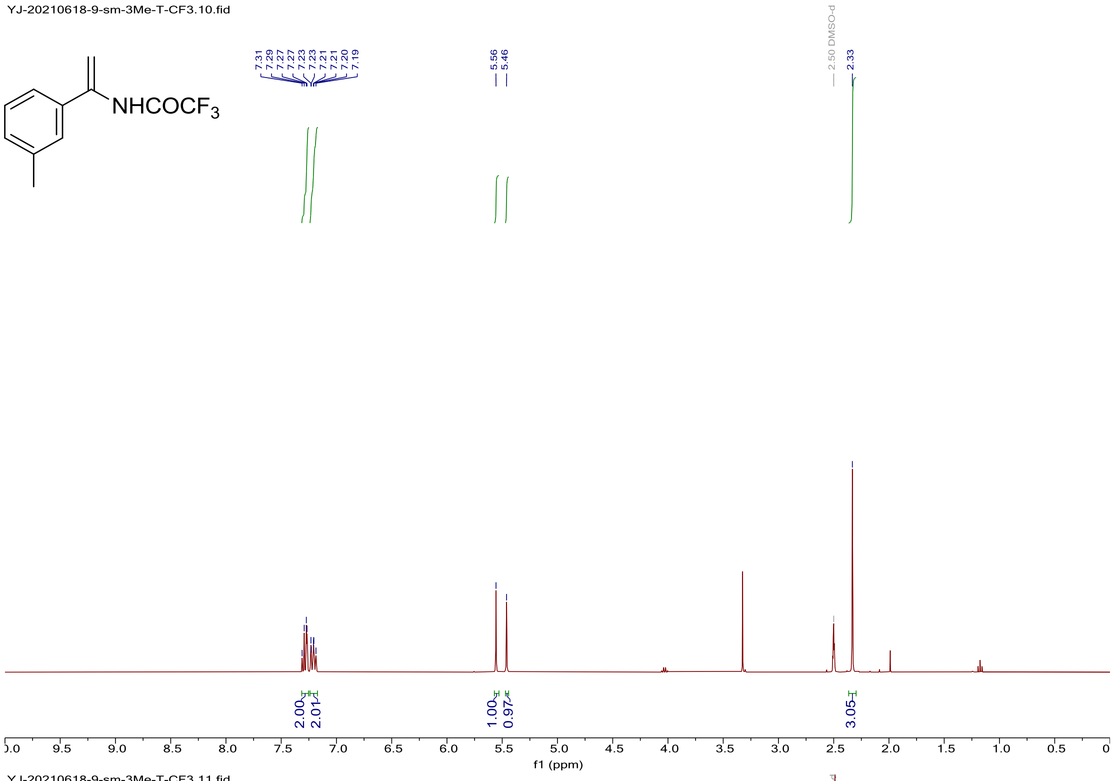
YJ-20210618-9-sm-3Me-T-CF3.10.fid
NHCOCF3
7.31
7.29
7.27
7.27
7.23
7.23
7.21
7.21
7.20
7.19
5.56
5.46
2.50 DMSO-d
2.33
2.00
2.01
1.00
0.97
3.05
f1 (ppm)

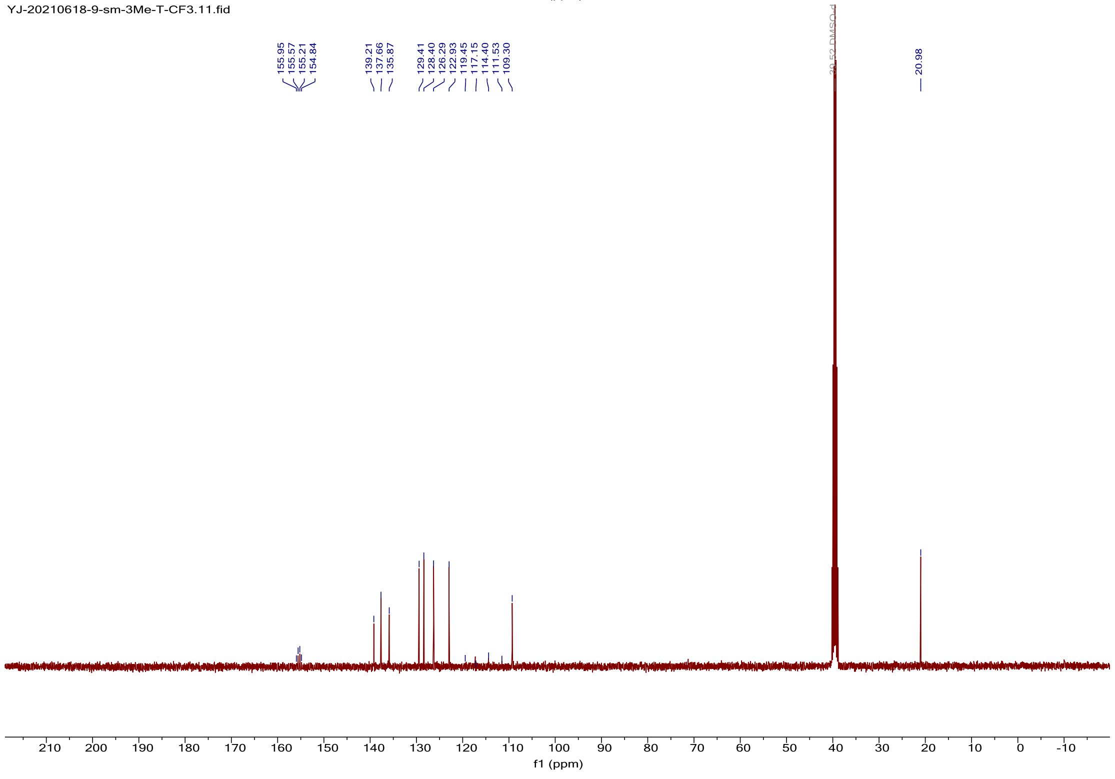
YJ-20210618-9-sm-3Me-T-CF3.11.fid
155.95
155.57
155.21
154.84
139.21
137.66
135.87
129.41
128.40
126.29
122.93
119.45
117.15
114.40
111.53
109.30
20.98
f1 (ppm)

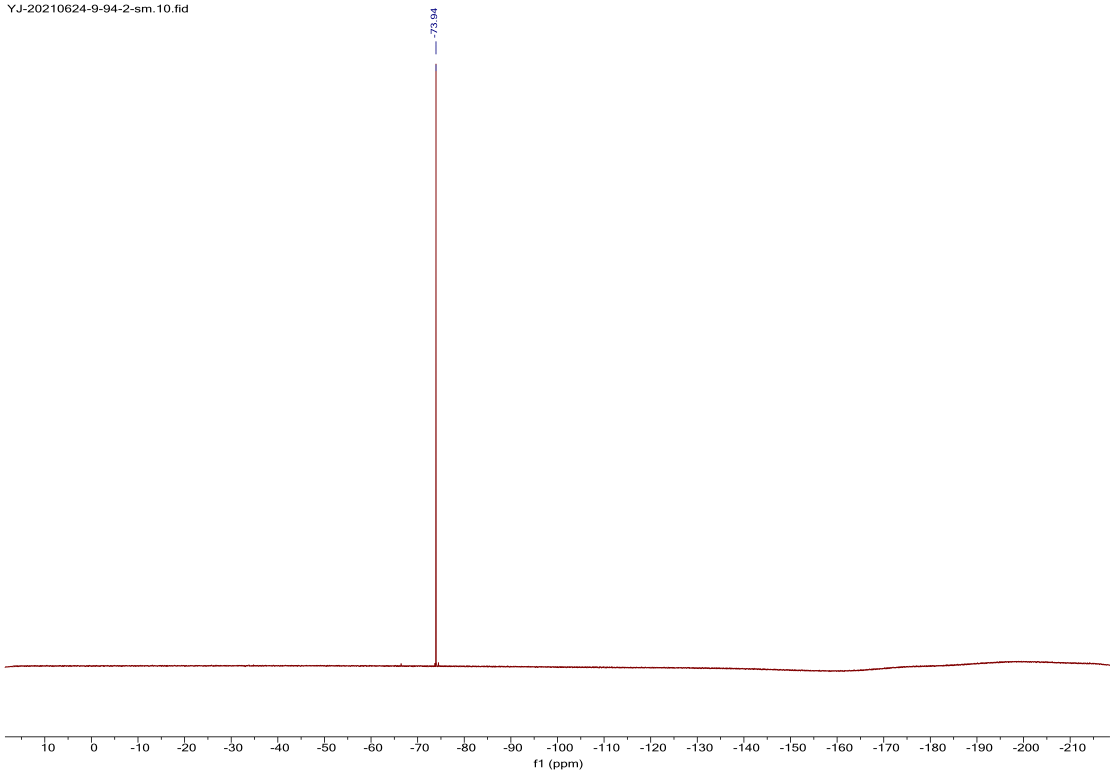

YJ-20210624-9-94-2-sm.10.fid
-73.94
10
0
-10
-20
-30
-40
-50
-60
-70
-80
-90
-100
-110
-120
-130
-140
-150
-160
-170
-180
-190
-200
-210
f1 (ppm)

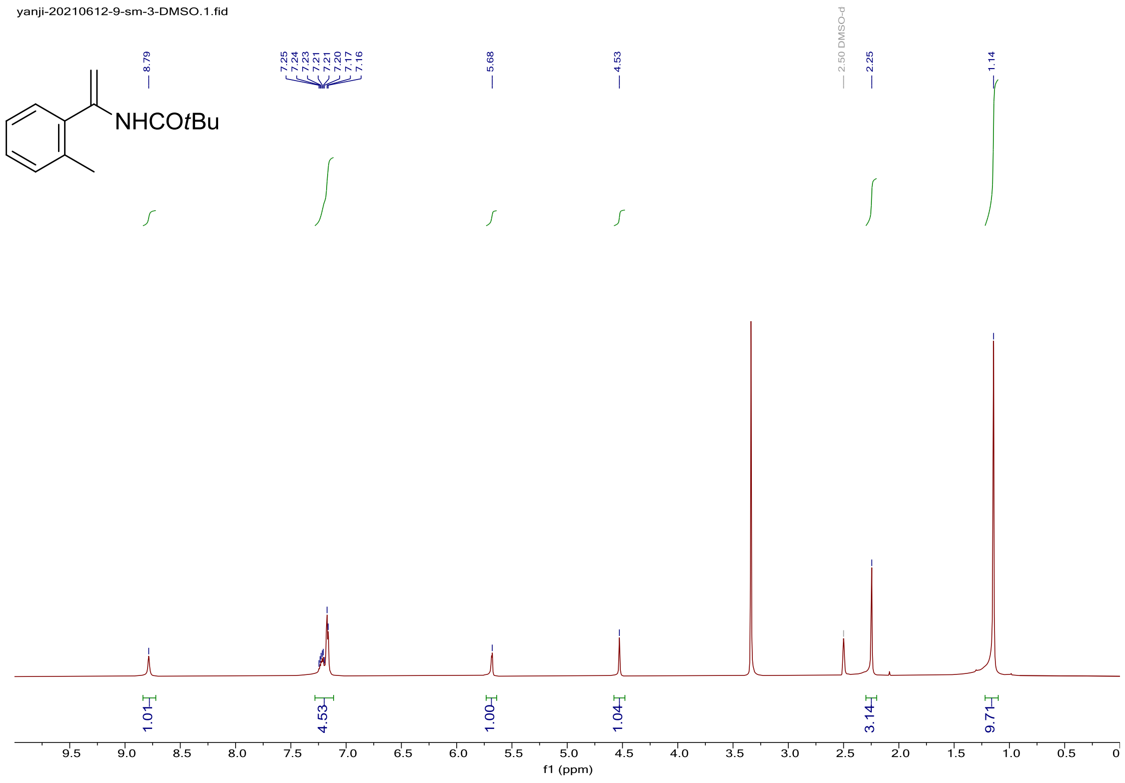
yanji-20210612-9-sm-3-DMSO.1.fid
NHCOtBu
8.79
7.25
7.24
7.23
7.21
7.21
7.20
7.17
7.16
5.68
4.53
2.50 DMSO-d
2.25
1.14
1.01
4.53
1.00
1.04
3.14
9.71
f1 (ppm)

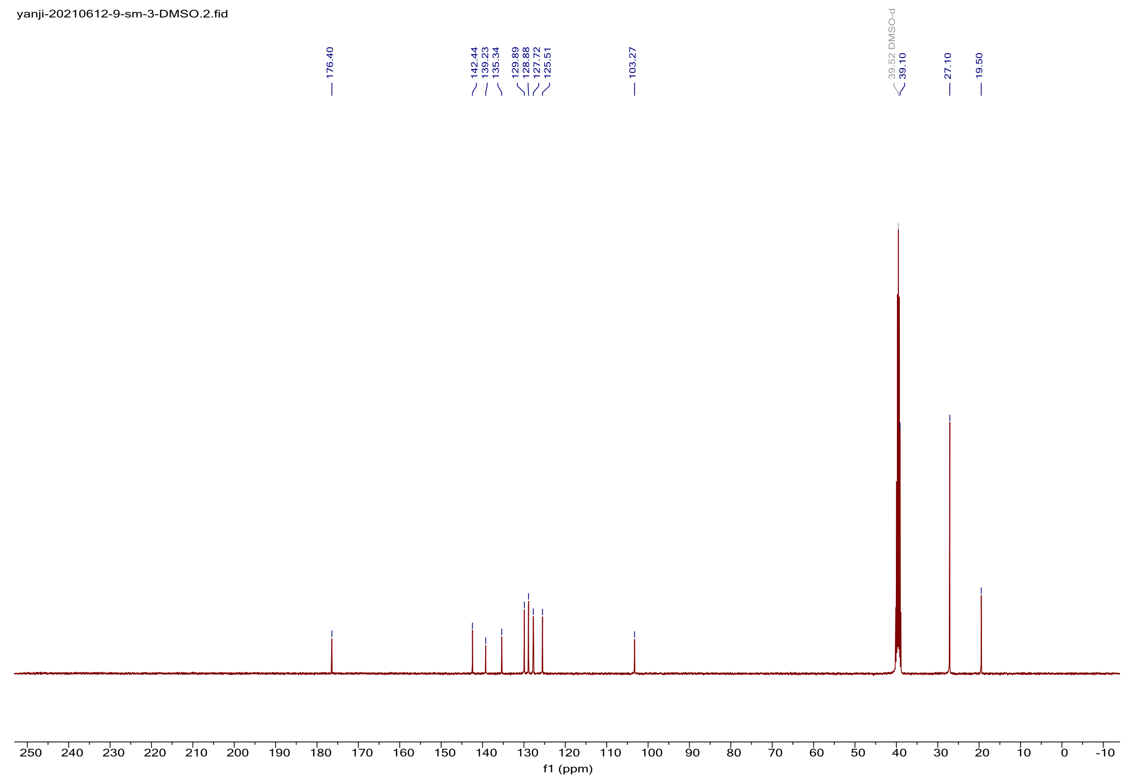
yanji-20210612-9-sm-3-DMSO.2.fid
176.40
142.44
139.23
135.34
129.89
128.88
127.72
125.51
103.27
39.52 DMSO-d
39.10
27.10
19.50
f1 (ppm)

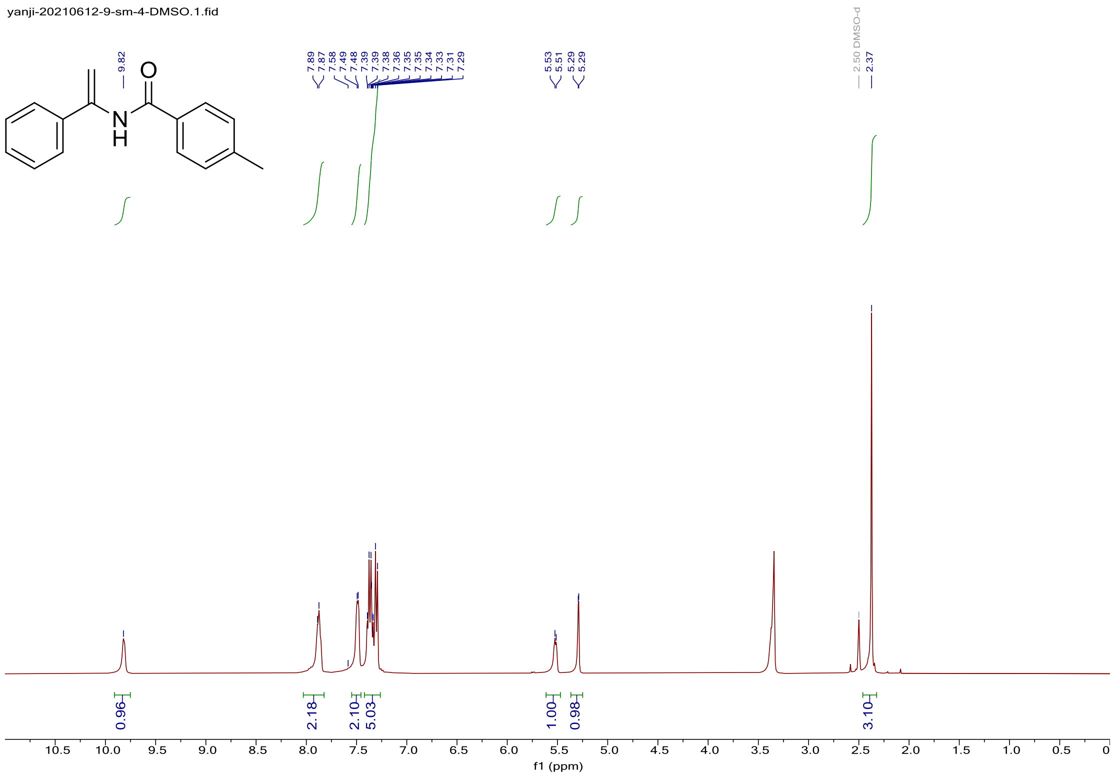
yanji-20210612-9-sm-4-DMSO.1.fid
9.82
7.89 7.87 7.58 7.49 7.48 7.39 7.39 7.38 7.36 7.35 7.34 7.33 7.31 7.29
5.53 5.51 5.29 5.29
2.50 DMSO-d
2.37
O
N
H
0.96
2.18
2.10
5.03
1.00
0.98
3.10
f1 (ppm)

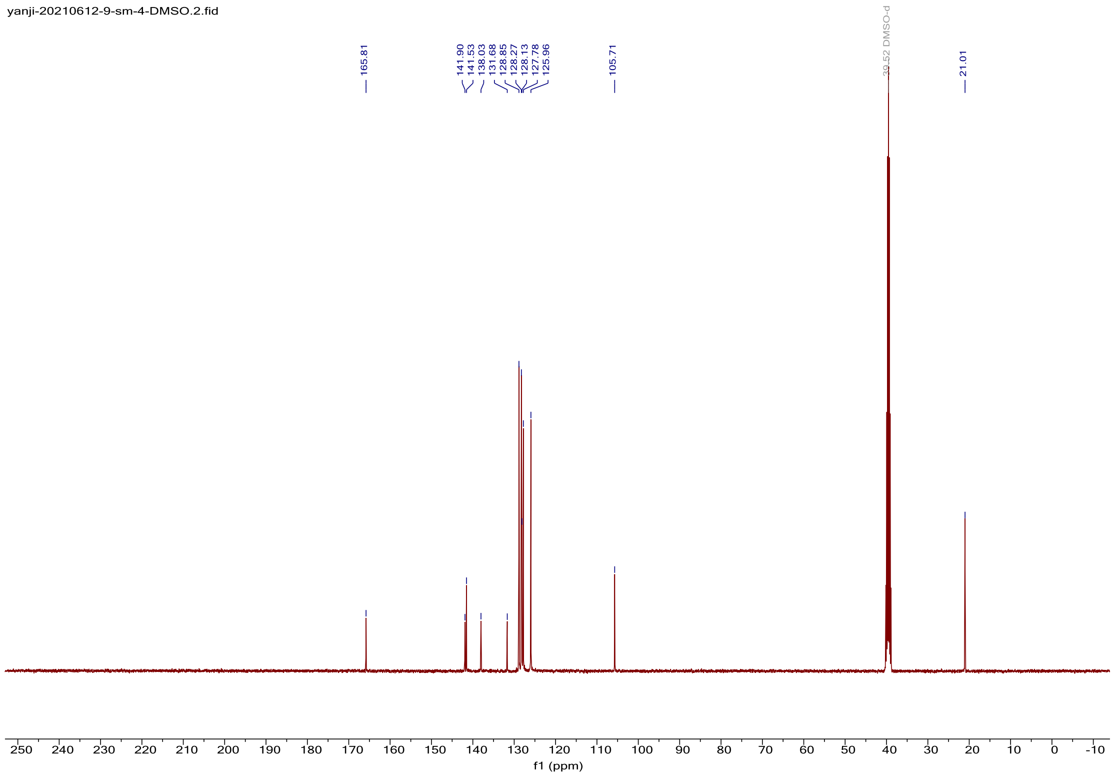
yanji-20210612-9-sm-4-DMSO.2.fid
165.81
141.90 141.53 138.03 131.68 128.85 128.27 128.13 127.78 125.96
105.71
39.52 DMSO-d
21.01
f1 (ppm)

YJ-20210624-9-4-3-sm.10.fid

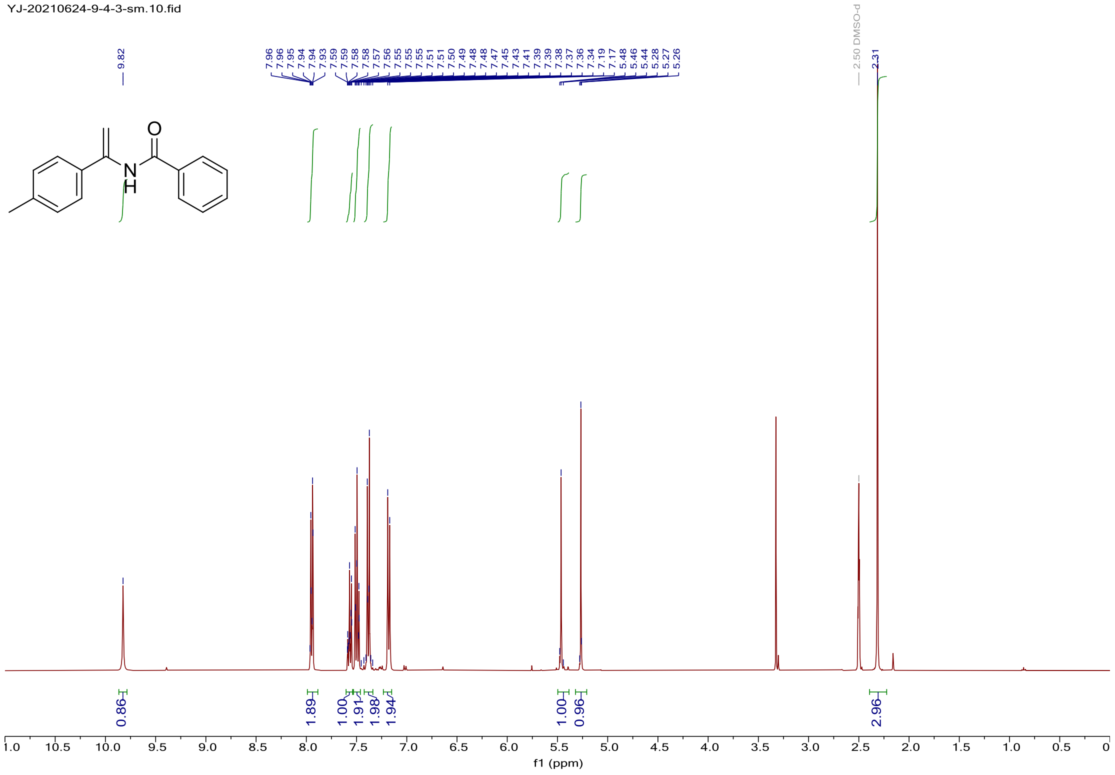

yanji-20210612-9-sm-5-DMSO.2.fid

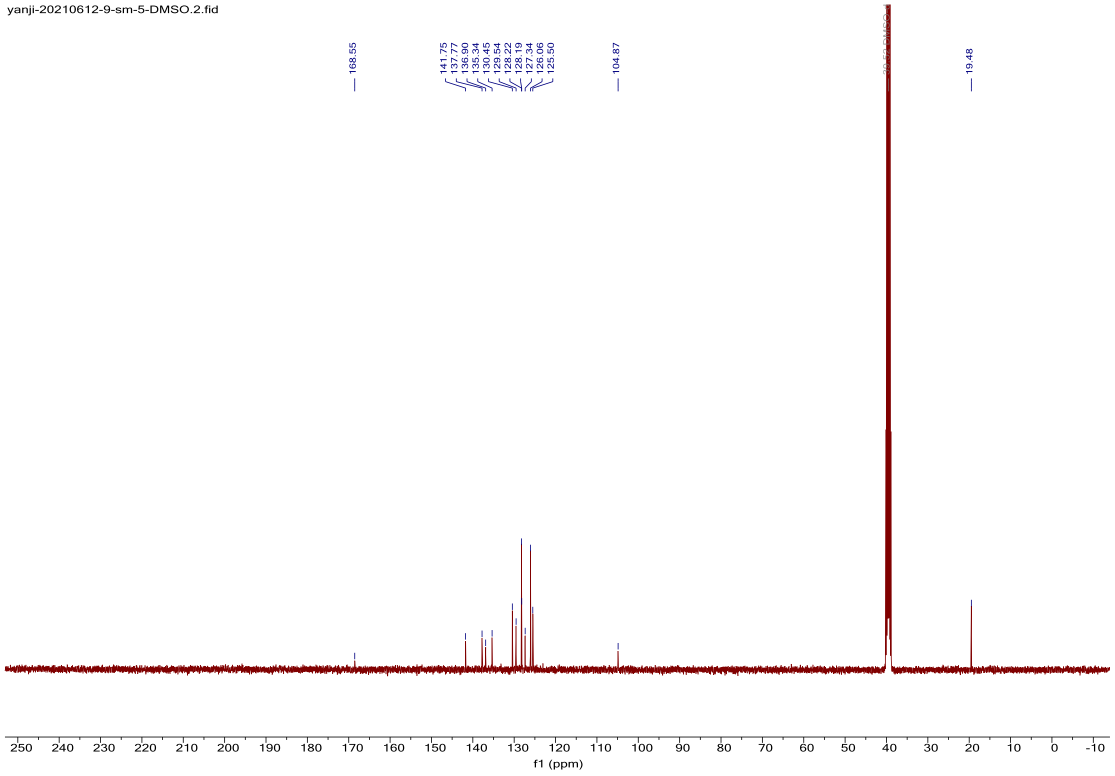

hwu-210621-206-82fsmdmso.10.fid

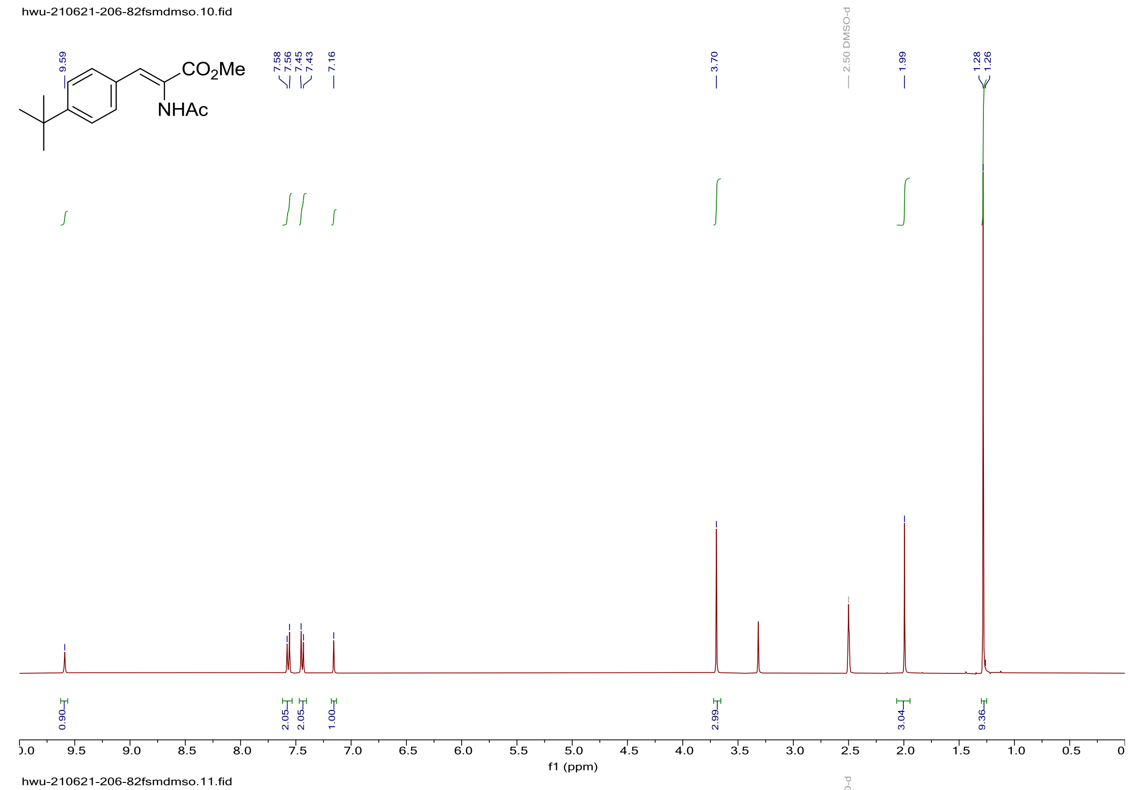


hwu-210621-206-82fsmdmso.11.fid

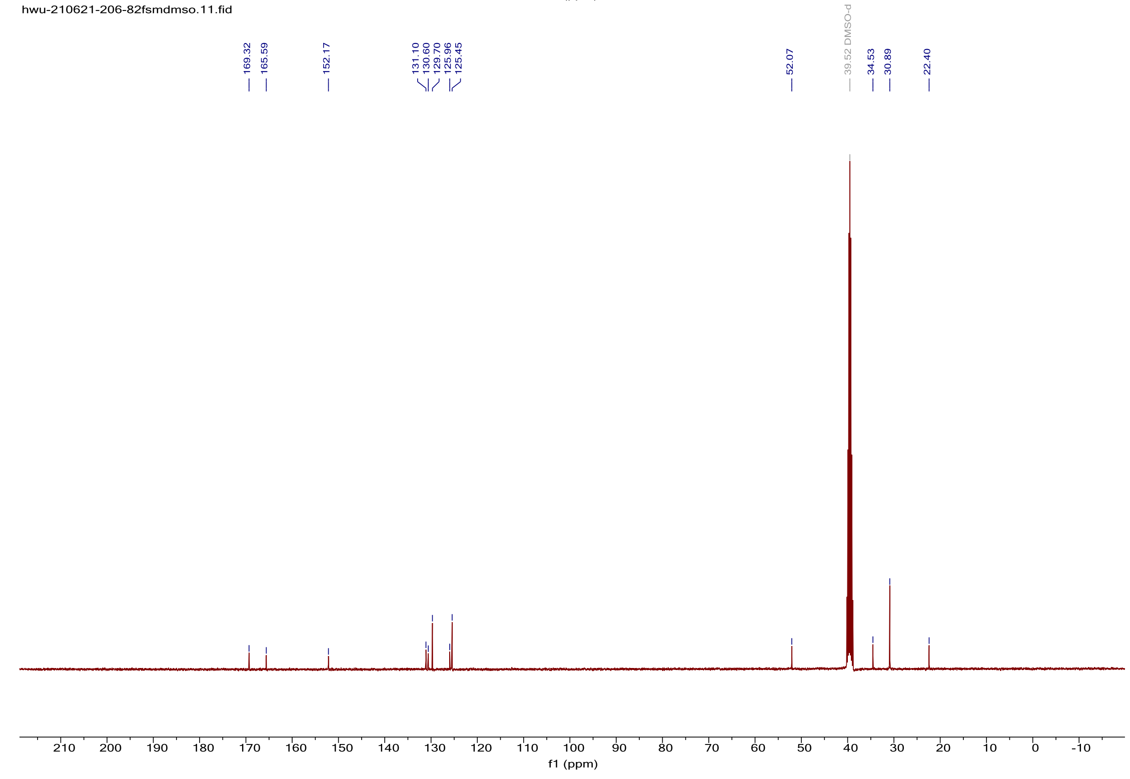

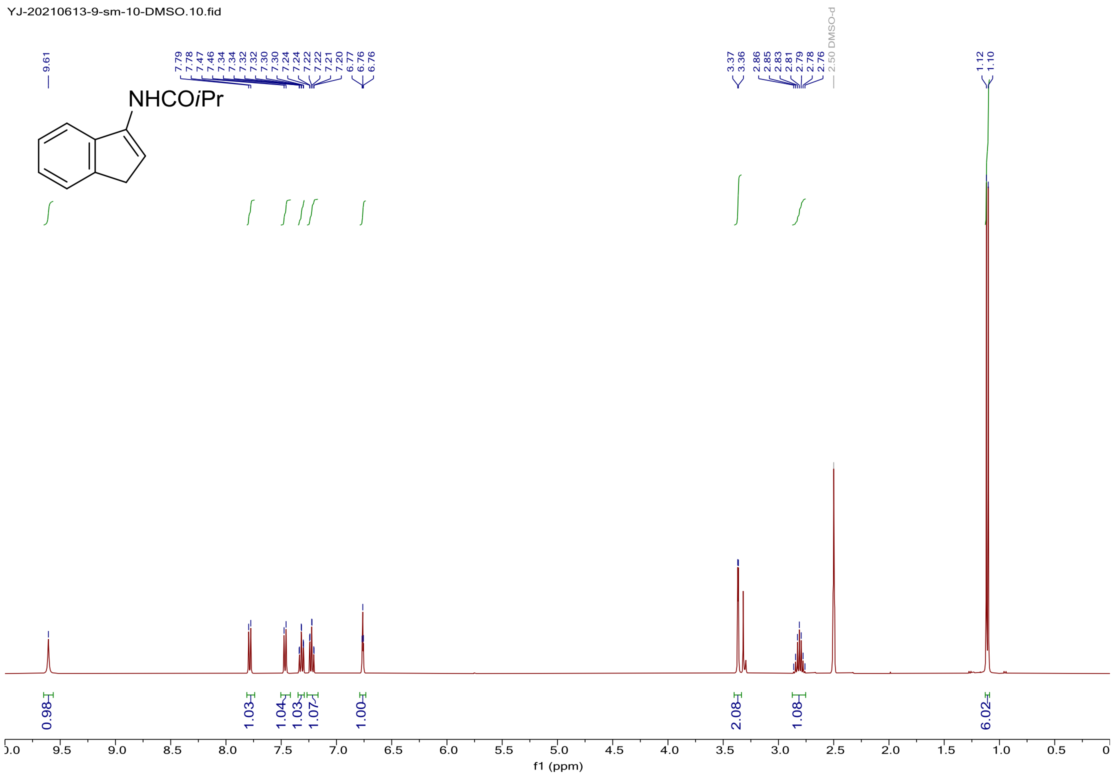
YJ-20210613-9-sm-10-DMSO.10.fid
NHCO*i*Pr
9.61
7.79 7.78 7.47 7.46 7.34 7.34 7.32 7.32 7.30 7.30 7.24 7.24 7.22 7.22 7.21 7.20 6.77 6.76 6.76
3.37 3.36 2.86 2.85 2.83 2.81 2.79 2.78 2.76
2.50 DMSO-d
1.12 1.10
0.98 1.03 1.04 1.03 1.07 1.00 2.08 1.08 6.02
f1 (ppm)

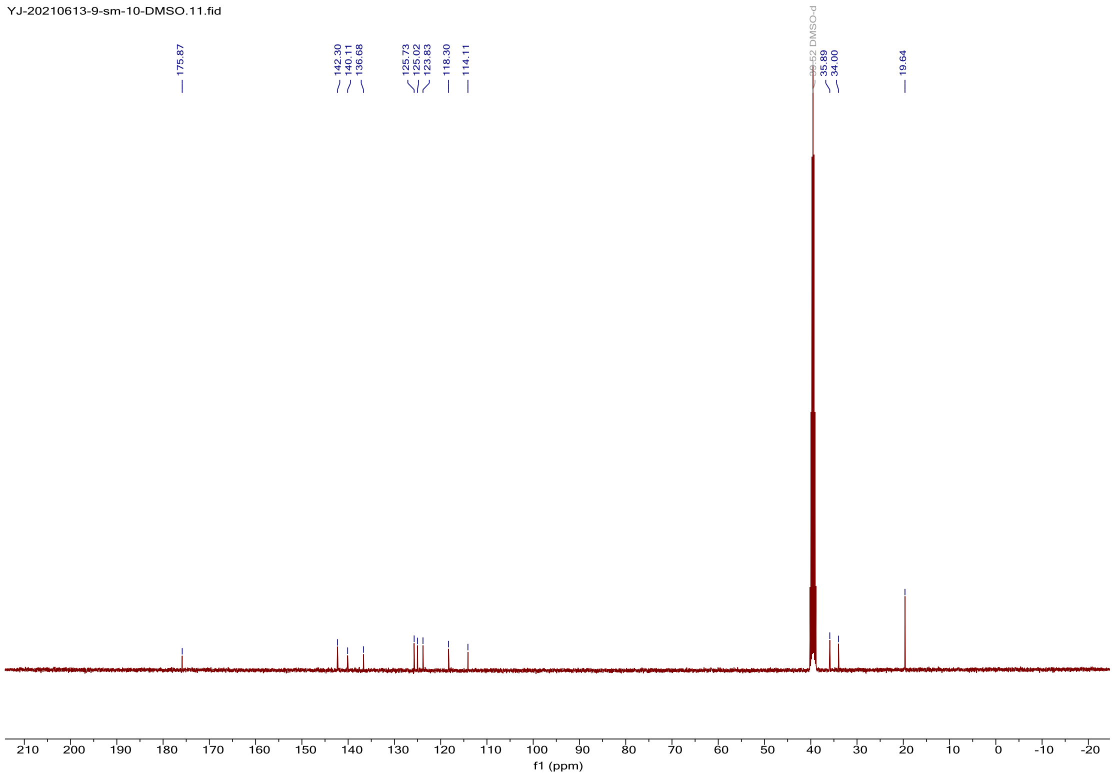
YJ-20210613-9-sm-10-DMSO.11.fid
175.87
142.30 140.11 136.68
125.73 125.02 123.83
118.30
114.11
39.52 DMSO-d
35.89 34.00
19.64
f1 (ppm)

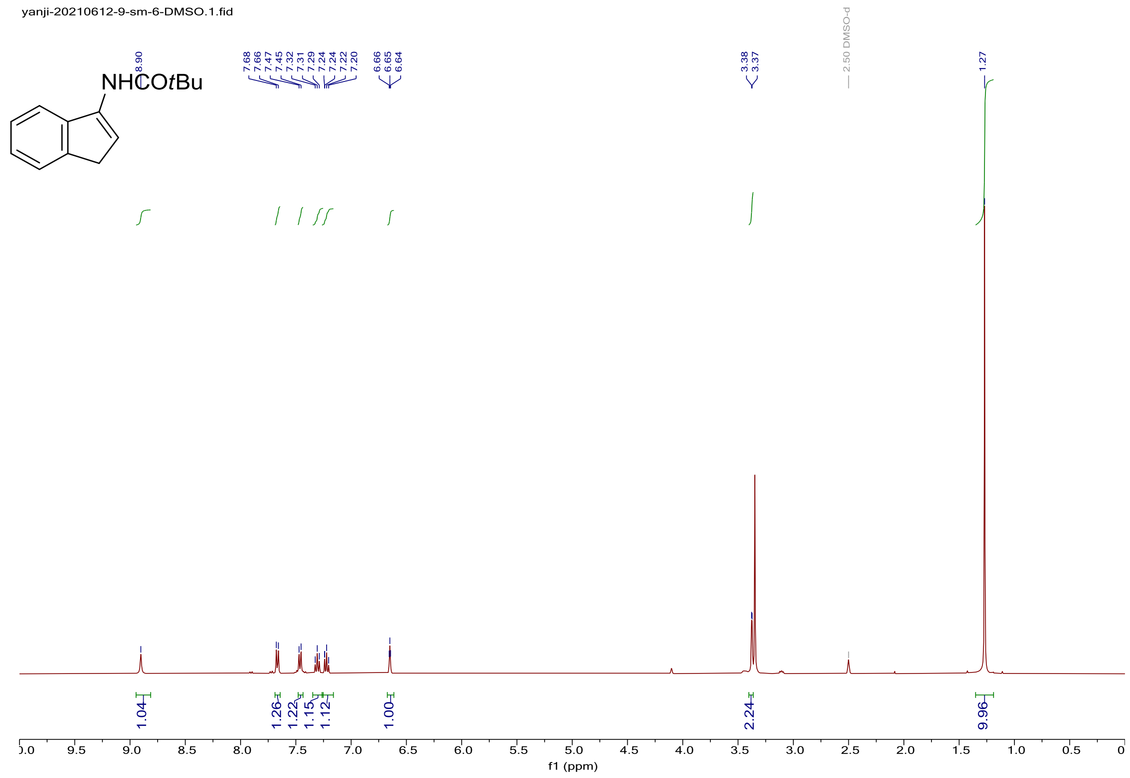
yanji-20210612-9-sm-6-DMSO.1.fid
NHCOtBu
8.90
7.68
7.66
7.47
7.45
7.32
7.31
7.29
7.24
7.24
7.22
7.20
6.66
6.65
6.64
3.38
3.37
2.50 DMSO-d
1.27
1.04
1.26
1.22
1.15
1.12
1.00
2.24
9.96
f1 (ppm)

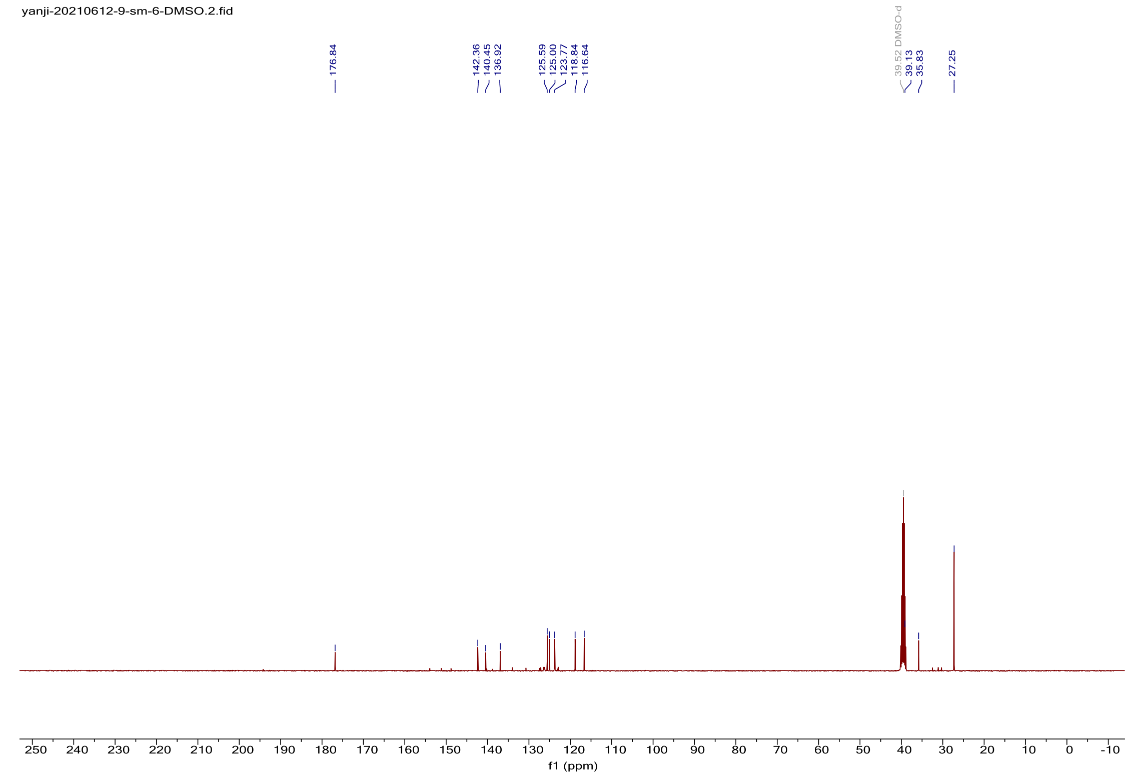
yanji-20210612-9-sm-6-DMSO.2.fid
176.84
142.36
140.45
136.92
125.59
125.00
123.77
118.84
116.64
39.52 DMSO-d
39.13
35.83
27.25
f1 (ppm)

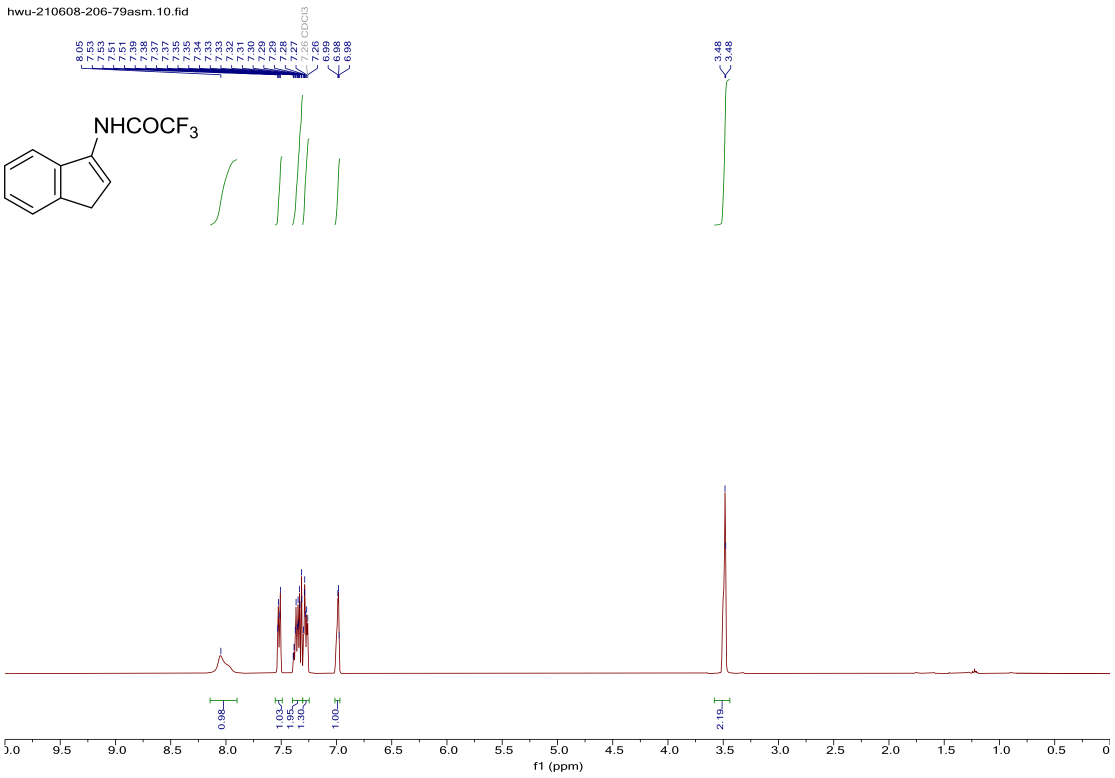
hwu-210608-206-79asm.10.fid
NHCOCF3
8.05 7.53 7.53 7.51 7.51 7.39 7.38 7.37 7.37 7.35 7.35 7.34 7.33 7.33 7.32 7.31 7.30 7.29 7.29 7.28 7.27 7.26 CDCl3 7.26 6.99 6.98 6.98
3.48 3.48
0.98 1.03 1.95 1.30 1.00 2.19
f1 (ppm)

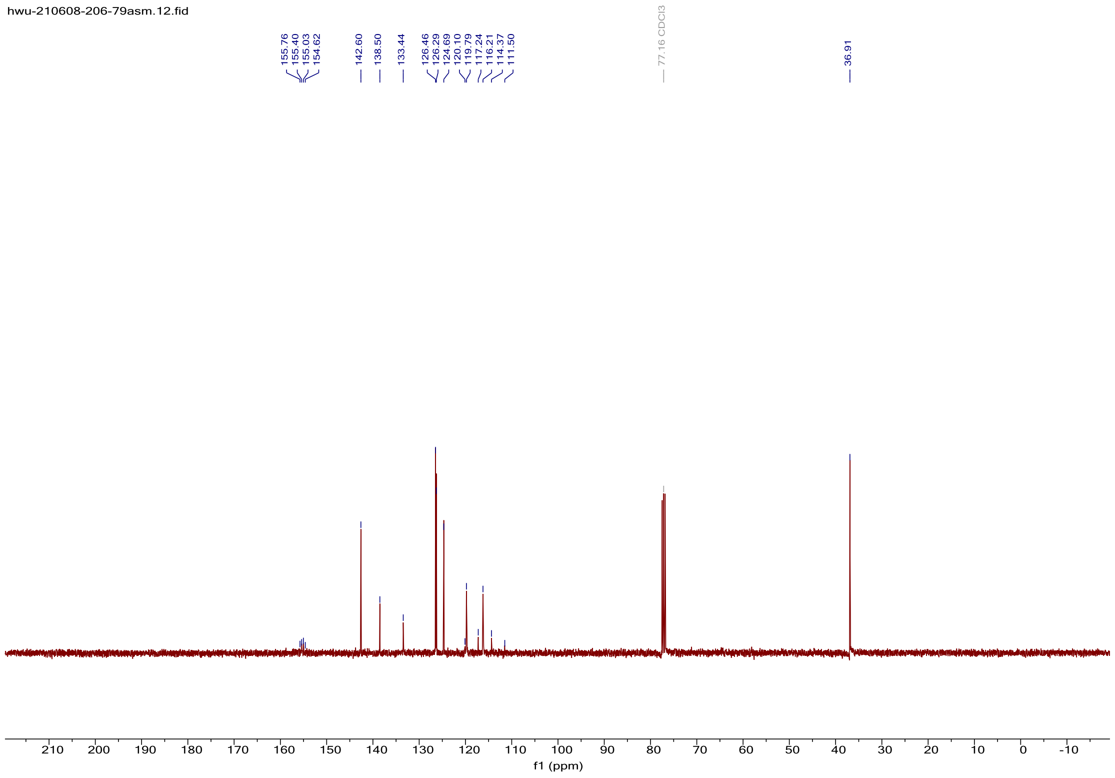
hwu-210608-206-79asm.12.fid
155.76 155.40 155.03 154.62 142.60 138.50 133.44 126.46 126.29 124.69 120.10 119.79 117.24 116.21 114.37 111.50
77.16 CDCl3
36.91
f1 (ppm)

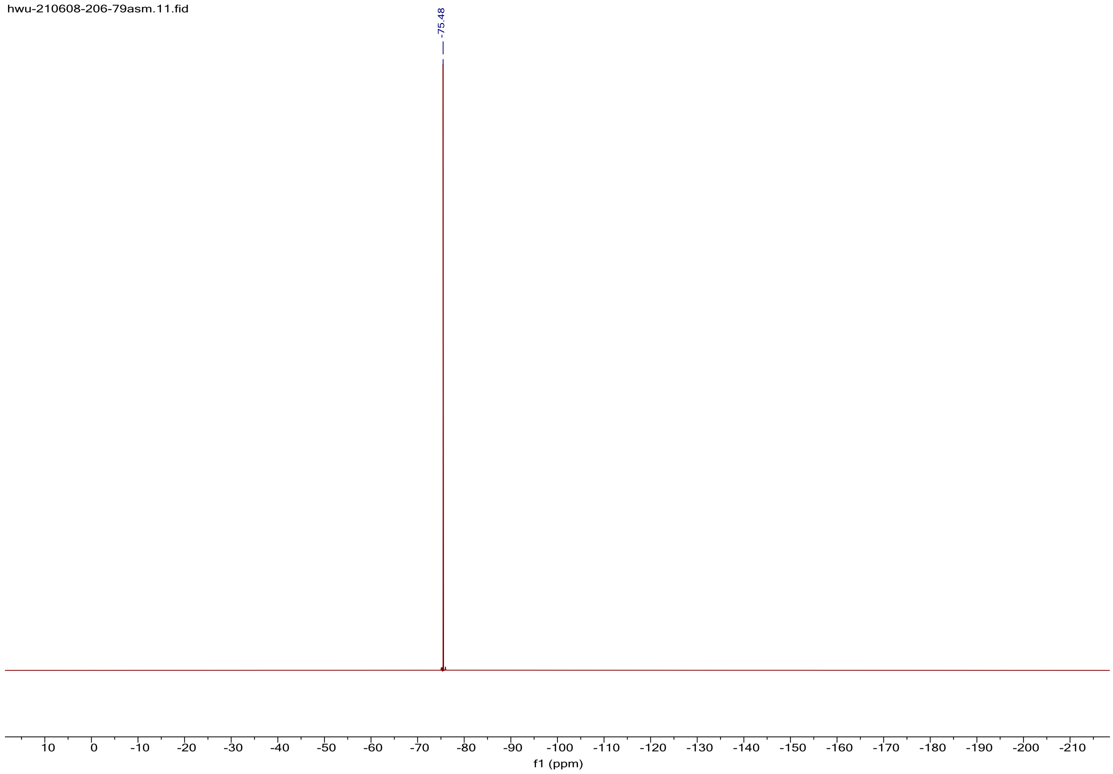
hwu-210608-206-79asm.11.fid
-75.48
10 0 -10 -20 -30 -40 -50 -60 -70 -80 -90 -100 -110 -120 -130 -140 -150 -160 -170 -180 -190 -200 -210
f1 (ppm)

hwu-210608-206-79dsm.10.fid

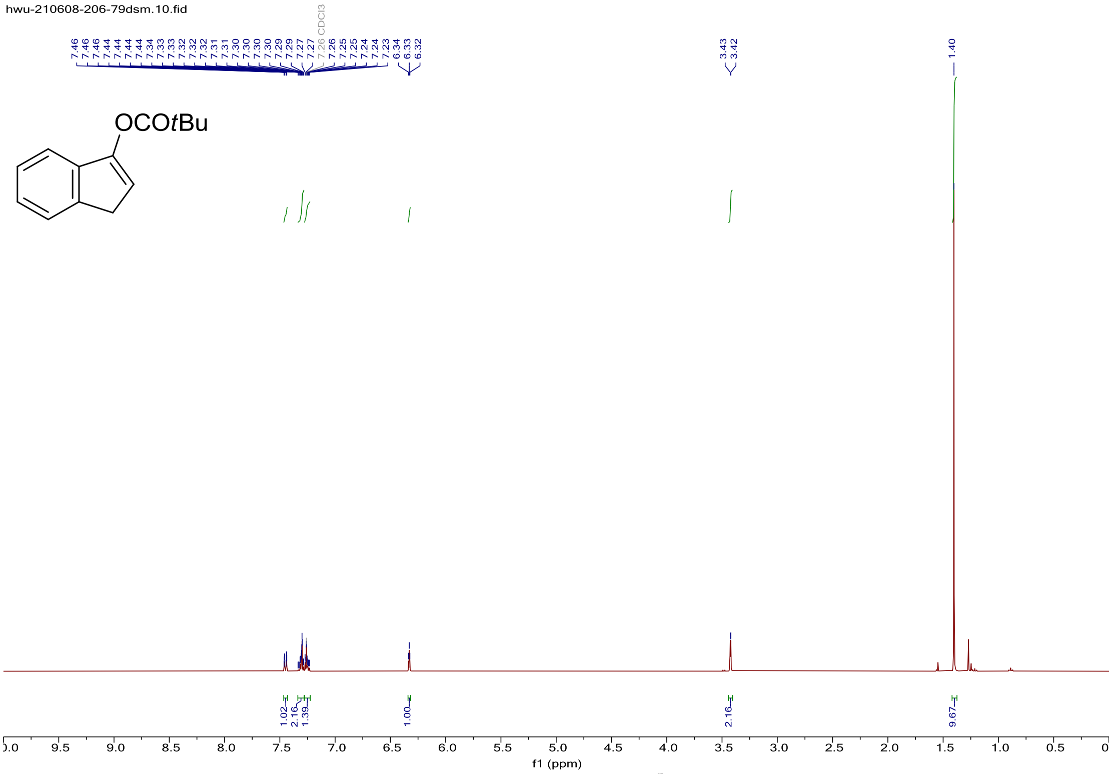


hwu-210608-206-79dsm.11.fid

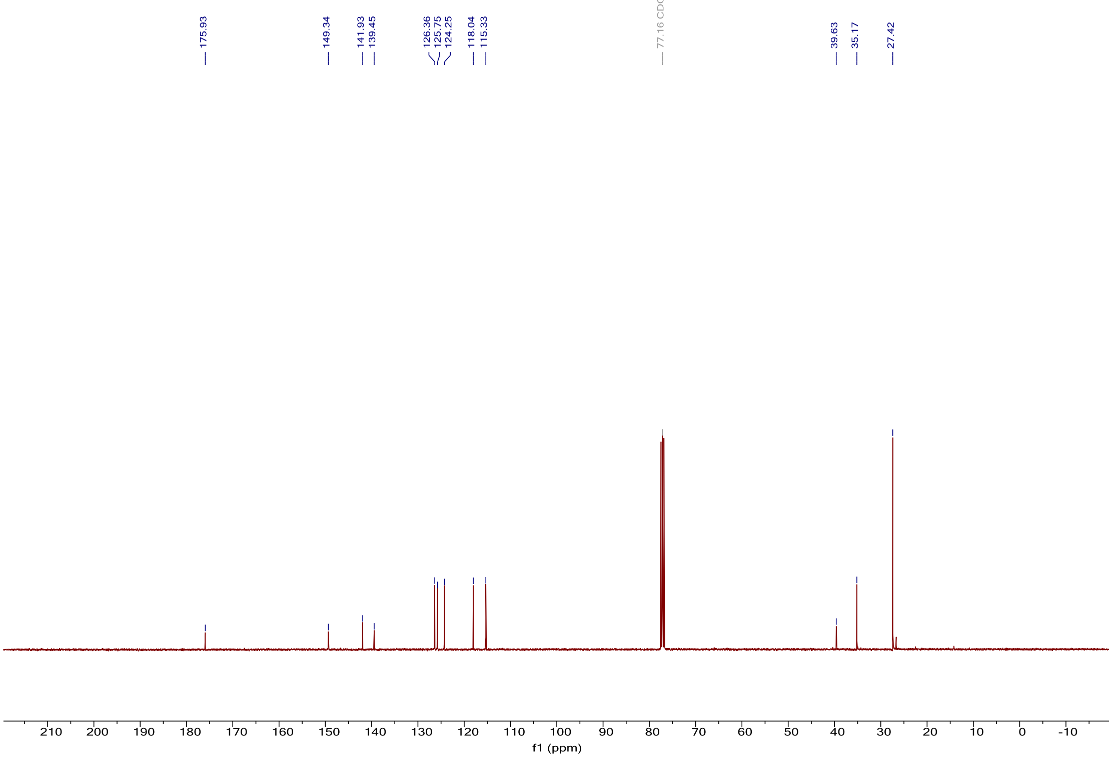

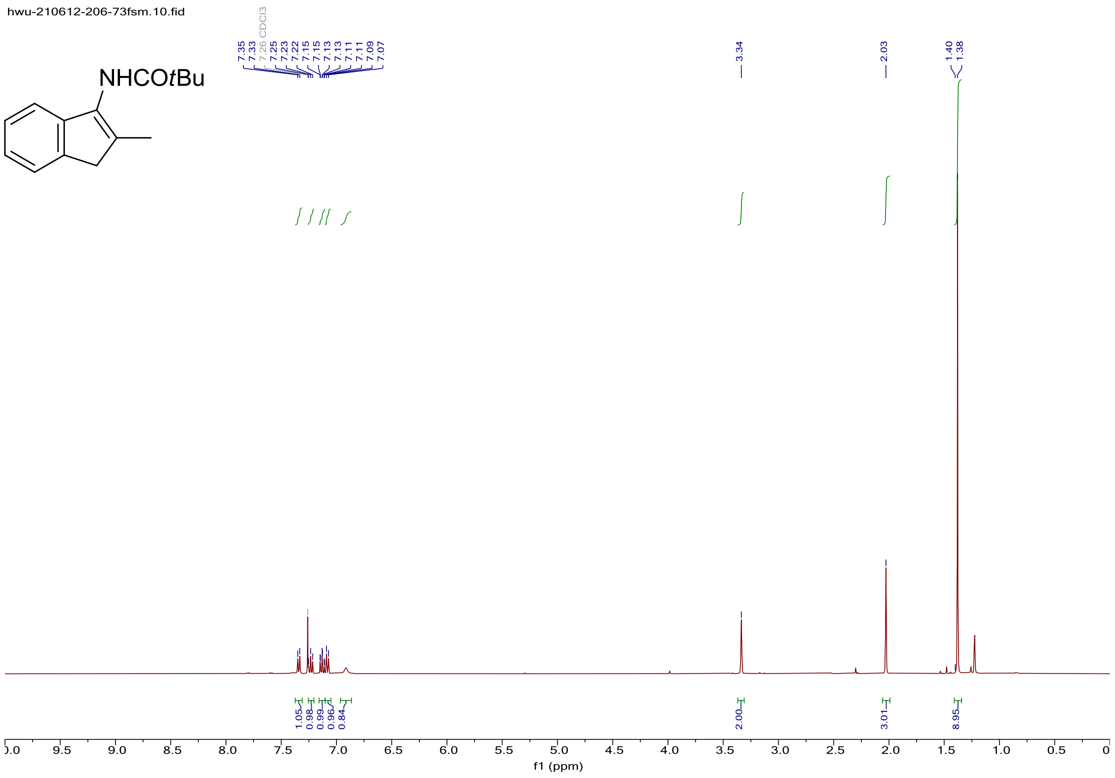
hwu-210612-206-73fsm.10.fid
NHCO*t*Bu
7.35
7.33
7.26 CDCl3
7.25
7.23
7.22
7.15
7.15
7.13
7.13
7.11
7.11
7.09
7.07
3.34
2.03
1.40
1.38
1.05
0.98
0.99
0.96
0.84
2.00
3.01
8.95
f1 (ppm)

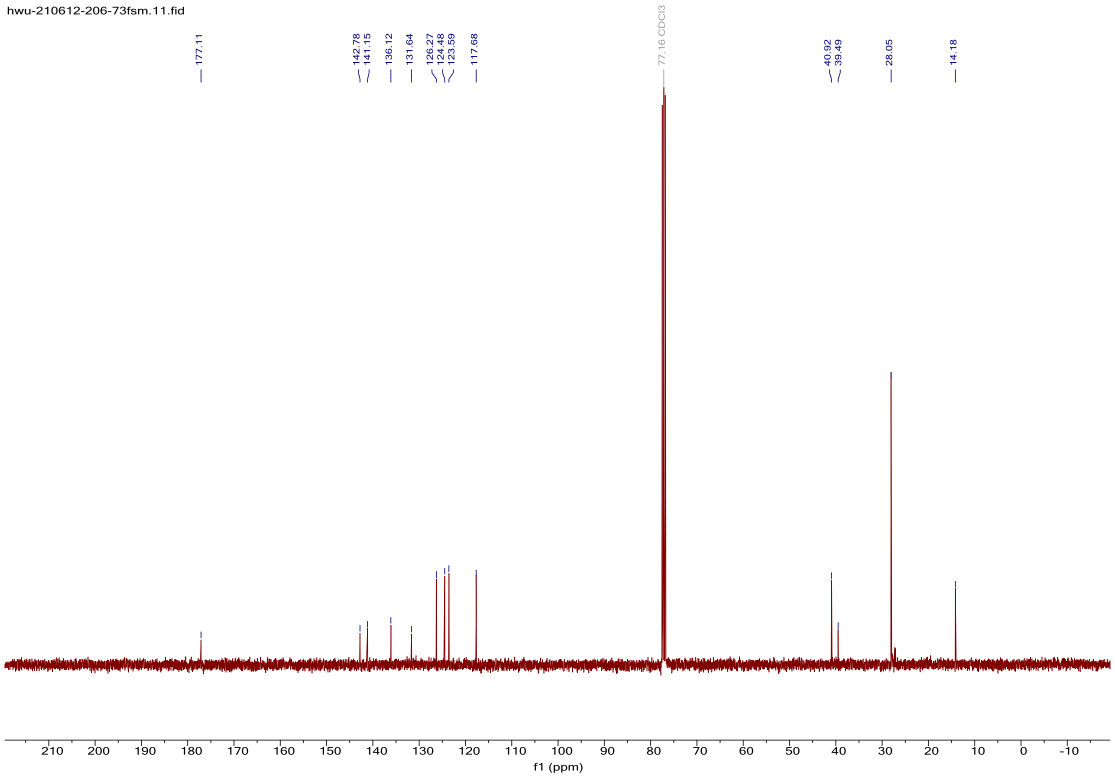
hwu-210612-206-73fsm.11.fid
177.11
142.78
141.15
136.12
131.64
126.27
124.48
123.59
117.68
77.16 CDCl3
40.92
39.49
28.05
14.18
f1 (ppm)

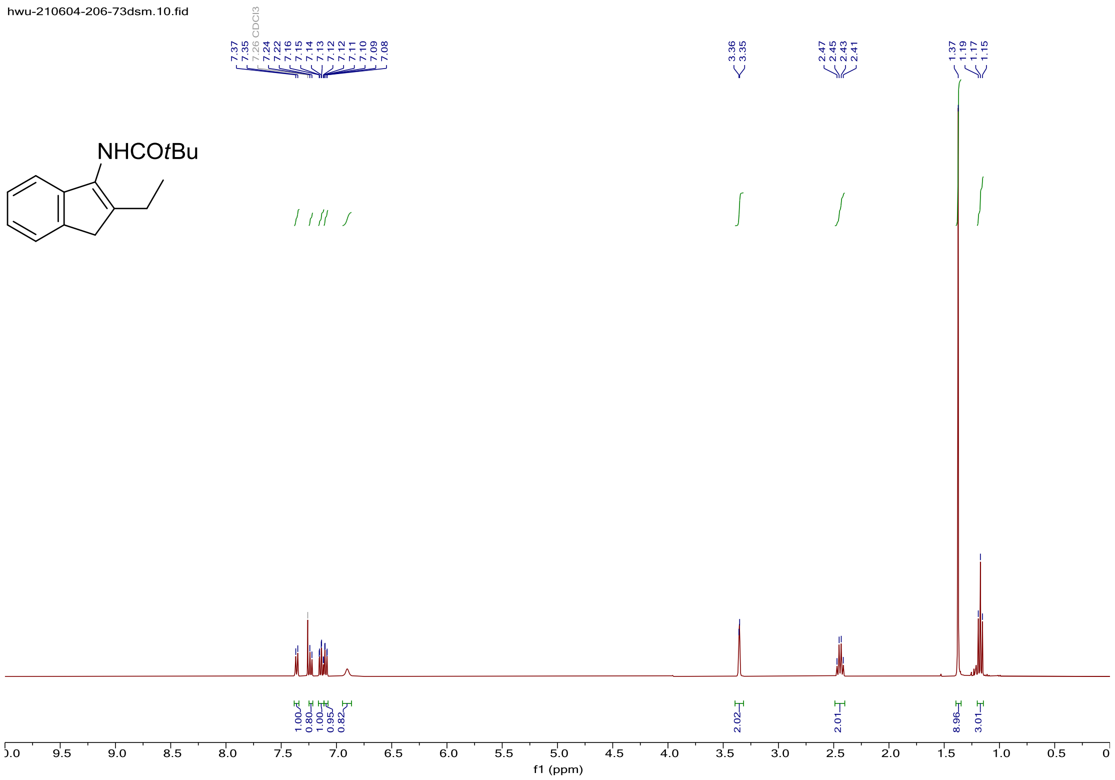
hwu-210604-206-73dsm.10.fid
7.37
7.35
7.26 CDCl3
7.24
7.22
7.16
7.15
7.14
7.13
7.12
7.12
7.11
7.10
7.09
7.08
3.36
3.35
2.47
2.45
2.43
2.41
1.37
1.19
1.17
1.15
NHCO*t*Bu
1.00
0.80
1.00
0.95
0.82
2.02
2.01
8.96
3.01
f1 (ppm)

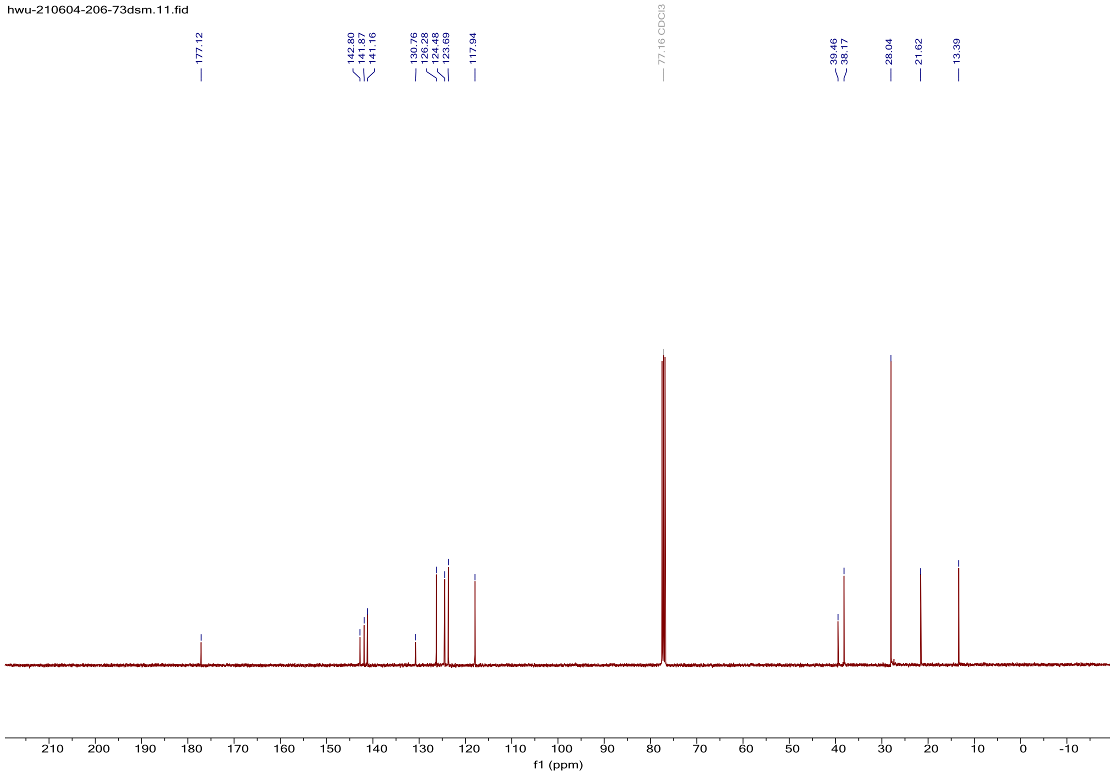
hwu-210604-206-73dsm.11.fid
177.12
142.80
141.87
141.16
130.76
126.28
124.48
123.69
117.94
77.16 CDCl3
39.46
38.17
28.04
21.62
13.39
f1 (ppm)

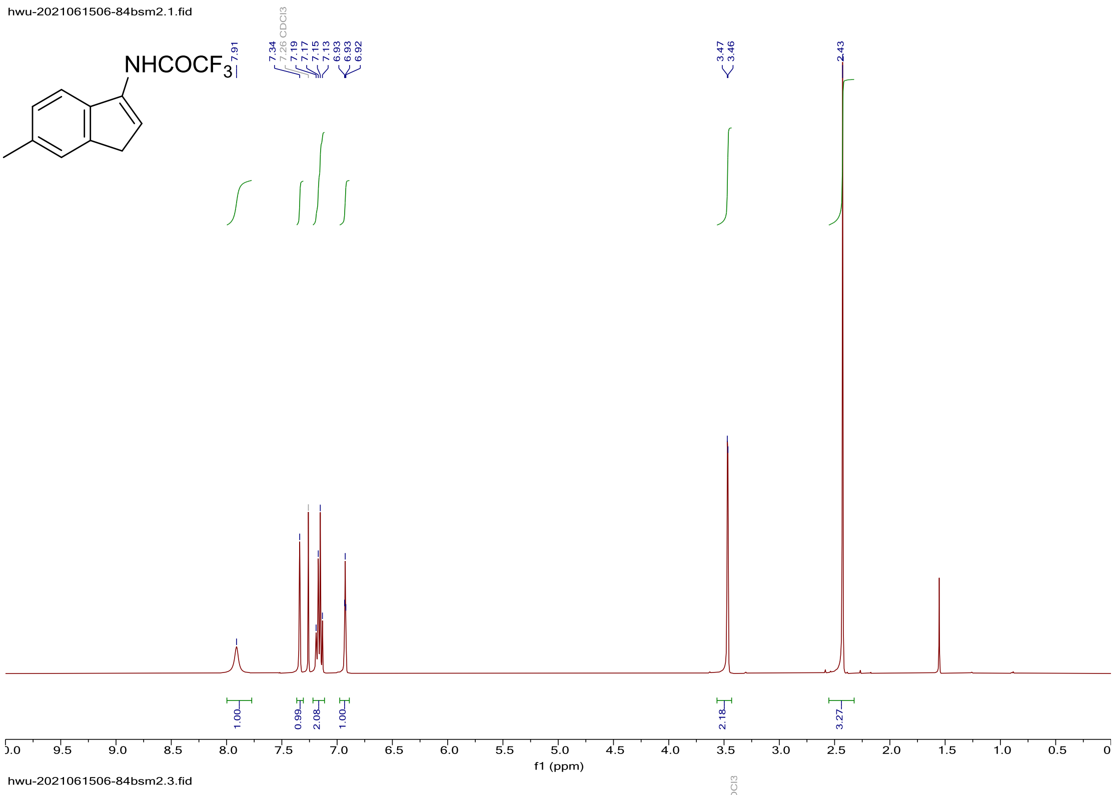

hwu-2021061506-84bsm2.1.fid
NHCOCF3
7.91
7.34
7.26 CDCl3
7.19
7.17
7.15
7.13
6.93
6.93
6.92
3.47
3.46
2.43
1.00
0.99
2.08
1.00
2.18
3.27
f1 (ppm)


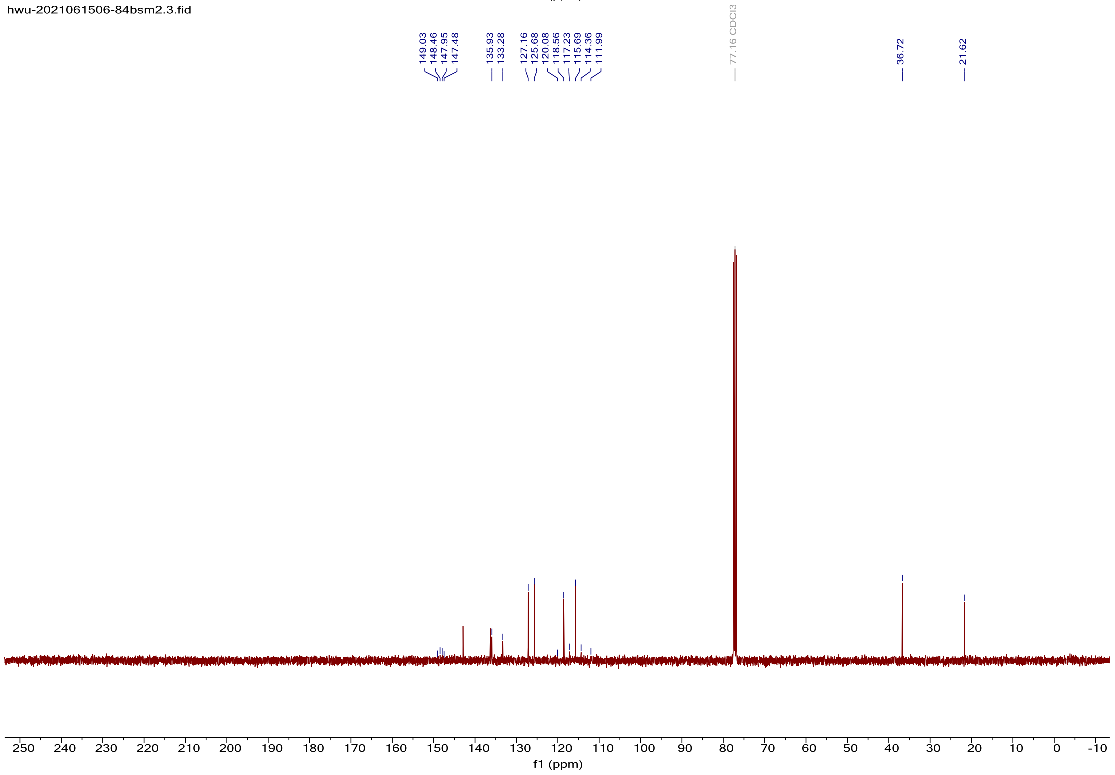

hwu-2021061506-84bsm2.3.fid
149.03
148.46
147.95
147.48
135.93
133.28
127.16
125.68
120.08
118.56
117.23
115.69
114.36
111.99
77.16 CDCl3
36.72
21.62
f1 (ppm)

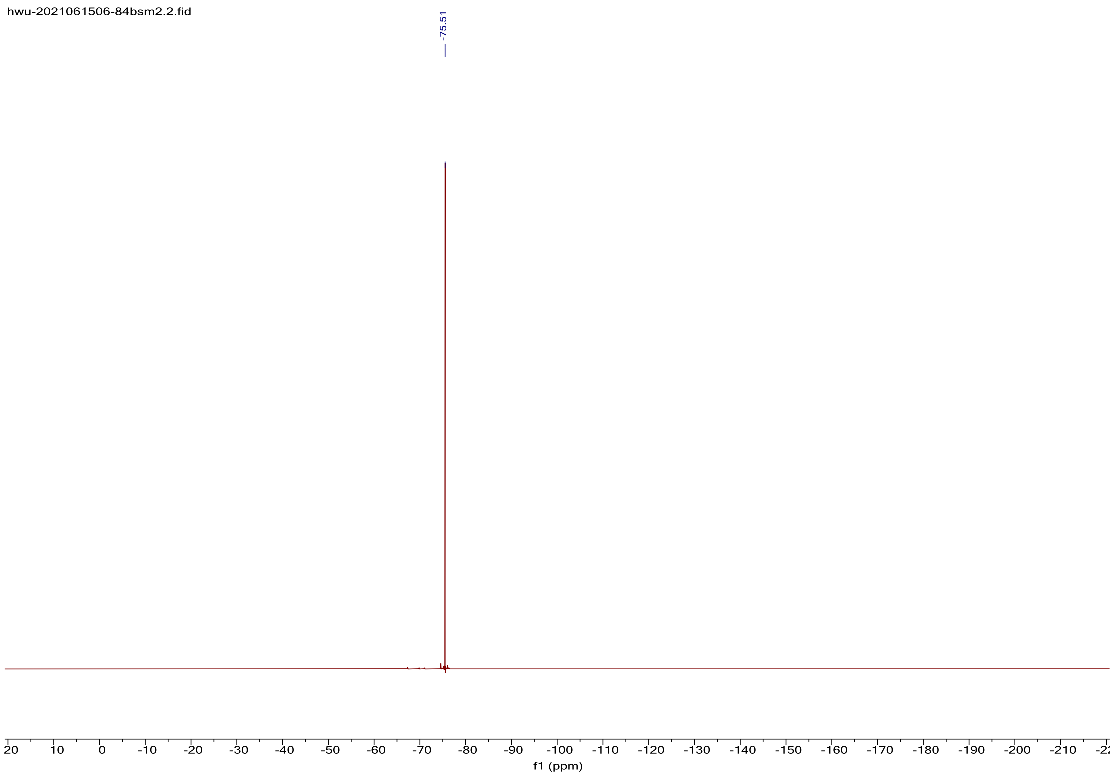
hwu-2021061506-84bsm2.2.fid
-75.51
20 10 0 -10 -20 -30 -40 -50 -60 -70 -80 -90 -100 -110 -120 -130 -140 -150 -160 -170 -180 -190 -200 -210
f1 (ppm)

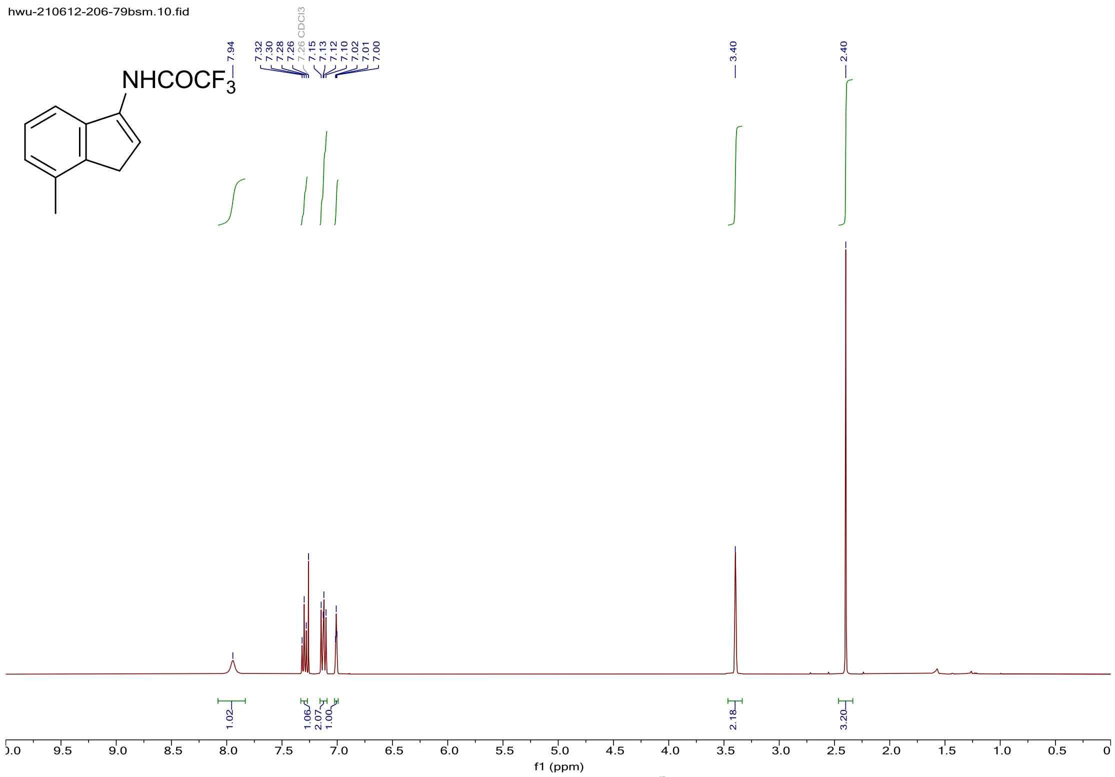
hwu-210612-206-79bsm.10.fid
NHCOCF3
7.94
7.32
7.30
7.28
7.26
7.26 CDCl3
7.15
7.13
7.12
7.10
7.02
7.01
7.00
3.40
2.40
1.02
1.06
2.07
1.00
2.18
3.20
f1 (ppm)

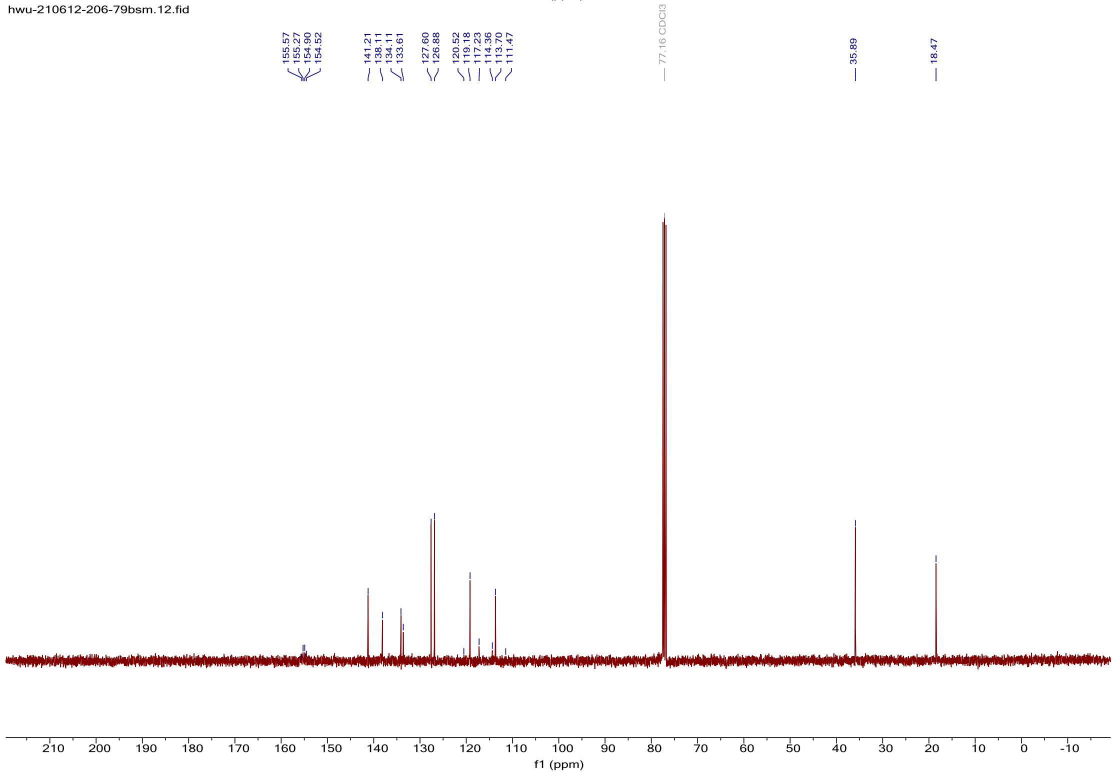
hwu-210612-206-79bsm.12.fid
155.57
155.27
154.90
154.52
141.21
138.11
134.11
133.61
127.60
126.88
120.52
119.18
117.23
114.36
113.70
111.47
77.16 CDCl3
35.89
18.47
f1 (ppm)

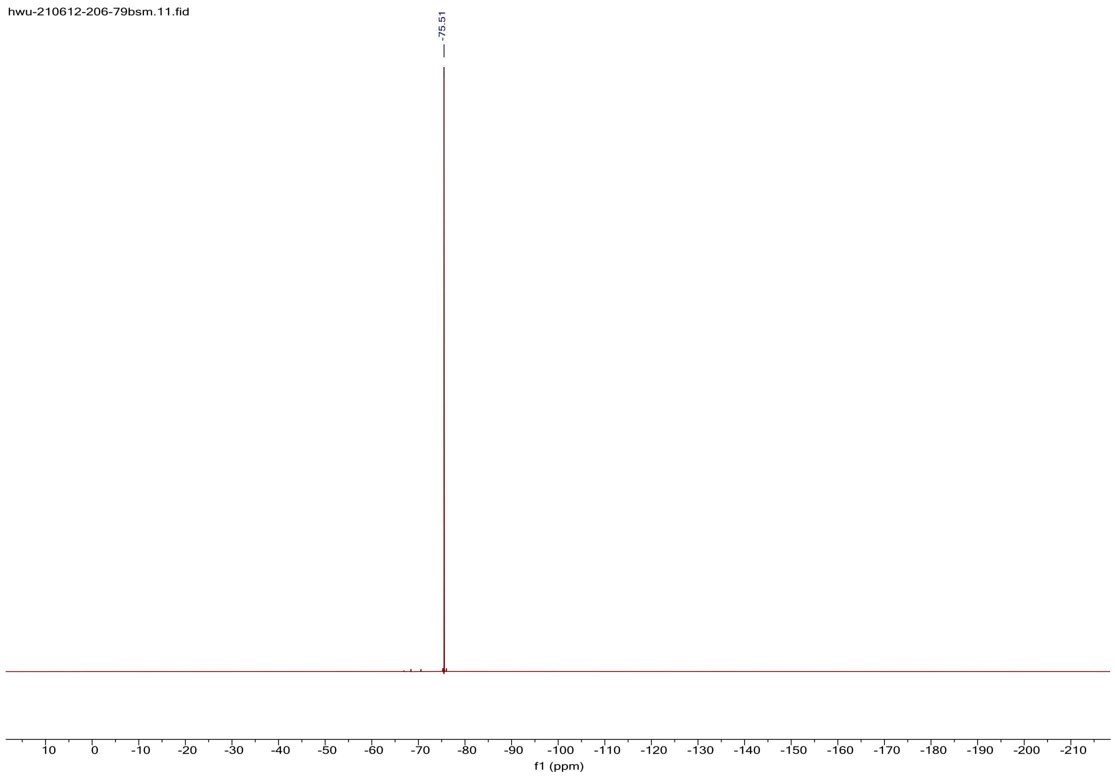
hwu-210612-206-79bsm.11.fid
-75.51
10 0 -10 -20 -30 -40 -50 -60 -70 -80 -90 -100 -110 -120 -130 -140 -150 -160 -170 -180 -190 -200 -210
f1 (ppm)

hwu-2021061606-84csm.1.fid

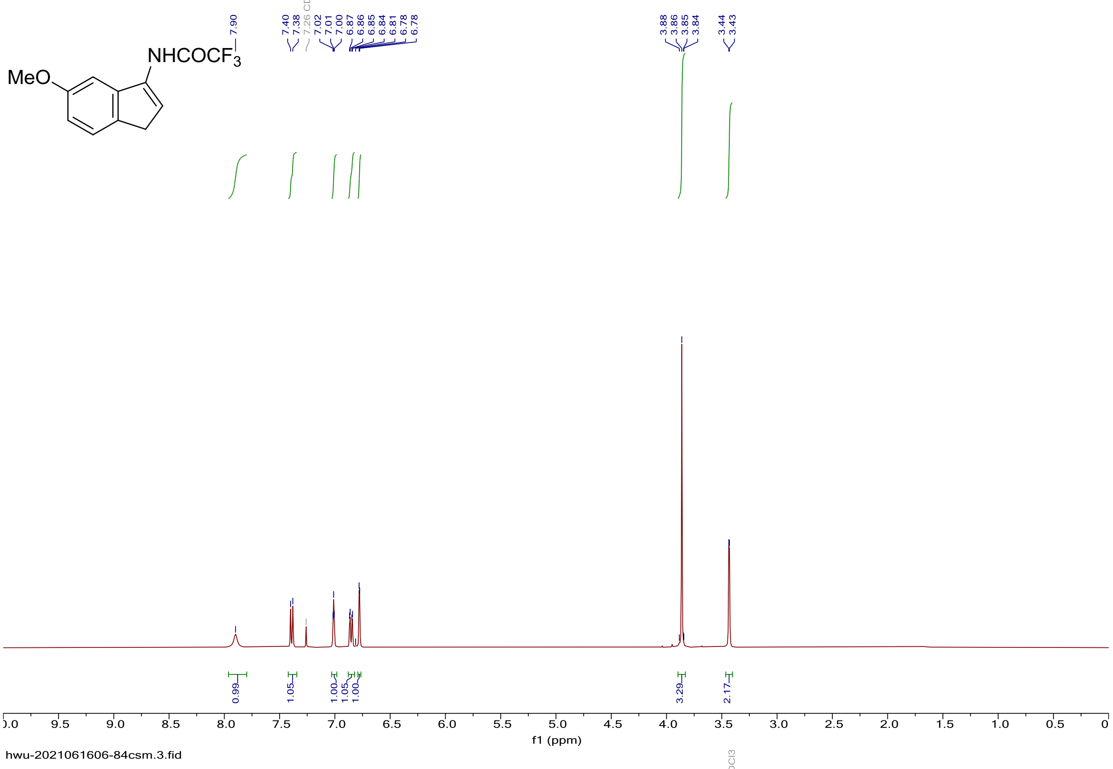

hwu-2021061606-84csm.3.fid

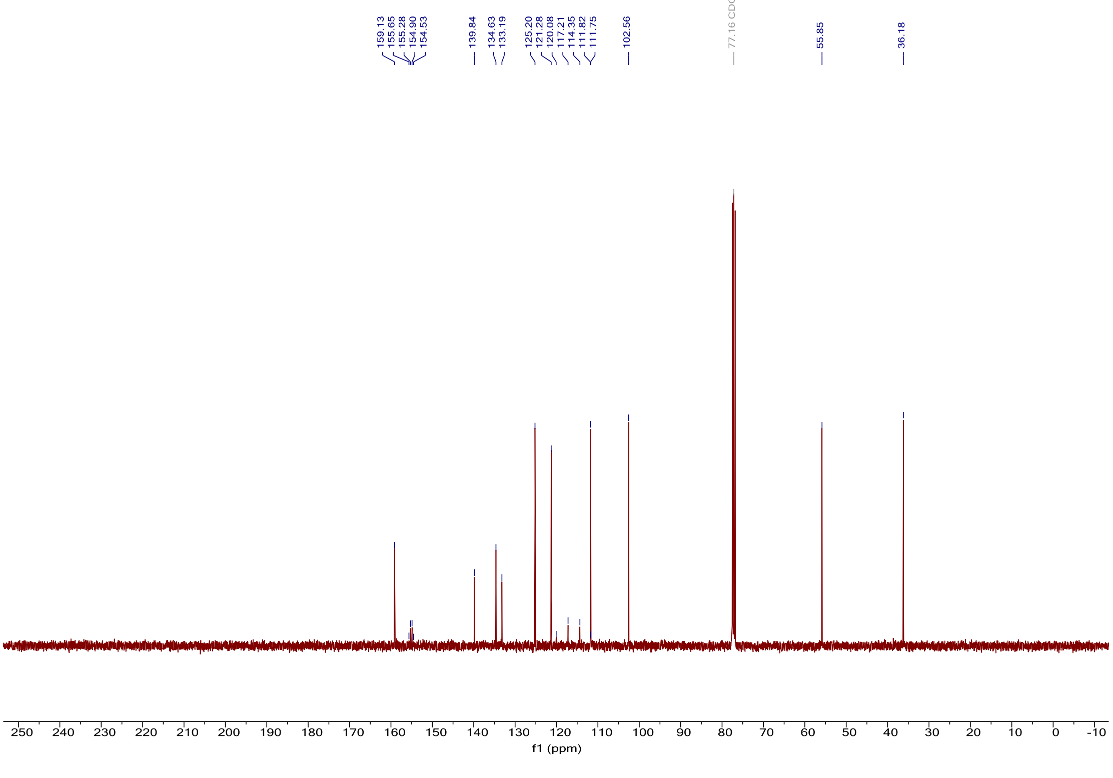

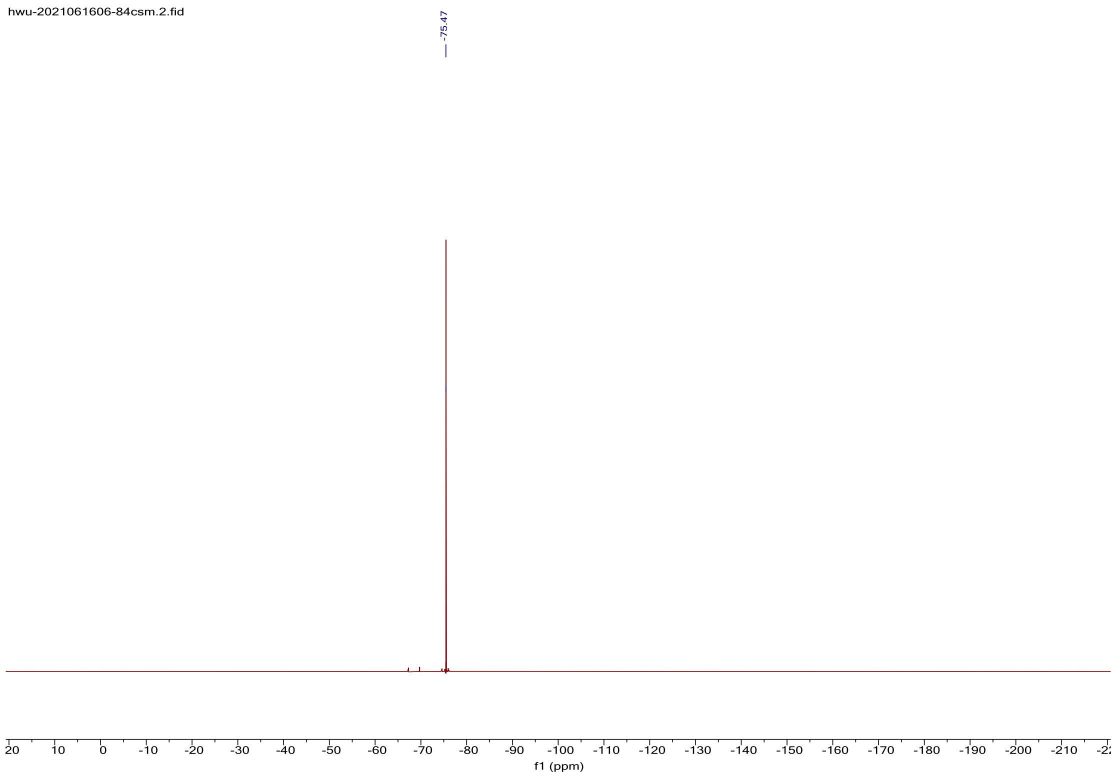
hwu-2021061606-84csm.2.fid
-75.47
20
10
0
-10
-20
-30
-40
-50
-60
-70
-80
-90
-100
-110
-120
-130
-140
-150
-160
-170
-180
-190
-200
-210
f1 (ppm)

hwu-210619-206-84asm.10.fid

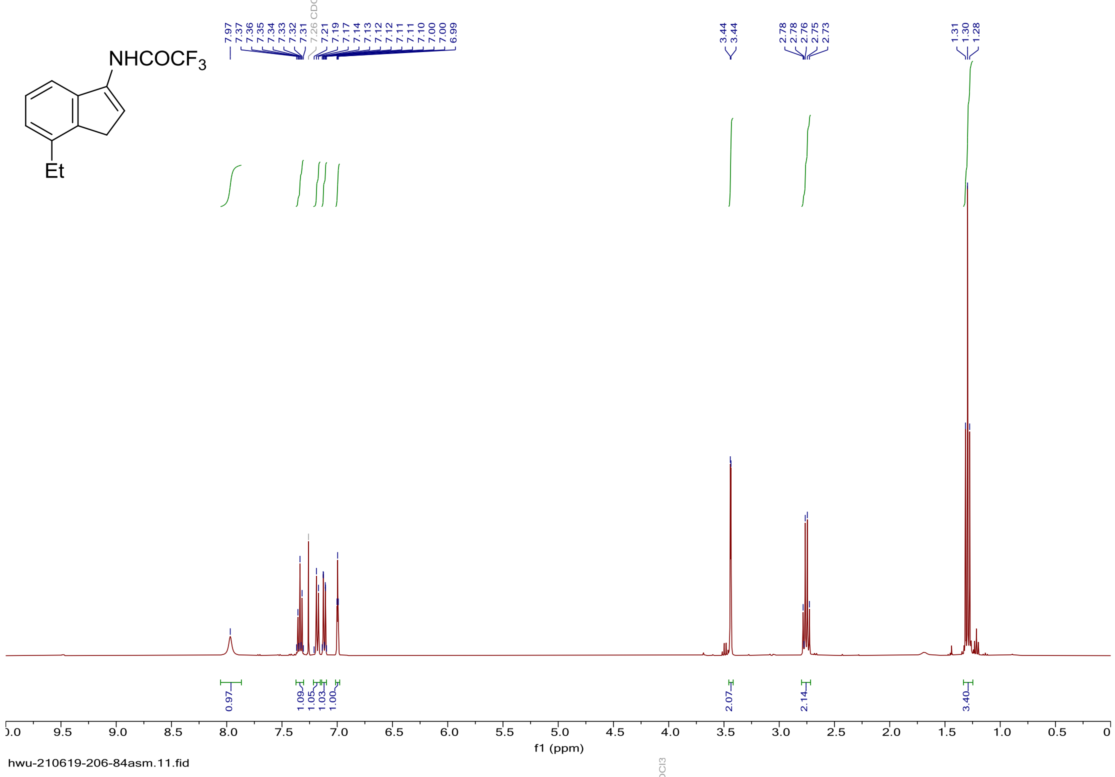


hwu-210619-206-84asm.11.fid

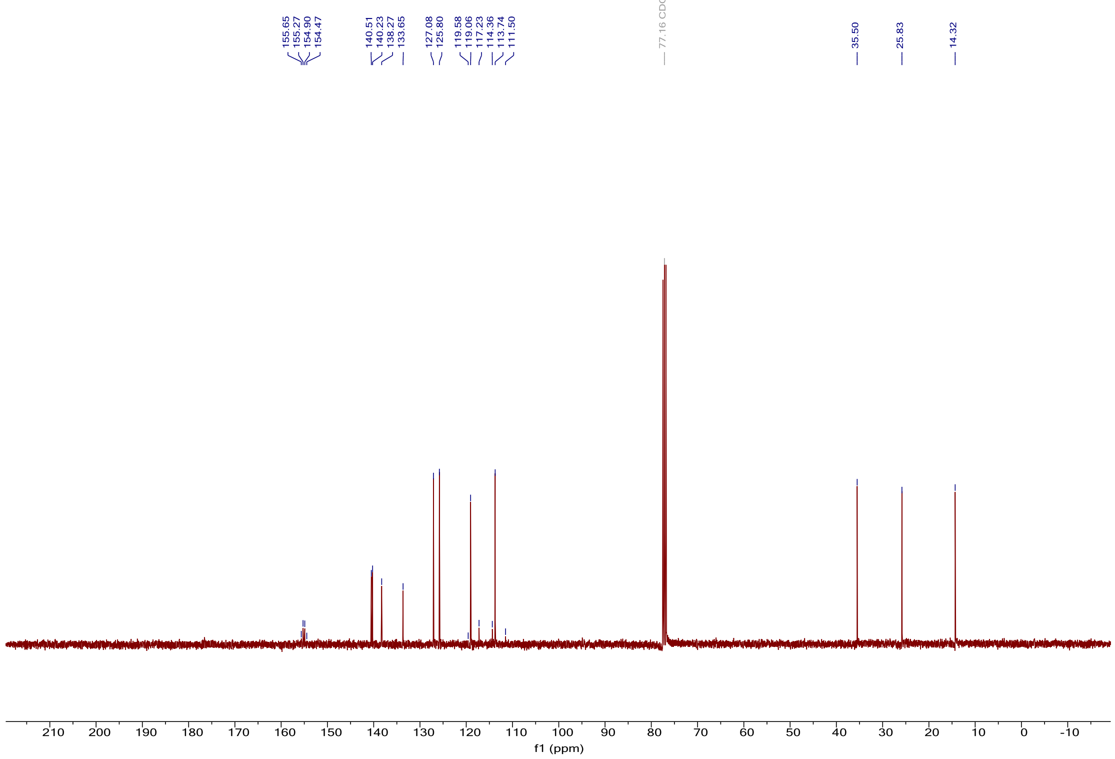

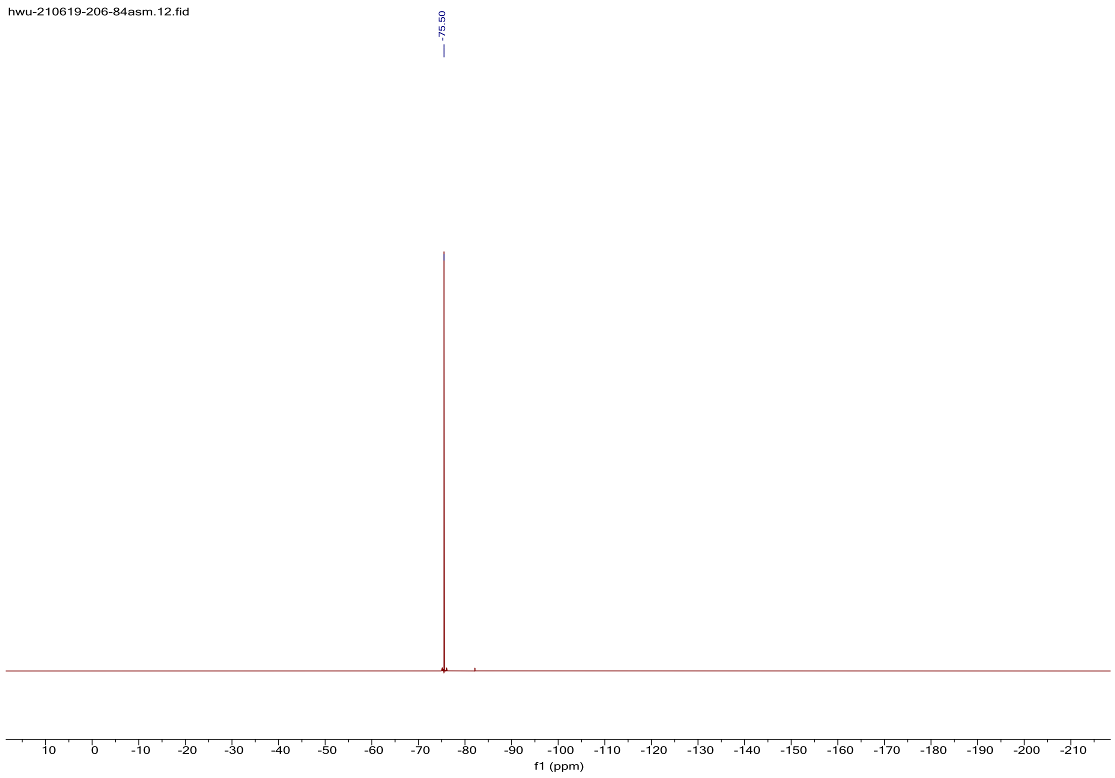
hwu-210619-206-84asm.12.fid
-75.50
10 0 -10 -20 -30 -40 -50 -60 -70 -80 -90 -100 -110 -120 -130 -140 -150 -160 -170 -180 -190 -200 -210
f1 (ppm)

yanji-20210612-9-sm-9-DMSO.1.fid

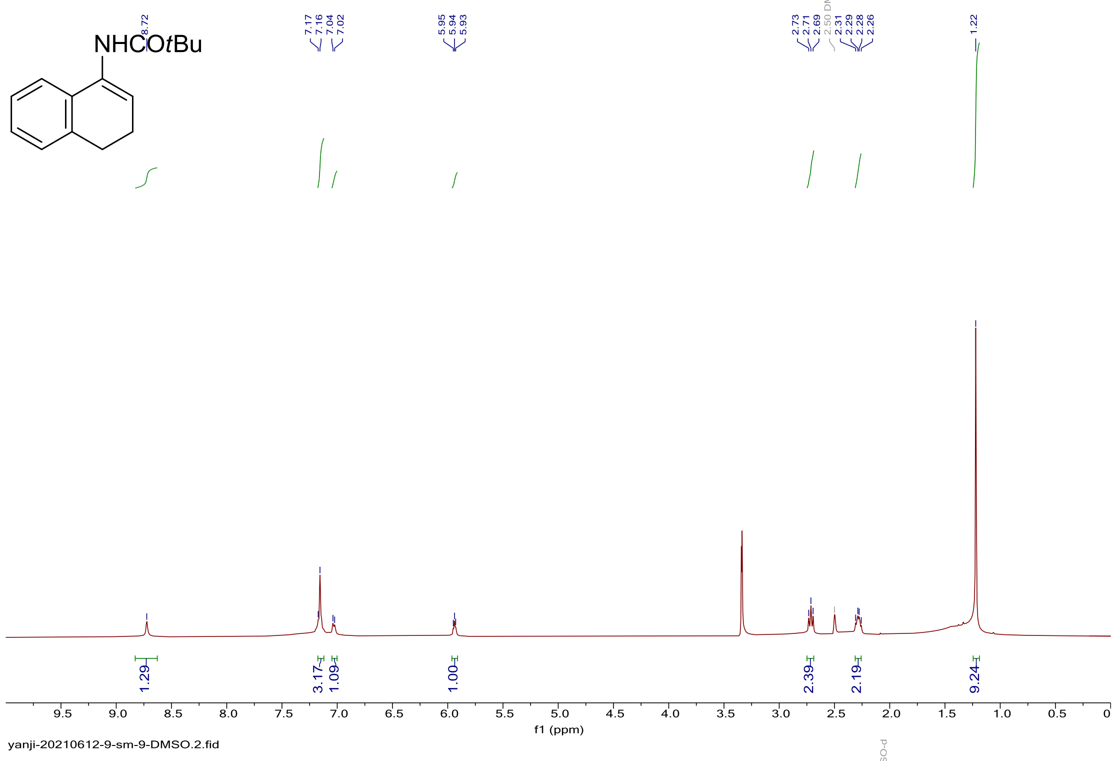


yanji-20210612-9-sm-9-DMSO.2.fid

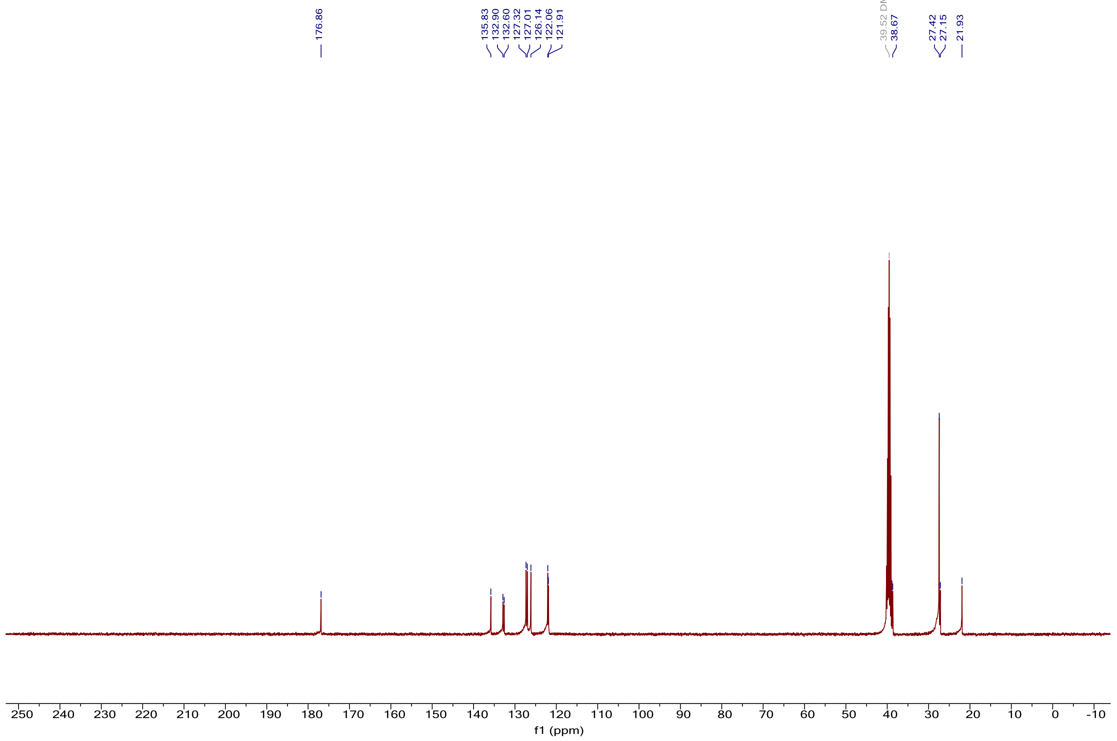

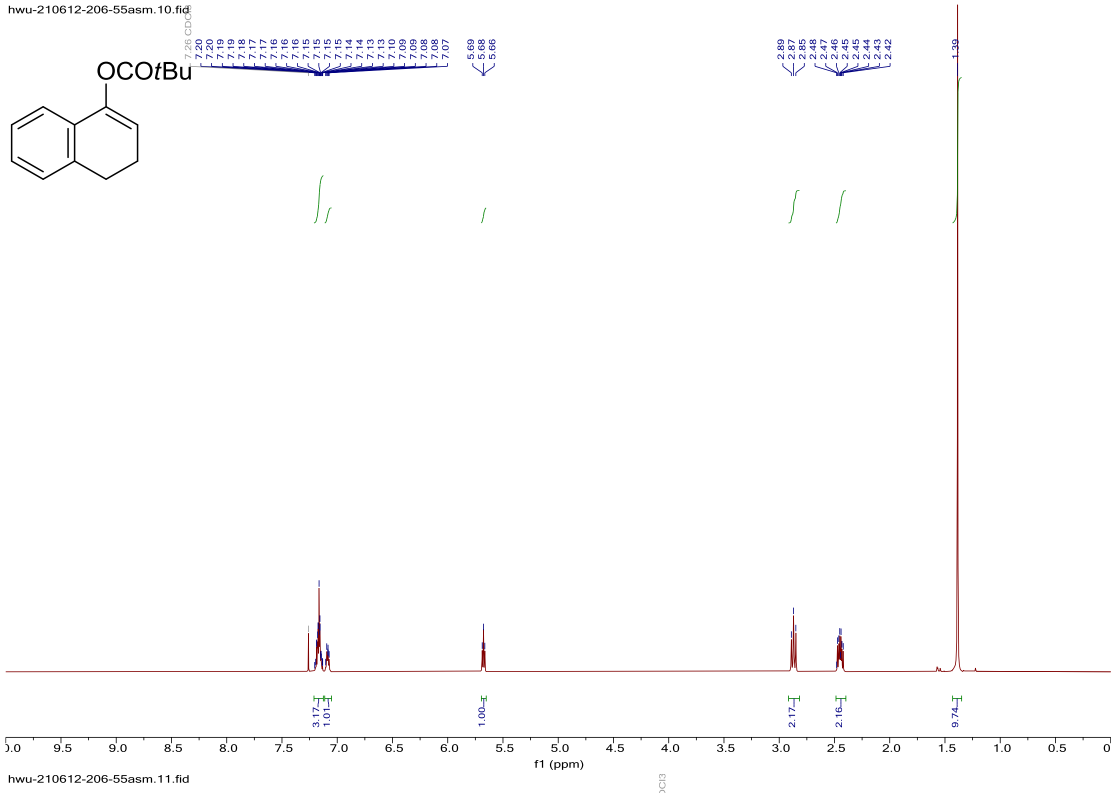
hwu-210612-206-55asm.10.fid
OCOtBu
7.26 CDCl3
7.20 7.20 7.19 7.19 7.18 7.17 7.17 7.16 7.16 7.16 7.15 7.15 7.15 7.15 7.14 7.14 7.13 7.13 7.10 7.09 7.09 7.08 7.08 7.07
5.69 5.68 5.66
2.89 2.87 2.85 2.48 2.47 2.46 2.45 2.45 2.44 2.43 2.42
1.39
3.17 1.01 1.00 2.17 2.16 9.74
f1 (ppm)

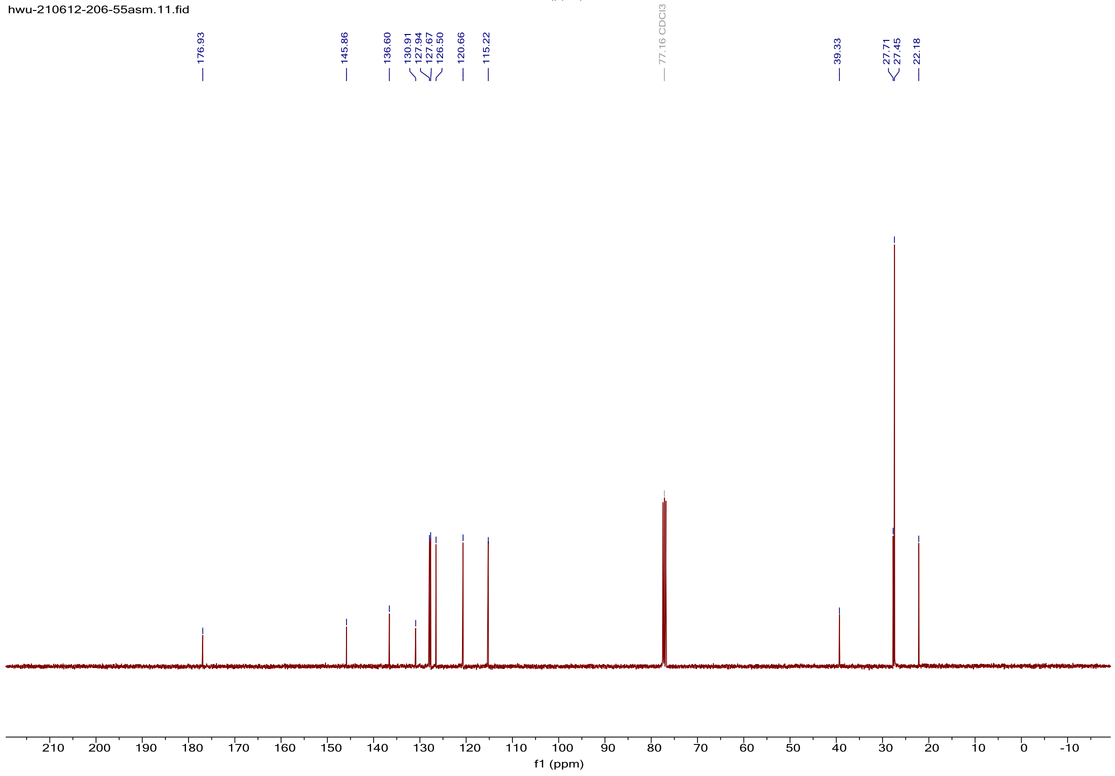
hwu-210612-206-55asm.11.fid
176.93
145.86
136.60
130.91 127.94 127.67 126.50
120.66
115.22
77.16 CDCl3
39.33
27.71 27.45
22.18
f1 (ppm)

hwu-210704-206-88dsm.10.fid

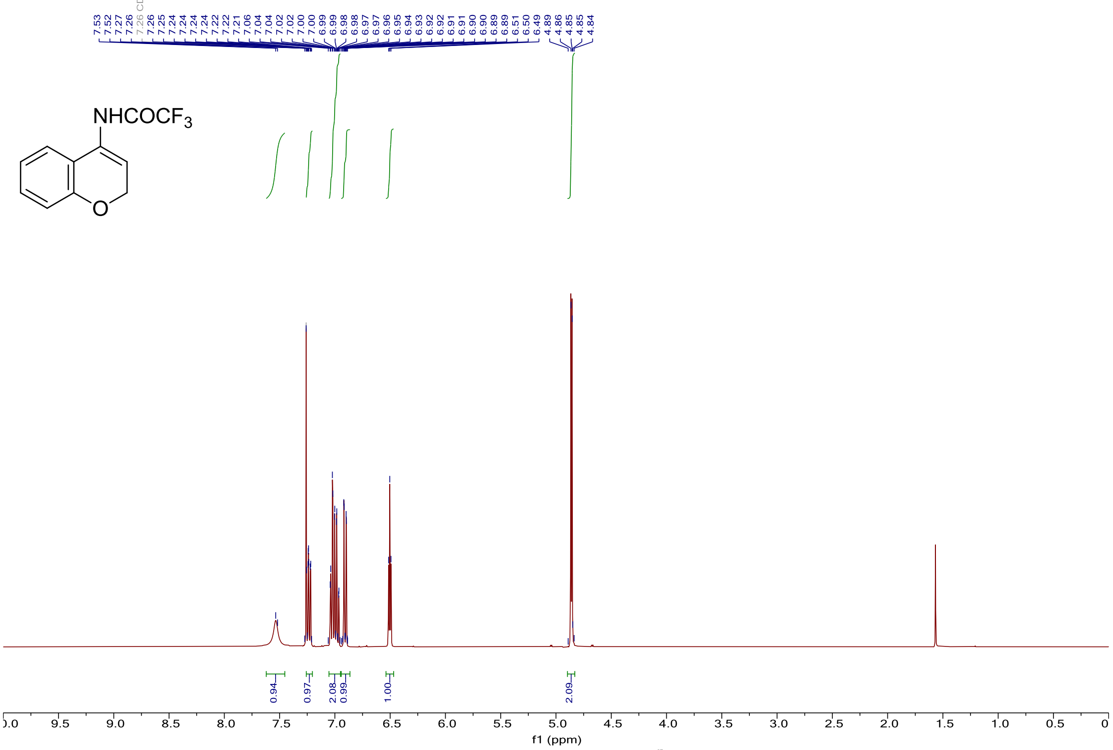


hwu-210704-206-88dsm.12.fid

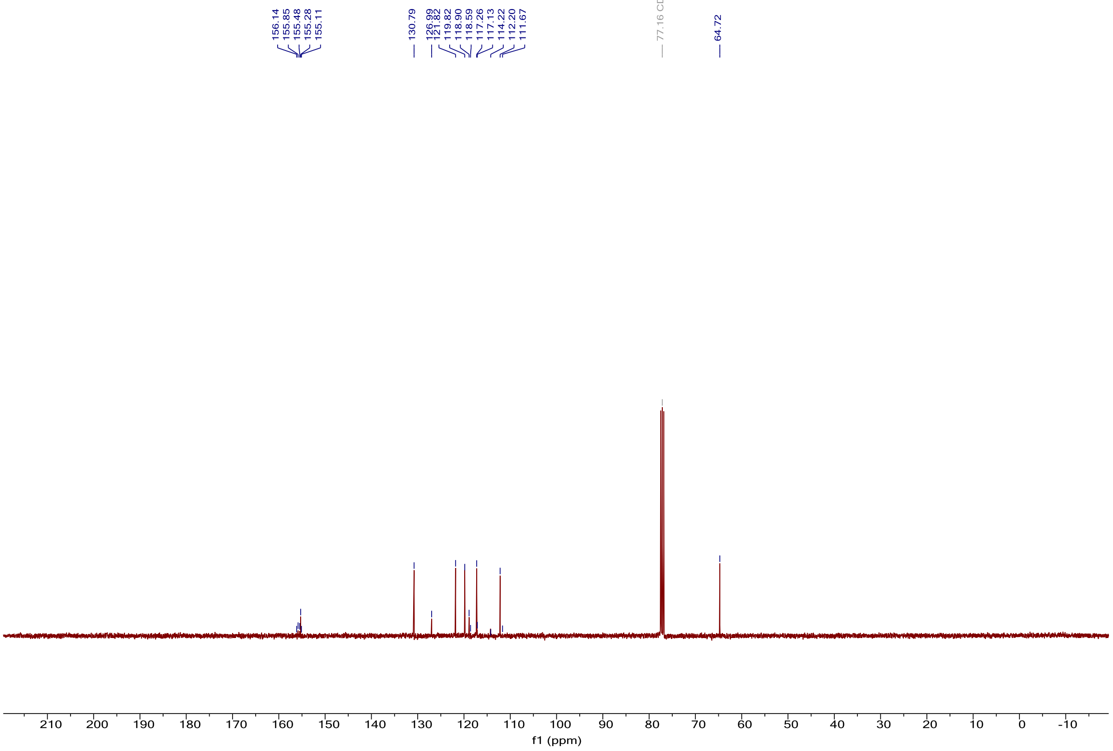

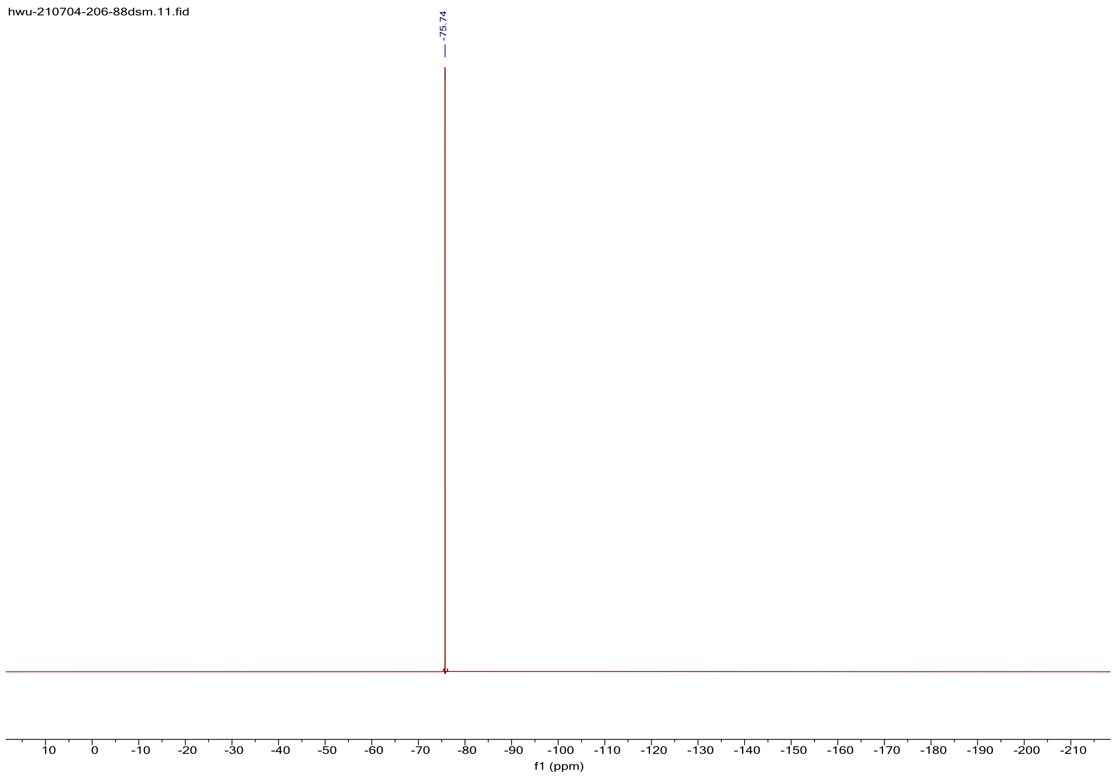
hwu-210704-206-88dsm.11.fid
-75.74
10 0 -10 -20 -30 -40 -50 -60 -70 -80 -90 -100 -110 -120 -130 -140 -150 -160 -170 -180 -190 -200 -210
f1 (ppm)

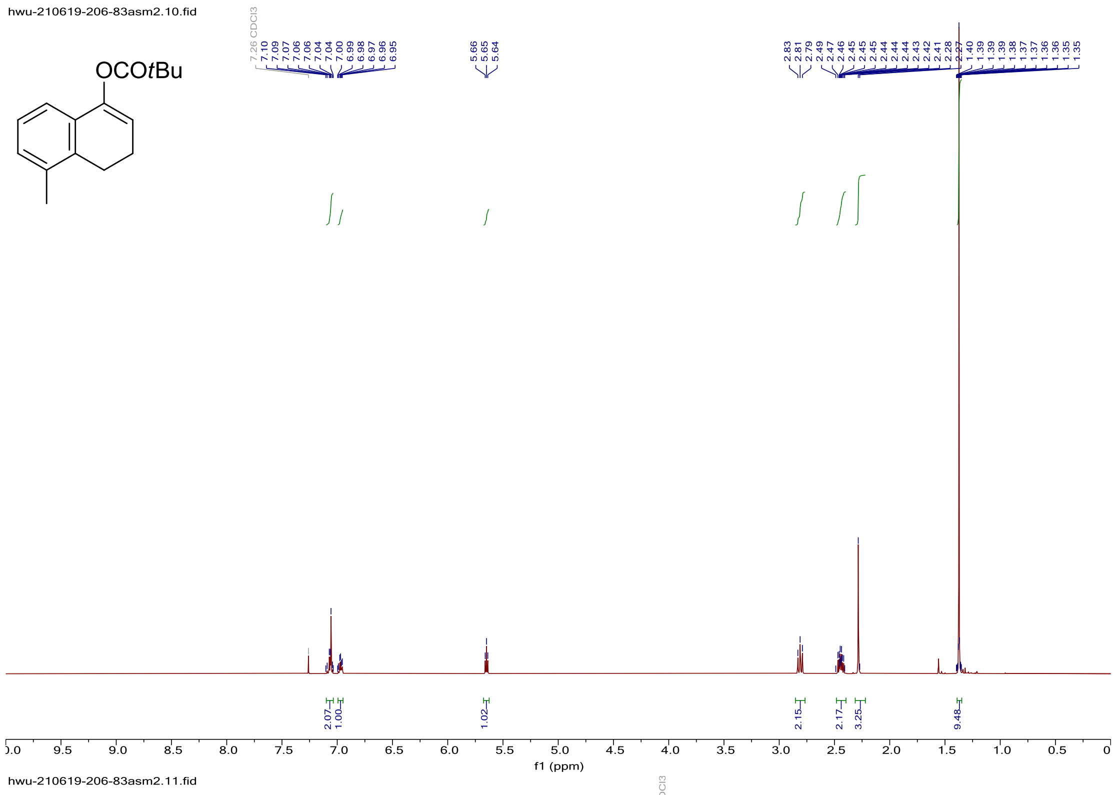
hwu-210619-206-83asm2.10.fid
OCOtBu
7.26 CDCl3
7.10 7.09 7.07 7.06 7.06 7.04 7.04 7.00 6.99 6.98 6.97 6.96 6.95
5.66 5.65 5.64
2.83 2.81 2.79 2.49 2.47 2.46 2.45 2.45 2.44 2.44 2.43 2.42 2.41 2.28 2.27 1.40 1.39 1.39 1.38 1.37 1.37 1.36 1.35 1.35
2.07 1.00 1.02 2.15 2.17 3.25 9.48
f1 (ppm)

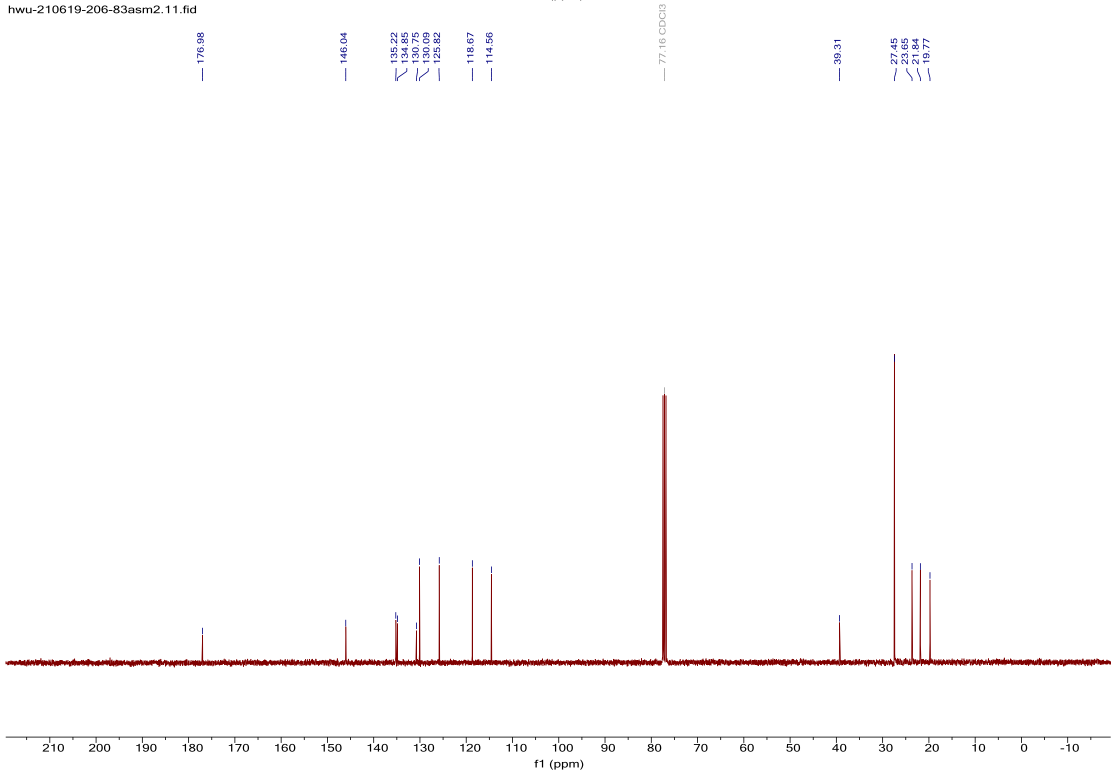
hwu-210619-206-83asm2.11.fid
176.98
146.04
135.22 134.85 130.75 130.09 125.82
118.67 114.56
77.16 CDCl3
39.31
27.45 23.65 21.84 19.77
f1 (ppm)

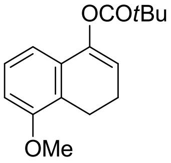
OCOtBu
OMe

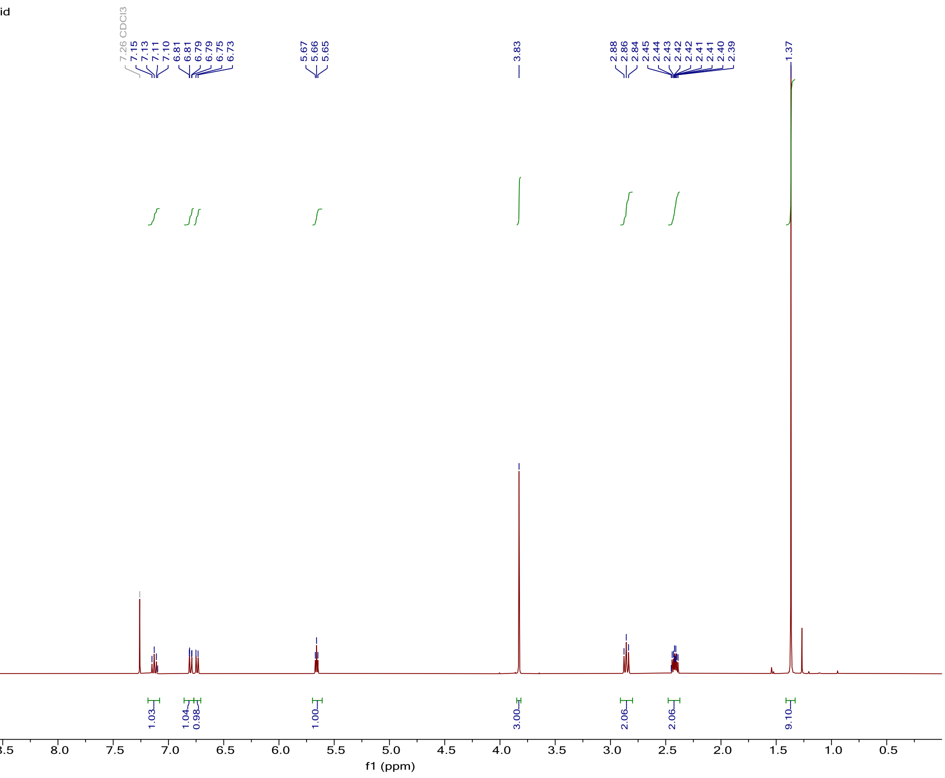
hwu-210508-206-54ap.10.fid
7.26 CDCl3
7.15
7.13
7.11
7.10
6.81
6.81
6.79
6.75
6.73
5.67
5.66
5.65
3.83
2.88
2.86
2.84
2.45
2.44
2.43
2.42
2.42
2.41
2.40
2.39
1.37
1.03
1.04
0.98
1.00
3.00
2.06
2.06
9.10
f1 (ppm)

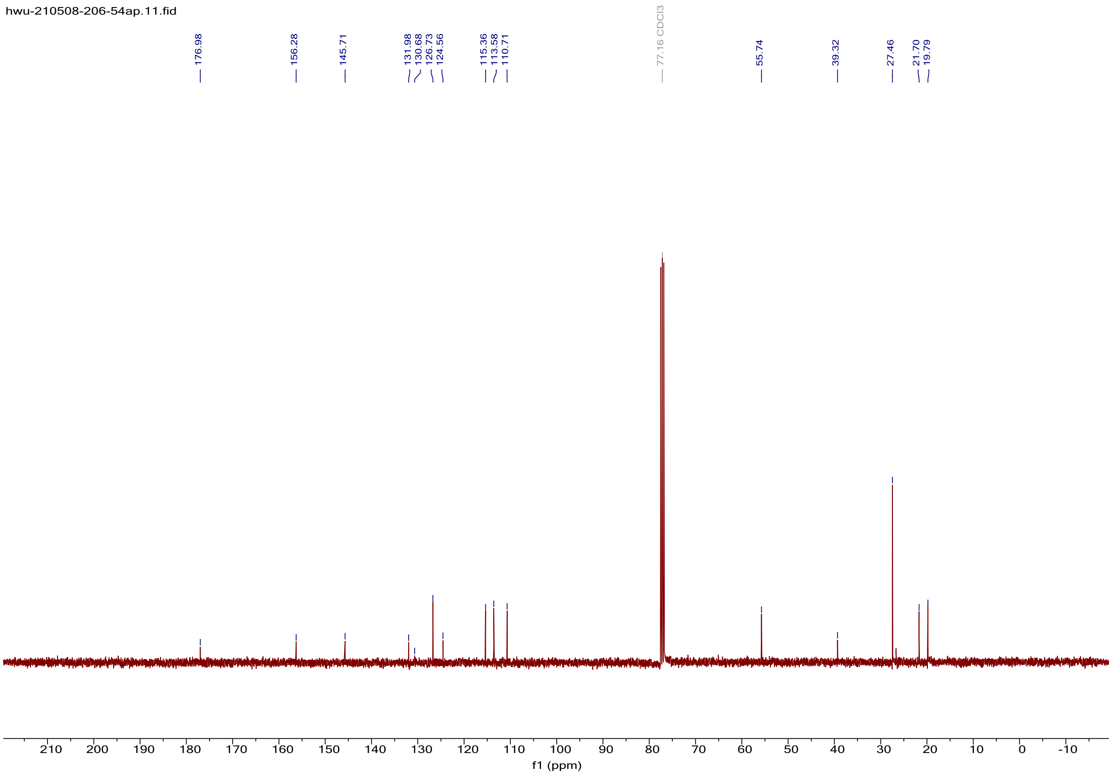
hwu-210508-206-54ap.11.fid
176.98
156.28
145.71
131.98
130.68
126.73
124.56
115.36
113.58
110.71
77.16 CDCl3
55.74
39.32
27.46
21.70
19.79
f1 (ppm)

hwu-210619-206-83bsm2.10.fid

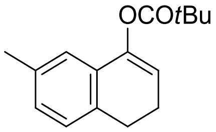

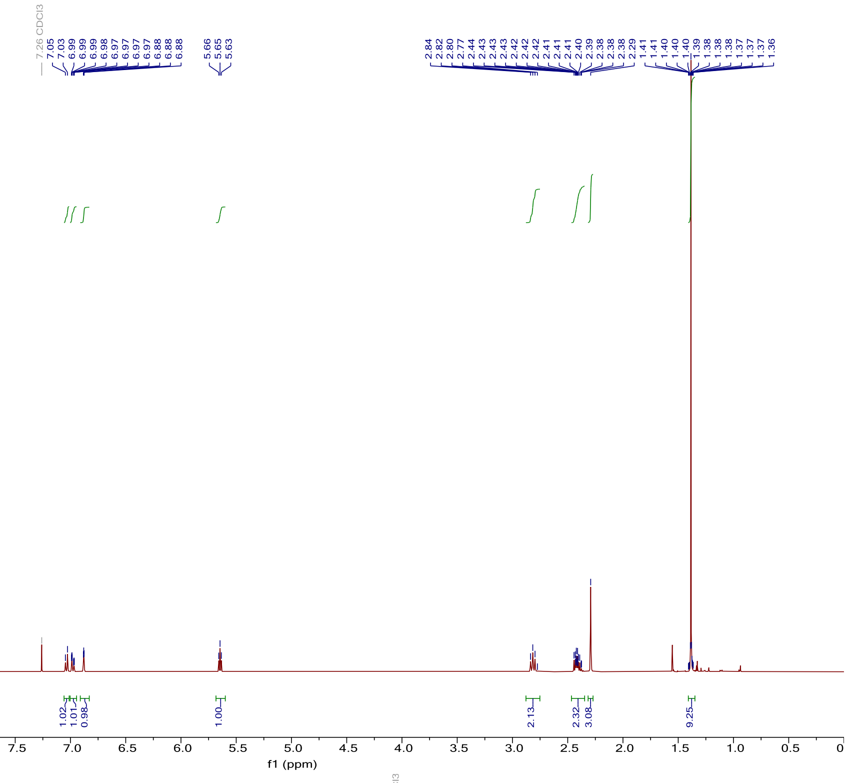

hwu-210619-206-83bsm2.11.fid

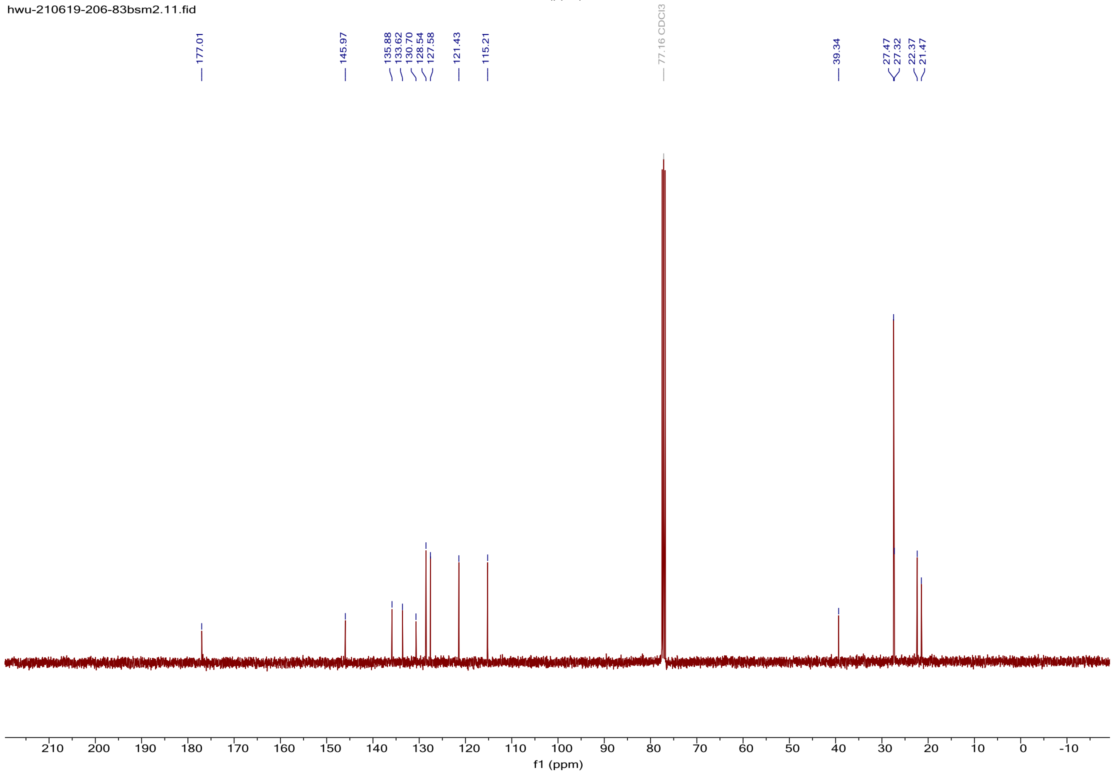

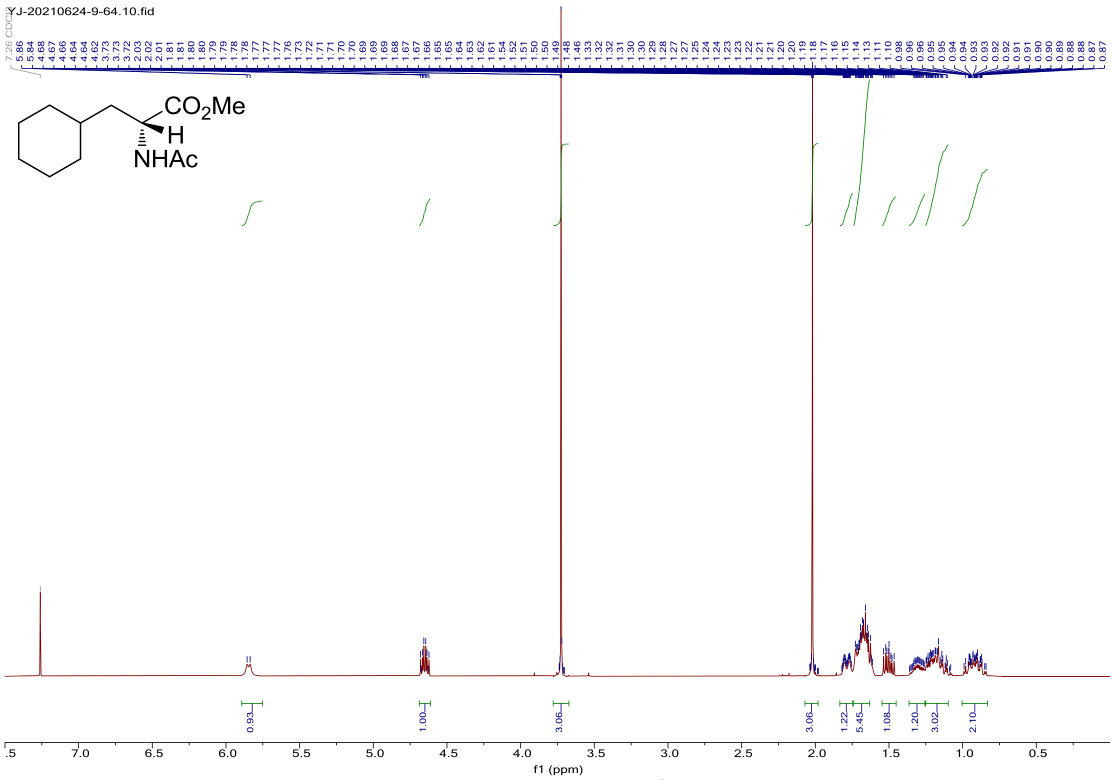
YJ-20210624-9-64.10.fid
7.26 CDCl3
5.86 5.84 4.68 4.67 4.66 4.64 4.64 4.62 3.73 3.72 2.03 2.02 2.01 1.81 1.81 1.80 1.80 1.79 1.79 1.78 1.77 1.77 1.77 1.76 1.73 1.72 1.71 1.71 1.70 1.69 1.69 1.69 1.68 1.67 1.67 1.66 1.65 1.65 1.64 1.63 1.62 1.61 1.54 1.52 1.51 1.50 1.50 1.49 1.48 1.46 1.33 1.32 1.31 1.30 1.30 1.29 1.28 1.27 1.27 1.25 1.24 1.24 1.23 1.23 1.22 1.21 1.21 1.20 1.20 1.19 1.18 1.17 1.16 1.15 1.14 1.13 1.11 1.10 0.98 0.96 0.96 0.95 0.95 0.94 0.94 0.93 0.93 0.92 0.92 0.91 0.91 0.90 0.90 0.89 0.88 0.87 0.87
CO2Me
H
NHAc
0.93
1.00
3.06
3.06
1.22
5.45
1.08
1.20
3.02
2.10
f1 (ppm)

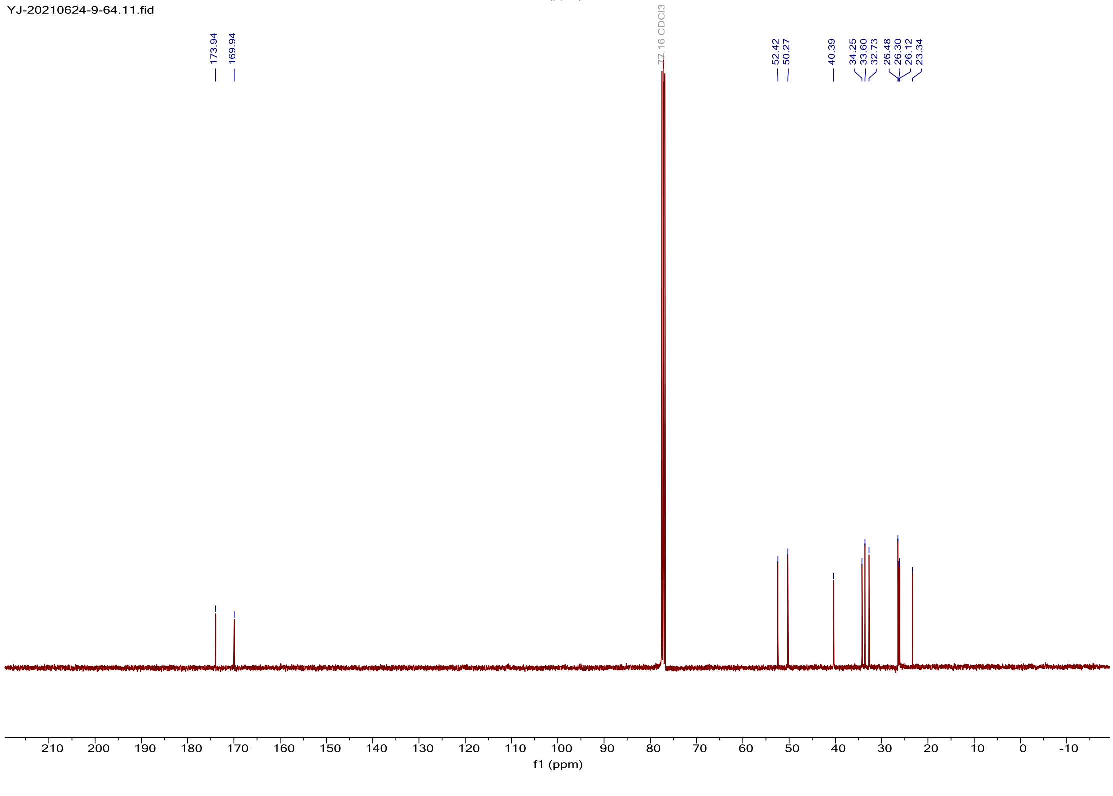
YJ-20210624-9-64.11.fid
173.94
169.94
77.16 CDCl3
52.42
50.27
40.39
34.25
33.60
32.73
26.48
26.30
26.12
23.34
f1 (ppm)

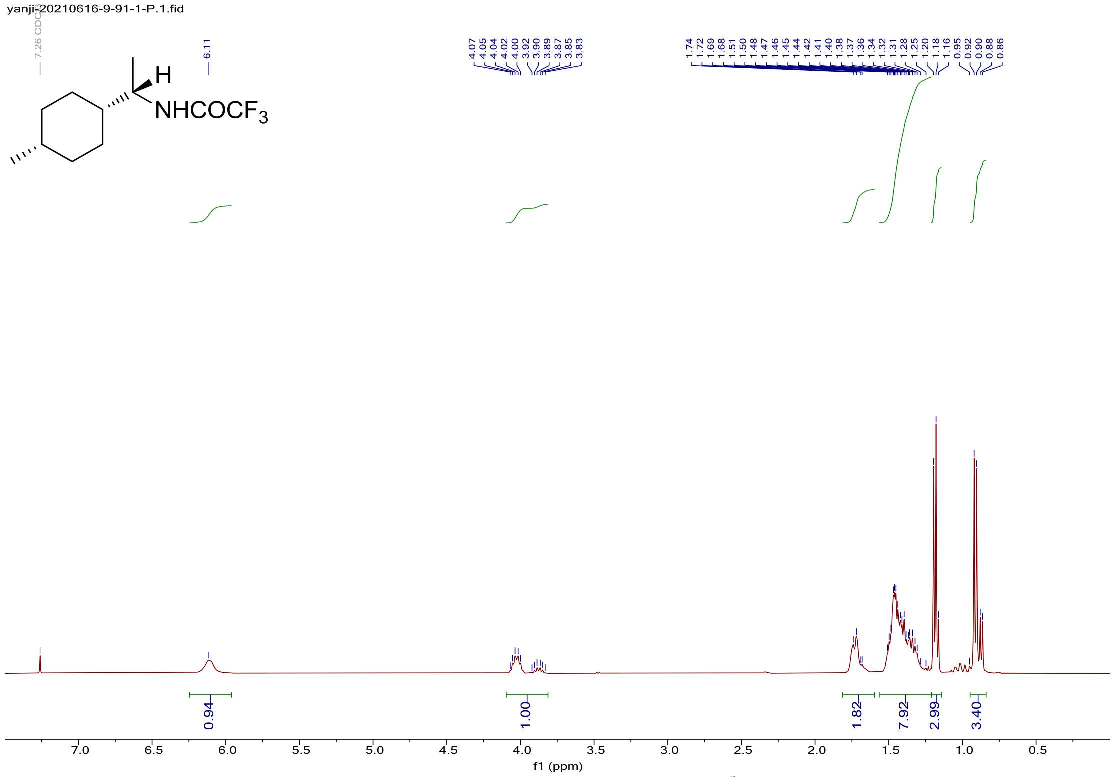
yanji-20210616-9-91-1-P.1.fid
NHCOCF3
f1 (ppm)

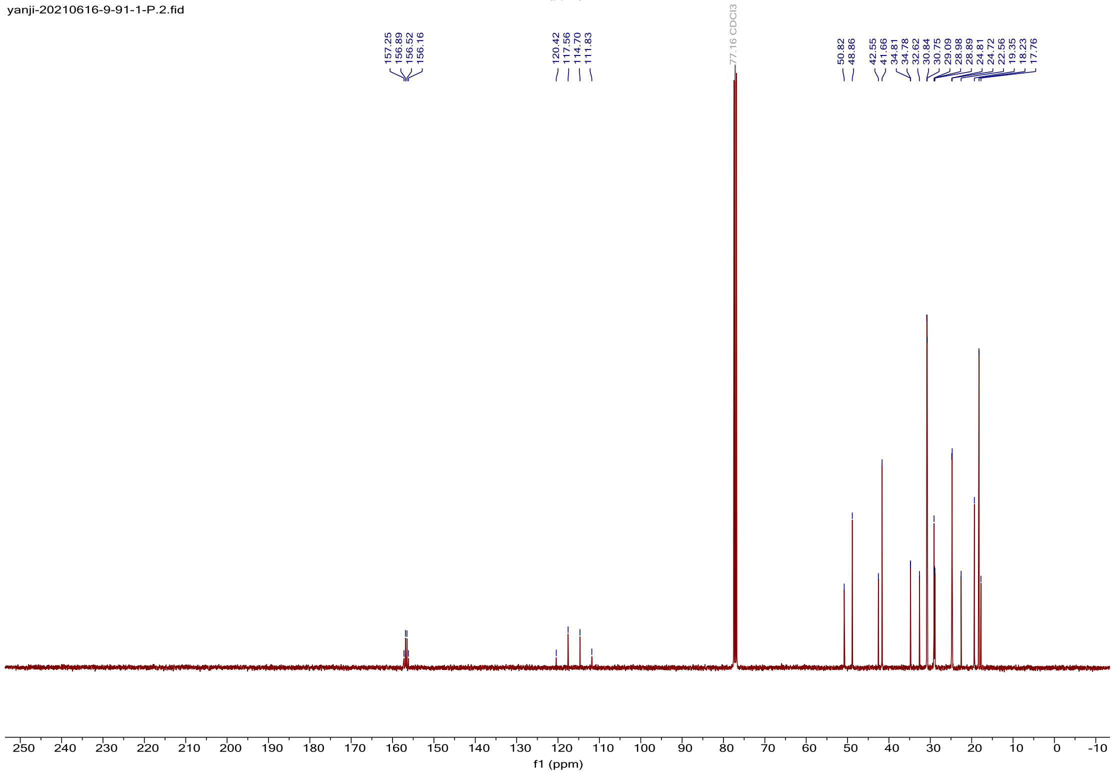
yanji-20210616-9-91-1-P.2.fid
77.16 CDCl3
f1 (ppm)

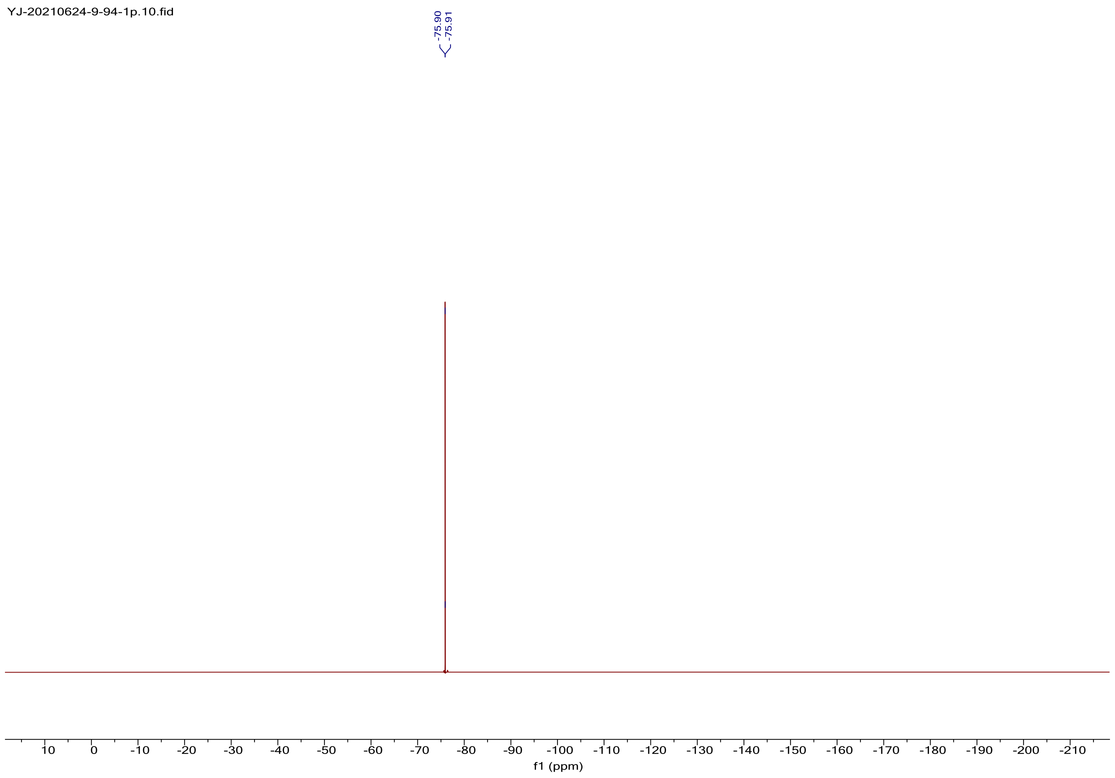
YJ-20210624-9-94-1p.10.fid
-75.90
-75.91
10 0 -10 -20 -30 -40 -50 -60 -70 -80 -90 -100 -110 -120 -130 -140 -150 -160 -170 -180 -190 -200 -210
f1 (ppm)

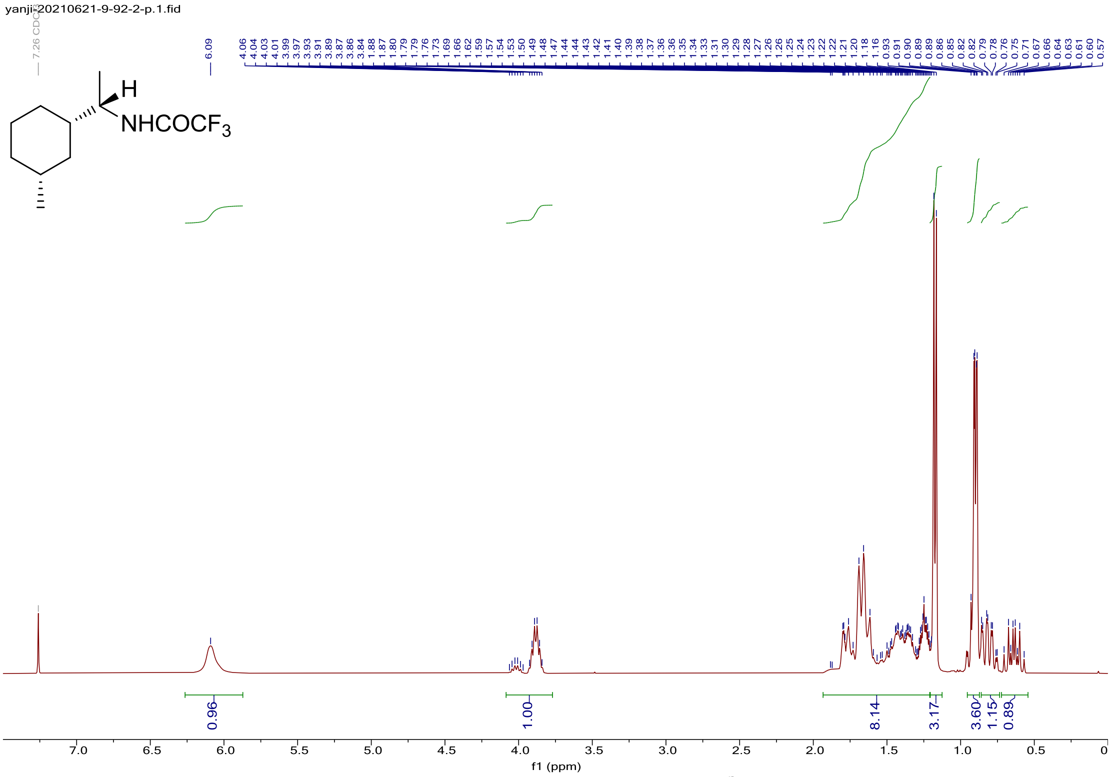

yanji-20210621-9-92-2-p.1.fid
7.26 CDCl3
6.09
4.06 4.04 4.03 4.01 3.99 3.97 3.93 3.91 3.89 3.87 3.86 3.84 1.88 1.87 1.80 1.79 1.79 1.76 1.73 1.69 1.66 1.62 1.59 1.57 1.54 1.53 1.50 1.49 1.48 1.47 1.44 1.44 1.43 1.42 1.41 1.40 1.39 1.38 1.37 1.36 1.36 1.35 1.34 1.33 1.31 1.30 1.29 1.28 1.27 1.26 1.26 1.25 1.24 1.23 1.22 1.22 1.21 1.20 1.18 1.16 0.93 0.91 0.90 0.89 0.89 0.86 0.85 0.82 0.82 0.79 0.78 0.76 0.75 0.71 0.67 0.66 0.64 0.63 0.61 0.60 0.57
H
NHCOCF3
0.96
1.00
8.14
3.17
3.60
1.15
0.89
7.0 6.5 6.0 5.5 5.0 4.5 4.0 3.5 3.0 2.5 2.0 1.5 1.0 0.5 0
f1 (ppm)


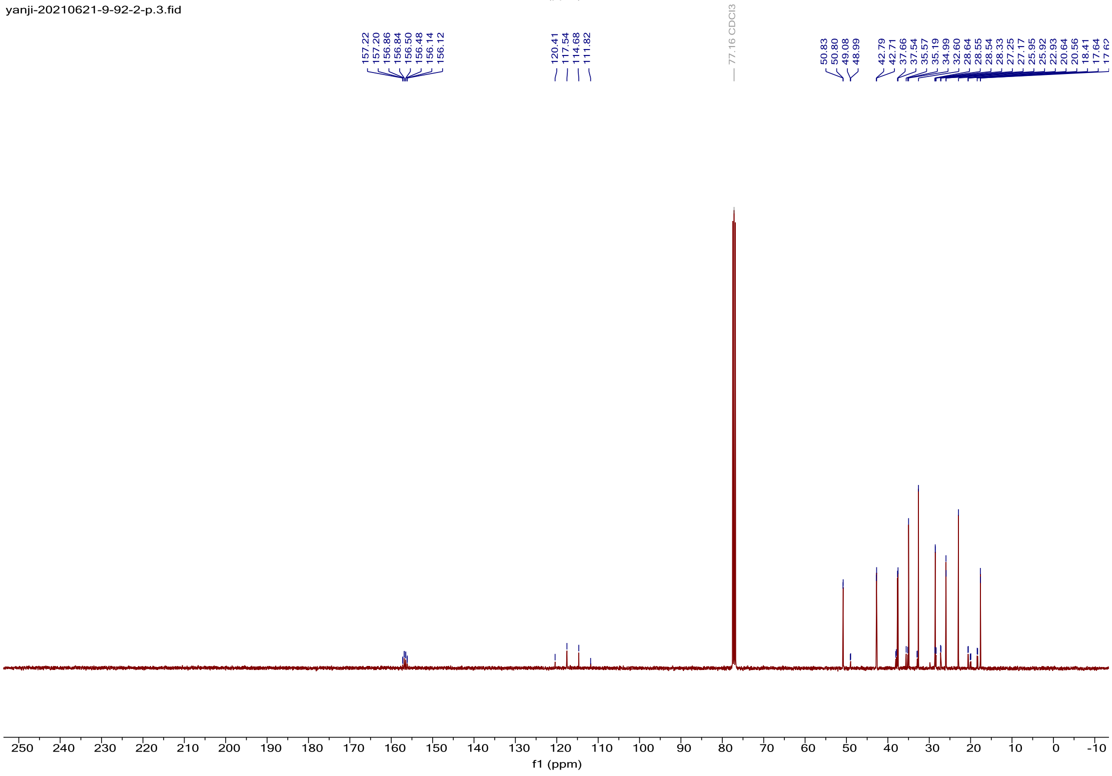

yanji-20210621-9-92-2-p.3.fid
157.22 157.20 156.86 156.84 156.50 156.48 156.14 156.12
120.41 117.54 114.68 111.82
77.16 CDCl3
50.83 50.80 49.08 48.99 42.79 42.71 37.66 37.54 35.57 35.19 34.99 32.60 28.64 28.55 28.54 28.33 27.25 27.17 25.95 25.92 22.93 20.64 20.56 18.41 17.64 17.62
250 240 230 220 210 200 190 180 170 160 150 140 130 120 110 100 90 80 70 60 50 40 30 20 10 0 -10
f1 (ppm)

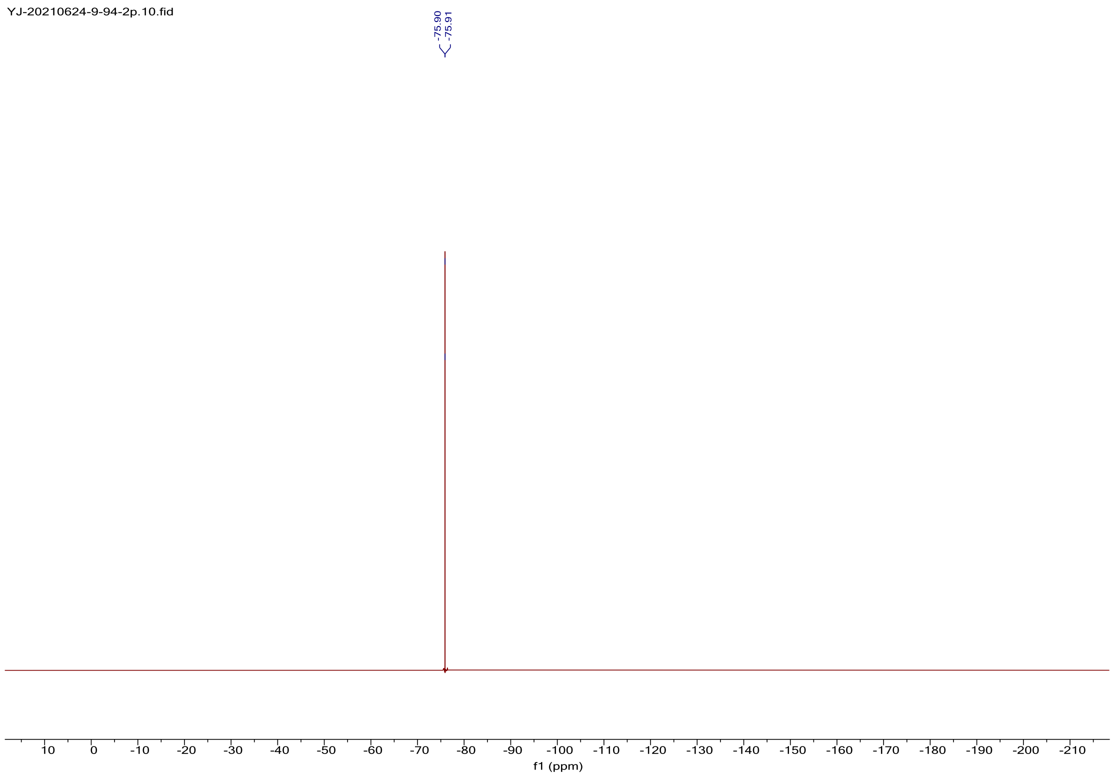
YJ-20210624-9-94-2p.10.fid
-75.90
-75.91
10 0 -10 -20 -30 -40 -50 -60 -70 -80 -90 -100 -110 -120 -130 -140 -150 -160 -170 -180 -190 -200 -210
f1 (ppm)

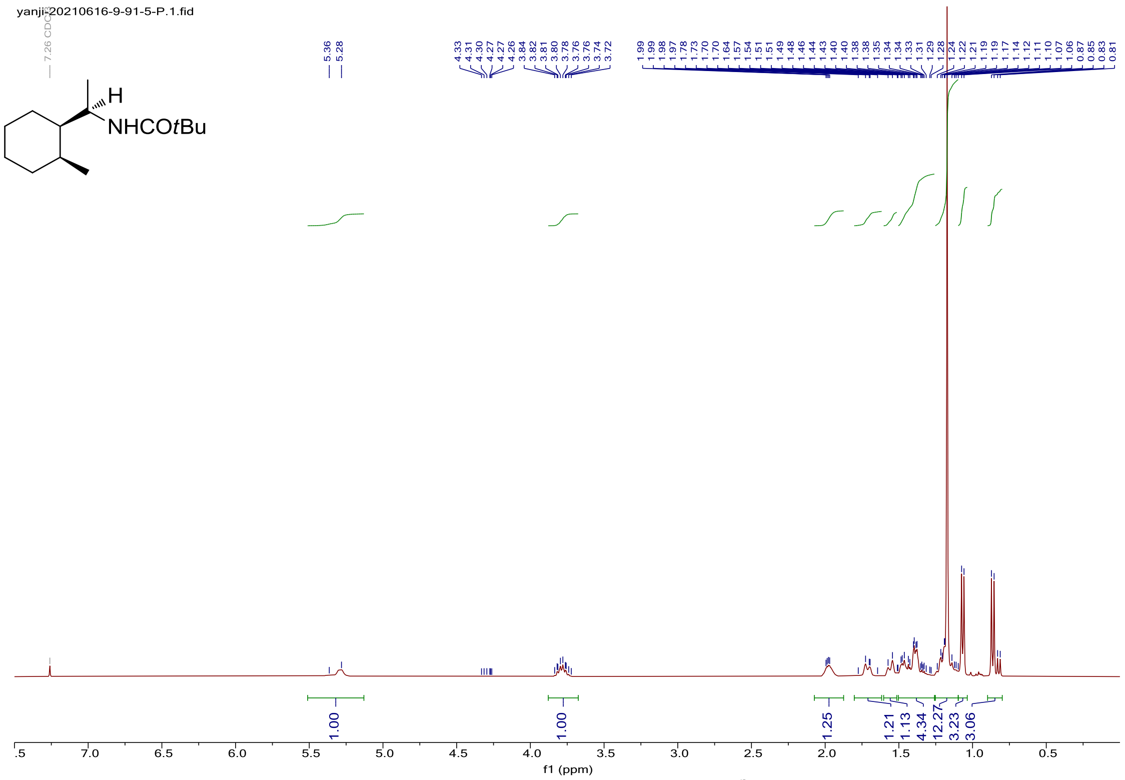
yanji-20210616-9-91-5-P.1.fid
7.26 CDCl3
H
NHCOtBu
5.36
5.28
4.33 4.31 4.30 4.27 4.27 4.26 3.84 3.82 3.81 3.80 3.78 3.76 3.76 3.74 3.72
1.99 1.99 1.98 1.97 1.78 1.73 1.70 1.64 1.57 1.54 1.51 1.49 1.48 1.46 1.44 1.43 1.40 1.40 1.38 1.38 1.35 1.34 1.34 1.33 1.31 1.29 1.28 1.24 1.22 1.21 1.19 1.19 1.17 1.14 1.12 1.11 1.10 1.07 1.06 0.87 0.85 0.83 0.81
1.00
1.00
1.25
1.21
1.13
4.34
12.27
3.23
3.06
f1 (ppm)
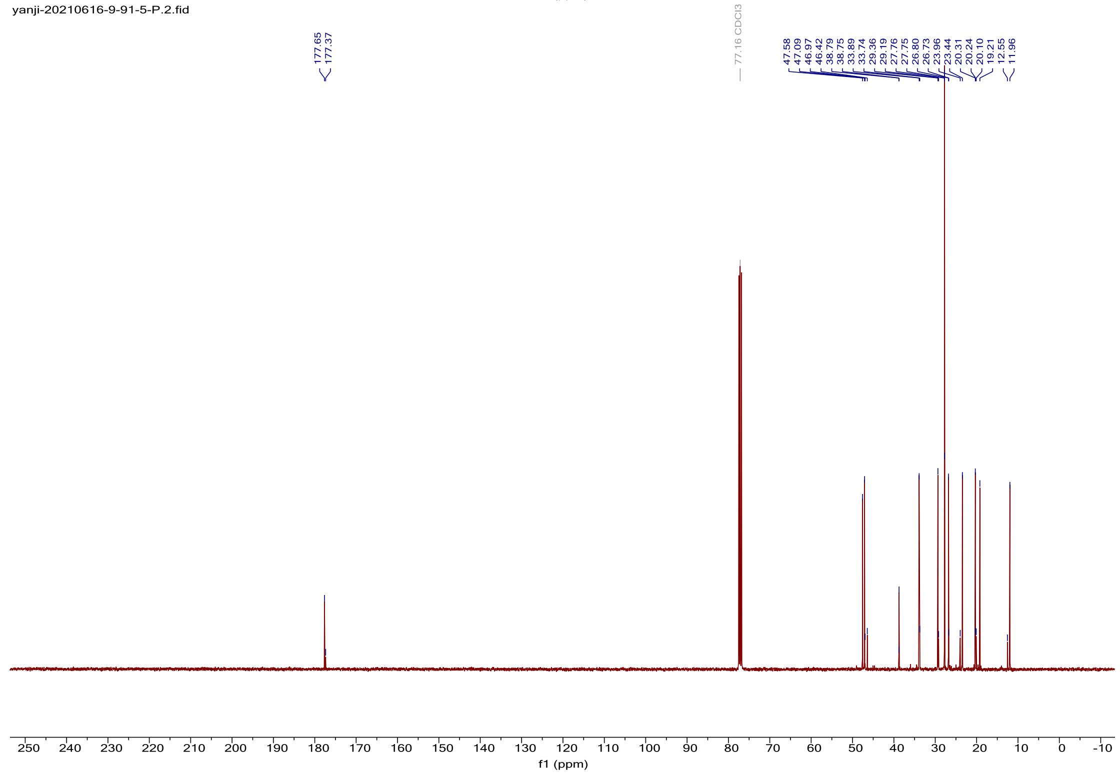
yanji-20210616-9-91-5-P.2.fid
177.65
177.37
77.16 CDCl3
47.58 47.09 46.97 46.42 38.79 38.75 33.89 33.74 29.36 29.19 27.76 27.75 26.80 26.73 23.96 23.44 20.31 20.24 20.10 19.21 12.55 11.96
f1 (ppm)

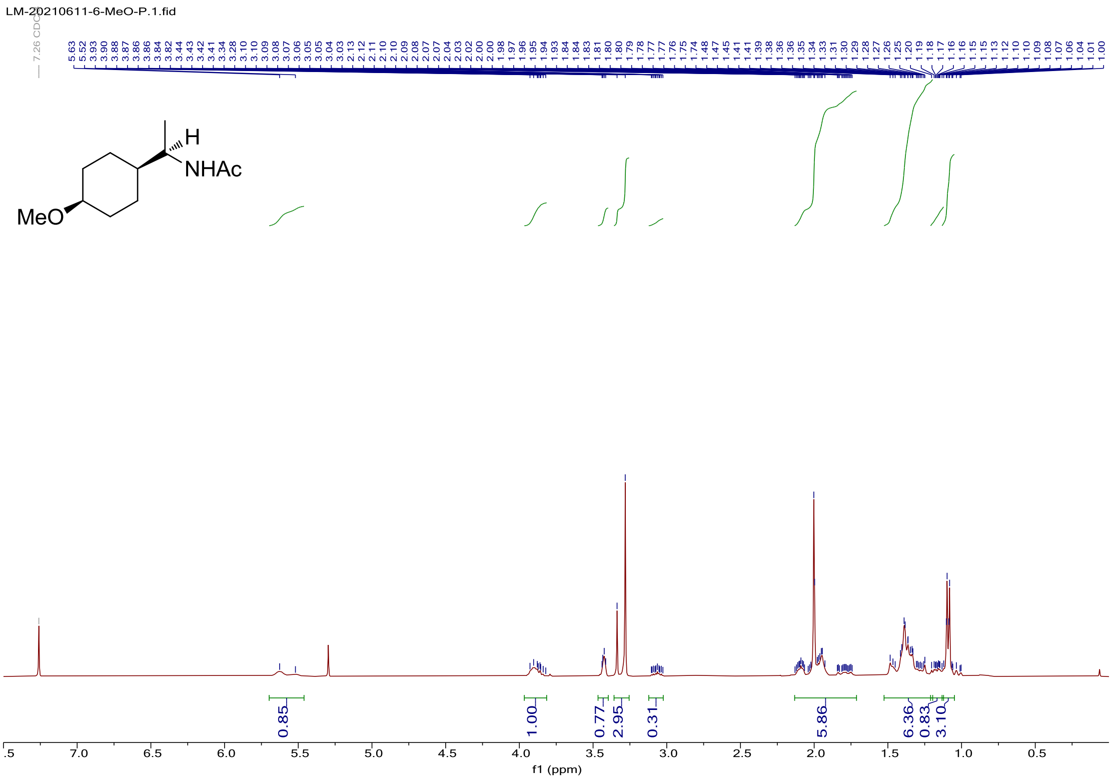
LM-20210611-6-MeO-P.1.fid
H
NHAc
MeO
f1 (ppm)

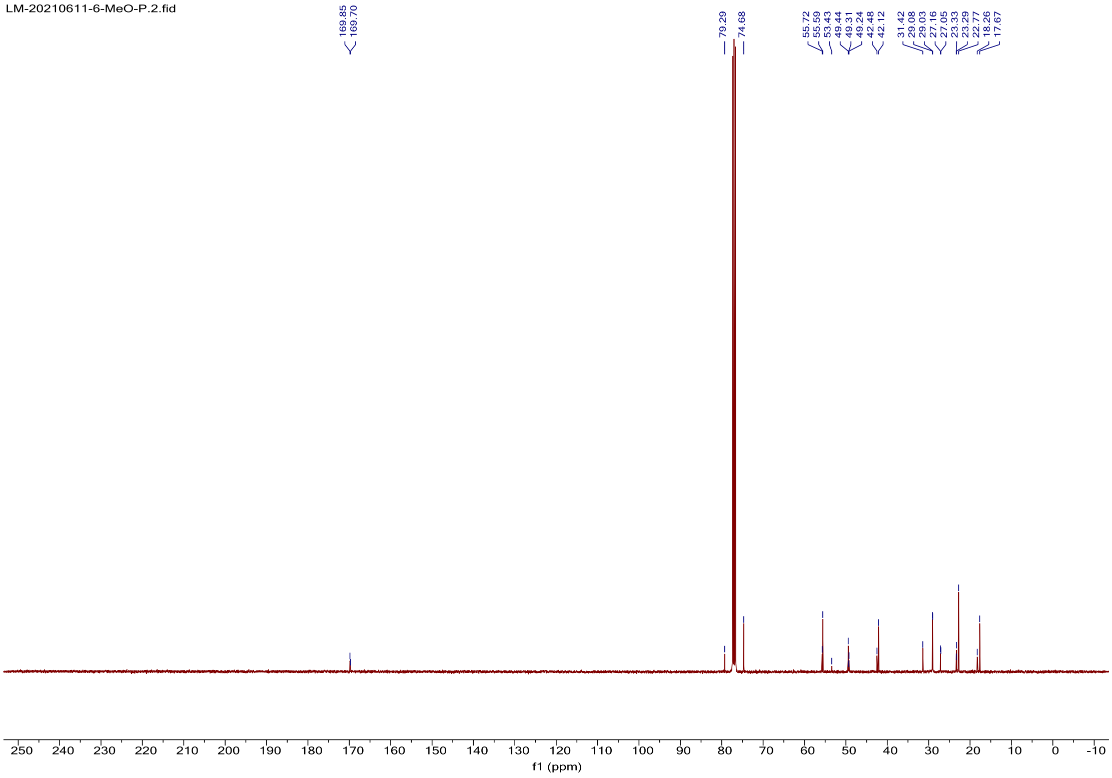
LM-20210611-6-MeO-P.2.fid
169.85
169.70
79.29
74.68
55.72
55.59
53.43
49.44
49.31
49.24
42.48
42.12
31.42
29.08
29.03
27.16
27.05
23.33
23.29
22.77
18.26
17.67
f1 (ppm)

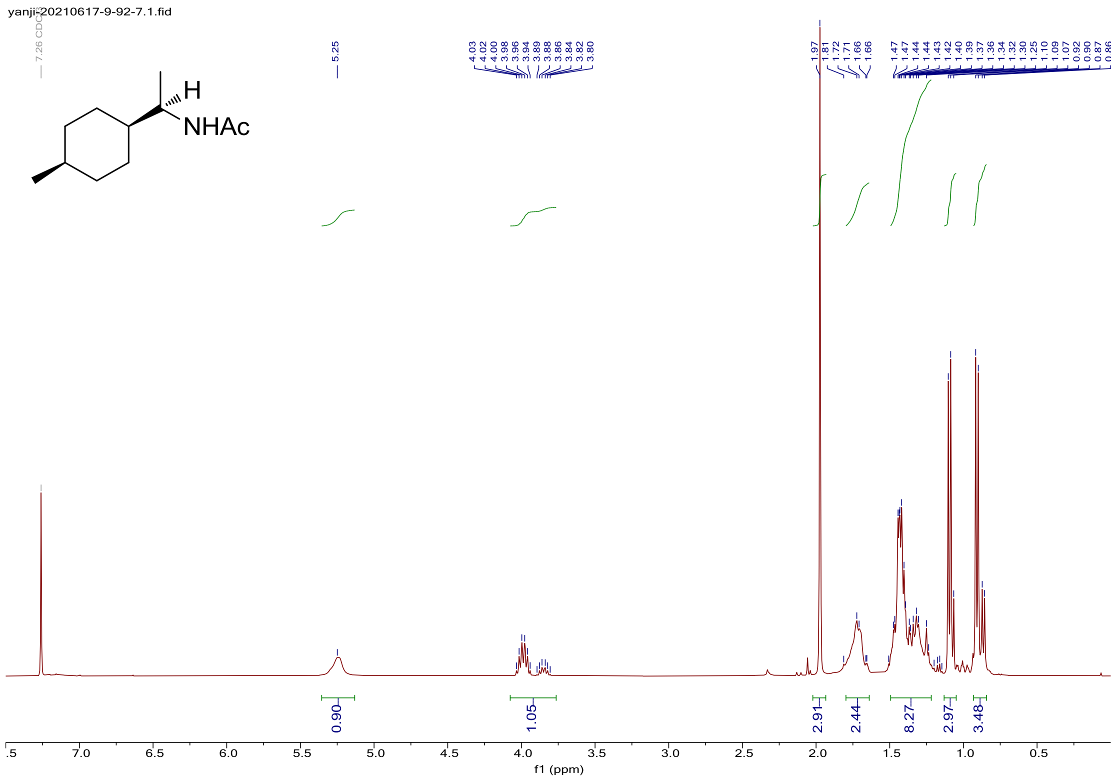
yanji-20210617-9-92-7.1.fid
H
NHAc
0.90
1.05
2.91
2.44
8.27
2.97
3.48
f1 (ppm)

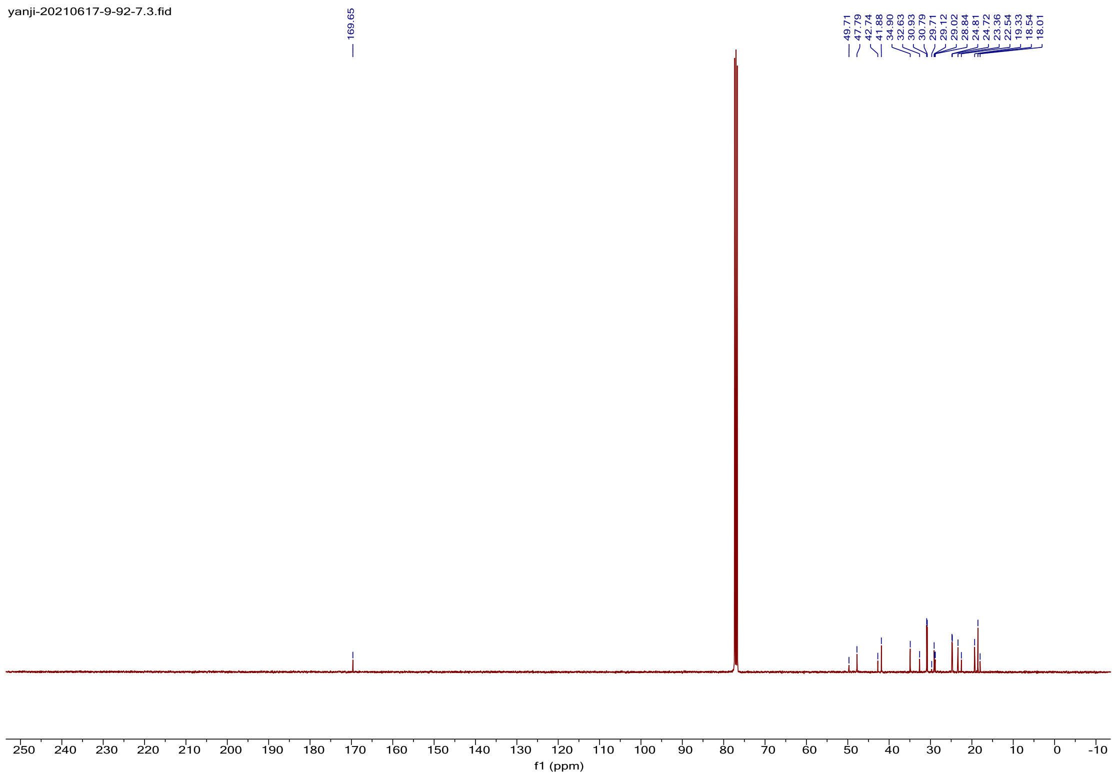
yanji-20210617-9-92-7.3.fid
169.65
49.71
47.79
42.74
41.88
34.90
32.63
30.93
30.79
29.71
29.12
29.02
28.84
24.81
24.72
23.36
22.54
19.33
18.54
18.01
f1 (ppm)

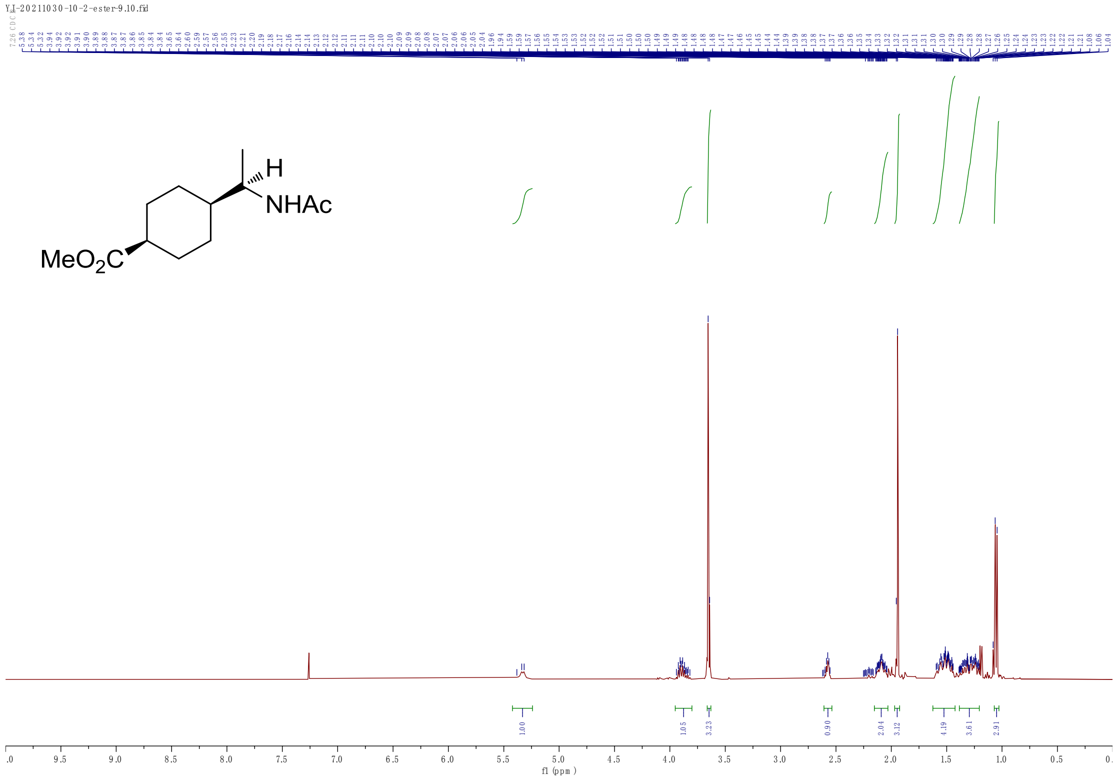
YJ-20211030-10-2-ester-9.10.fid
H
NHAc
MeO2C
1.00
1.05
3.23
0.90
2.04
3.12
4.39
3.61
2.91
f1 (ppm)

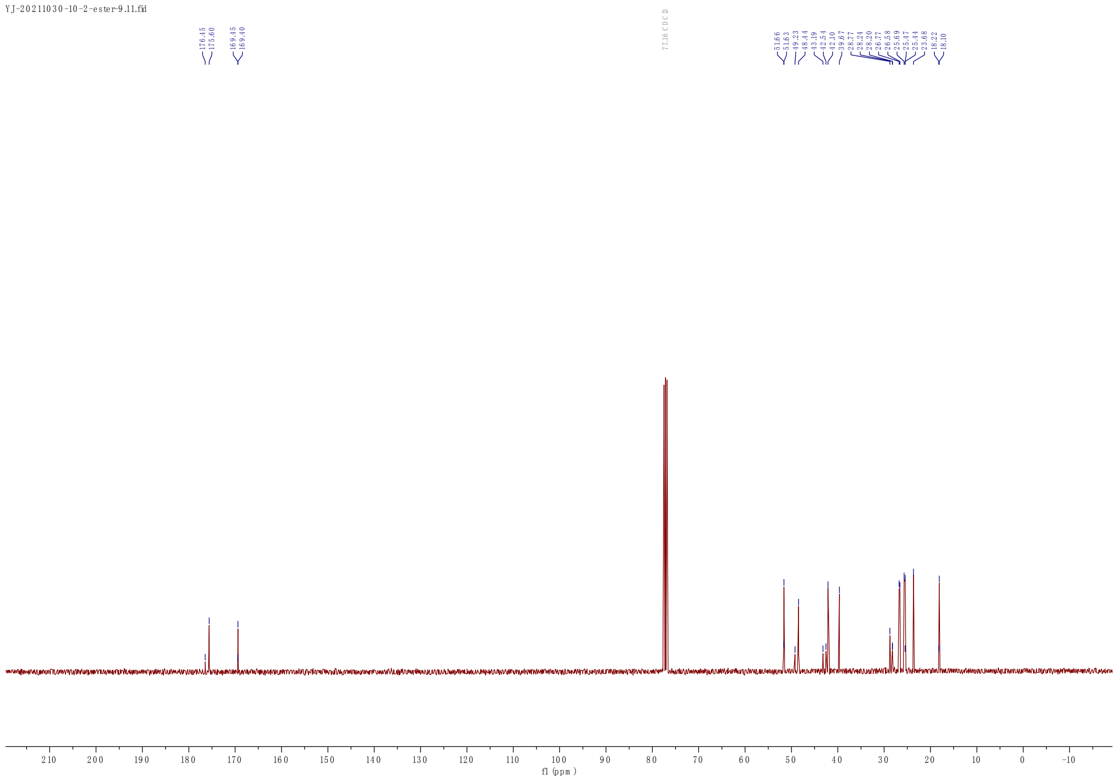
YJ-20211030-10-2-ester-9.11.fid
f1 (ppm)

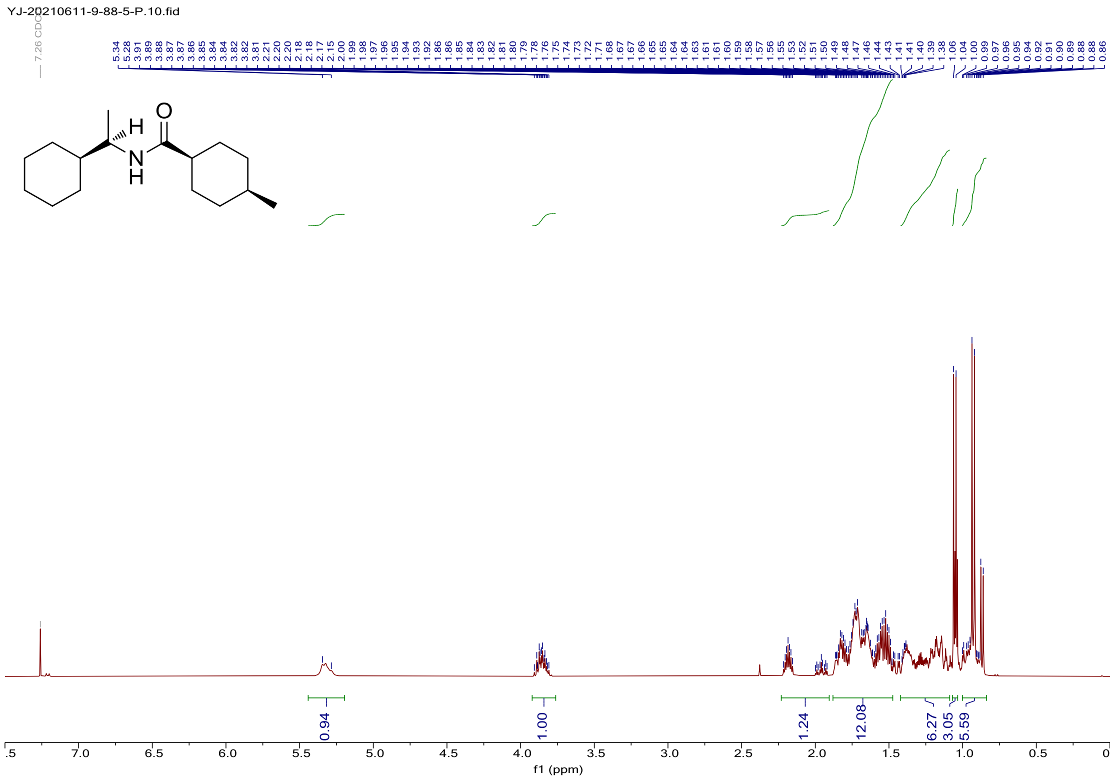
YJ-20210611-9-88-5-P.10.fid
7.26 CDCl3
5.34 5.28 3.91 3.89 3.88 3.87 3.87 3.86 3.85 3.84 3.84 3.82 3.82 3.81 2.21 2.20 2.20 2.18 2.18 2.17 2.15 2.00 1.99 1.98 1.97 1.96 1.95 1.94 1.93 1.92 1.86 1.86 1.85 1.84 1.83 1.82 1.81 1.80 1.79 1.78 1.76 1.75 1.74 1.73 1.72 1.71 1.68 1.67 1.67 1.66 1.65 1.65 1.64 1.64 1.63 1.61 1.61 1.60 1.59 1.58 1.57 1.56 1.55 1.53 1.52 1.51 1.50 1.49 1.48 1.47 1.46 1.44 1.43 1.41 1.41 1.40 1.39 1.38 1.06 1.04 1.00 0.99 0.97 0.96 0.95 0.94 0.92 0.91 0.90 0.89 0.88 0.88 0.86
H
N
H
O
0.94
1.00
1.24
12.08
6.27
3.05
5.59
f1 (ppm)

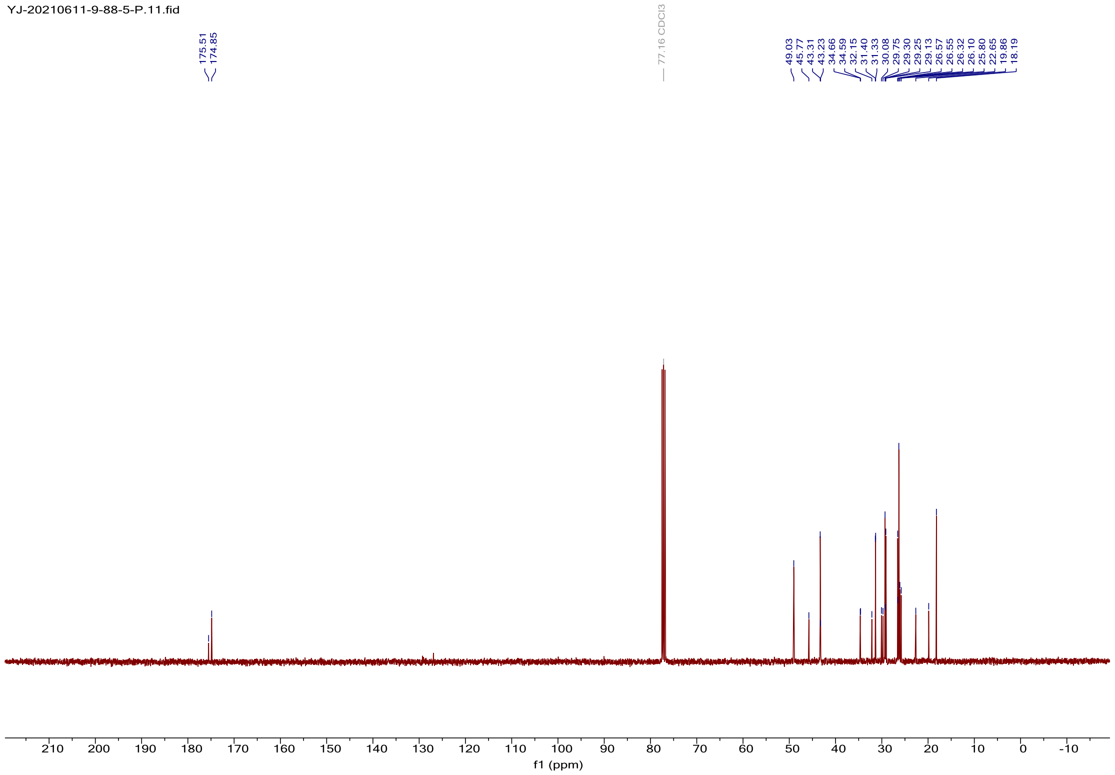
YJ-20210611-9-88-5-P.11.fid
175.51
174.85
77.16 CDCl3
49.03 45.77 43.31 43.23 34.66 34.59 32.15 31.40 31.33 30.08 29.75 29.30 29.25 29.13 26.57 26.55 26.32 26.10 25.80 22.65 19.86 18.19
f1 (ppm)

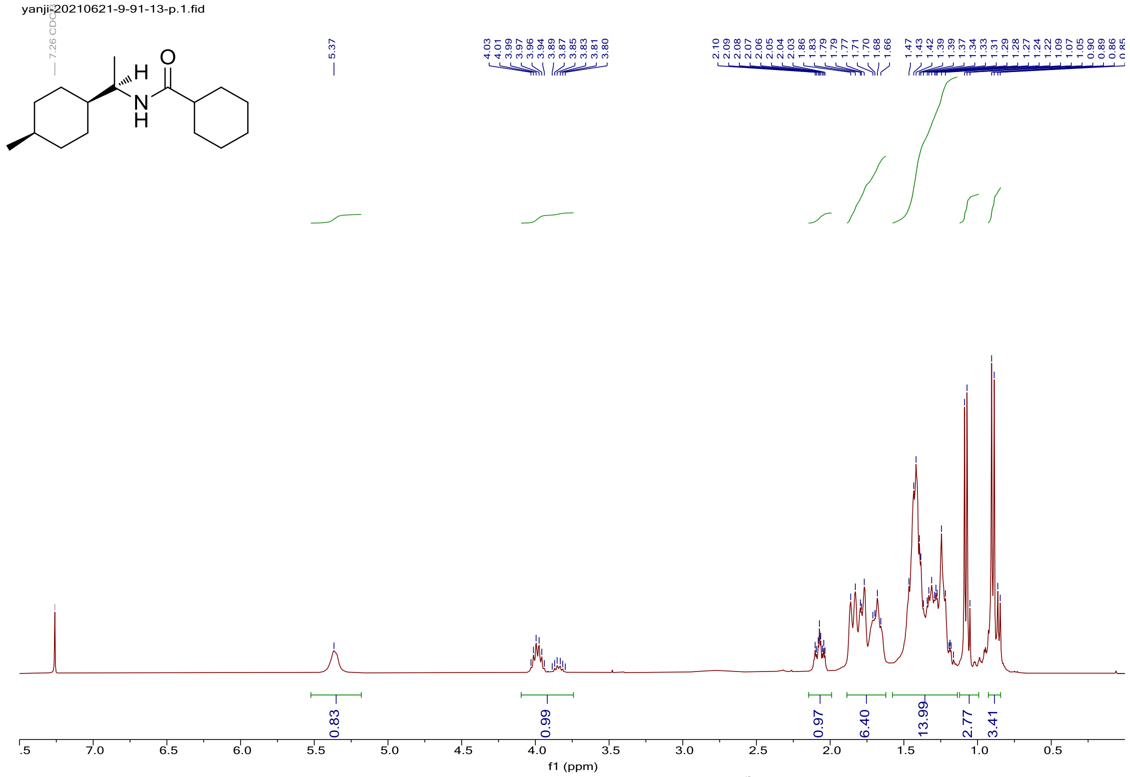
yanji-20210621-9-91-13-p.1.fid
7.26 CDCl3
5.37
0.83
0.99
0.97
6.40
13.99
2.77
3.41
f1 (ppm)

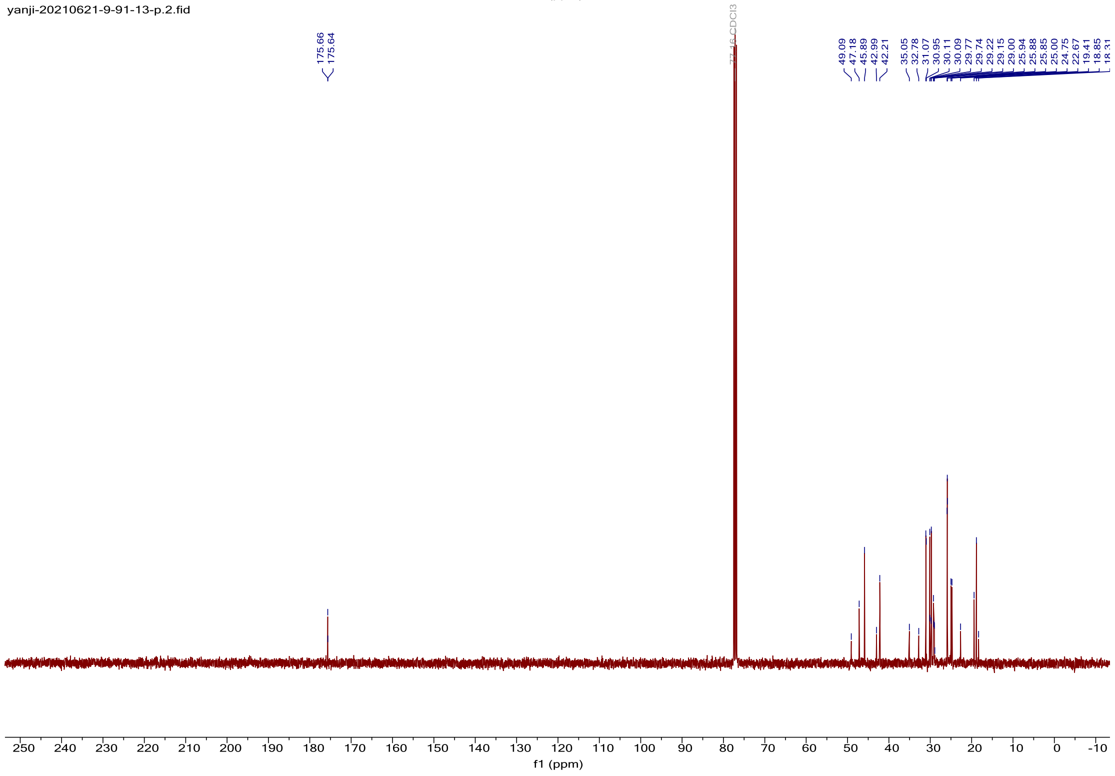
yanji-20210621-9-91-13-p.2.fid
175.66
175.64
49.09
47.18
45.89
42.99
42.21
35.05
32.78
31.07
30.95
30.11
30.09
29.77
29.74
29.22
29.15
29.00
25.94
25.88
25.85
25.00
24.75
22.67
19.41
18.85
f1 (ppm)

YJ-20210625-9-94-5-2.10.fid

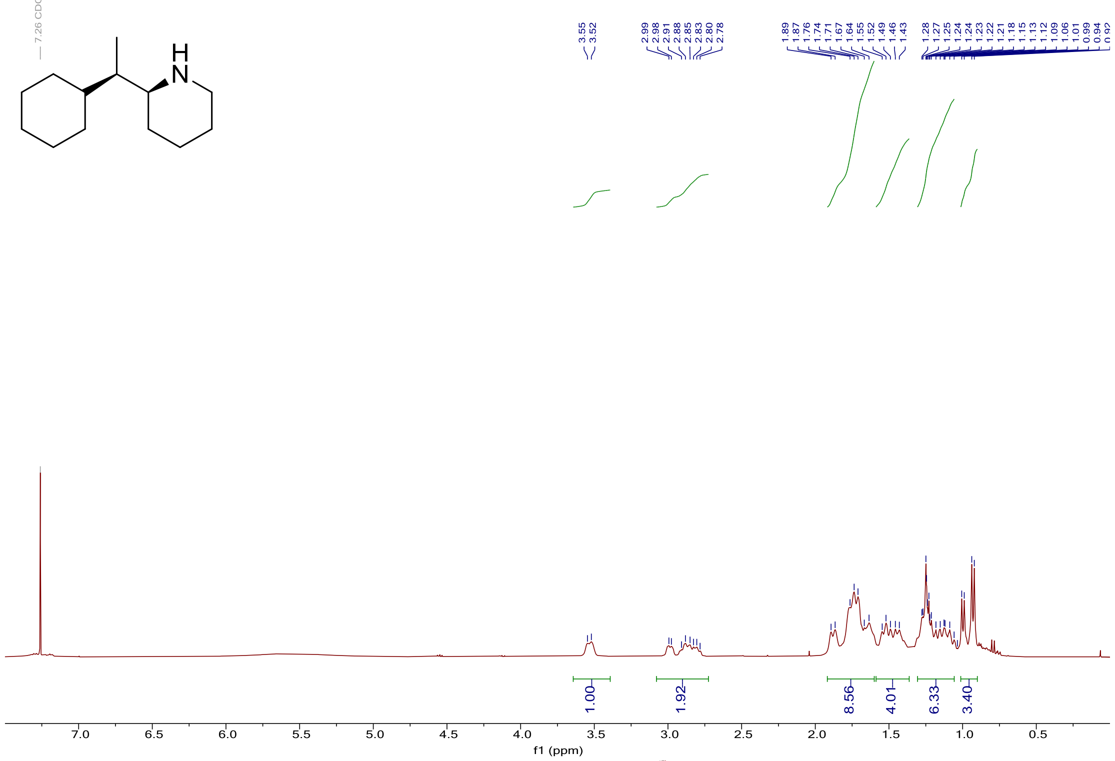


YJ-20210625-9-94-5-2.11.fid

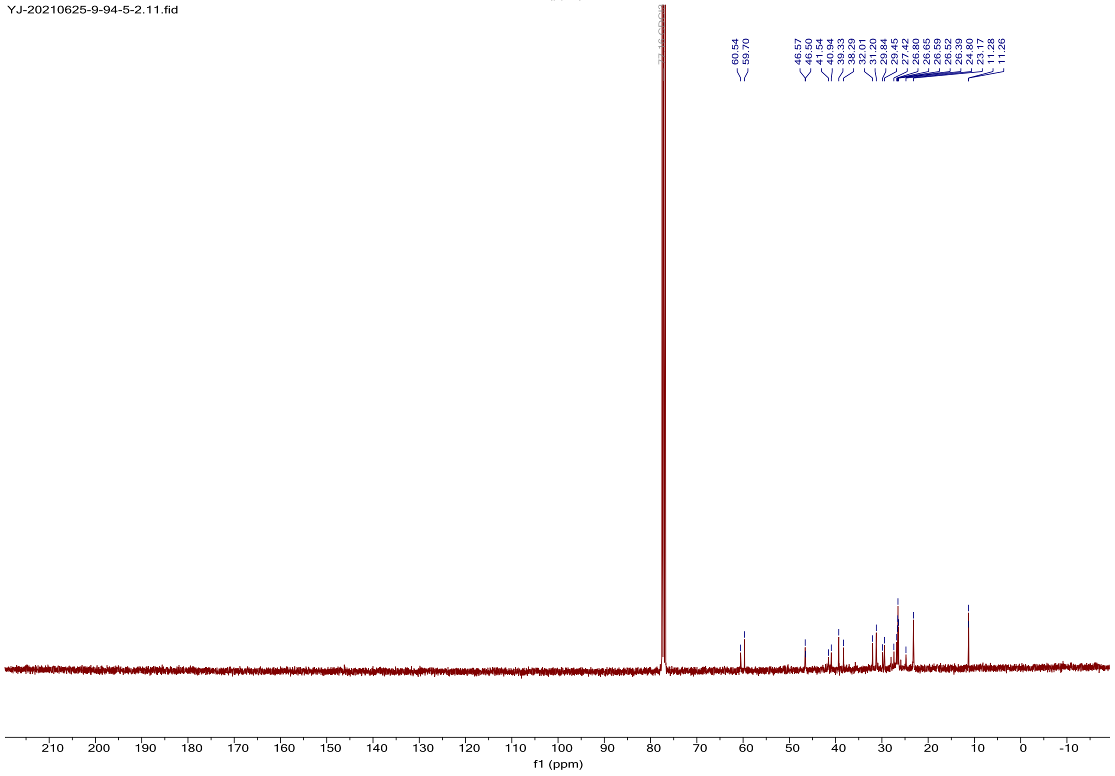

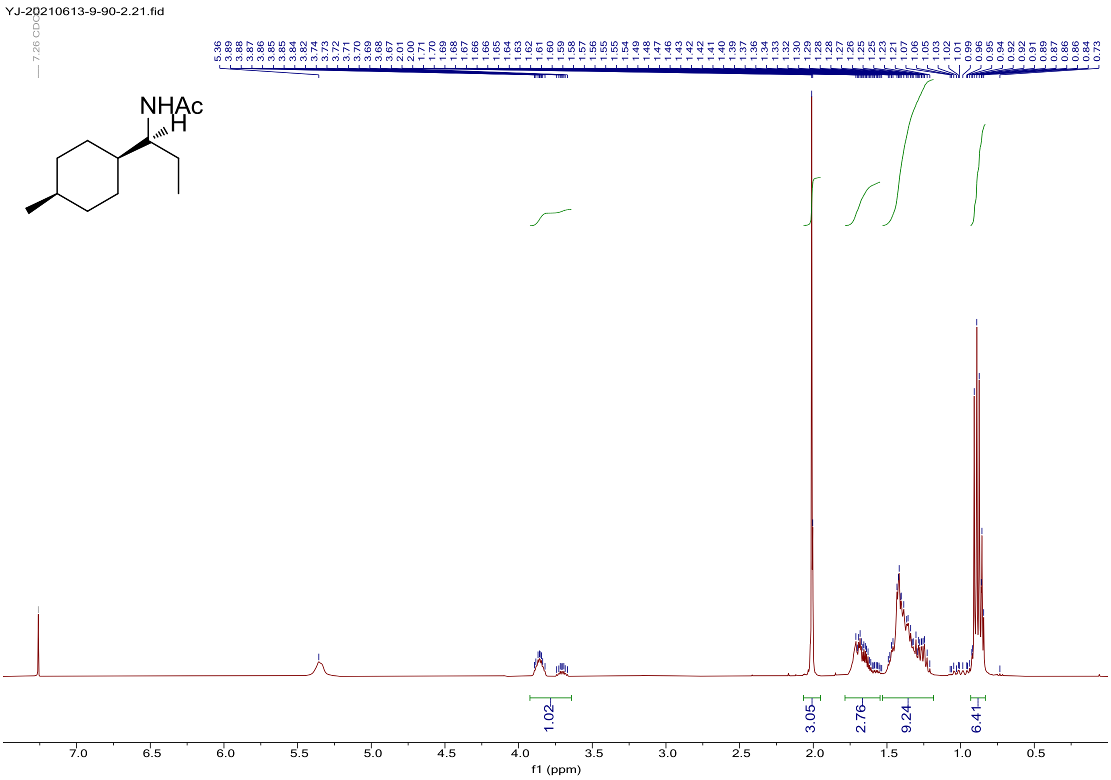
YJ-20210613-9-90-2.21.fid
NHAc
H
7.26 CDCl3
5.36 3.89 3.88 3.87 3.86 3.85 3.85 3.84 3.82 3.74 3.73 3.72 3.71 3.70 3.69 3.68 3.67 2.01 2.00 1.71 1.70 1.69 1.68 1.67 1.66 1.66 1.65 1.64 1.63 1.62 1.61 1.60 1.59 1.58 1.57 1.56 1.55 1.55 1.54 1.49 1.48 1.47 1.46 1.43 1.42 1.42 1.41 1.40 1.39 1.37 1.36 1.34 1.33 1.32 1.30 1.29 1.28 1.28 1.27 1.26 1.25 1.25 1.23 1.21 1.07 1.06 1.05 1.03 1.02 1.01 0.99 0.96 0.95 0.94 0.92 0.92 0.91 0.89 0.87 0.86 0.86 0.84 0.73
1.02
3.05
2.76
9.24
6.41
f1 (ppm)

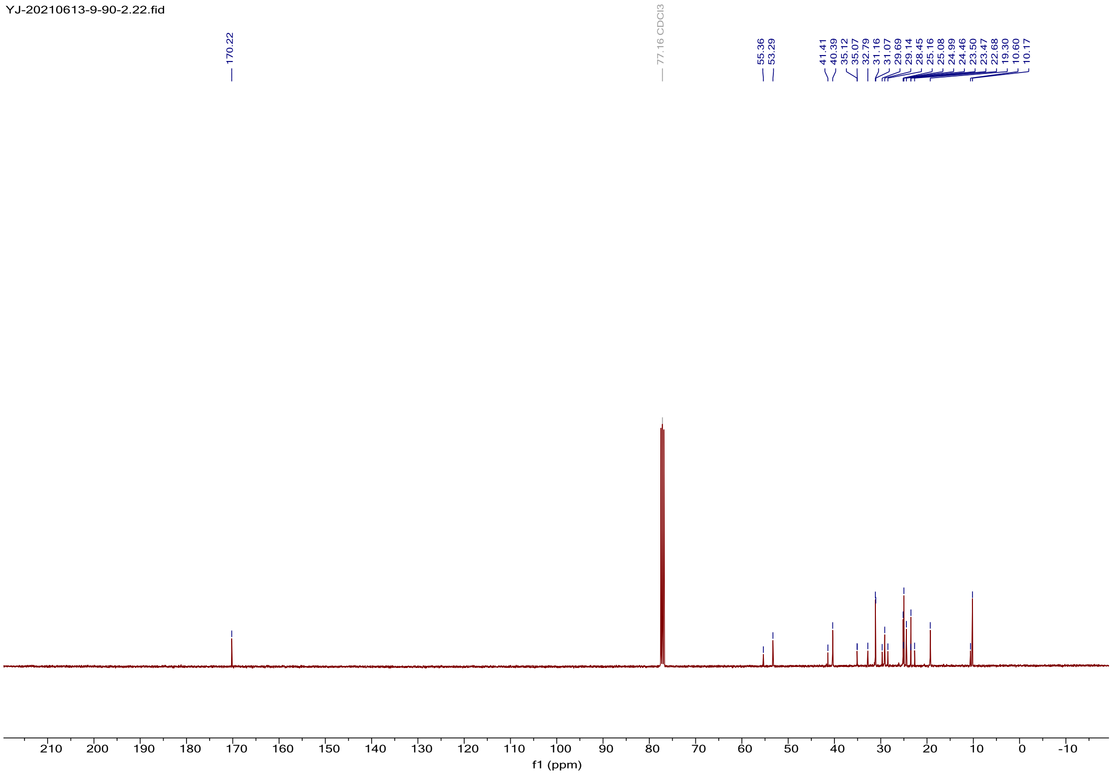
YJ-20210613-9-90-2.22.fid
170.22
77.16 CDCl3
55.36 53.29
41.41 40.39 35.12 35.07 32.79 31.16 31.07 29.69 29.14 28.45 25.16 25.08 24.99 24.46 23.50 23.47 22.68 19.30 10.60 10.17
f1 (ppm)

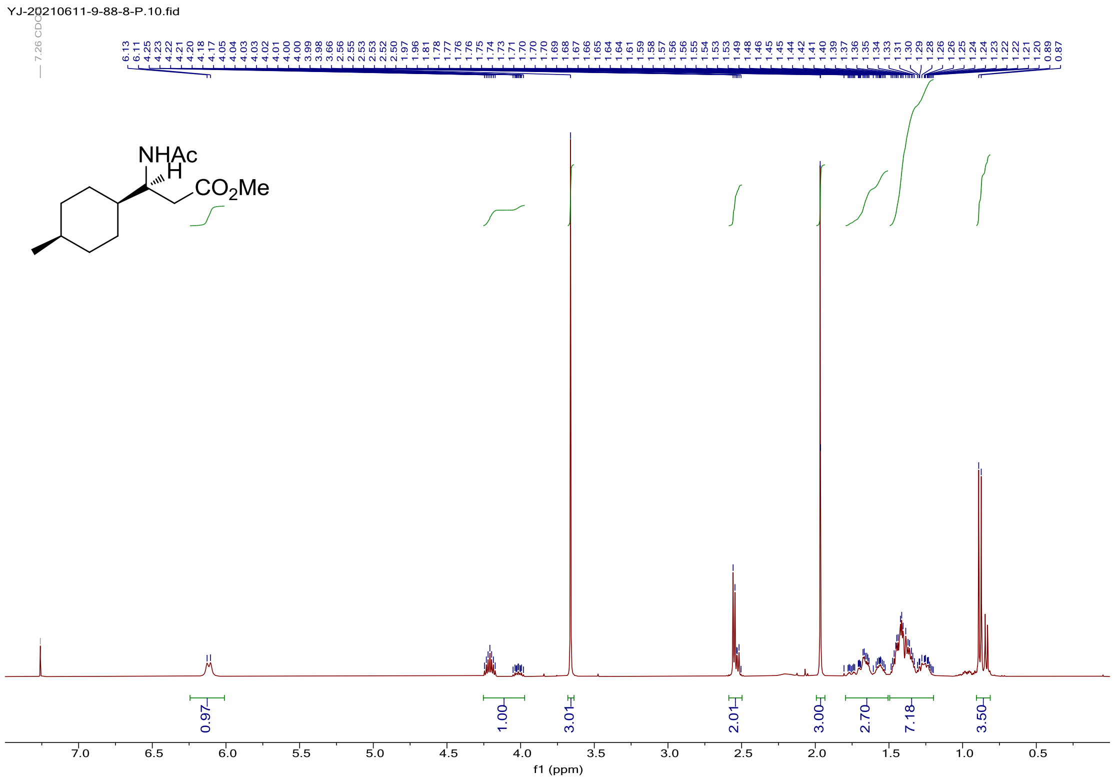
YJ-20210611-9-88-8-P.10.fid
7.26 CDCl3
6.13 6.11 4.25 4.23 4.22 4.21 4.20 4.18 4.17 4.05 4.04 4.03 4.03 4.02 4.01 4.00 4.00 3.99 3.98 3.66 2.56 2.55 2.53 2.53 2.52 2.50 1.97 1.96 1.81 1.78 1.77 1.76 1.75 1.74 1.73 1.71 1.70 1.70 1.69 1.68 1.67 1.66 1.65 1.64 1.64 1.61 1.59 1.58 1.57 1.56 1.56 1.55 1.54 1.53 1.53 1.49 1.48 1.46 1.45 1.45 1.44 1.42 1.41 1.40 1.39 1.37 1.36 1.35 1.34 1.33 1.31 1.30 1.29 1.28 1.26 1.25 1.24 1.24 1.23 1.22 1.21 1.20 0.89 0.87
NHAc
H
CO2Me
0.97
1.00
3.01
2.01
3.00
2.70
7.18
3.50
7.0 6.5 6.0 5.5 5.0 4.5 4.0 3.5 3.0 2.5 2.0 1.5 1.0 0.5
f1 (ppm)

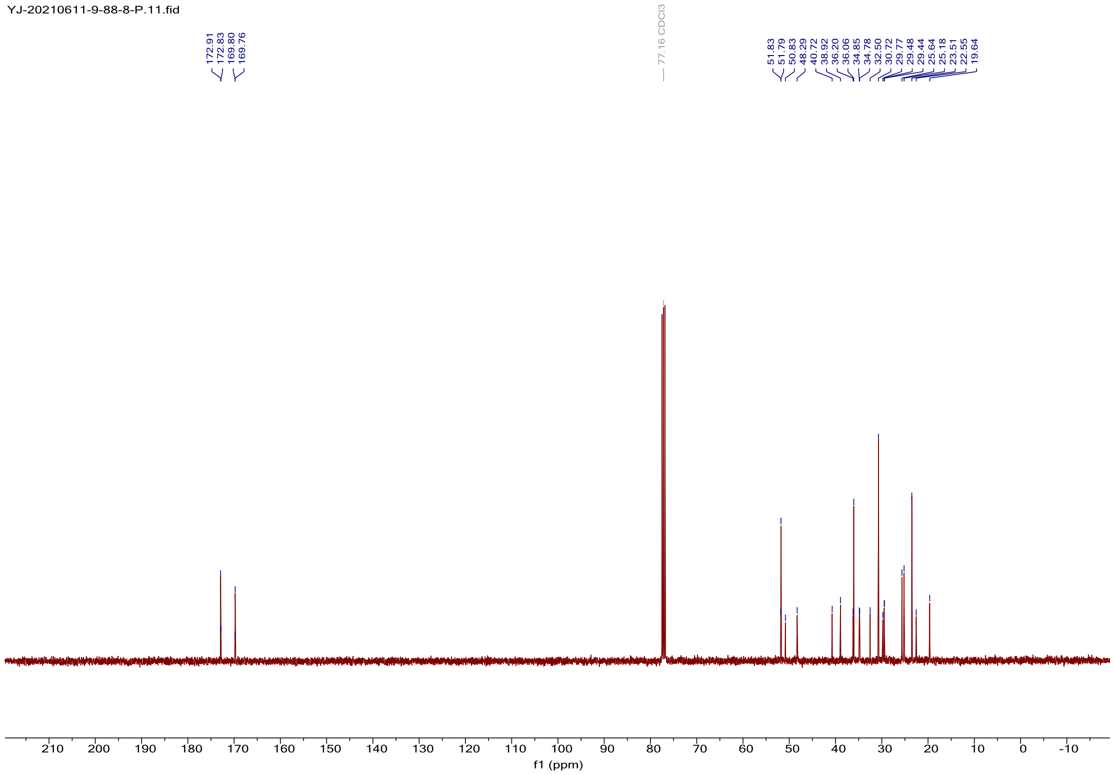
YJ-20210611-9-88-8-P.11.fid
172.91 172.83 169.80 169.76
77.16 CDCl3
51.83 51.79 50.83 48.29 40.72 38.92 36.20 36.06 34.85 34.78 32.50 30.72 29.77 29.48 29.44 25.64 25.18 23.51 22.55 19.64
210 200 190 180 170 160 150 140 130 120 110 100 90 80 70 60 50 40 30 20 10 0 -10
f1 (ppm)

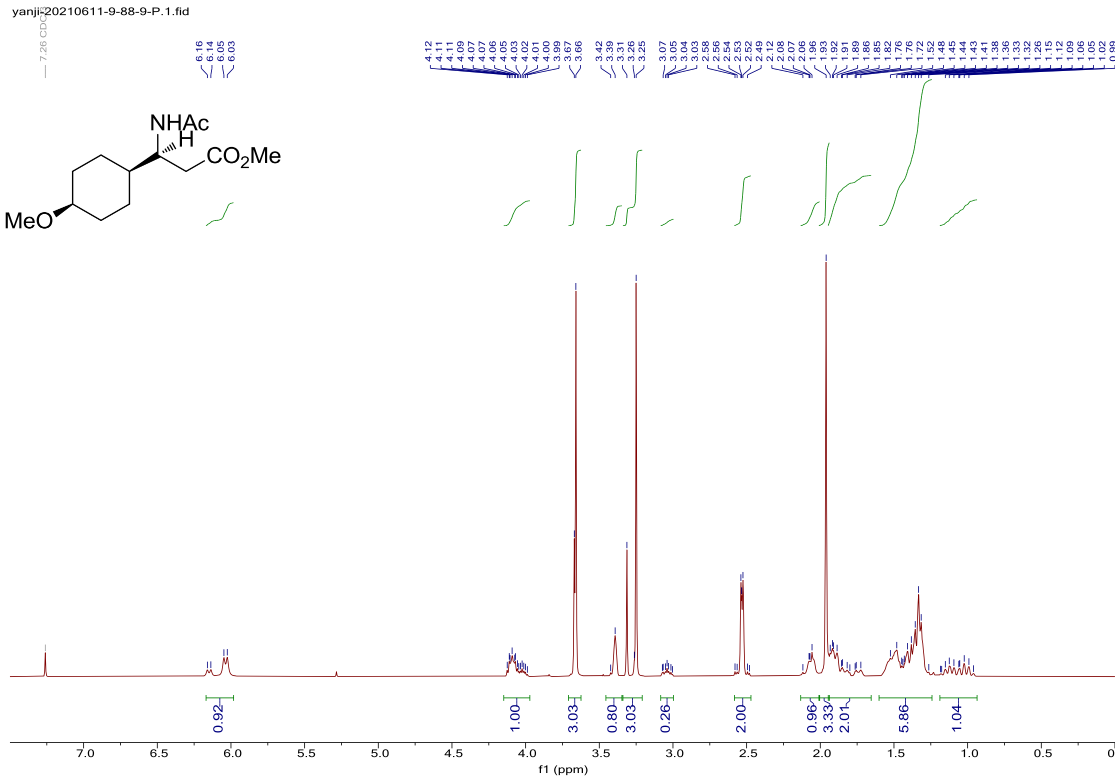
yanji-20210611-9-88-9-P.1.fid
NHAc
H
CO2Me
MeO
f1 (ppm)

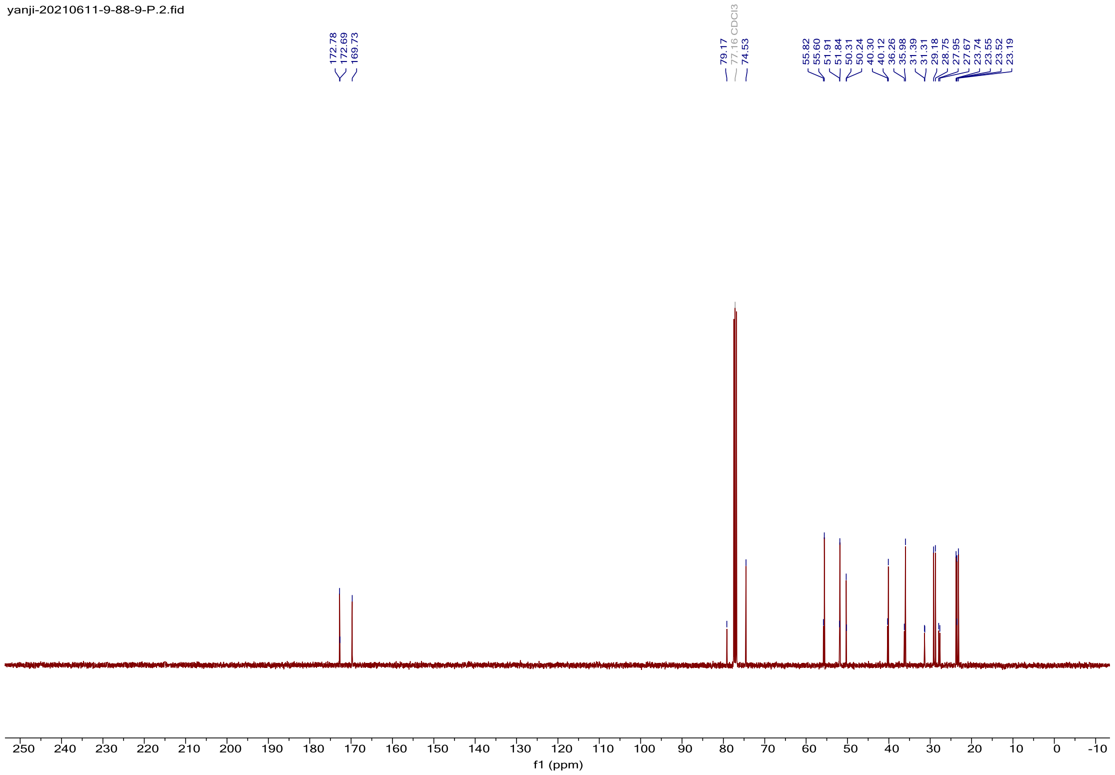
yanji-20210611-9-88-9-P.2.fid
f1 (ppm)

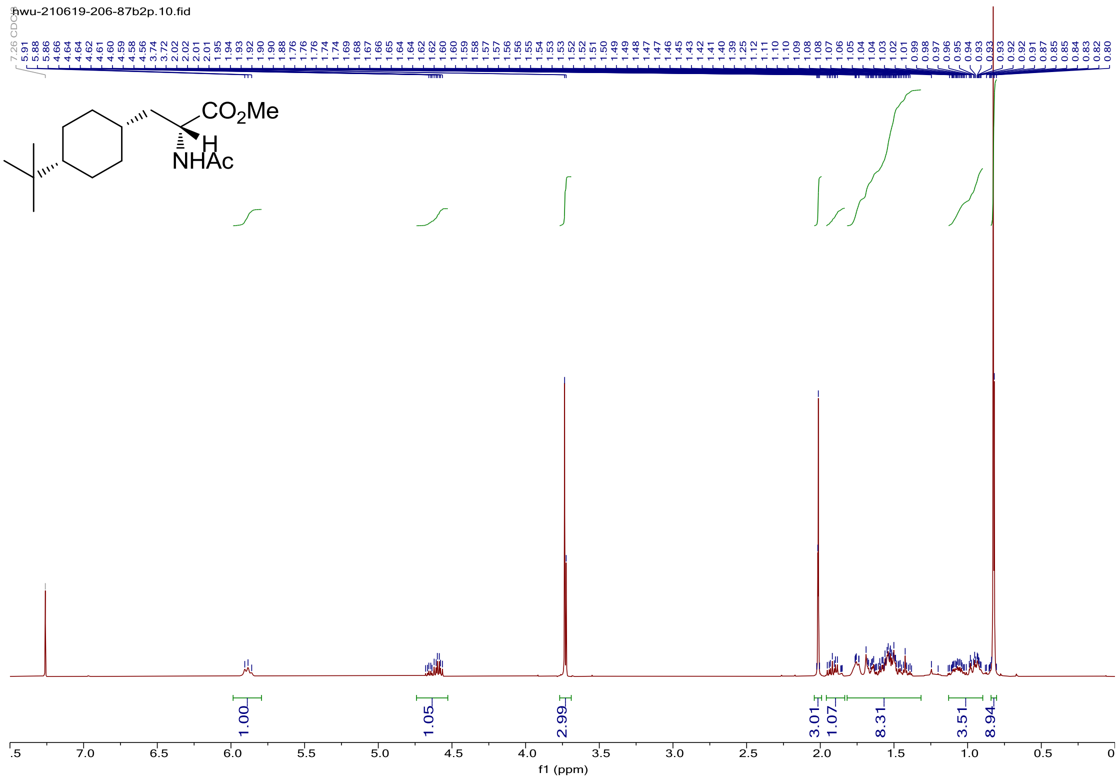
hwu-210619-206-87b2p.10.fid
CO2Me
H
NHAc
1.00
1.05
2.99
3.01
1.07
8.31
3.51
8.94
f1 (ppm)

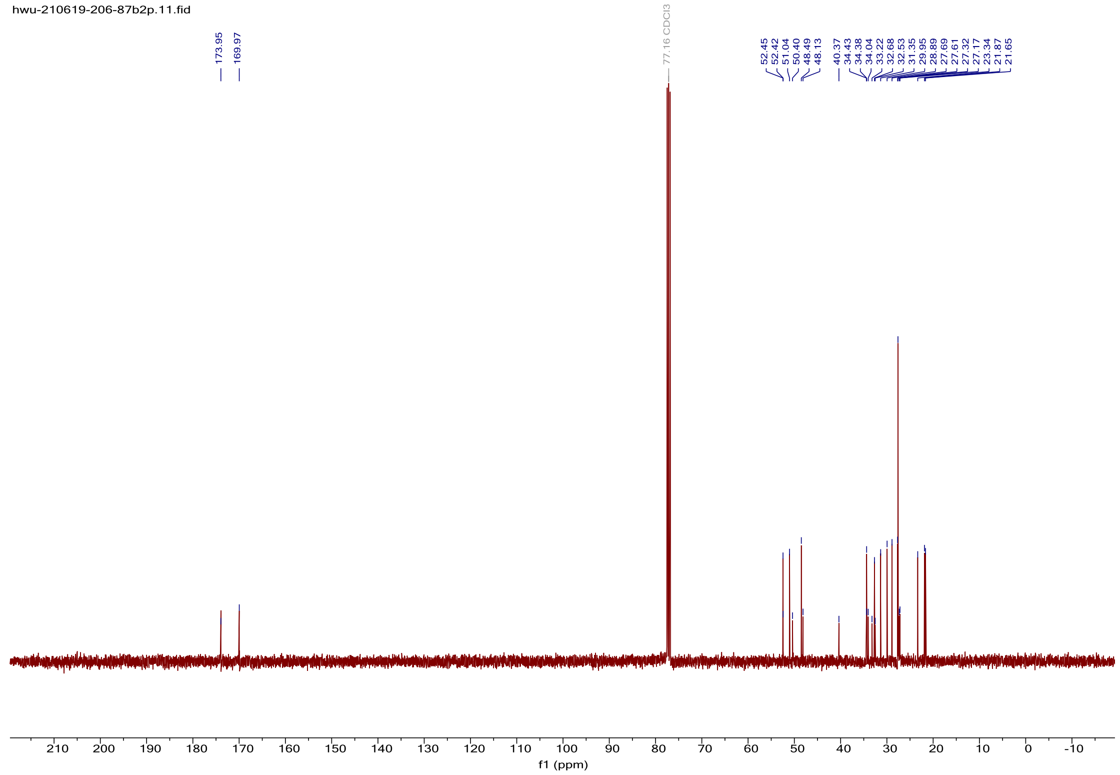
hwu-210619-206-87b2p.11.fid
173.95
169.97
77.16 CDCl3
52.45
52.42
51.04
50.40
48.49
48.13
40.37
34.43
34.38
34.04
33.22
32.68
32.53
31.35
29.95
28.89
27.69
27.61
27.32
27.17
23.34
21.87
21.65
f1 (ppm)

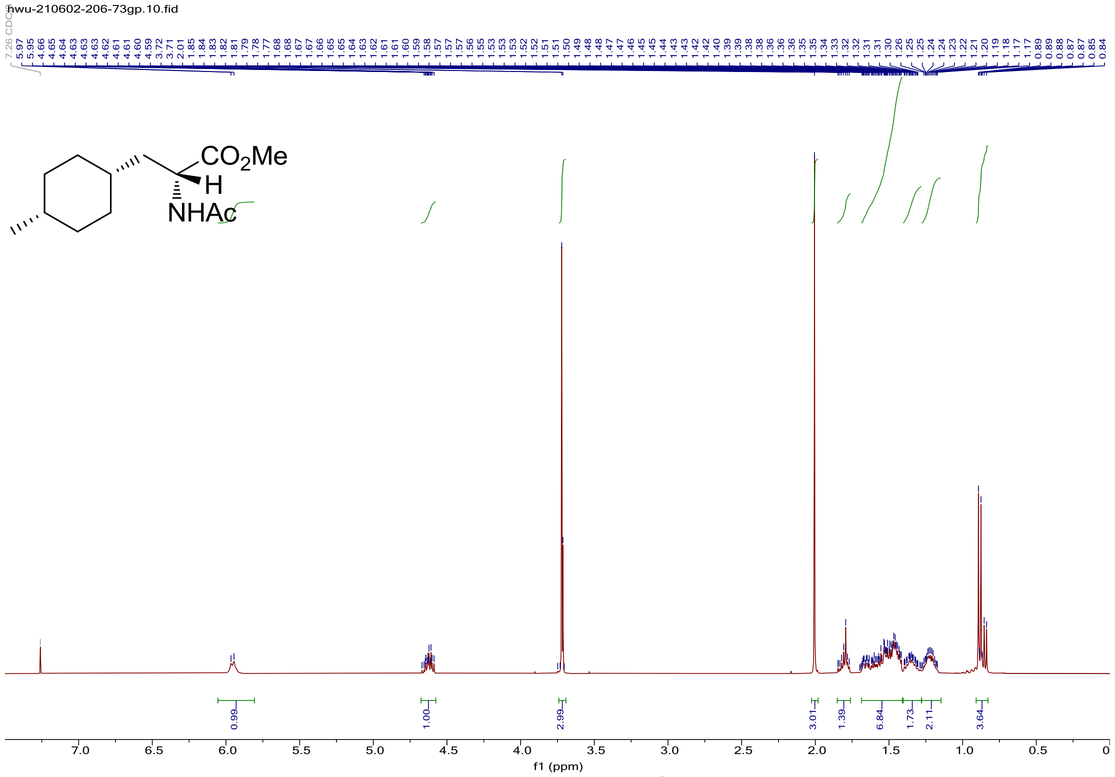
hwu-210602-206-73gp.10.fid
7.26 CDCl3
5.97 5.95 4.66 4.65 4.64 4.63 4.63 4.62 4.61 4.61 4.60 4.59 3.72 3.71 2.01 1.85 1.84 1.83 1.82 1.81 1.79 1.78 1.77 1.68 1.68 1.67 1.67 1.66 1.65 1.65 1.64 1.63 1.62 1.61 1.61 1.60 1.59 1.58 1.57 1.57 1.57 1.56 1.55 1.53 1.53 1.52 1.52 1.51 1.51 1.50 1.49 1.48 1.48 1.47 1.46 1.45 1.45 1.44 1.43 1.43 1.42 1.40 1.39 1.39 1.38 1.38 1.36 1.36 1.36 1.35 1.34 1.33 1.32 1.32 1.31 1.31 1.30 1.26 1.25 1.25 1.24 1.24 1.23 1.22 1.21 1.20 1.19 1.18 1.17 1.17 0.89 0.89 0.88 0.87 0.85 0.84
CO2Me
H
NHAc
0.99
1.00
2.99
3.01
1.39
6.84
1.73
2.11
3.64
7.0 6.5 6.0 5.5 5.0 4.5 4.0 3.5 3.0 2.5 2.0 1.5 1.0 0.5 0
f1 (ppm)

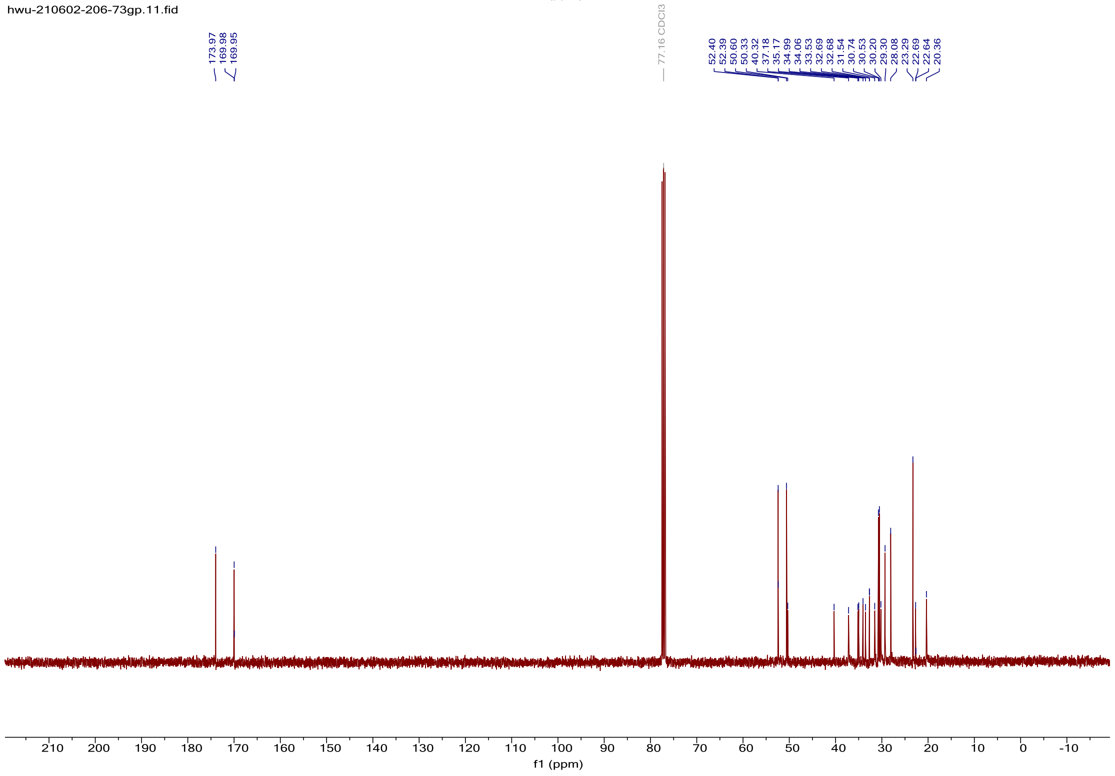
hwu-210602-206-73gp.11.fid
173.97 169.98 169.95
77.16 CDCl3
52.40 52.39 50.60 50.33 40.32 37.18 35.17 34.99 34.06 33.53 32.69 32.68 31.54 30.74 30.53 30.20 29.30 28.08 23.29 22.69 22.64 20.36
210 200 190 180 170 160 150 140 130 120 110 100 90 80 70 60 50 40 30 20 10 0 -10
f1 (ppm)

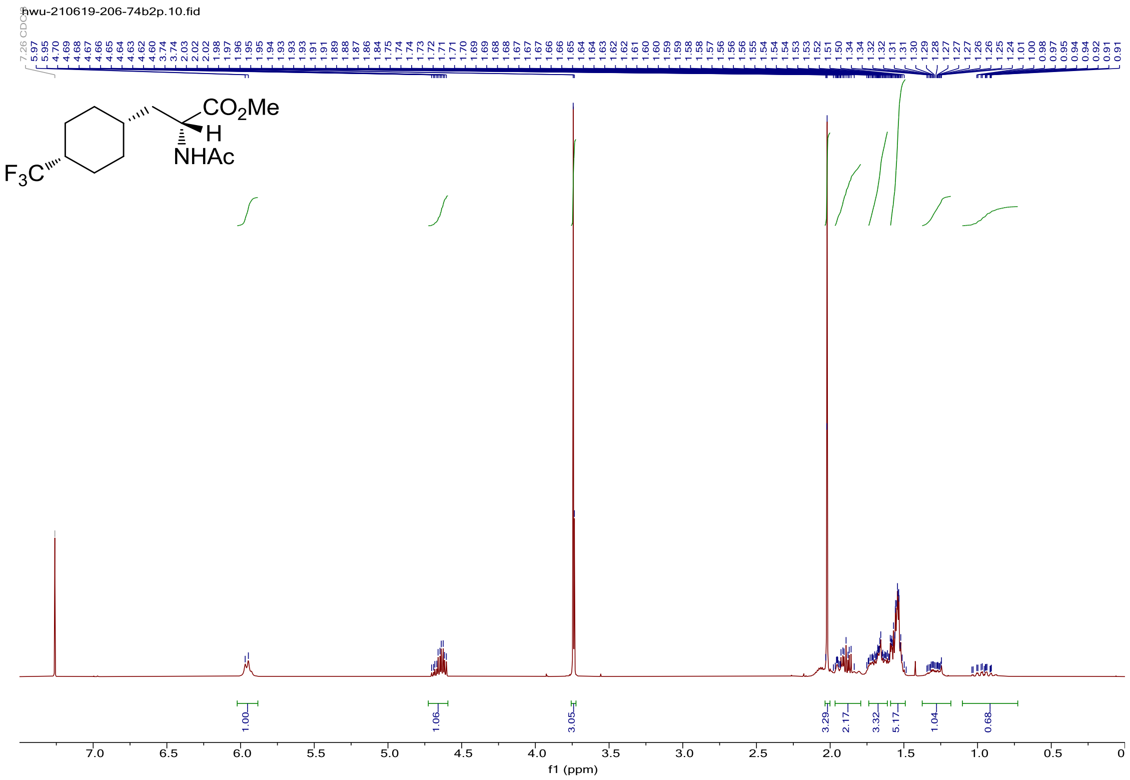
hwu-210619-206-74b2p.10.fid
7.26 CDCl3
5.97 5.95 4.70 4.69 4.68 4.67 4.66 4.65 4.64 4.63 4.62 4.60 3.74 3.74 2.03 2.02 2.02 1.98 1.97 1.96 1.95 1.95 1.94 1.93 1.93 1.93 1.91 1.91 1.89 1.88 1.87 1.86 1.84 1.75 1.74 1.74 1.73 1.72 1.71 1.71 1.70 1.69 1.69 1.68 1.68 1.67 1.67 1.67 1.66 1.66 1.65 1.64 1.64 1.63 1.63 1.62 1.62 1.61 1.60 1.60 1.59 1.59 1.58 1.58 1.57 1.56 1.56 1.56 1.55 1.54 1.54 1.54 1.53 1.53 1.52 1.51 1.50 1.34 1.34 1.32 1.32 1.31 1.31 1.30 1.29 1.28 1.27 1.27 1.27 1.26 1.25 1.24 1.01 1.00 0.98 0.97 0.95 0.94 0.94 0.92 0.91 0.91 0.91
CO2Me
H
NHAc
F3C
1.00
1.06
3.05
3.29
2.17
3.32
5.17
1.04
0.68
7.0 6.5 6.0 5.5 5.0 4.5 4.0 3.5 3.0 2.5 2.0 1.5 1.0 0.5 0
f1 (ppm)

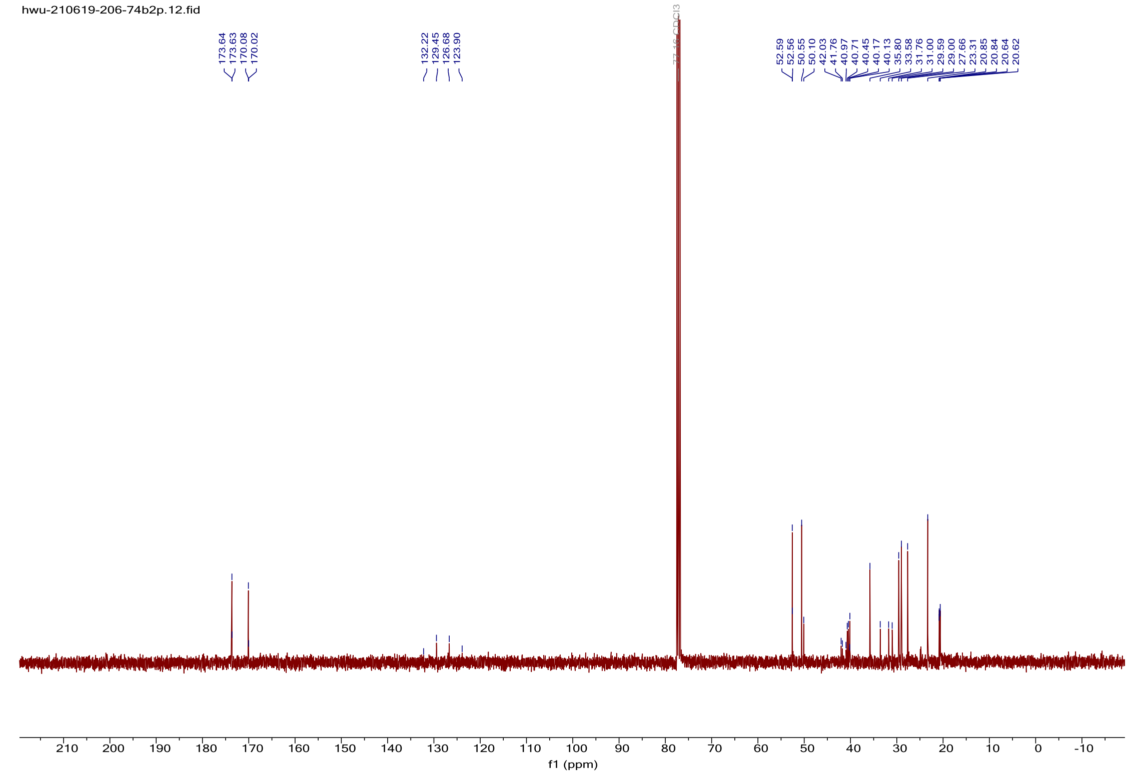
hwu-210619-206-74b2p.12.fid
173.64 173.63 170.08 170.02
132.22 129.45 126.68 123.90
CDCl3
52.59 52.56 50.55 50.10 42.03 41.76 40.97 40.71 40.45 40.17 40.13 35.80 33.58 31.76 31.00 29.59 29.00 27.66 23.31 20.85 20.84 20.64 20.62
210 200 190 180 170 160 150 140 130 120 110 100 90 80 70 60 50 40 30 20 10 0 -10
f1 (ppm)

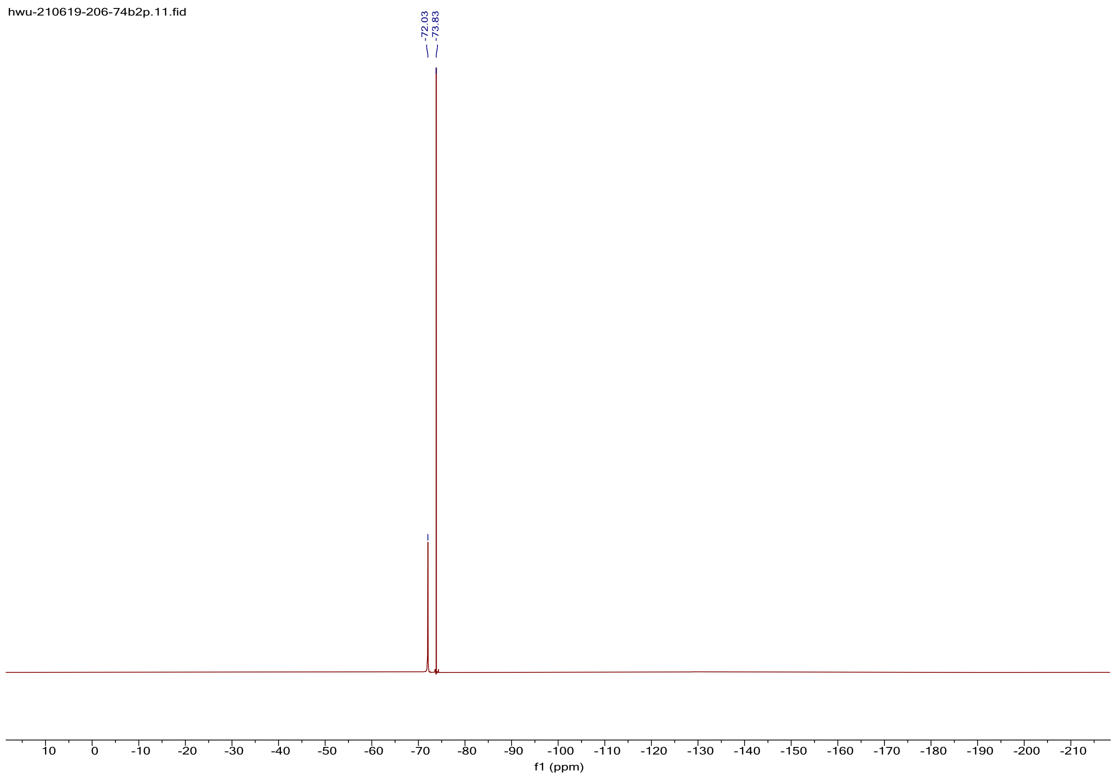
hwu-210619-206-74b2p.11.fid
-72.03
-73.83
10 0 -10 -20 -30 -40 -50 -60 -70 -80 -90 -100 -110 -120 -130 -140 -150 -160 -170 -180 -190 -200 -210
f1 (ppm)

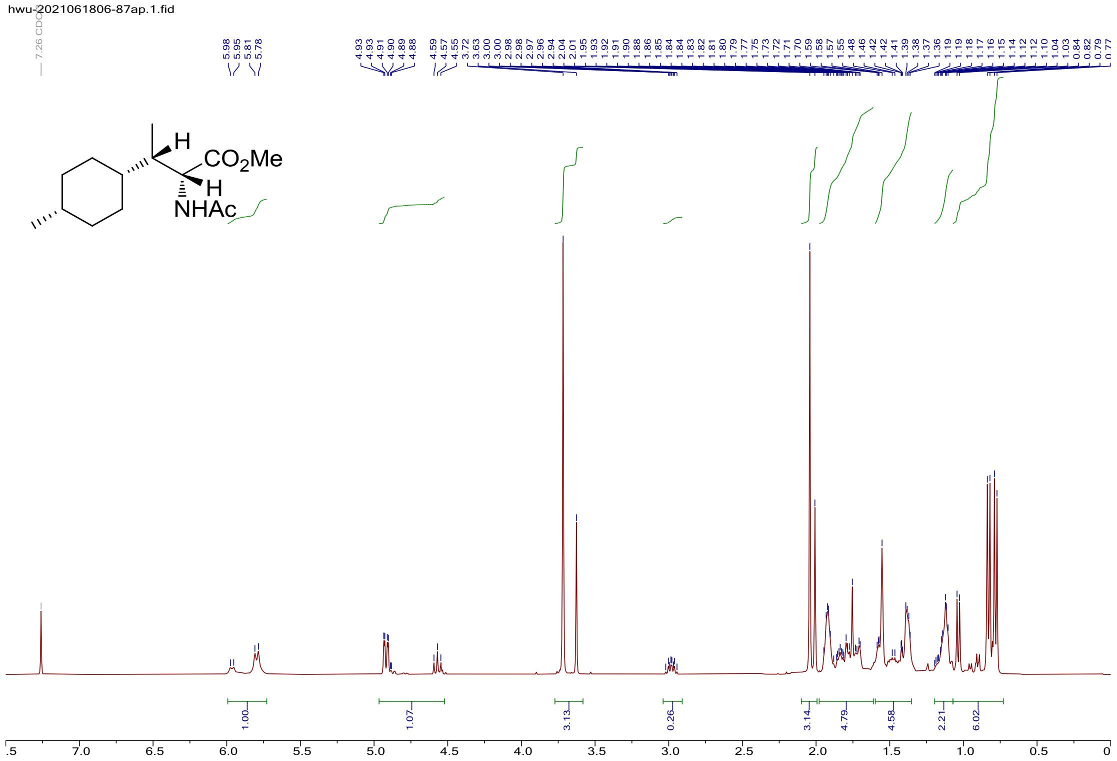
hwu-2021061806-87ap.1.fid
7.26 CDCl3
5.98 5.95 5.81 5.78
4.93 4.93 4.91 4.90 4.89 4.88
4.59 4.57 4.55
3.72 3.63 3.00 3.00 2.98 2.98 2.97 2.96 2.94 2.04 2.01 1.95 1.93 1.93 1.92 1.91 1.90 1.88 1.86 1.85 1.84 1.84 1.83 1.82 1.81 1.80 1.79 1.77 1.75 1.73 1.72 1.71 1.70 1.59 1.58 1.57 1.55 1.48 1.46 1.42 1.42 1.41 1.39 1.38 1.37 1.36 1.19 1.19 1.18 1.17 1.16 1.15 1.14 1.12 1.12 1.10 1.04 1.03 0.84 0.82 0.79 0.77
H
CO2Me
H
NHAc
1.00
1.07
3.13
0.26
3.14
4.79
4.58
2.21
6.02
f1 (ppm)

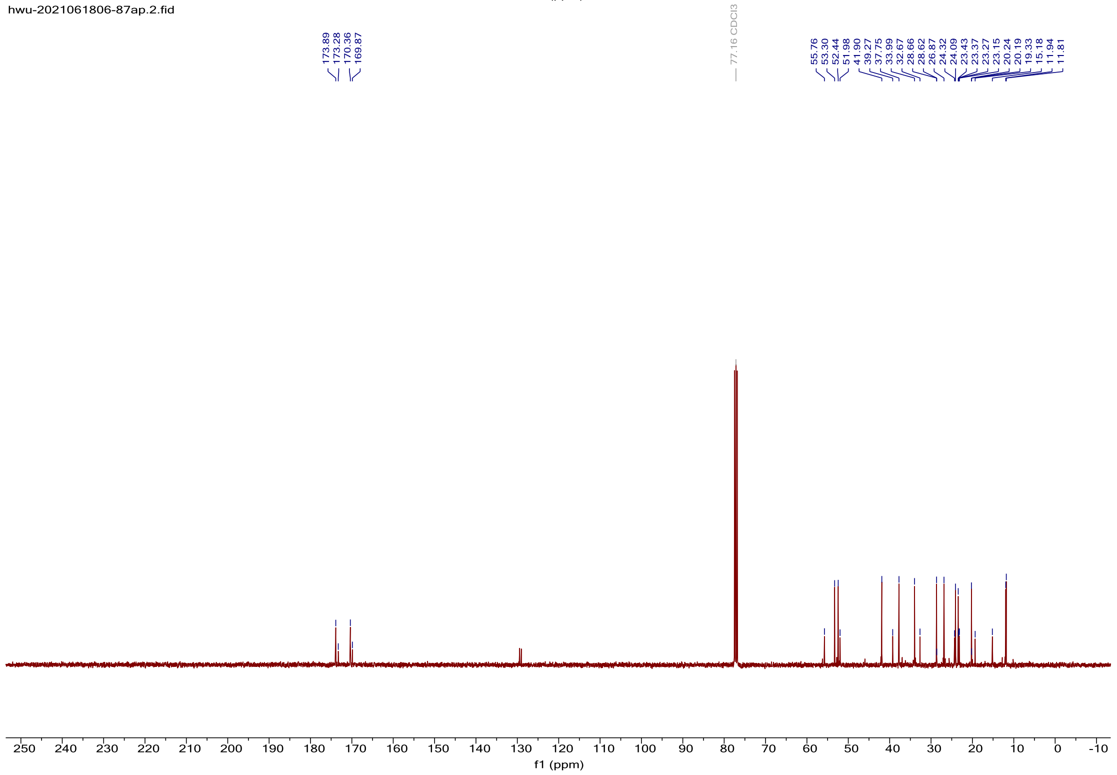
hwu-2021061806-87ap.2.fid
173.89 173.28 170.36 169.87
77.16 CDCl3
55.76 53.30 52.44 51.98 41.90 39.27 37.75 33.99 32.67 28.66 28.62 26.87 24.32 24.09 23.43 23.37 23.27 23.15 20.24 20.19 19.33 15.18 11.94 11.81
f1 (ppm)

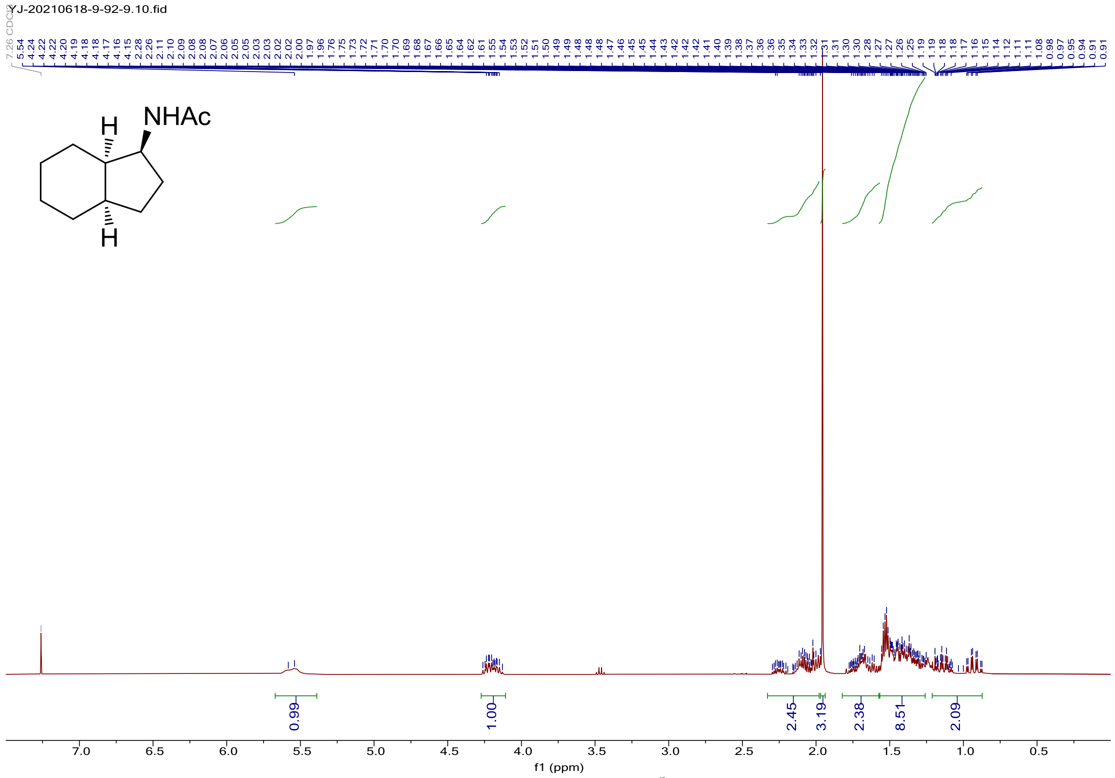
YJ-20210618-9-92-9.10.fid
7.26 CDCl3
5.54 4.24 4.22 4.20 4.19 4.18 4.18 4.17 4.16 4.15 2.28 2.26 2.11 2.10 2.09 2.08 2.08 2.07 2.06 2.05 2.05 2.03 2.03 2.02 2.02 2.00 1.97 1.96 1.76 1.75 1.73 1.71 1.70 1.70 1.69 1.68 1.67 1.66 1.65 1.64 1.62 1.61 1.55 1.54 1.53 1.52 1.51 1.50 1.49 1.49 1.48 1.48 1.48 1.47 1.46 1.45 1.45 1.44 1.43 1.42 1.42 1.42 1.41 1.40 1.39 1.38 1.37 1.36 1.36 1.35 1.34 1.33 1.32 1.31 1.31 1.30 1.28 1.27 1.27 1.26 1.25 1.19 1.19 1.18 1.18 1.17 1.16 1.15 1.14 1.12 1.11 1.11 1.08 0.98 0.97 0.95 0.94 0.91 0.91
NHAc
H
H
0.99
1.00
2.45
3.19
2.38
8.51
2.09
f1 (ppm)

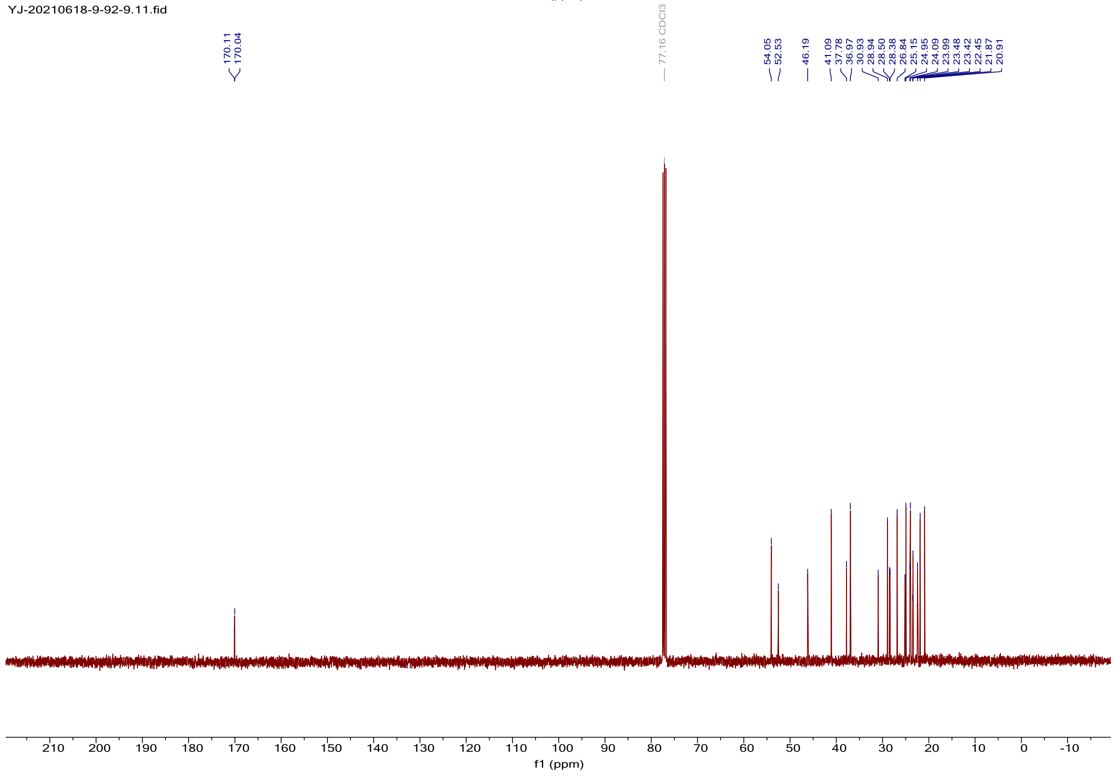
YJ-20210618-9-92-9.11.fid
170.11 170.04
77.16 CDCl3
54.05 52.53 46.19 41.09 37.78 36.97 30.93 28.94 28.50 28.38 26.84 25.15 24.95 24.09 23.99 23.48 23.42 22.45 21.87 20.91
f1 (ppm)

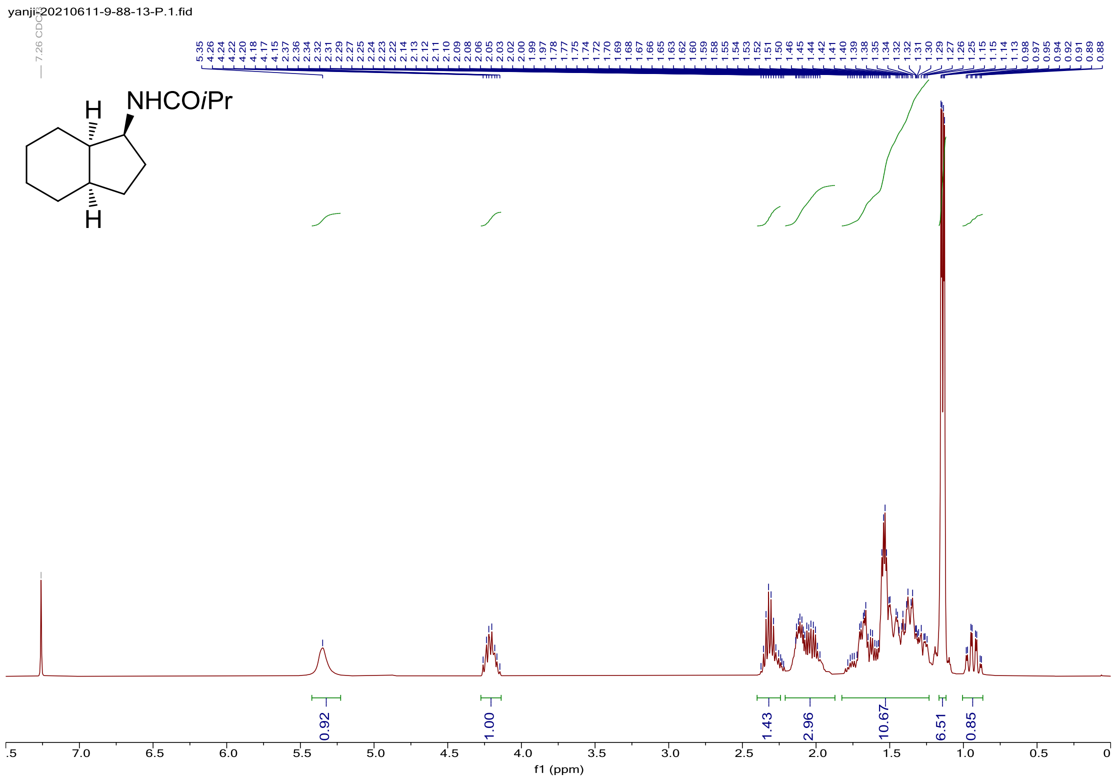
yanji-20210611-9-88-13-P.1.fid
7.26 CDCl3
5.35 4.26 4.24 4.22 4.20 4.18 4.17 4.15 2.37 2.36 2.34 2.32 2.31 2.29 2.27 2.25 2.24 2.23 2.22 2.14 2.13 2.12 2.11 2.10 2.09 2.08 2.06 2.05 2.03 2.02 2.00 1.99 1.97 1.78 1.77 1.75 1.74 1.72 1.70 1.69 1.68 1.67 1.66 1.65 1.63 1.62 1.60 1.59 1.58 1.55 1.54 1.53 1.52 1.51 1.50 1.46 1.45 1.44 1.42 1.41 1.40 1.39 1.38 1.35 1.34 1.32 1.32 1.31 1.30 1.29 1.27 1.26 1.25 1.15 1.15 1.14 1.13 0.98 0.97 0.95 0.94 0.92 0.91 0.89 0.88
H
NHCOiPr
H
0.92
1.00
1.43
2.96
10.67
6.51
0.85
f1 (ppm)

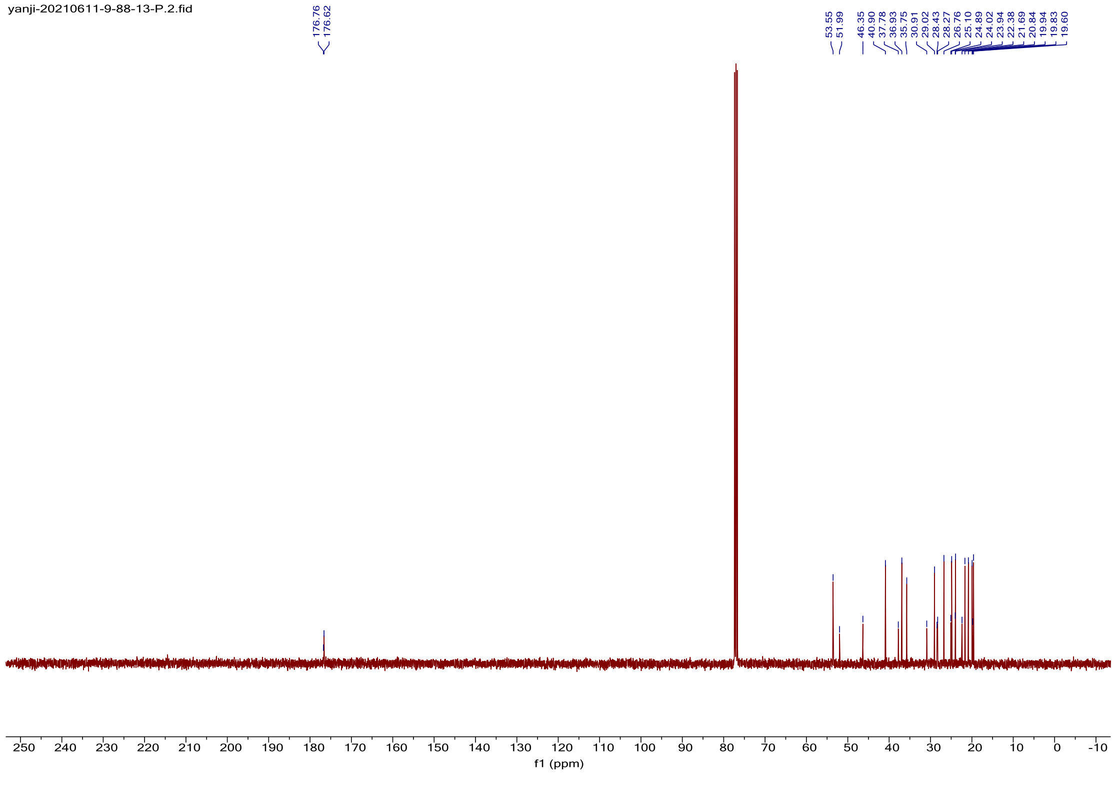
yanji-20210611-9-88-13-P.2.fid
176.76 176.62
53.55 51.99 46.35 40.90 37.78 36.93 35.75 30.91 29.02 28.43 28.27 26.76 25.10 24.89 24.02 23.94 22.38 21.69 20.84 19.94 19.83 19.60
f1 (ppm)

YJ-20210611-9-88-12-P.10.fid

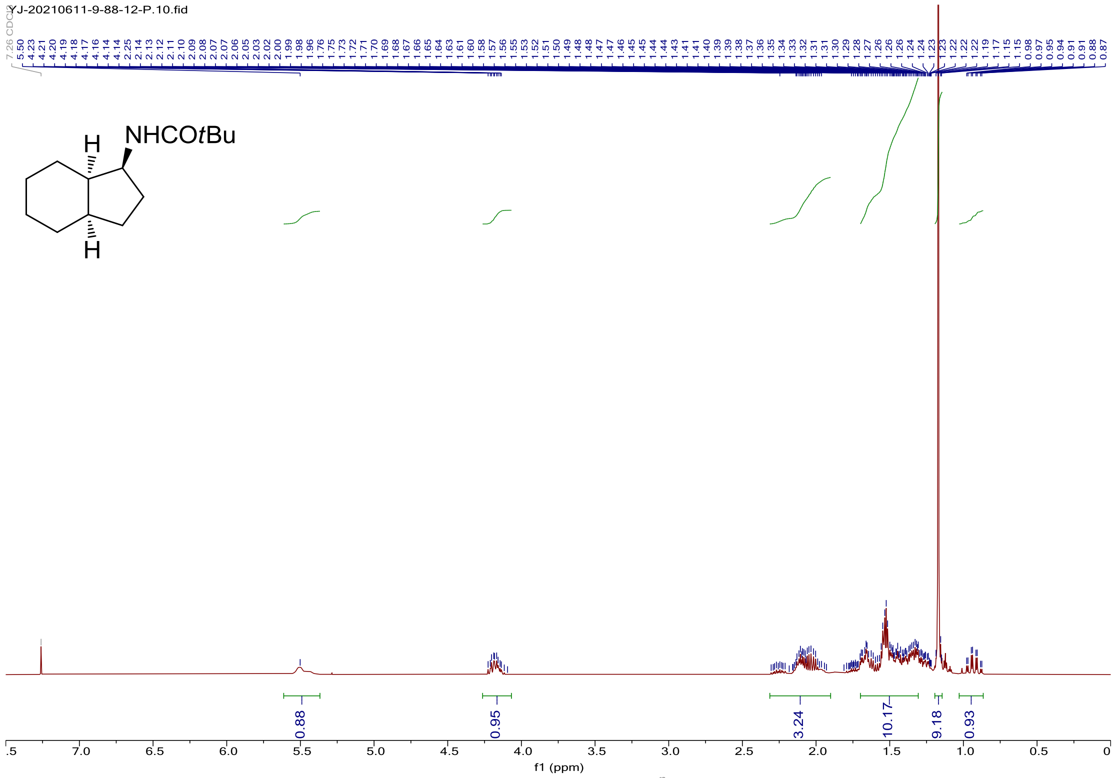

YJ-20210611-9-88-12-P.11.fid

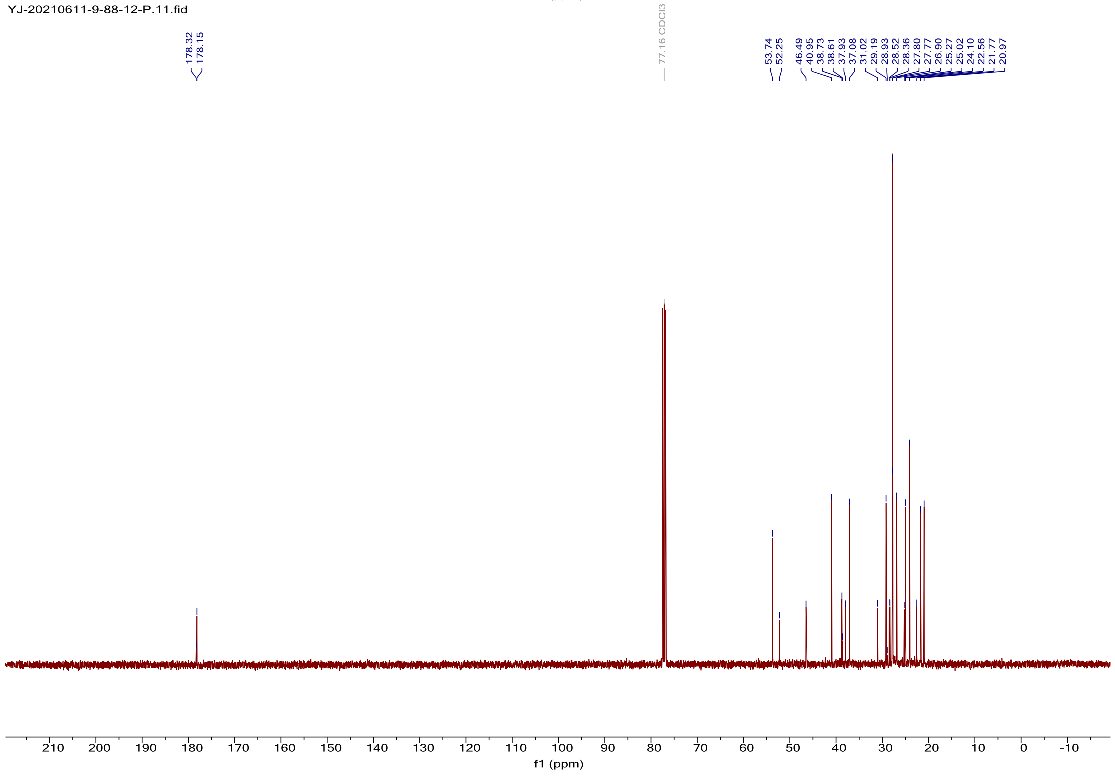

hwu-210614-206-63cp.10.fid

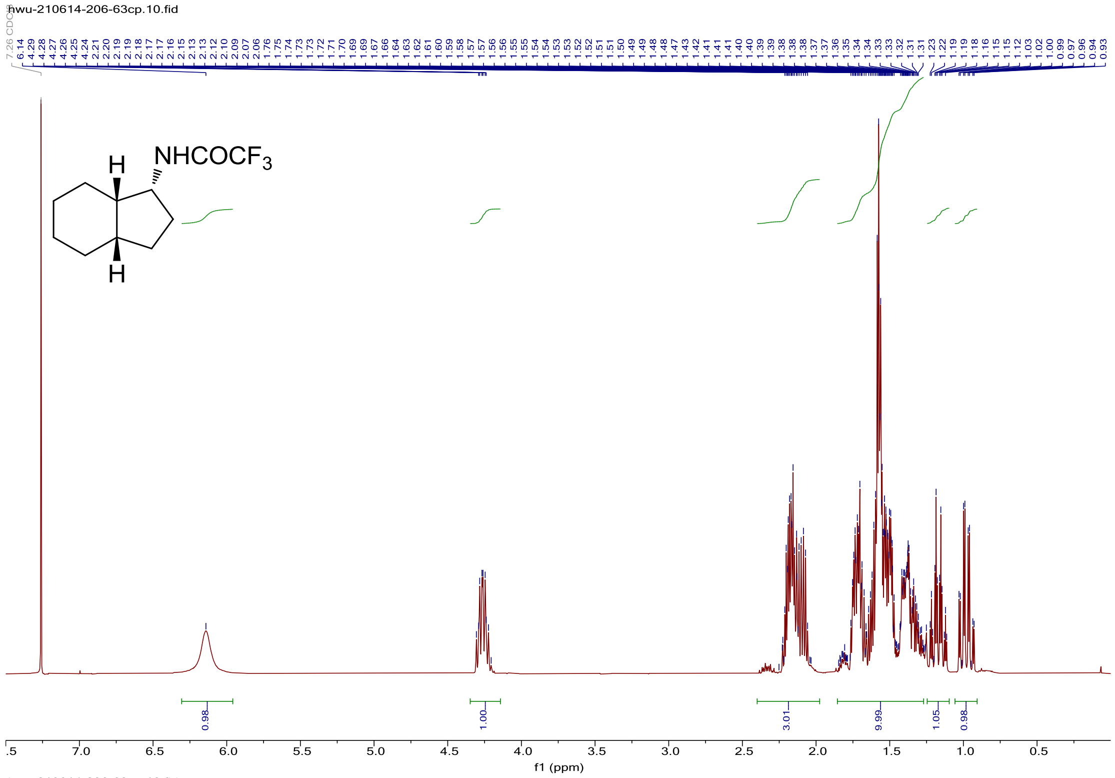


hwu-210614-206-63cp.12.fid

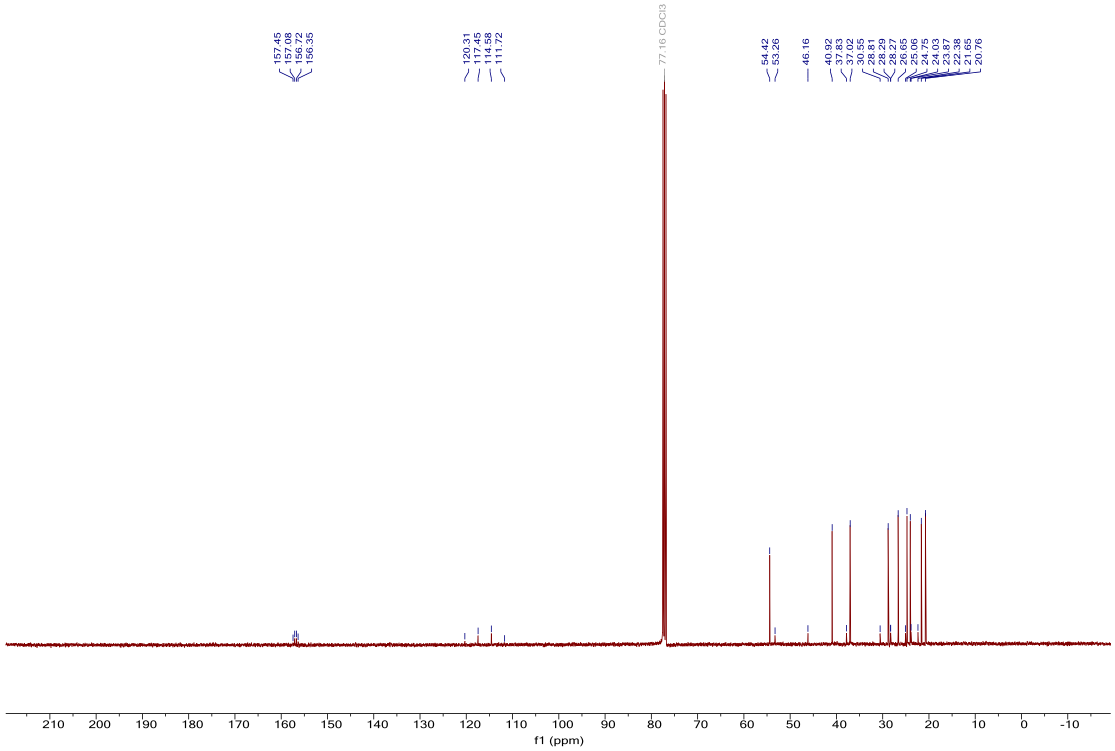

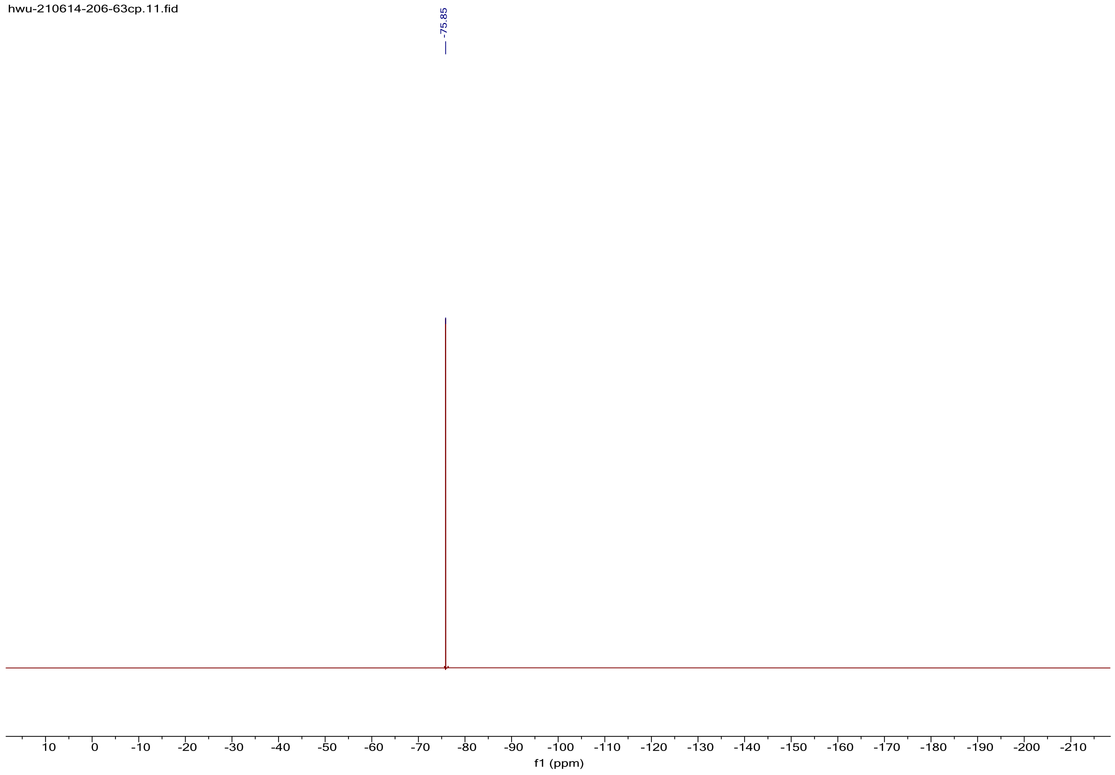
hwu-210614-206-63cp.11.fid
-75.85
10 0 -10 -20 -30 -40 -50 -60 -70 -80 -90 -100 -110 -120 -130 -140 -150 -160 -170 -180 -190 -200 -210
f1 (ppm)

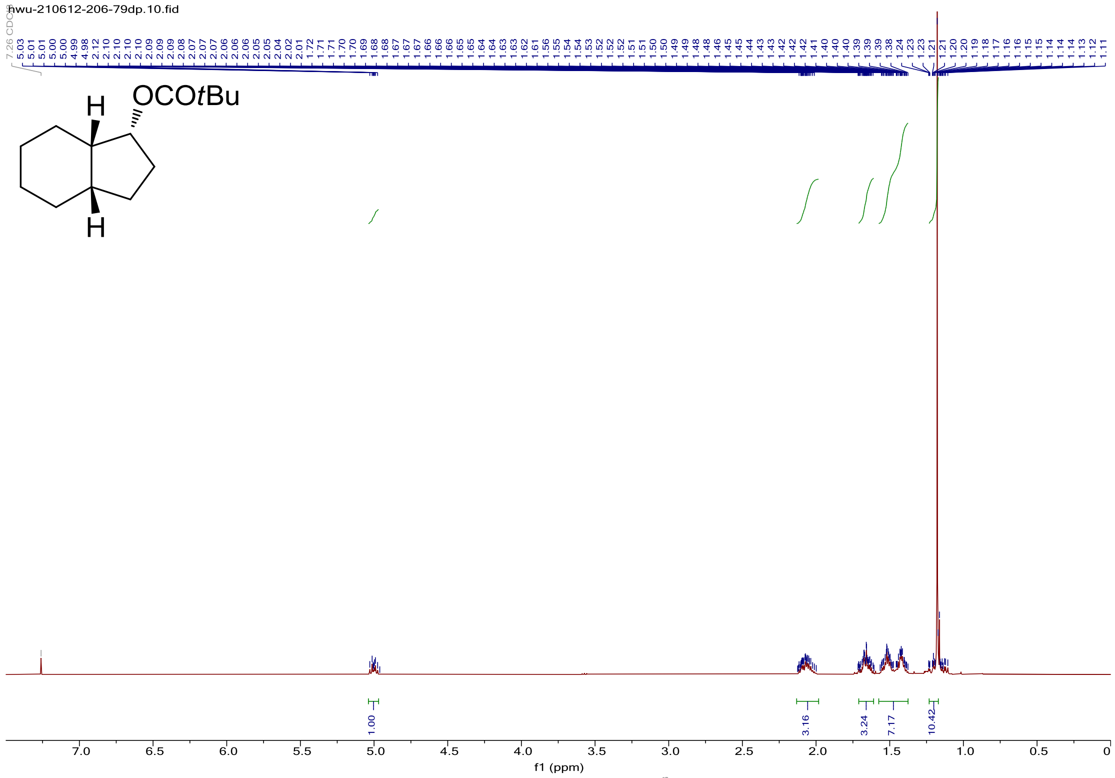
hwu-210612-206-79dp.10.fid
OCOtBu
H
H
f1 (ppm)

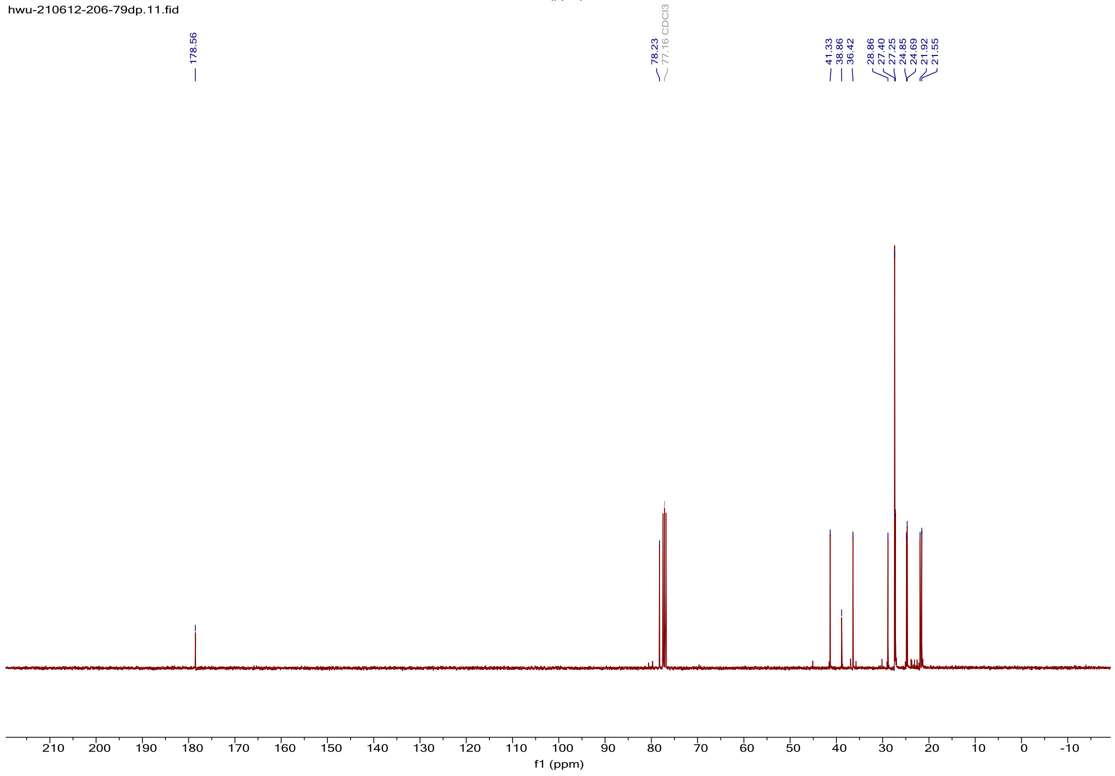
hwu-210612-206-79dp.11.fid
178.56
78.23
77.16 CDCl3
41.33
38.86
36.42
28.86
27.40
27.25
24.85
24.69
21.92
21.55
f1 (ppm)

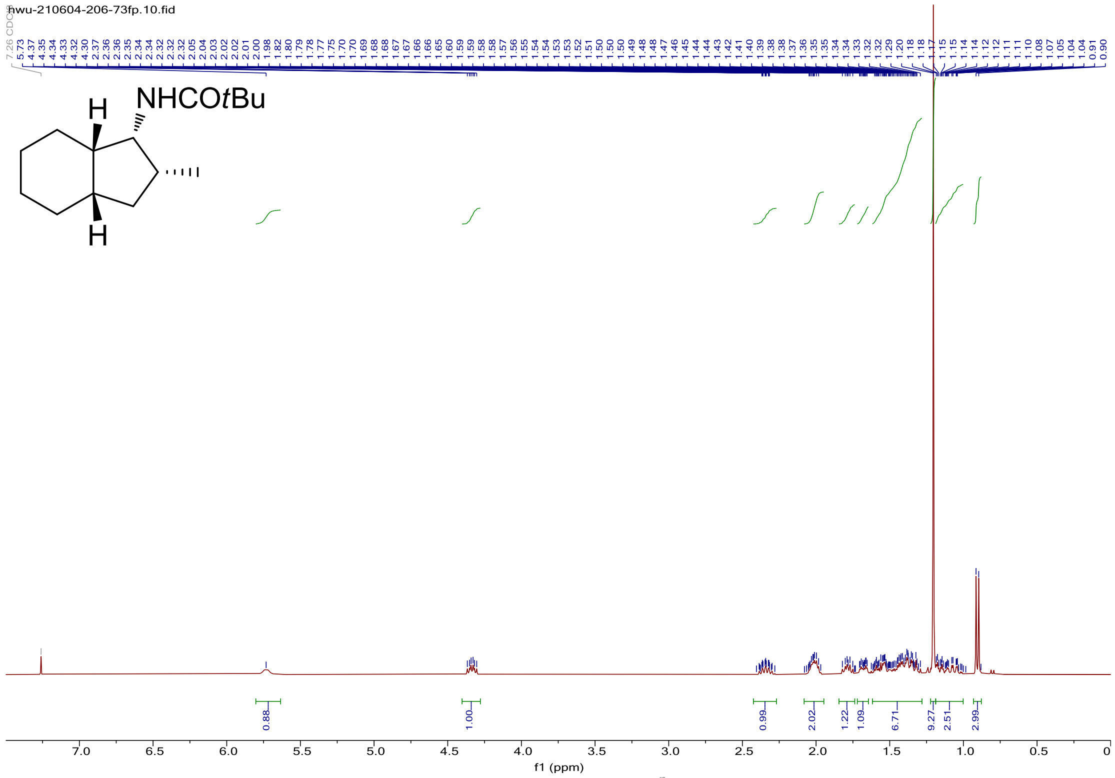
hwu-210604-206-73fp.10.fid
7.26 CDCl3
5.73 4.37 4.35 4.34 4.33 4.32 4.30 2.37 2.36 2.35 2.34 2.34 2.32 2.32 2.05 2.04 2.03 2.02 2.02 2.01 2.00 1.98 1.82 1.80 1.79 1.78 1.77 1.75 1.70 1.69 1.68 1.68 1.67 1.67 1.66 1.65 1.60 1.59 1.59 1.58 1.58 1.57 1.56 1.55 1.54 1.54 1.53 1.53 1.52 1.51 1.50 1.50 1.50 1.49 1.48 1.48 1.47 1.46 1.45 1.44 1.43 1.42 1.41 1.40 1.39 1.38 1.38 1.37 1.36 1.35 1.35 1.34 1.34 1.33 1.32 1.32 1.29 1.20 1.18 1.17 1.15 1.15 1.14 1.14 1.12 1.12 1.11 1.10 1.08 1.07 1.05 1.04 0.91 0.90
NHCOtBu
H
H
0.88 1.00 0.99 2.02 1.22 1.09 6.71 9.27 2.51 2.99
7.0 6.5 6.0 5.5 5.0 4.5 4.0 3.5 3.0 2.5 2.0 1.5 1.0 0.5 0
f1 (ppm)

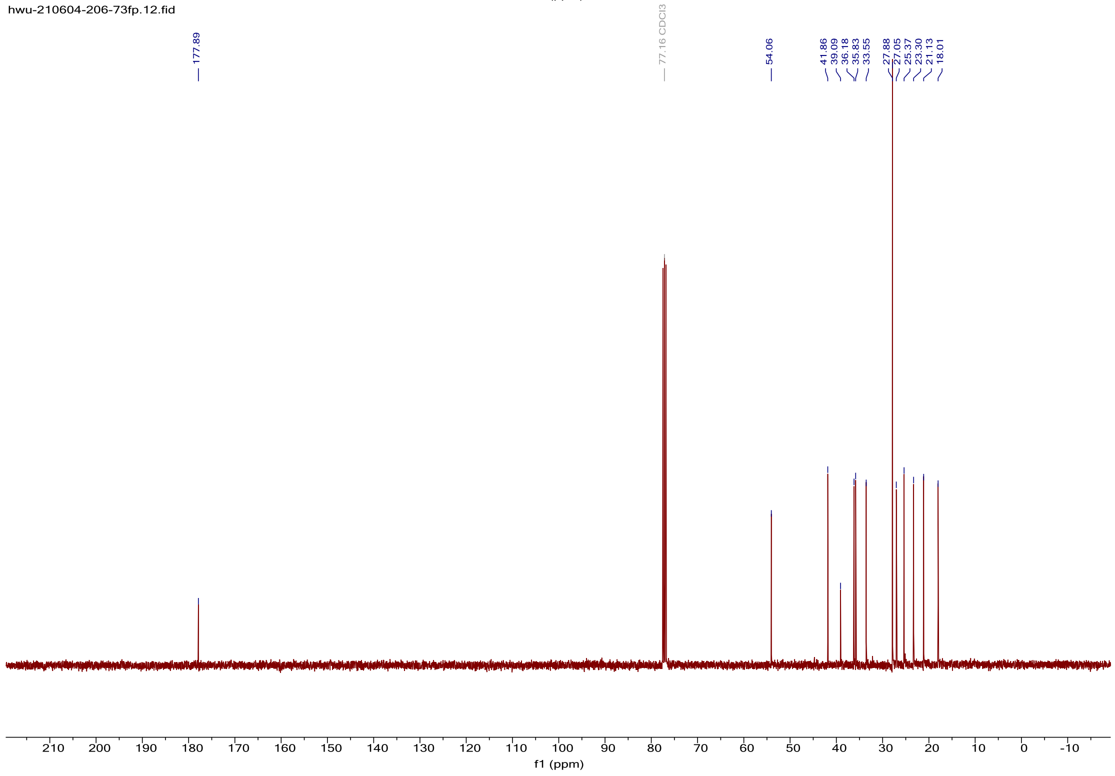
hwu-210604-206-73fp.12.fid
177.89
77.16 CDCl3
54.06
41.86 39.09 36.18 35.83 33.55
27.88 27.05 25.37 23.30 21.13 18.01
210 200 190 180 170 160 150 140 130 120 110 100 90 80 70 60 50 40 30 20 10 0 -10
f1 (ppm)

hwu-2021060406-73dp.1.fid

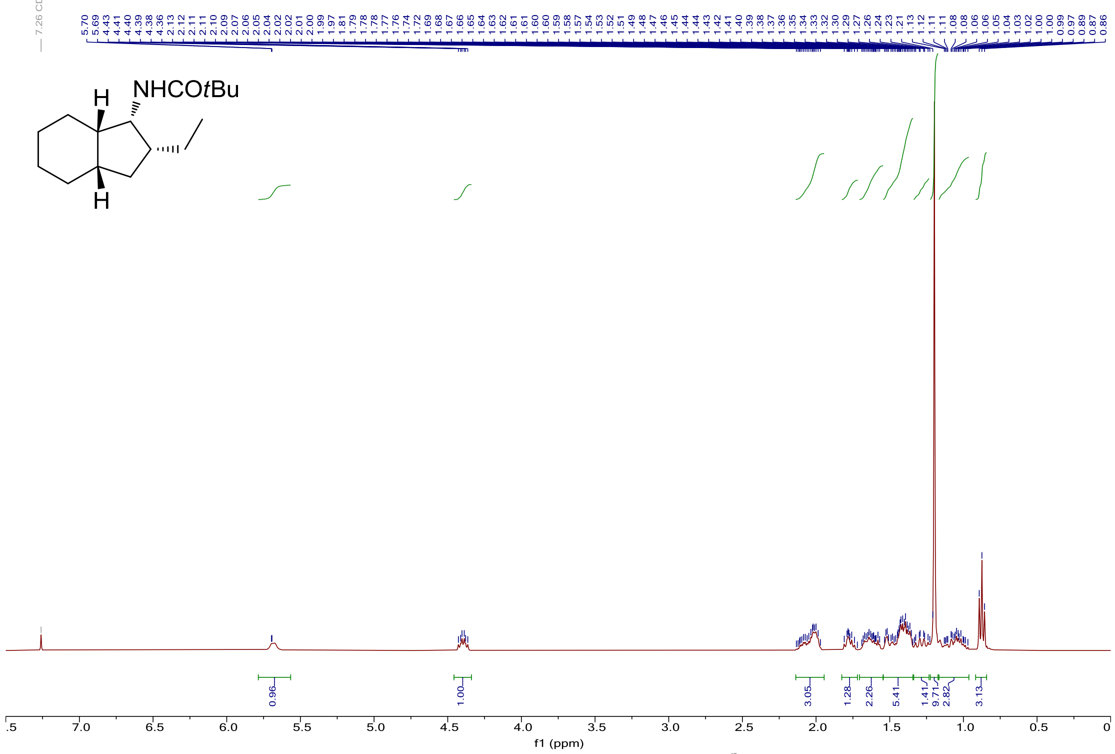


hwu-2021060406-73dp.2.fid

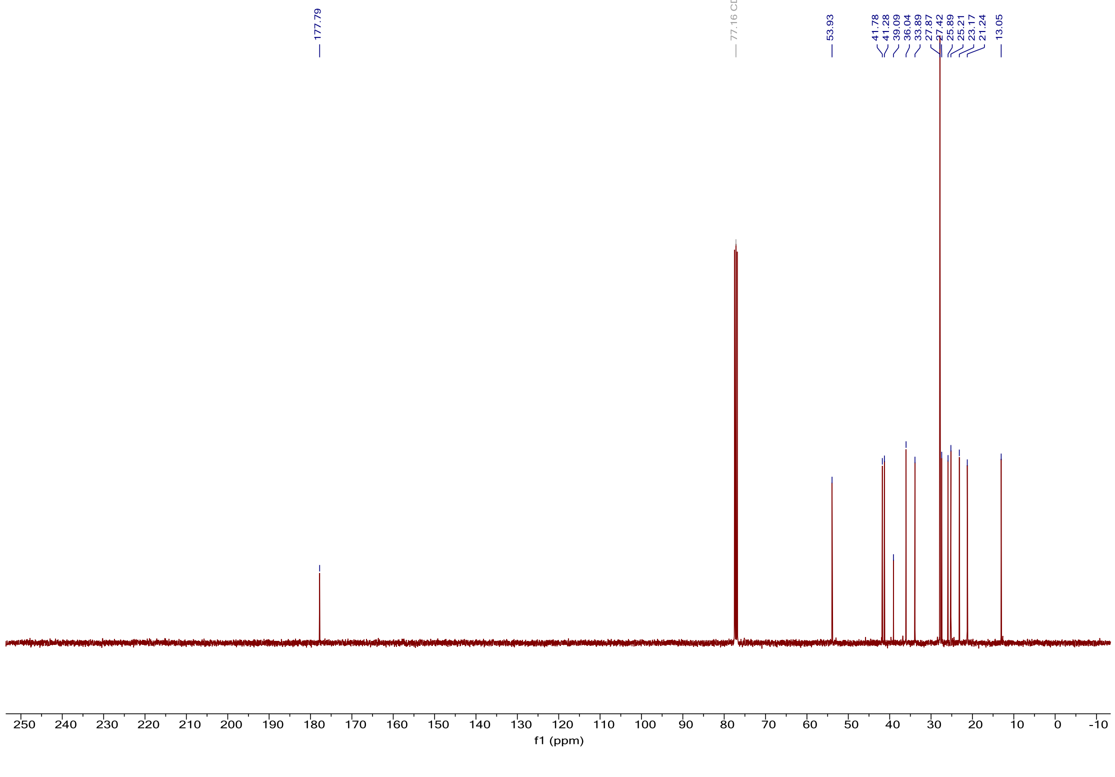

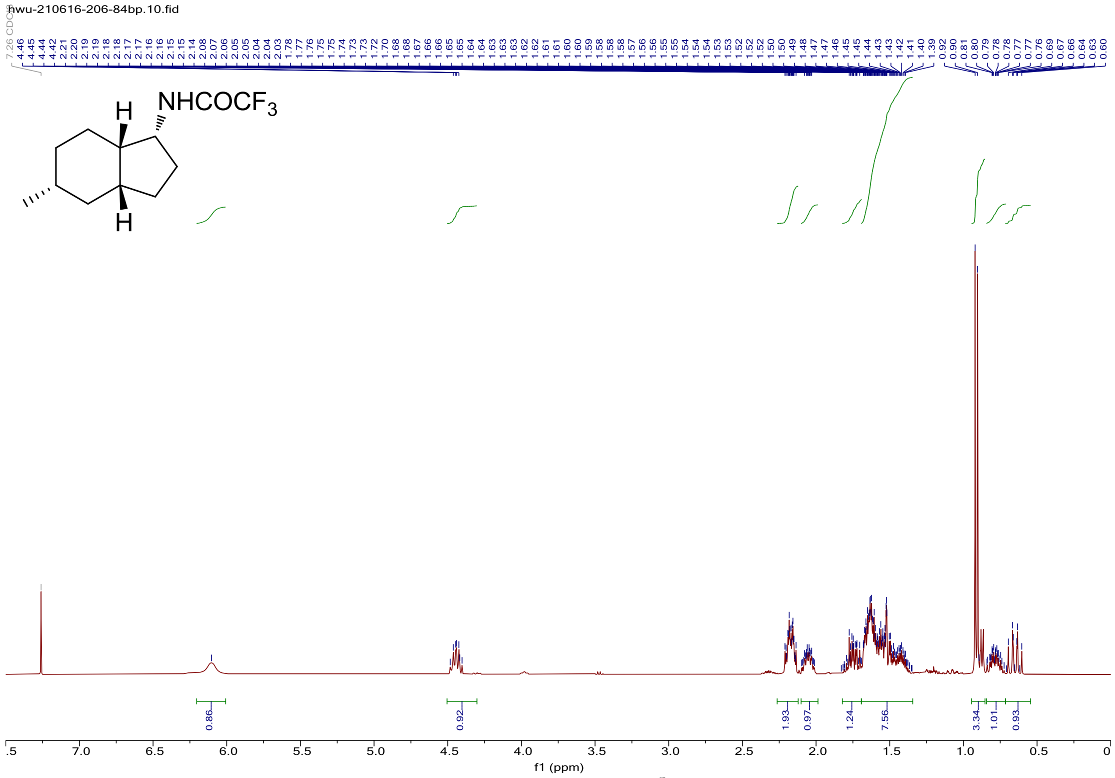
hwu-210616-206-84bp.10.fid
NHCOCF3
H
H
f1 (ppm)

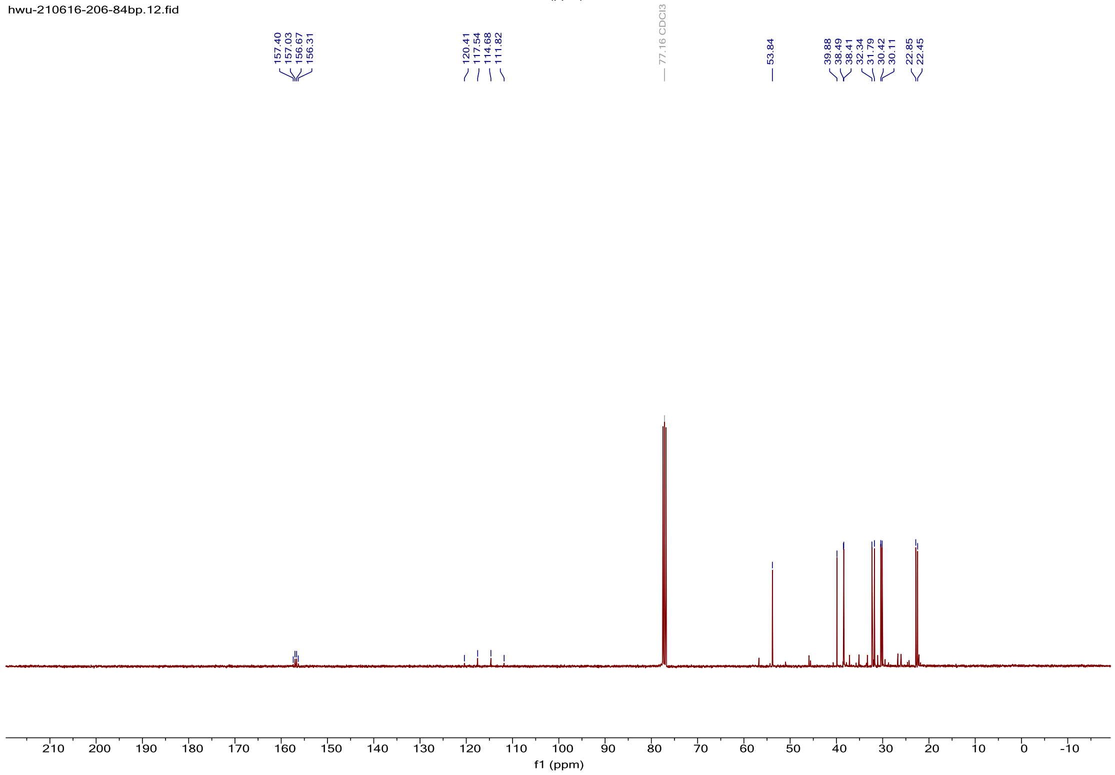
hwu-210616-206-84bp.12.fid
157.40
157.03
156.67
156.31
120.41
117.54
114.68
111.82
77.16 CDCl3
53.84
39.88
38.49
38.41
32.34
31.79
30.42
30.11
22.85
22.45
f1 (ppm)

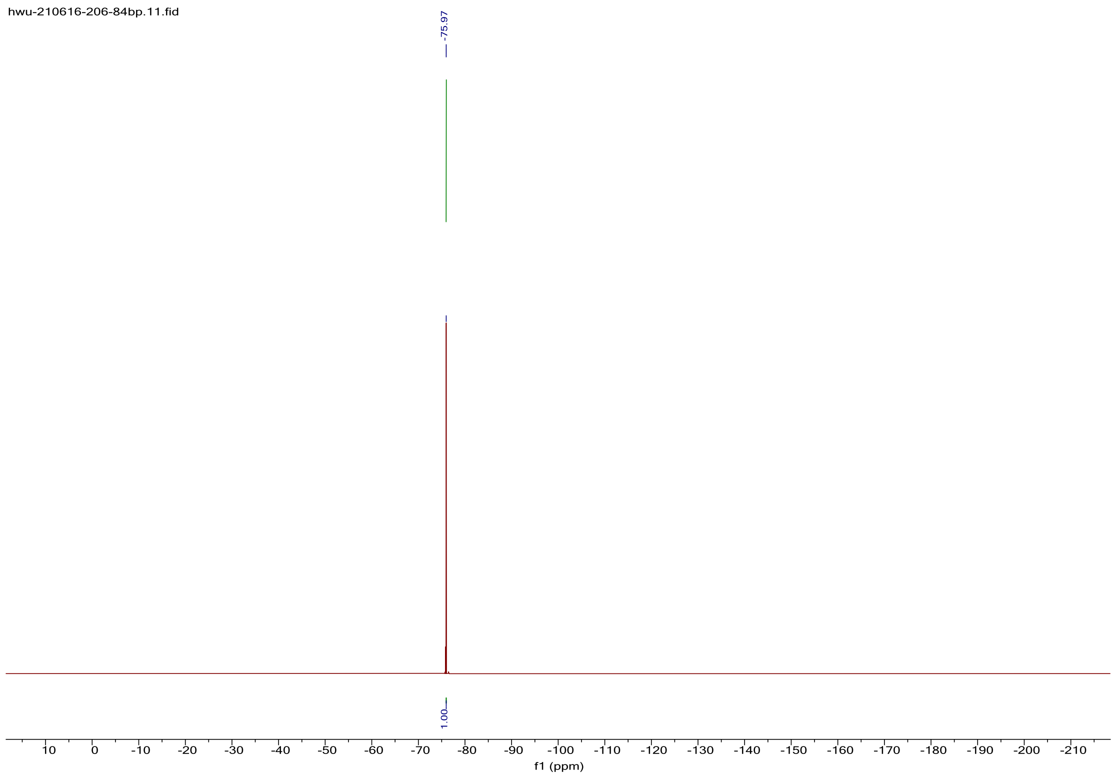
hwu-210616-206-84bp.11.fid
-75.97
1.00
10 0 -10 -20 -30 -40 -50 -60 -70 -80 -90 -100 -110 -120 -130 -140 -150 -160 -170 -180 -190 -200 -210
f1 (ppm)

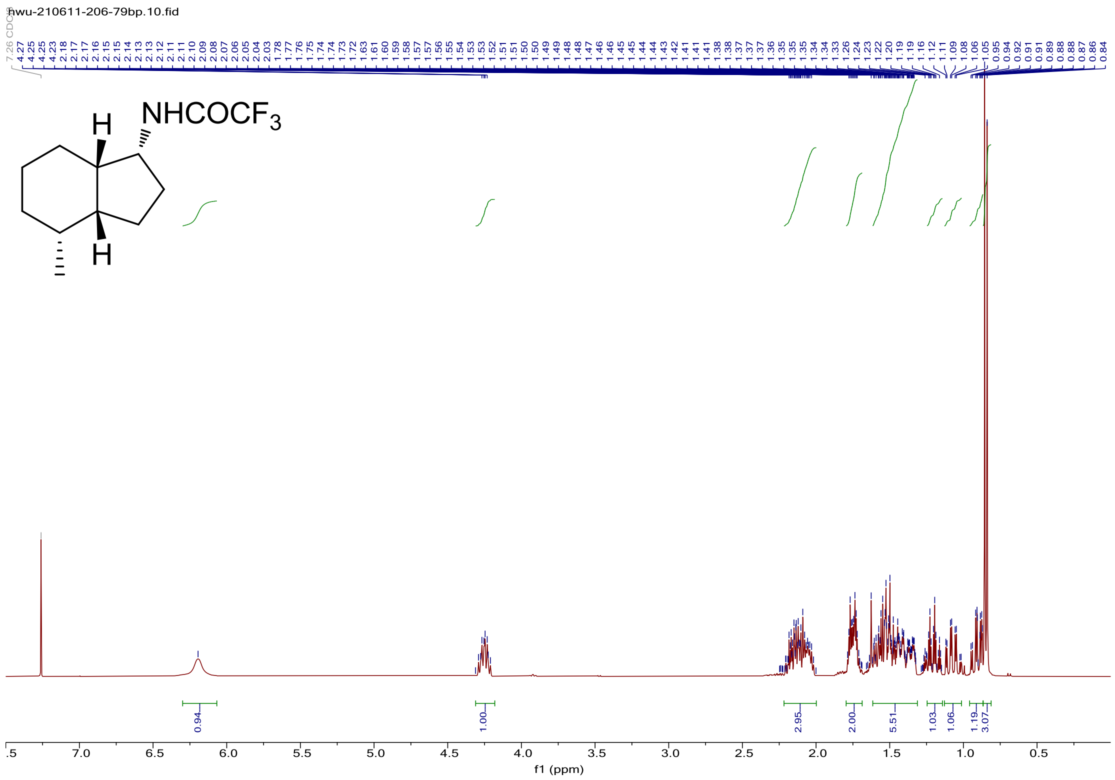

hwu-210611-206-79bp.10.fid
7.26 CDCl3
4.27
4.25
4.25
4.23
2.18
2.17
2.17
2.16
2.15
2.15
2.14
2.13
2.13
2.12
2.11
2.11
2.10
2.09
2.08
2.07
2.06
2.05
2.04
2.03
1.78
1.77
1.76
1.75
1.74
1.74
1.73
1.72
1.63
1.61
1.60
1.59
1.58
1.57
1.57
1.56
1.55
1.54
1.53
1.53
1.52
1.51
1.51
1.50
1.50
1.49
1.49
1.48
1.48
1.47
1.46
1.46
1.45
1.45
1.44
1.44
1.43
1.42
1.41
1.41
1.41
1.38
1.38
1.37
1.37
1.37
1.36
1.35
1.35
1.35
1.34
1.34
1.33
1.26
1.24
1.23
1.22
1.20
1.19
1.19
1.16
1.12
1.11
1.09
1.08
1.06
1.05
0.95
0.94
0.92
0.91
0.91
0.89
0.88
0.88
0.87
0.86
0.84
H
NHCOCF3
H
0.94
1.00
2.95
2.00
5.51
1.03
1.06
1.19
3.07
.5
7.0
6.5
6.0
5.5
5.0
4.5
4.0
3.5
3.0
2.5
2.0
1.5
1.0
0.5
f1 (ppm)


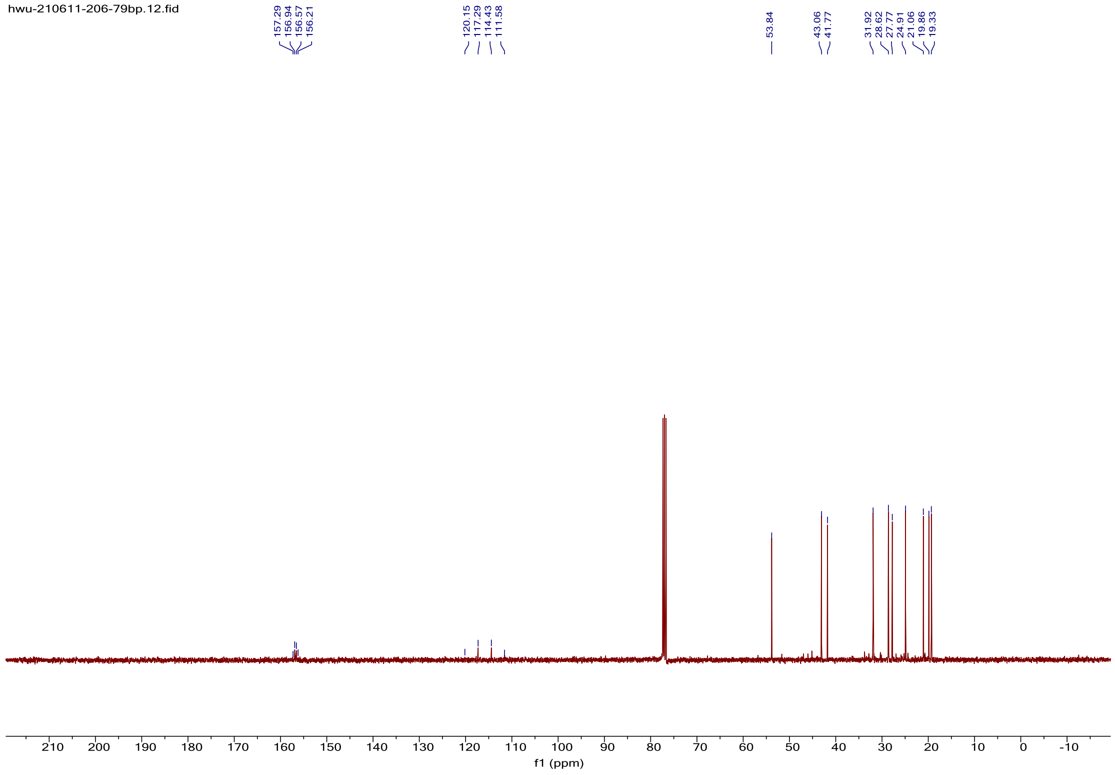

hwu-210611-206-79bp.12.fid
157.29
156.94
156.57
156.21
120.15
117.29
114.43
111.58
53.84
43.06
41.77
31.92
28.62
27.77
24.91
21.06
19.86
19.33
210
200
190
180
170
160
150
140
130
120
110
100
90
80
70
60
50
40
30
20
10
0
-10
f1 (ppm)

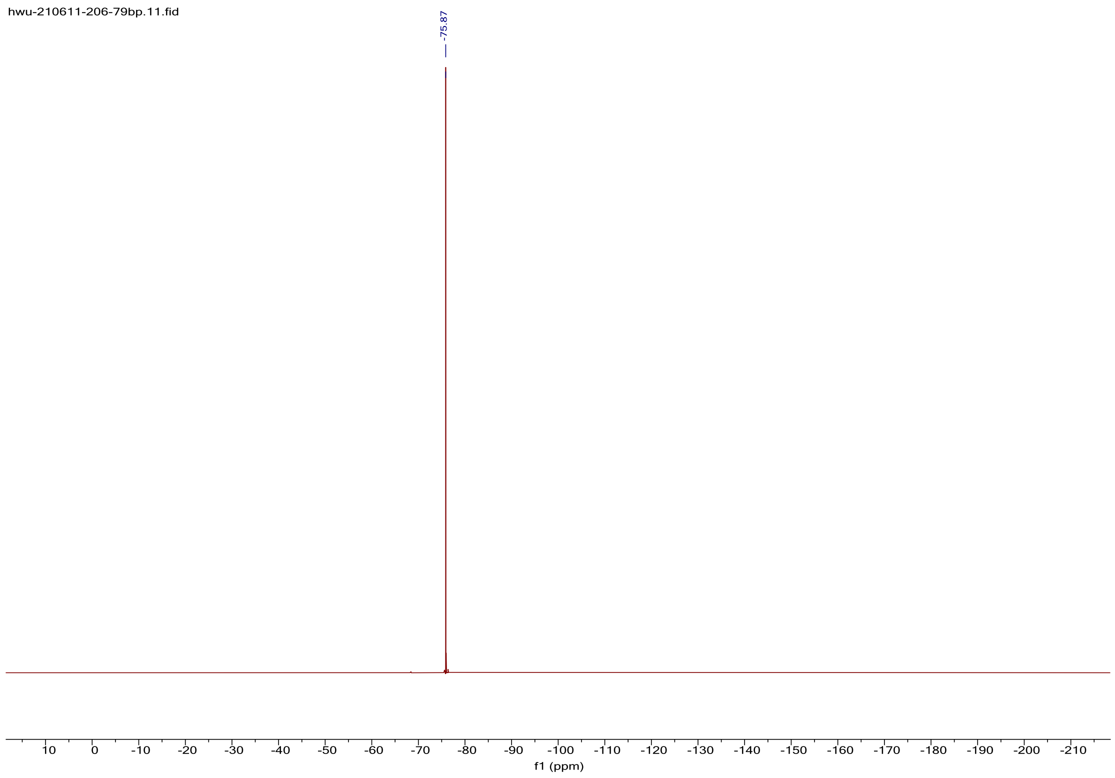
hwu-210611-206-79bp.11.fid
-75.87
10
0
-10
-20
-30
-40
-50
-60
-70
-80
-90
-100
-110
-120
-130
-140
-150
-160
-170
-180
-190
-200
-210
f1 (ppm)

hwu-210616-206-84cp.10.fid

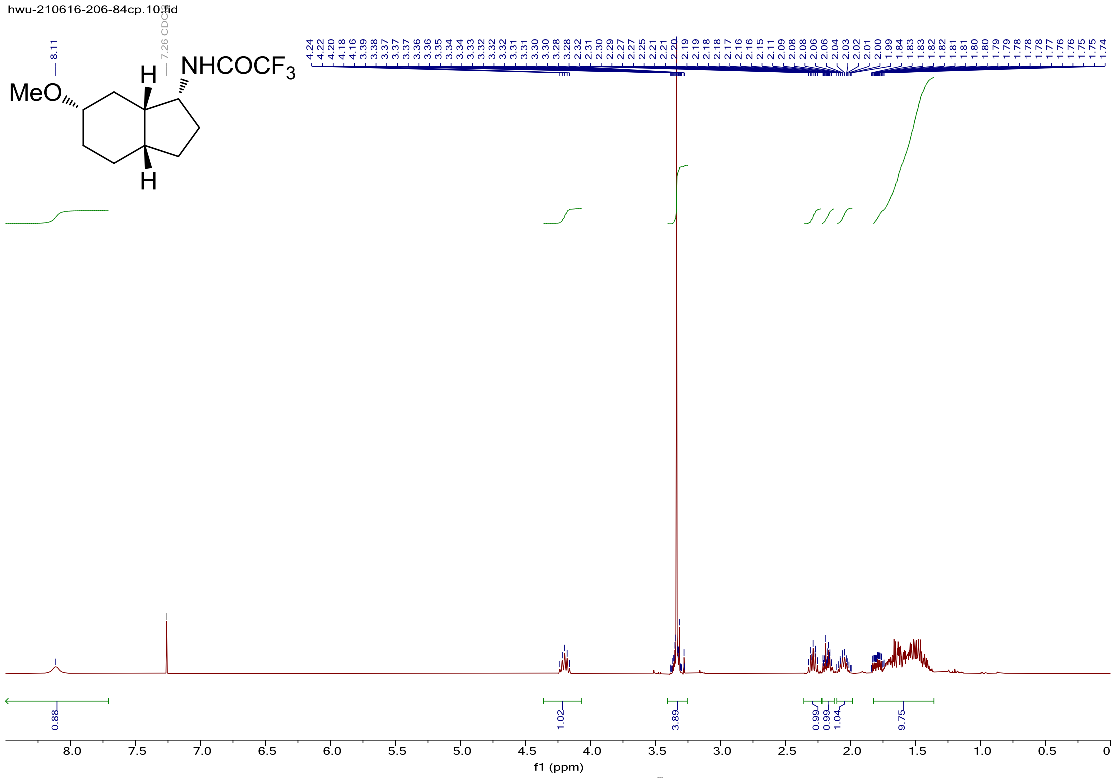


hwu-210616-206-84cp.12.fid

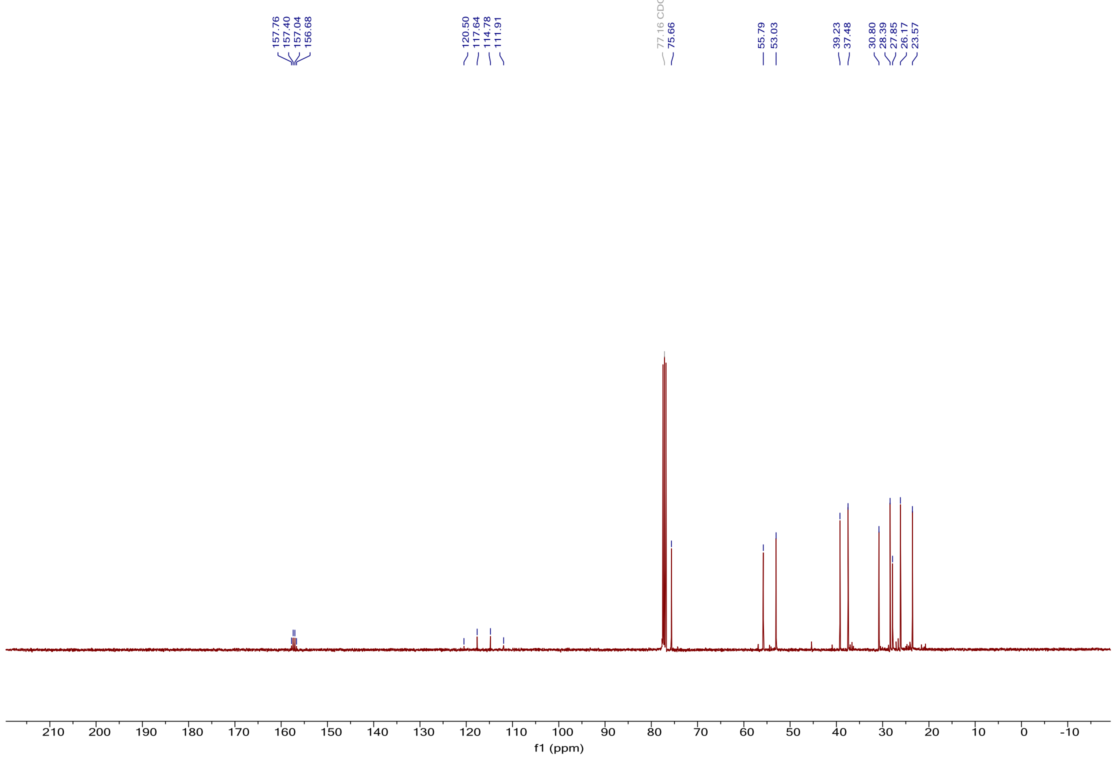

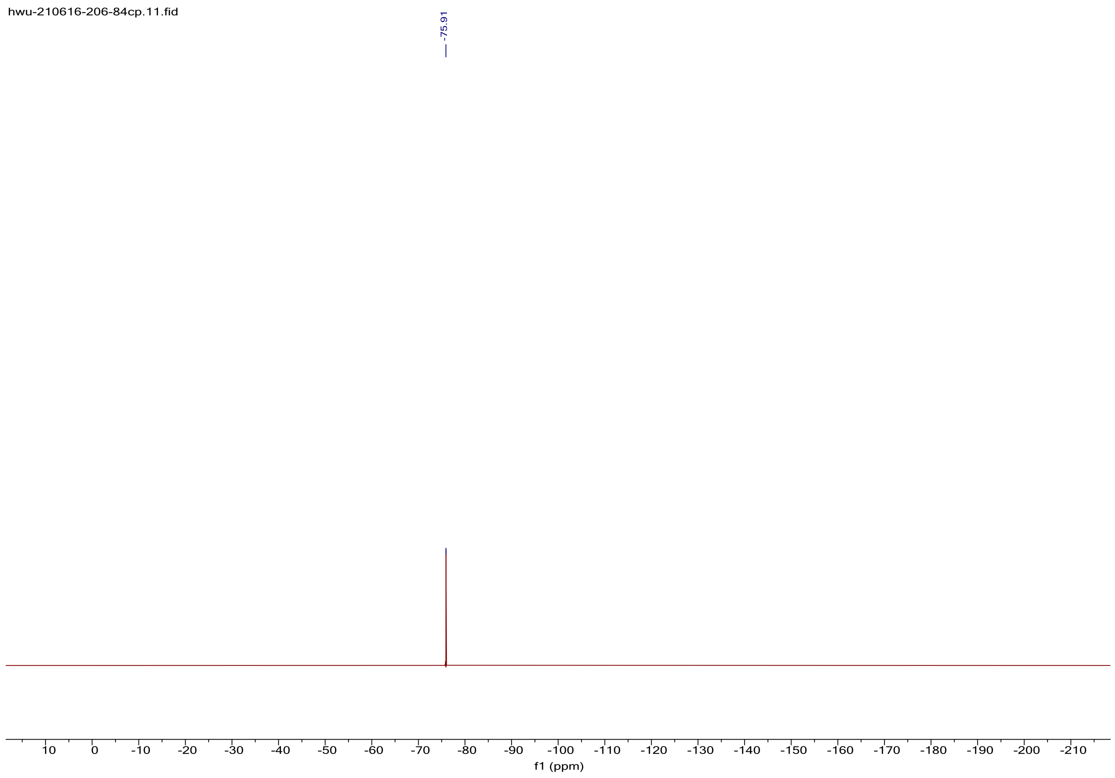
hwu-210616-206-84cp.11.fid
-75.91
10
0
-10
-20
-30
-40
-50
-60
-70
-80
-90
-100
-110
-120
-130
-140
-150
-160
-170
-180
-190
-200
-210
f1 (ppm)

hwu-2021061806-84ap2.1.fid

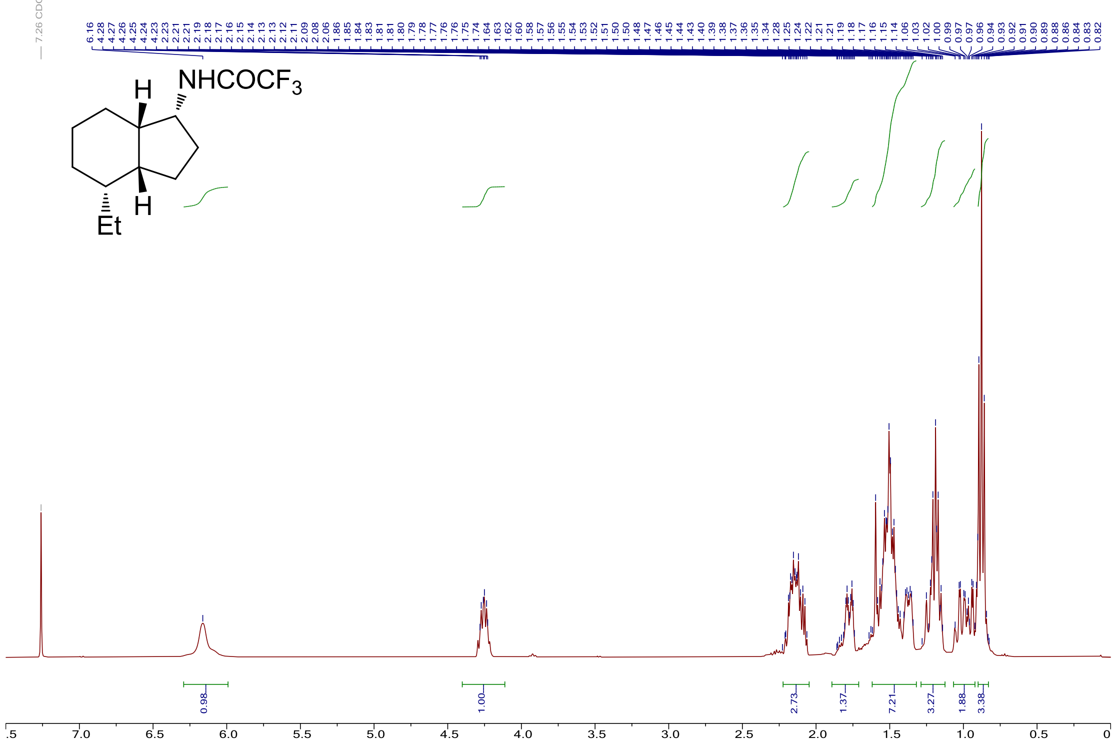


hwu-2021061806-84ap2.3.fid

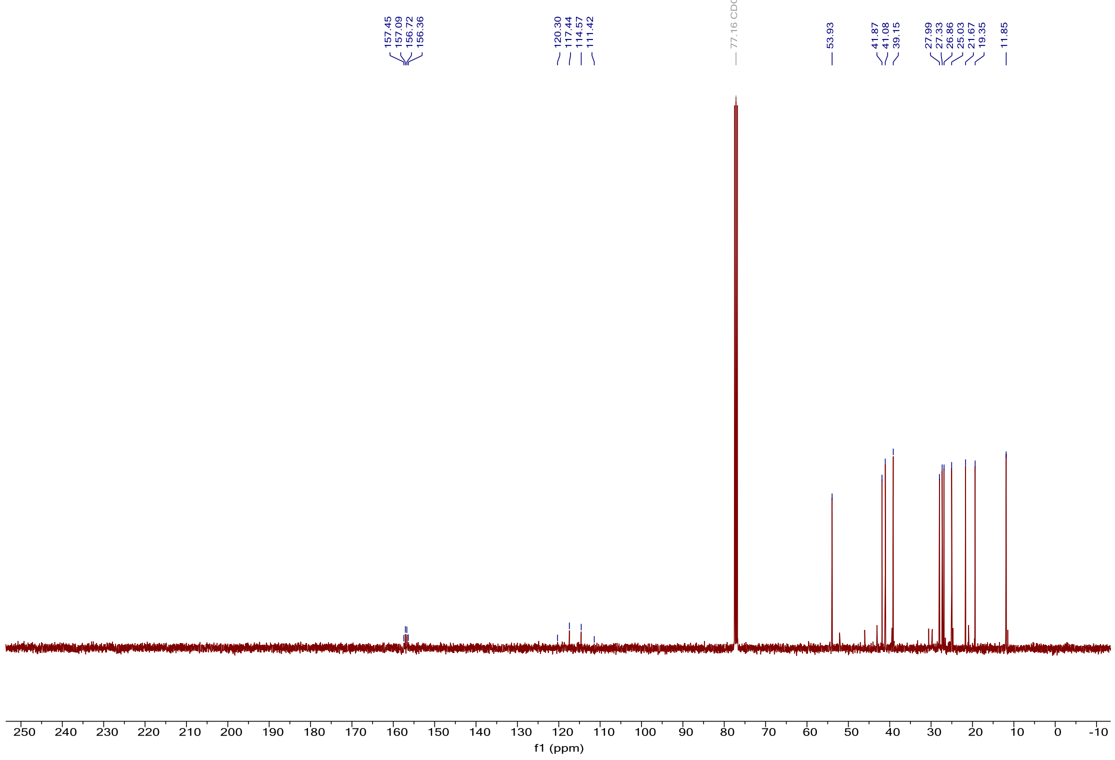

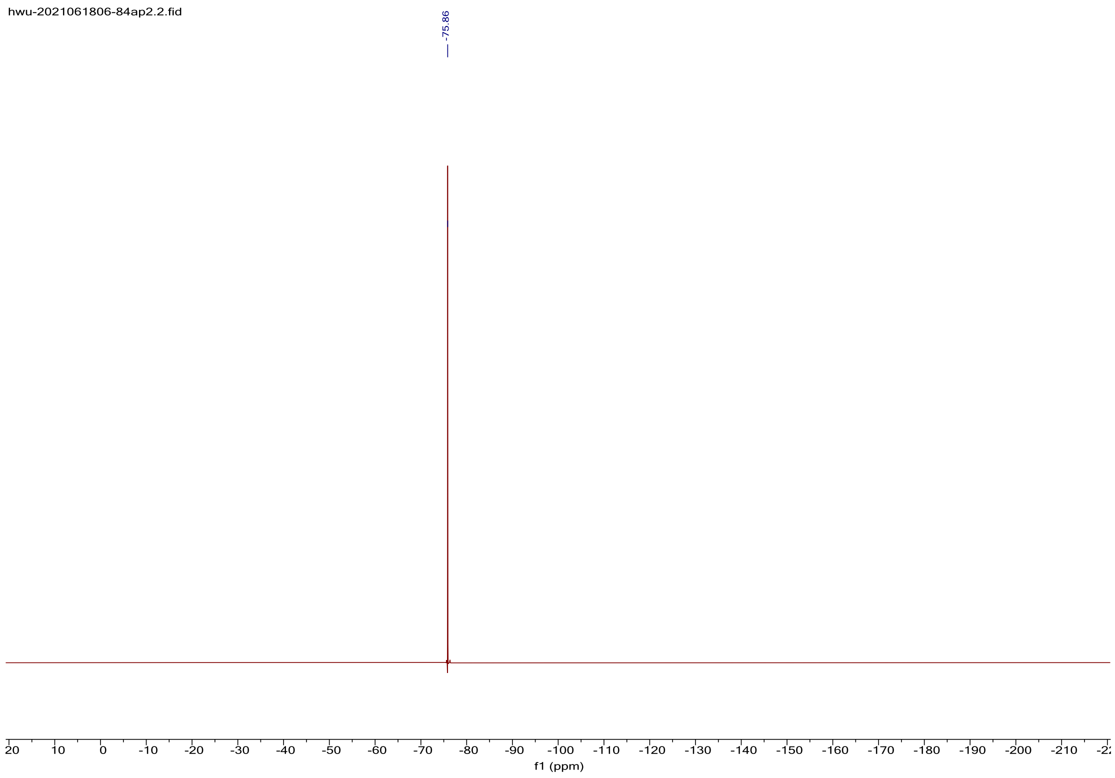
hwu-2021061806-84ap2.2.fid
-75.86
f1 (ppm)

yanji-20210618-9-91-8-P.1.fid

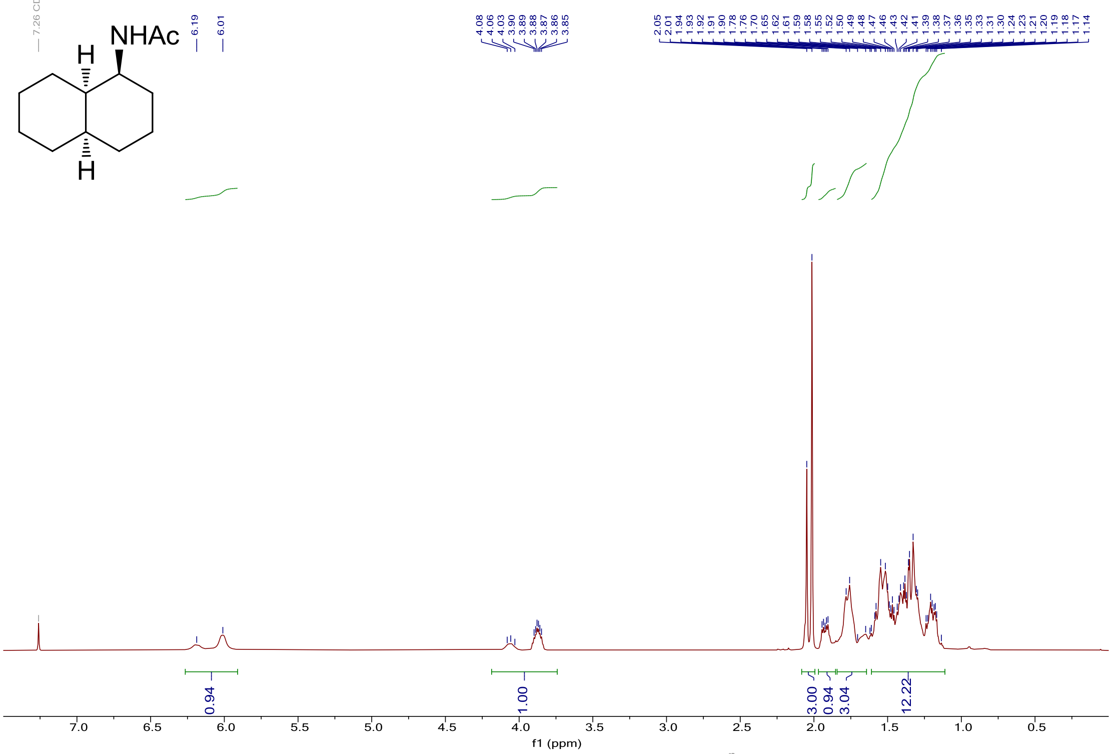

yanji-20210618-9-91-8-P.2.fid

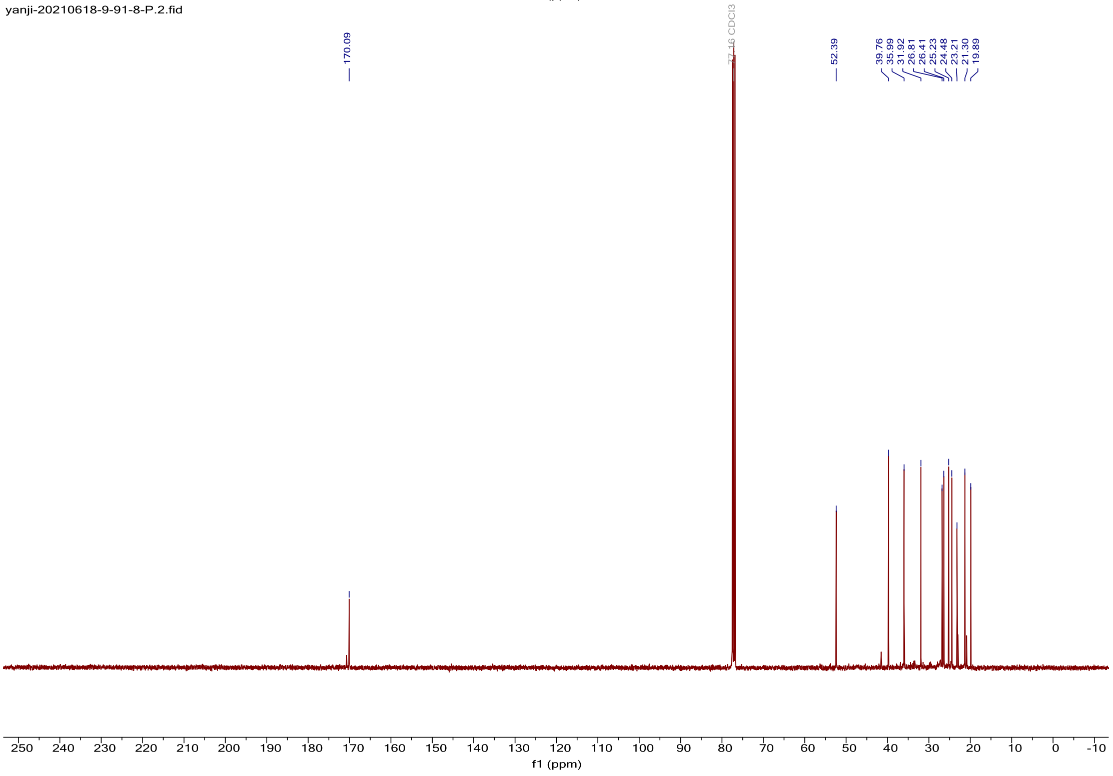

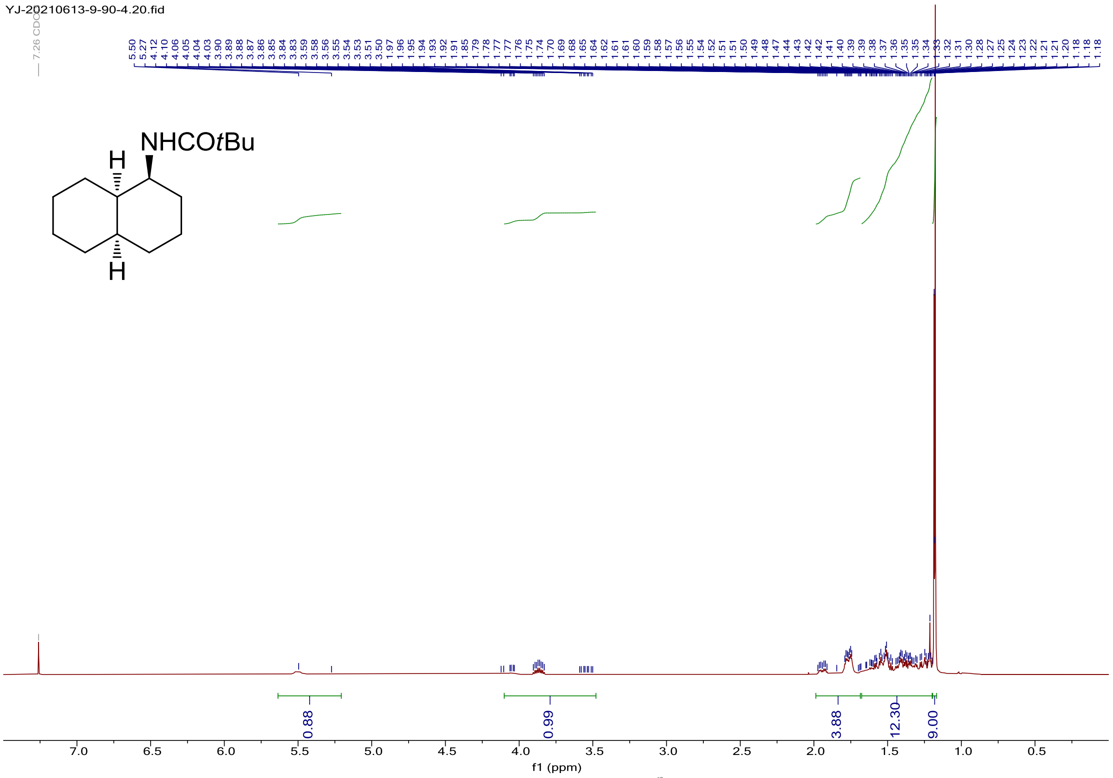
YJ-20210613-9-90-4.20.fid
7.26 CDCl3
5.50 5.27 4.12 4.10 4.06 4.05 4.04 4.03 3.90 3.89 3.88 3.87 3.86 3.85 3.84 3.83 3.59 3.58 3.56 3.55 3.54 3.53 3.51 3.50 1.97 1.96 1.95 1.94 1.93 1.92 1.91 1.85 1.79 1.78 1.77 1.77 1.76 1.75 1.74 1.70 1.69 1.68 1.65 1.64 1.62 1.61 1.61 1.60 1.59 1.58 1.57 1.56 1.55 1.54 1.52 1.51 1.51 1.50 1.49 1.48 1.47 1.44 1.43 1.42 1.42 1.41 1.40 1.39 1.39 1.38 1.37 1.36 1.35 1.35 1.34 1.33 1.32 1.31 1.30 1.28 1.27 1.25 1.24 1.23 1.22 1.21 1.21 1.20 1.18 1.18
NHCOtBu
H
H
0.88
0.99
3.88
12.30
9.00
7.0 6.5 6.0 5.5 5.0 4.5 4.0 3.5 3.0 2.5 2.0 1.5 1.0 0.5
f1 (ppm)

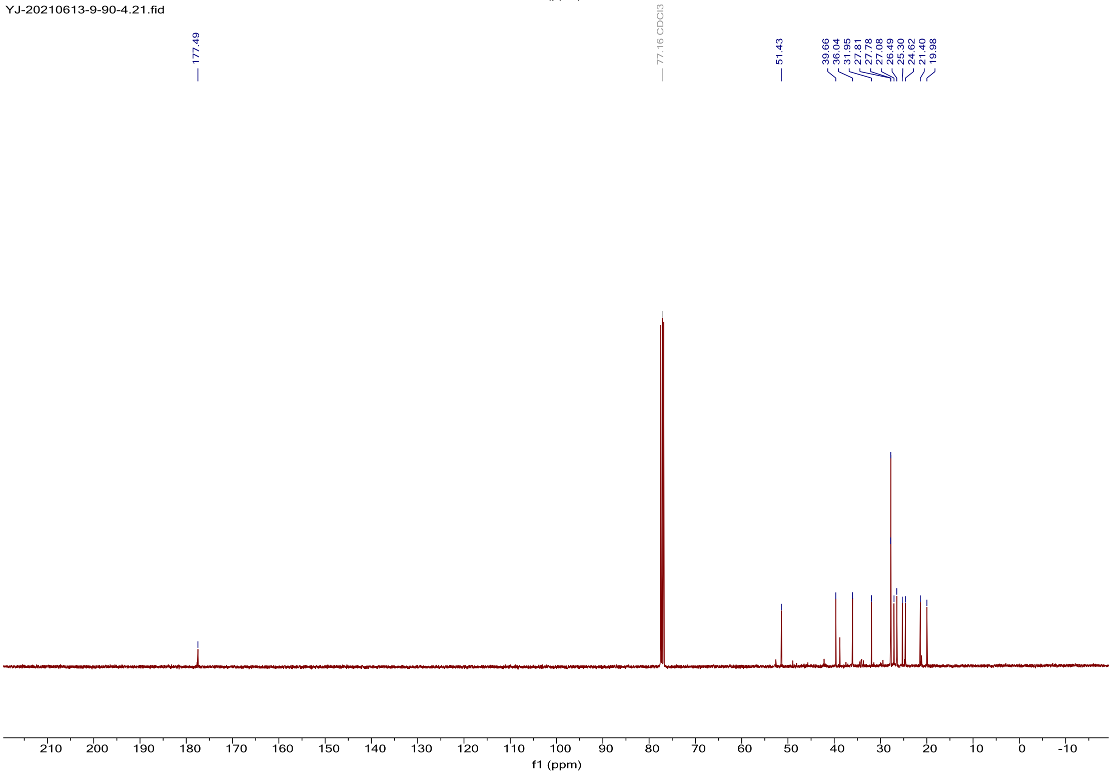
YJ-20210613-9-90-4.21.fid
177.49
77.16 CDCl3
51.43
39.66 36.04 31.95 27.81 27.78 27.08 26.49 25.30 24.62 21.40 19.98
210 200 190 180 170 160 150 140 130 120 110 100 90 80 70 60 50 40 30 20 10 0 -10
f1 (ppm)

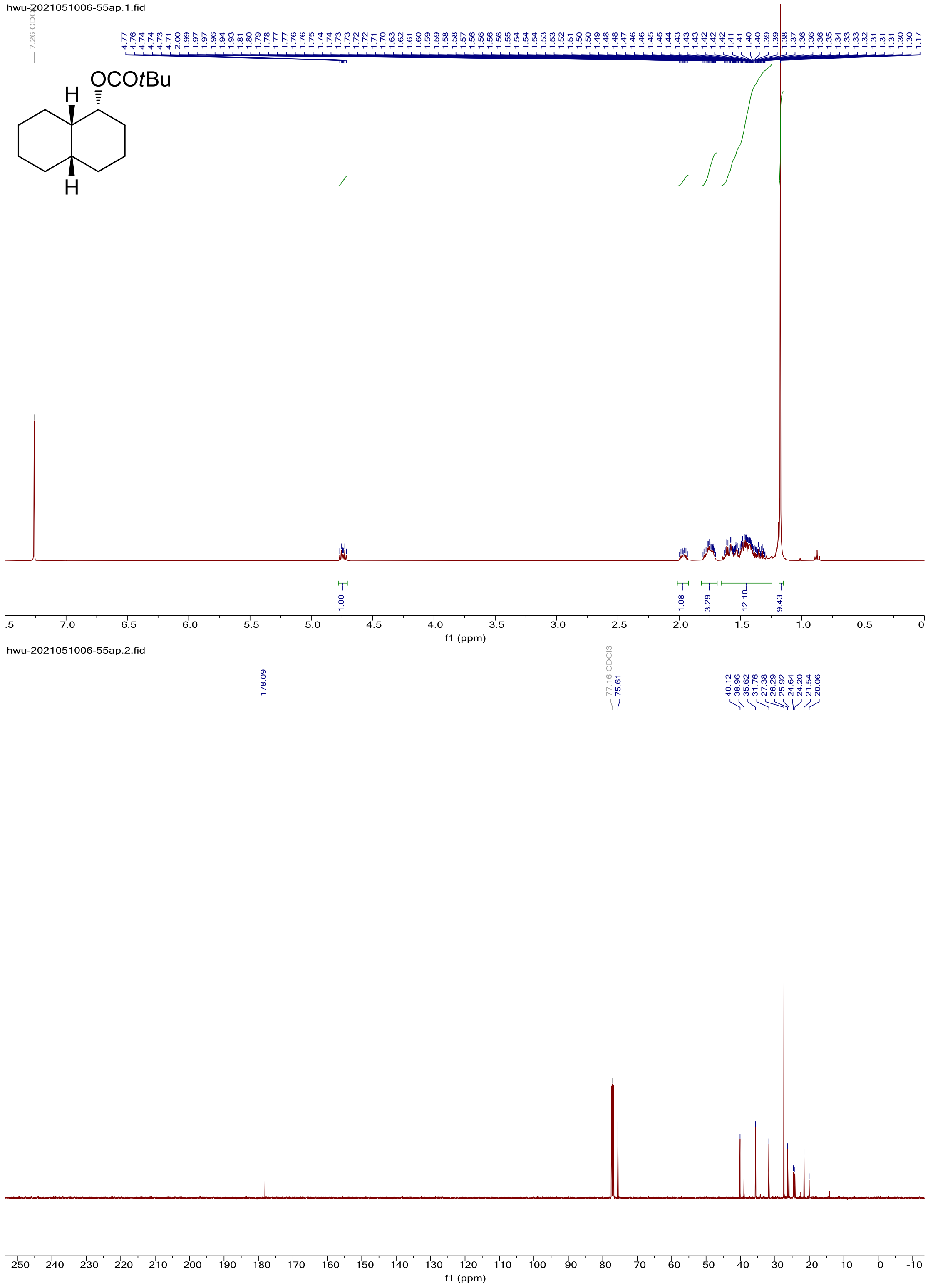
hwu-2021051006-55ap.1.fid
7.26 CDCl3
OCOtBu
H
H
1.00
1.08
3.29
12.10
9.43
f1 (ppm)
hwu-2021051006-55ap.2.fid
178.09
77.16 CDCl3
75.61
40.12
38.96
35.62
31.76
27.38
26.29
25.92
24.64
24.20
21.54
20.06
f1 (ppm)

hwu-2021070506-96apf.1.fid

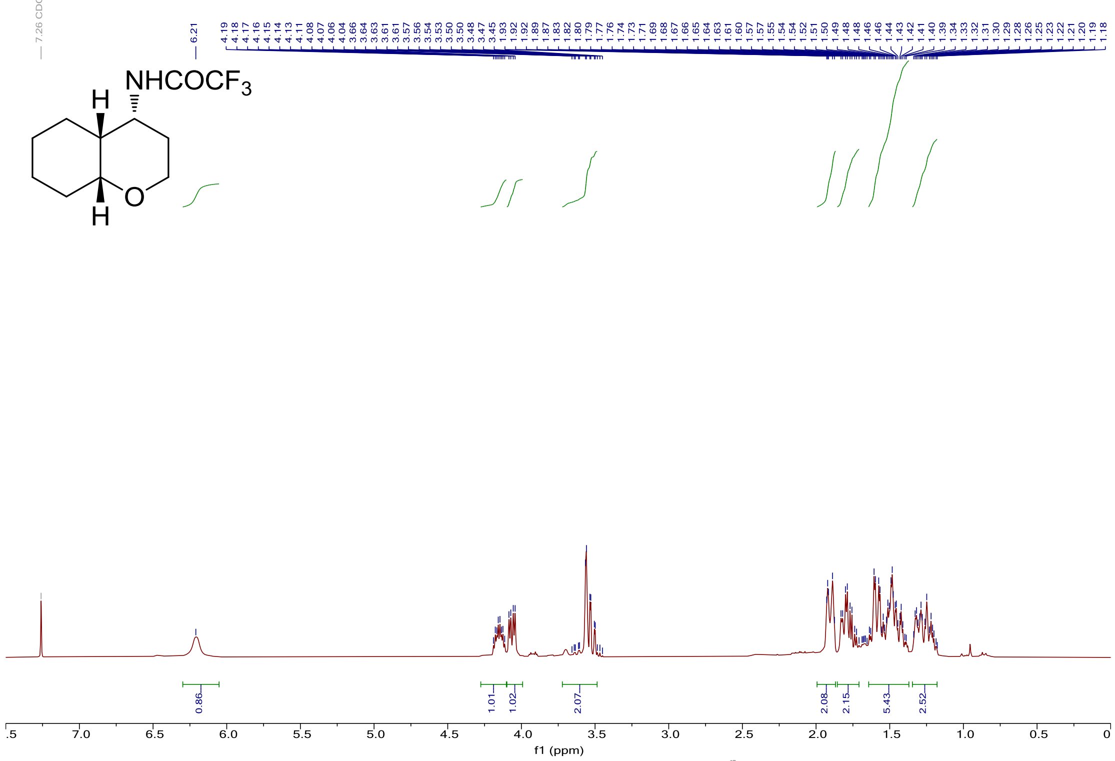


hwu-2021070506-96apf.3.fid

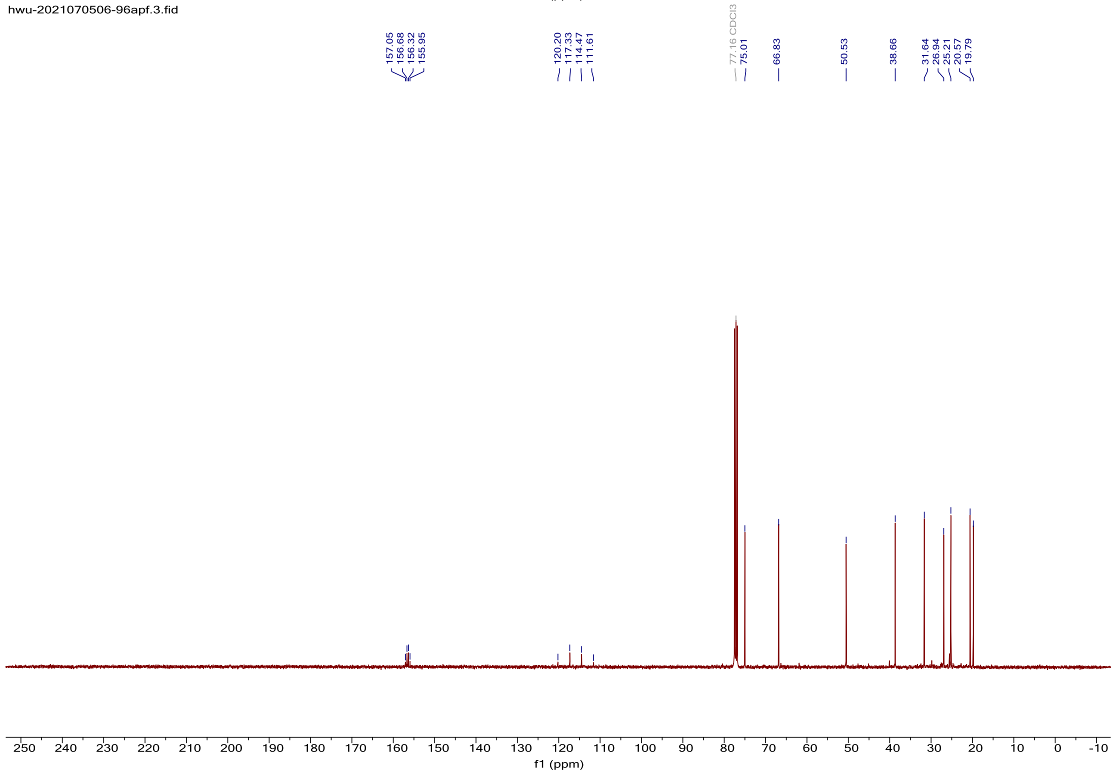

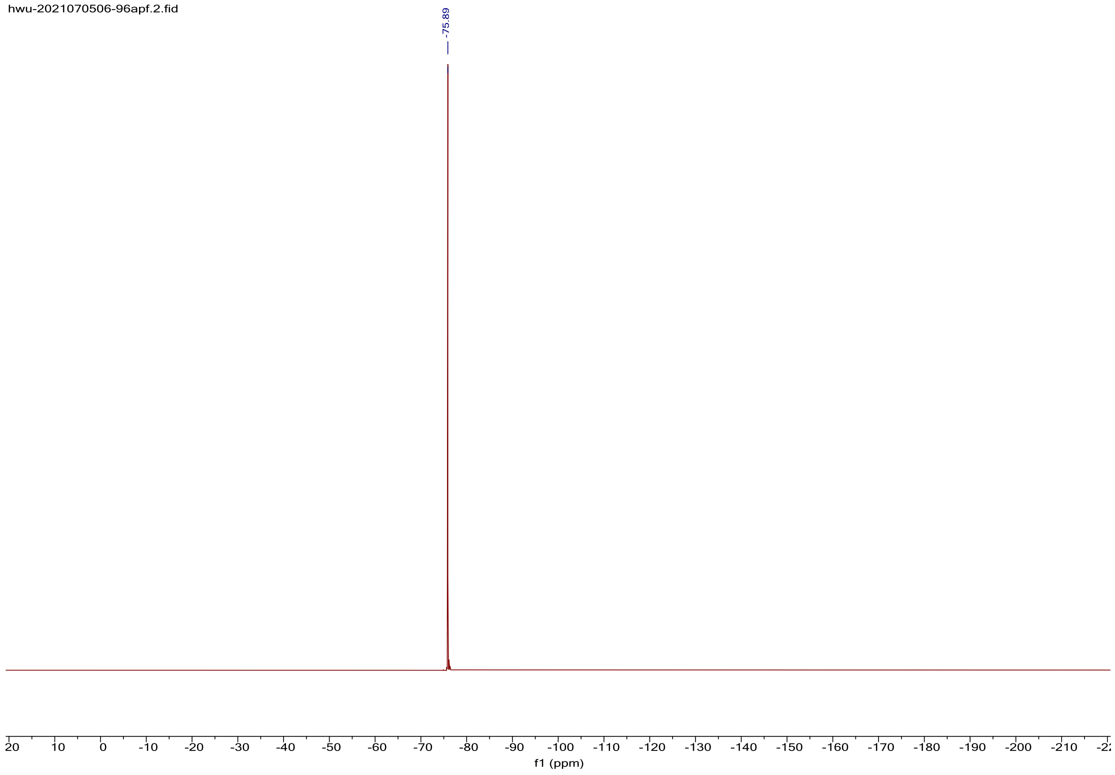
hwu-2021070506-96apf.2.fid
-75.89
20 10 0 -10 -20 -30 -40 -50 -60 -70 -80 -90 -100 -110 -120 -130 -140 -150 -160 -170 -180 -190 -200 -210
f1 (ppm)

hwu-210613-206-83ap.10.fid

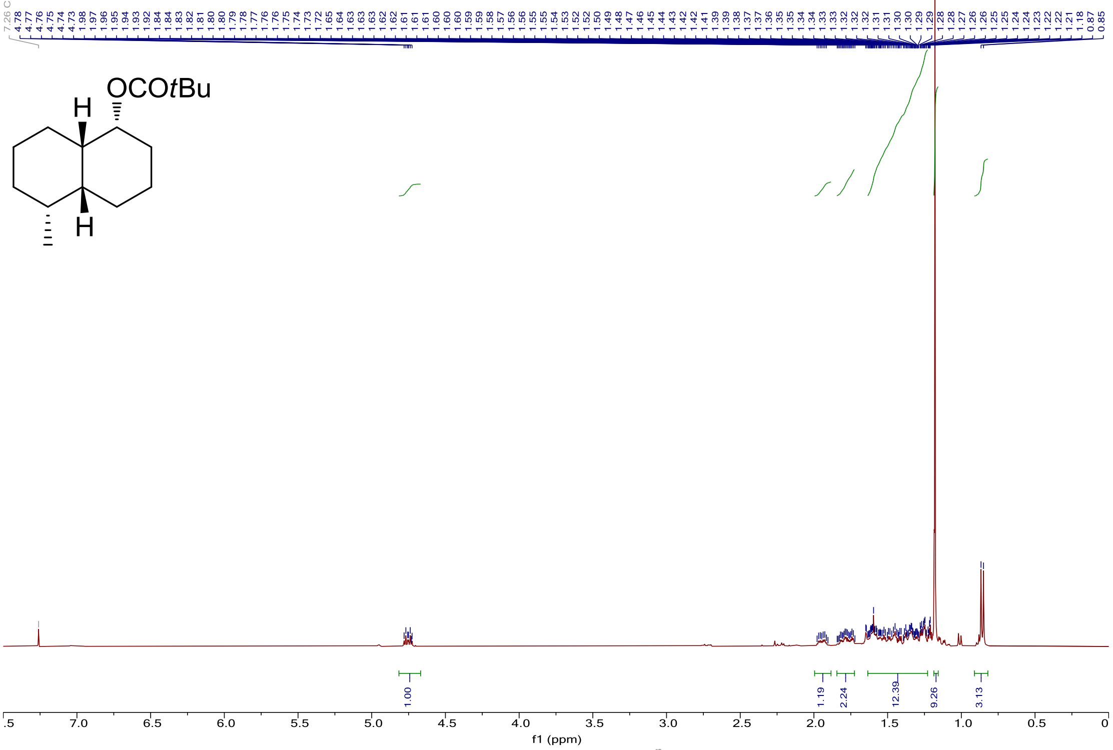


hwu-210613-206-83ap.11.fid

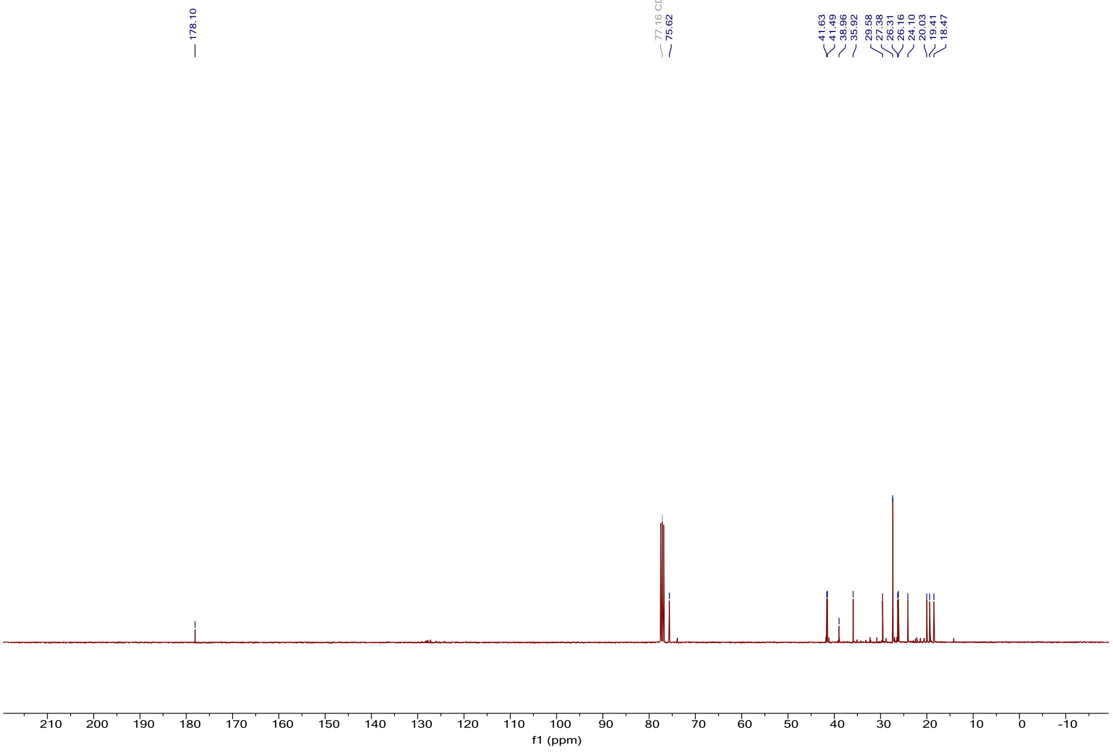

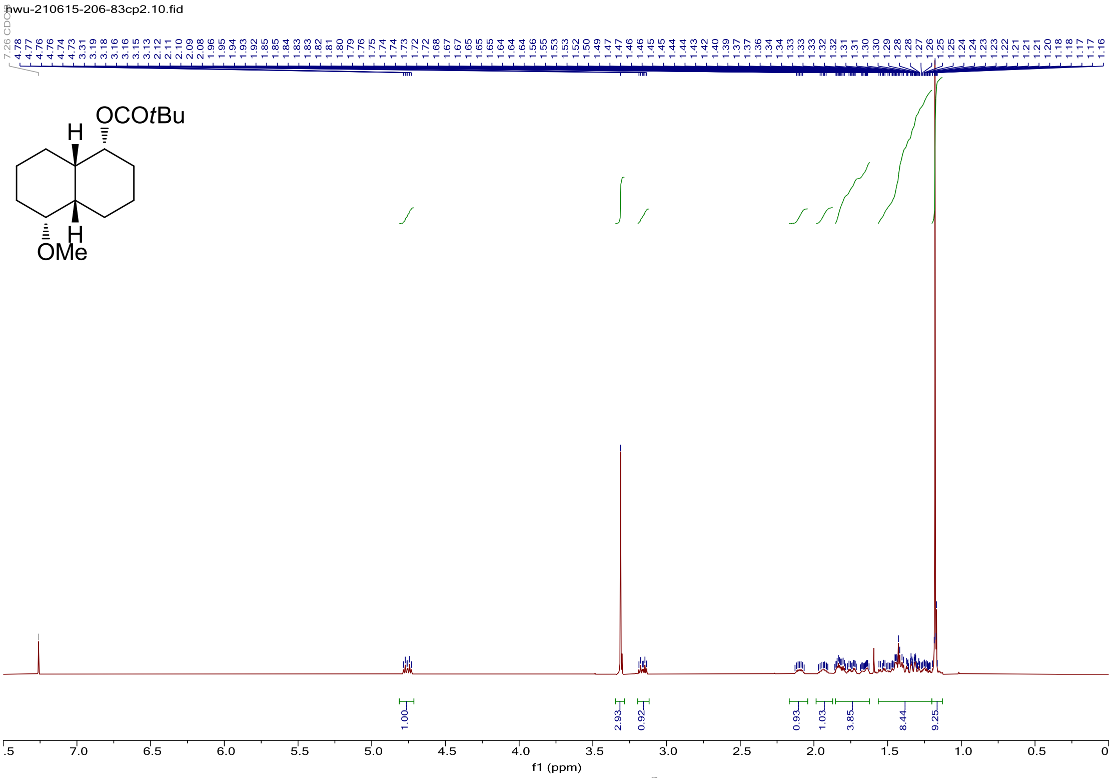
hwu-210615-206-83cp2.10.fid
7.26 CDCl3
4.78 4.77 4.76 4.76 4.74 4.73 3.31 3.19 3.18 3.16 3.16 3.15 3.13 2.12 2.11 2.10 2.09 2.08 1.96 1.95 1.94 1.93 1.92 1.85 1.85 1.84 1.83 1.83 1.82 1.81 1.80 1.79 1.76 1.75 1.74 1.74 1.73 1.73 1.72 1.68 1.67 1.67 1.65 1.65 1.64 1.64 1.64 1.56 1.55 1.55 1.53 1.53 1.52 1.50 1.49 1.47 1.47 1.46 1.46 1.45 1.45 1.44 1.44 1.43 1.42 1.40 1.39 1.37 1.37 1.36 1.34 1.34 1.33 1.33 1.33 1.32 1.32 1.31 1.31 1.30 1.30 1.29 1.28 1.28 1.27 1.26 1.26 1.25 1.25 1.24 1.24 1.23 1.23 1.22 1.21 1.21 1.21 1.20 1.18 1.18 1.17 1.17 1.16
H
OCOtBu
H
OMe
1.00
2.93
0.92
0.93
1.03
3.85
8.44
9.25
.5 7.0 6.5 6.0 5.5 5.0 4.5 4.0 3.5 3.0 2.5 2.0 1.5 1.0 0.5 0
f1 (ppm)

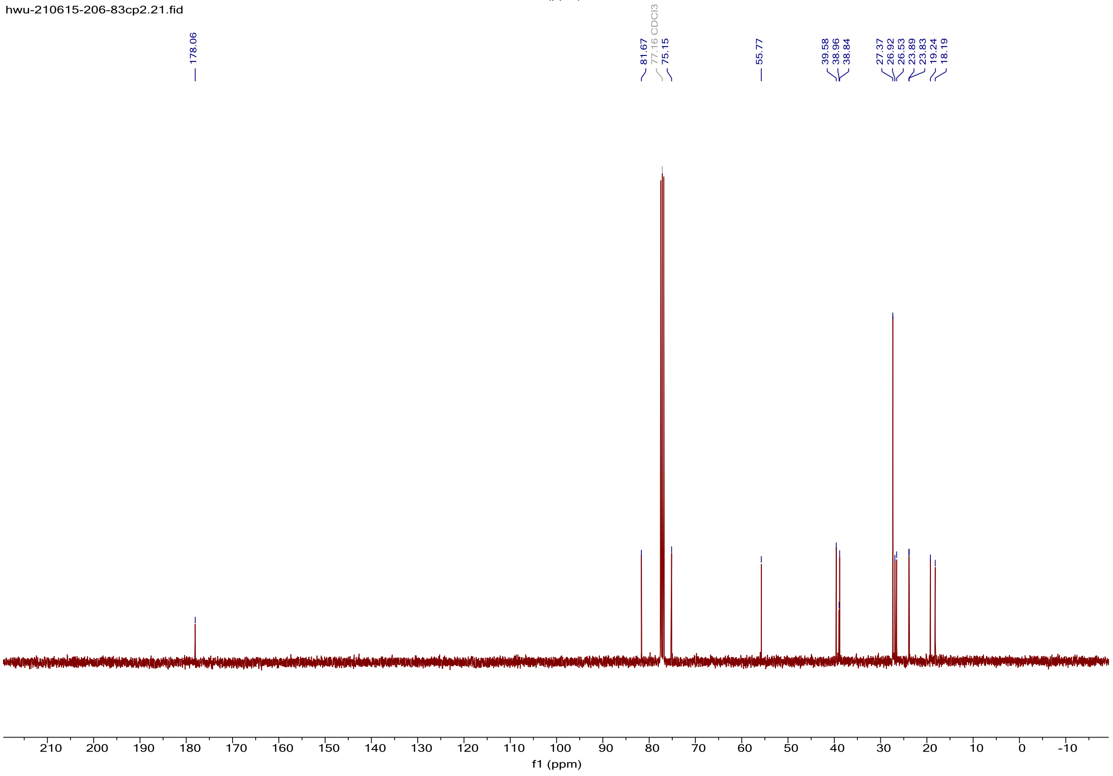
hwu-210615-206-83cp2.21.fid
178.06
81.67
77.16 CDCl3
75.15
55.77
39.58
38.96
38.84
27.37
26.92
26.53
23.89
23.83
19.24
18.19
210 200 190 180 170 160 150 140 130 120 110 100 90 80 70 60 50 40 30 20 10 0 -10
f1 (ppm)

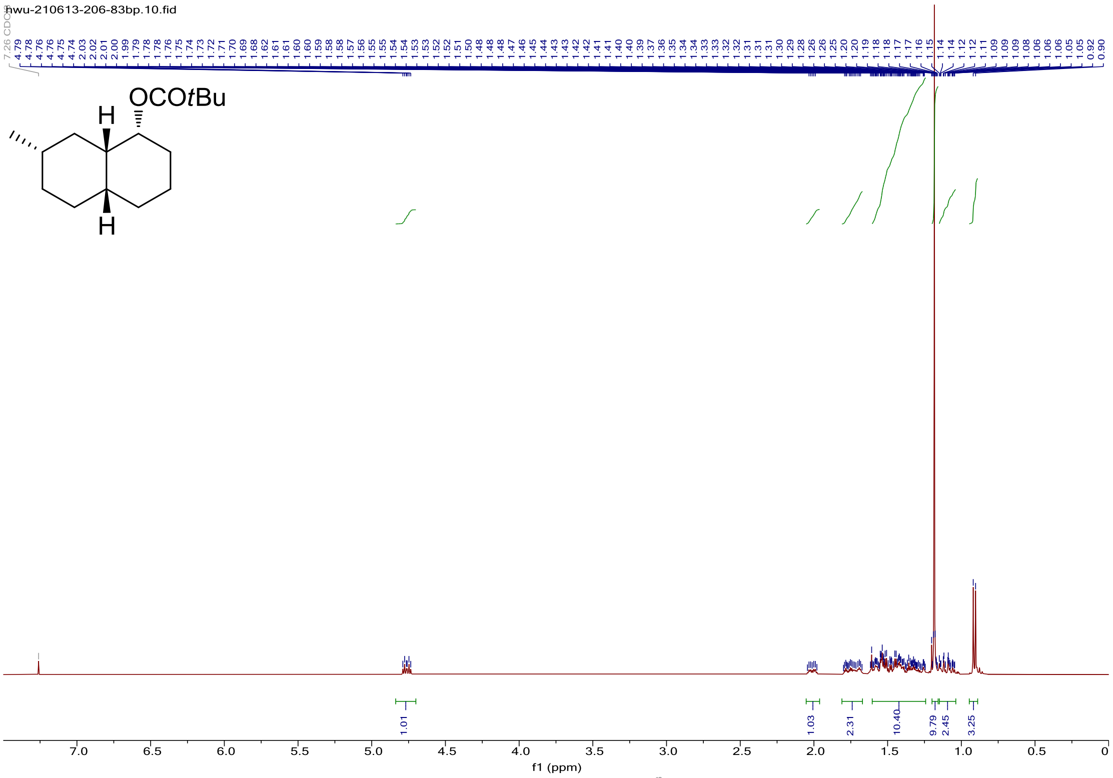
hwu-210613-206-83bp.10.fid
OCOtBu
H
H
1.01
1.03
2.31
10.40
9.79
2.45
3.25
f1 (ppm)

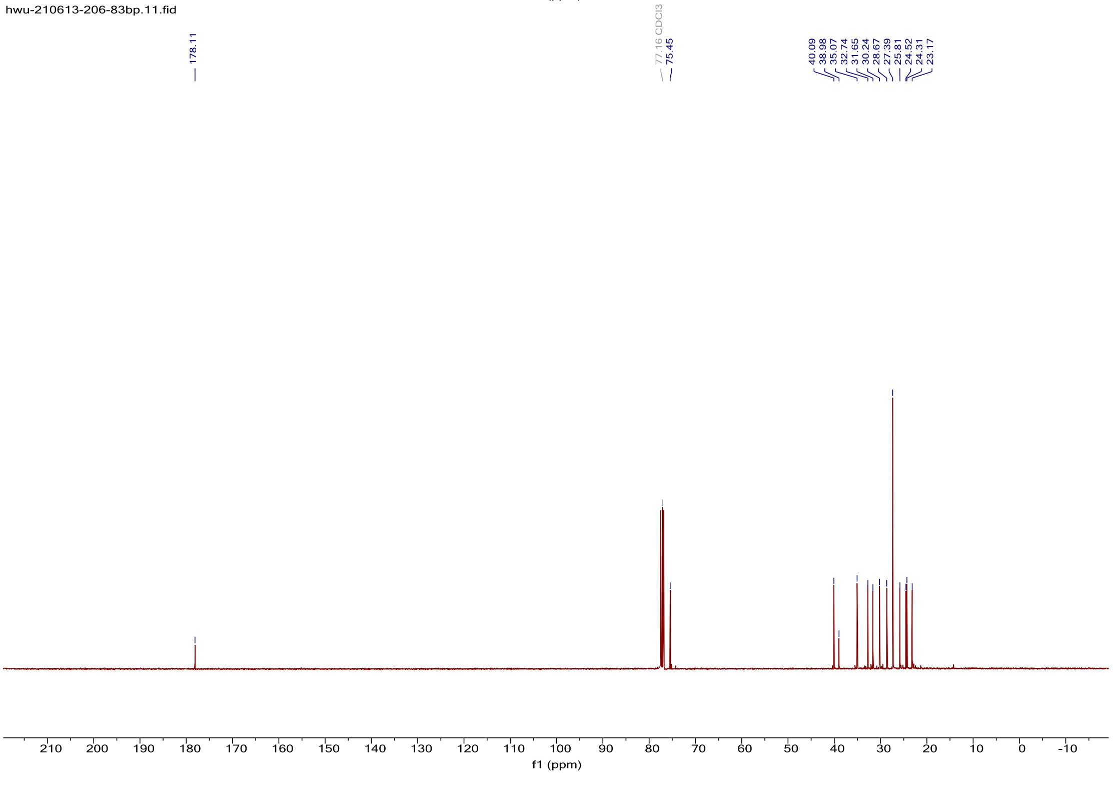
hwu-210613-206-83bp.11.fid
178.11
77.16 CDCl3
75.45
40.09
38.98
35.07
32.74
31.65
30.24
28.67
27.39
25.81
24.52
24.31
23.17
f1 (ppm)

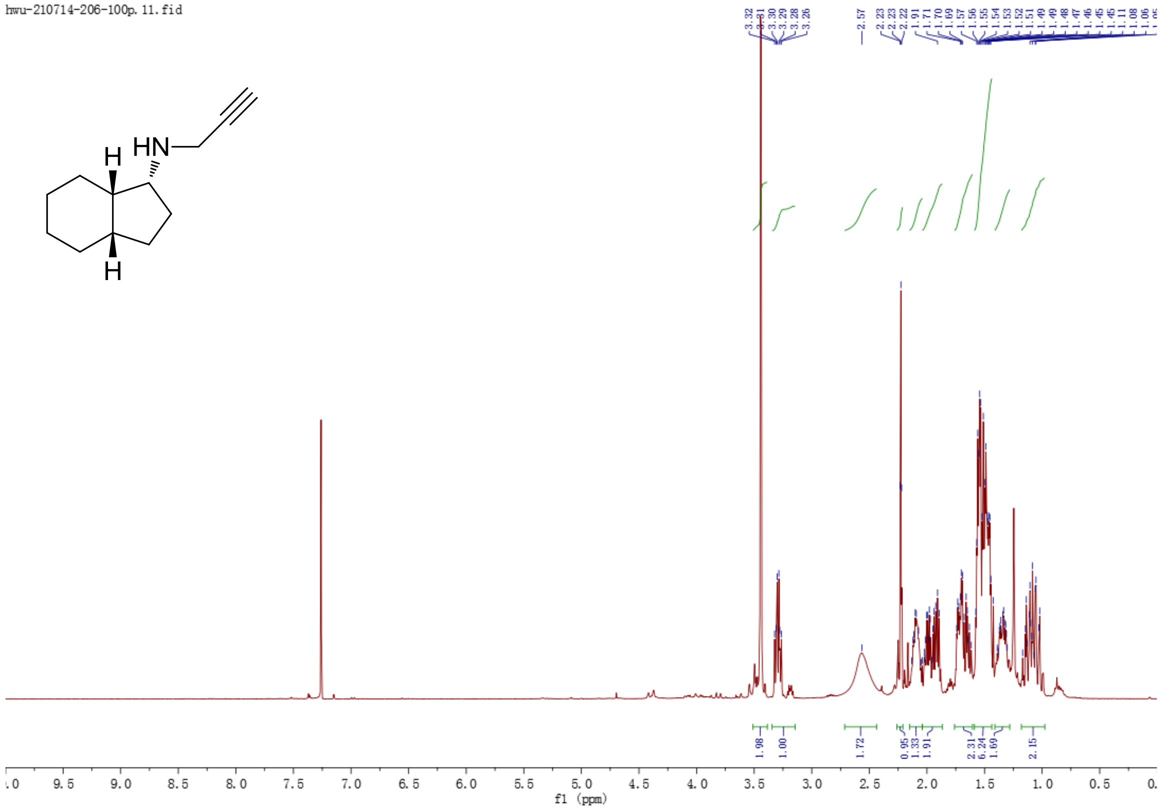
hwu-210714-206-100p.11.fid
HN
H
H
1.98
1.00
1.72
0.95
1.33
1.91
2.31
6.24
1.69
2.15
f1 (ppm)

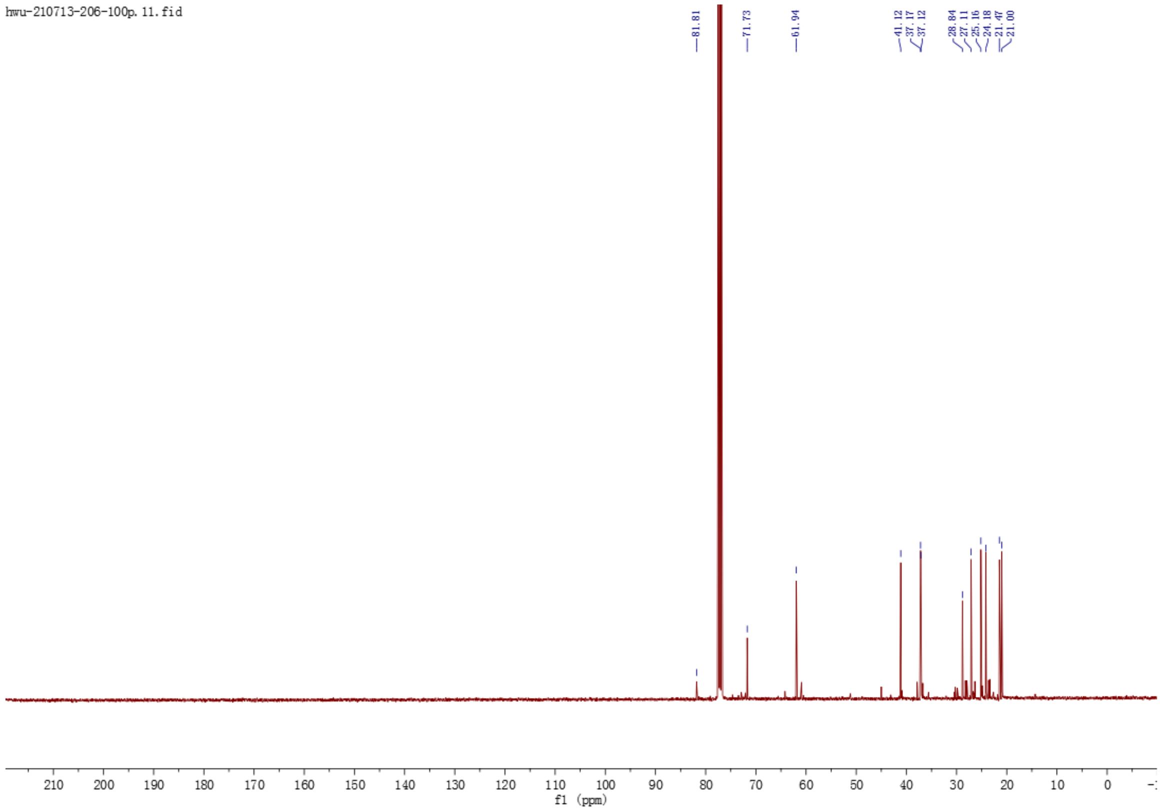
hwu-210713-206-100p.11.fid
81.81
71.73
61.94
41.12
37.17
37.12
28.84
27.11
25.16
24.18
21.47
21.00
f1 (ppm)

# 10. GC Chromatograms of hydrogenated products

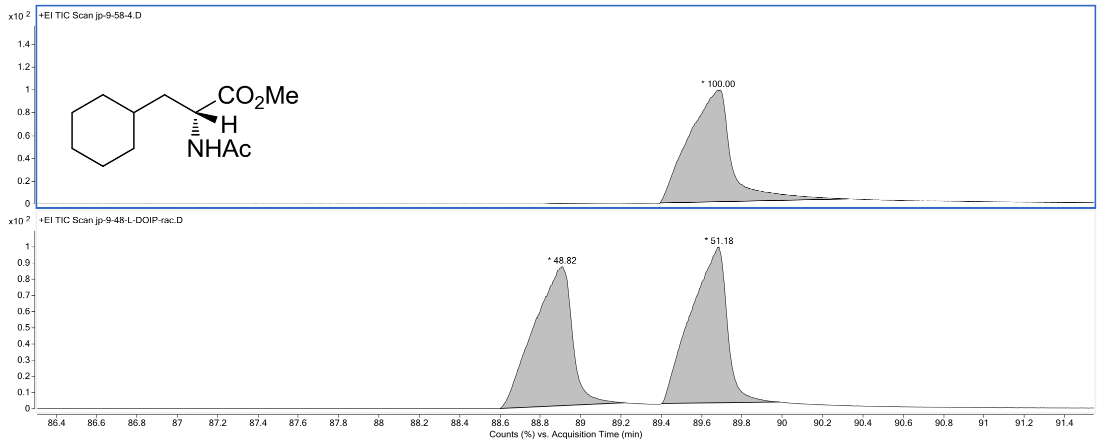


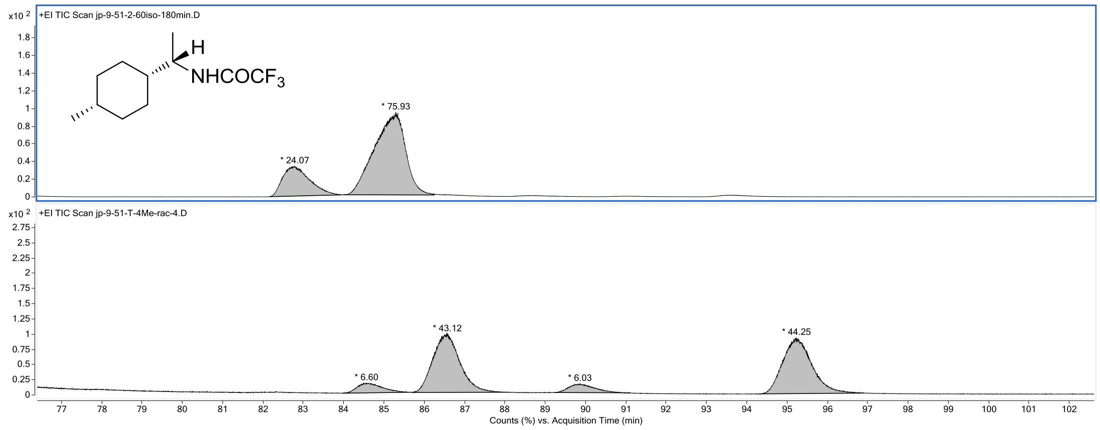


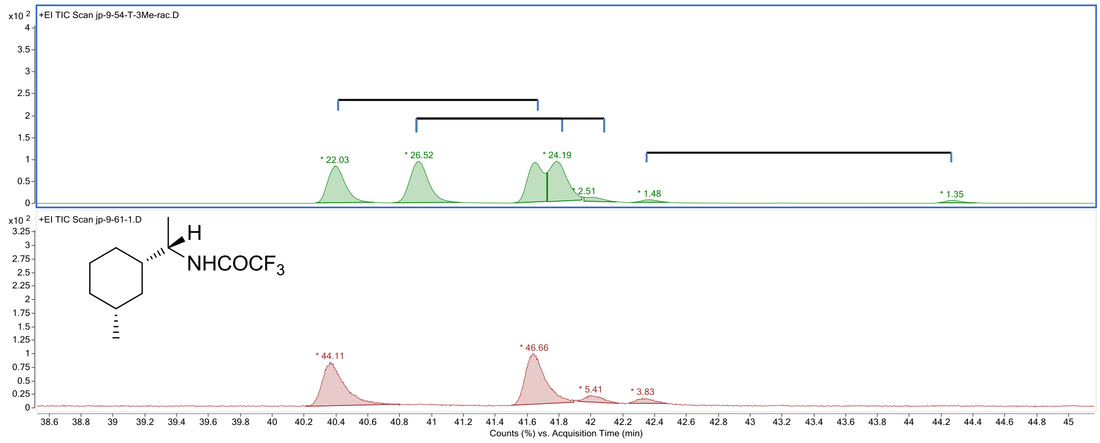

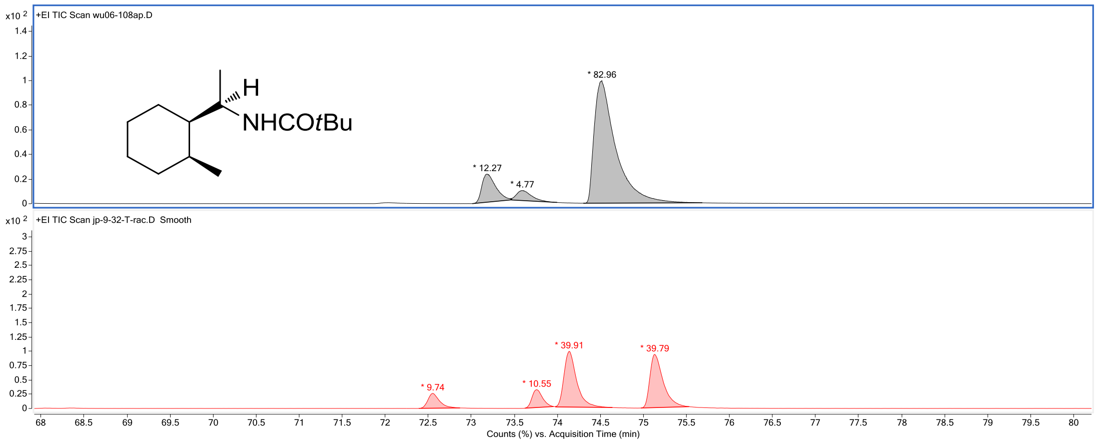
+EI TIC Scan wu06-108ap.D
NHCOtBu
H
* 82.96
* 12.27
* 4.77
+EI TIC Scan jp-9-32-T-rac.D Smooth
* 9.74
* 10.55
* 39.91
* 39.79
Counts (%) vs. Acquisition Time (min)

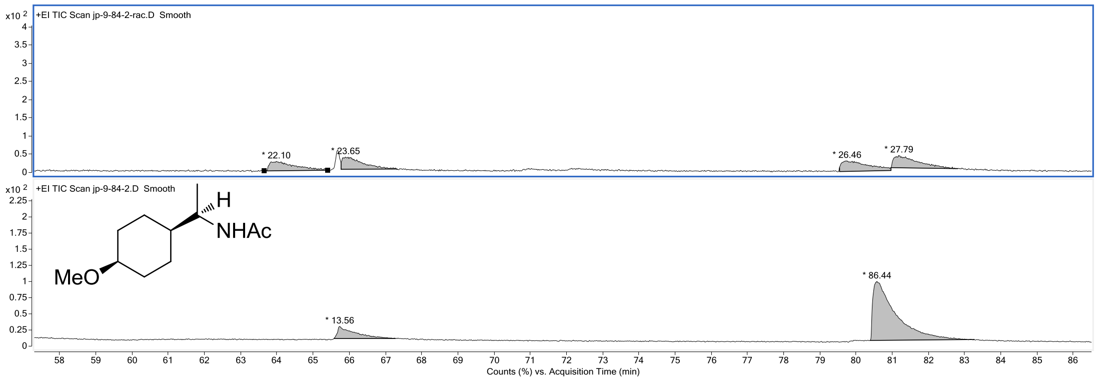
+EI TIC Scan jp-9-84-2-rac.D Smooth
* 22.10
* 23.65
* 26.46
* 27.79
+EI TIC Scan jp-9-84-2.D Smooth
H
NHAc
MeO
* 86.44
* 13.56
Counts (%) vs. Acquisition Time (min)

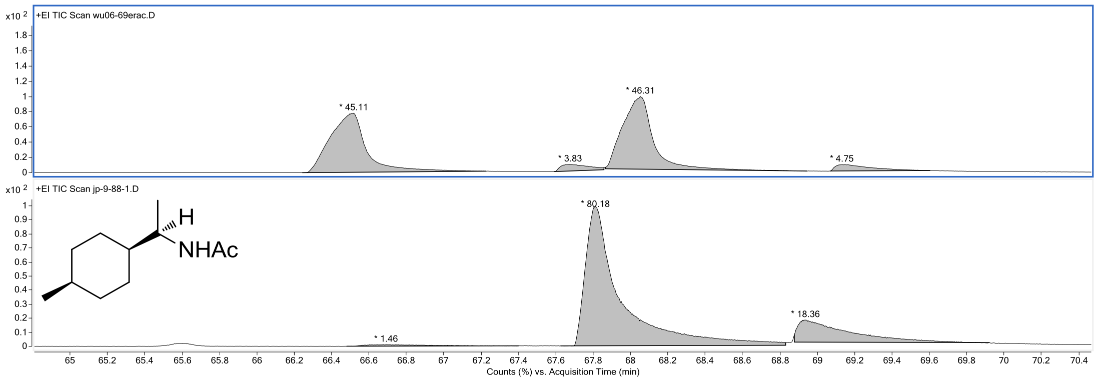
+EI TIC Scan wu06-69erac.D
* 45.11
* 46.31
* 3.83
* 4.75
+EI TIC Scan jp-9-88-1.D
H
NHAc
* 80.18
* 18.36
* 1.46
Counts (%) vs. Acquisition Time (min)

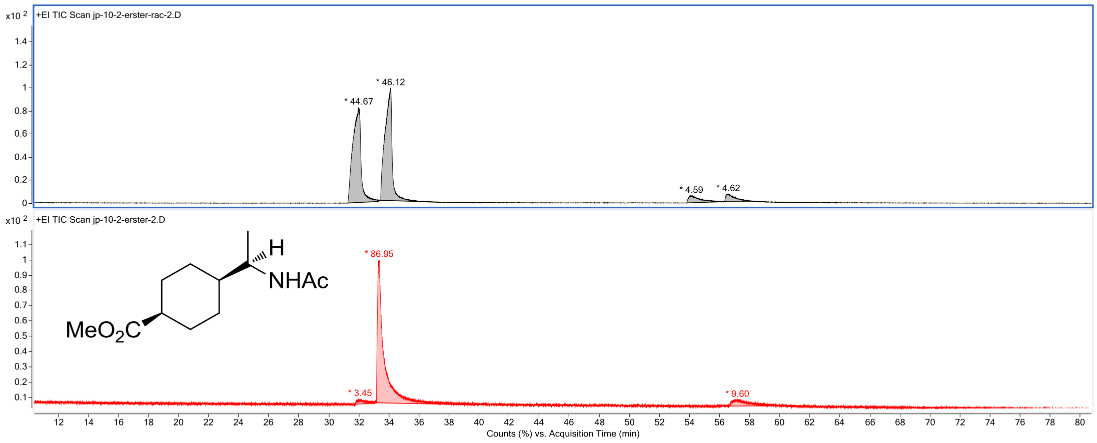
+EI TIC Scan jp-10-2-erster-rac-2.D
* 44.67
* 46.12
* 4.59
* 4.62
+EI TIC Scan jp-10-2-erster-2.D
H
NHAc
MeO2C
* 86.95
* 3.45
* 9.60
Counts (%) vs. Acquisition Time (min)

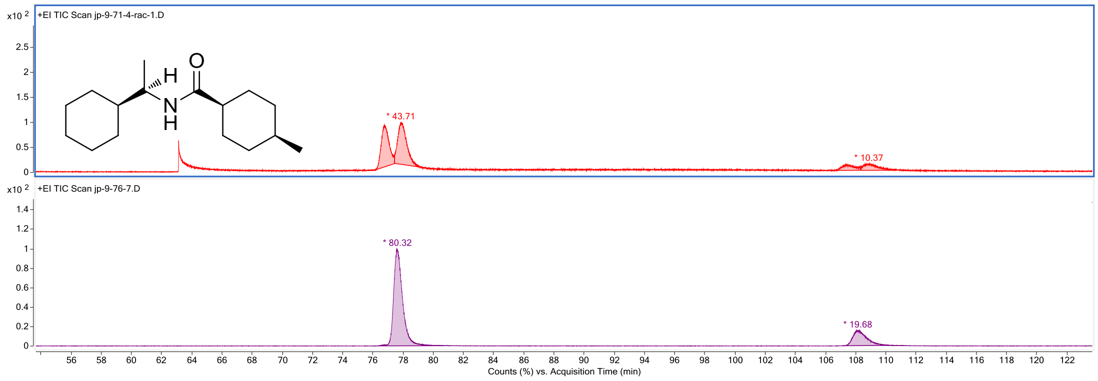
+EI TIC Scan jp-9-71-4-rac-1.D
* 43.71
* 10.37
+EI TIC Scan jp-9-76-7.D
* 80.32
* 19.68
Counts (%) vs. Acquisition Time (min)

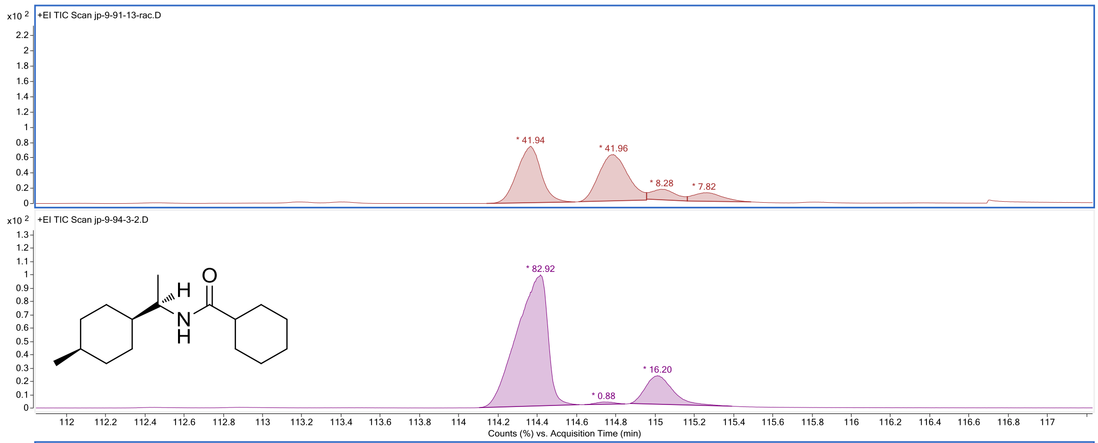
+EI TIC Scan jp-9-91-13-rac.D
* 41.94
* 41.96
* 8.28
* 7.82
+EI TIC Scan jp-9-94-3-2.D
* 82.92
* 0.88
* 16.20
Counts (%) vs. Acquisition Time (min)

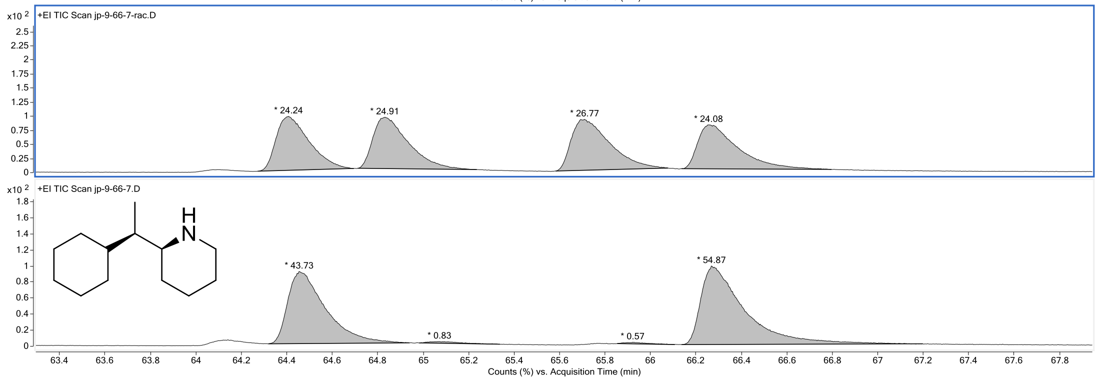
+EI TIC Scan jp-9-66-7-rac.D
* 24.24
* 24.91
* 26.77
* 24.08
+EI TIC Scan jp-9-66-7.D
* 43.73
* 0.83
* 0.57
* 54.87
Counts (%) vs. Acquisition Time (min)

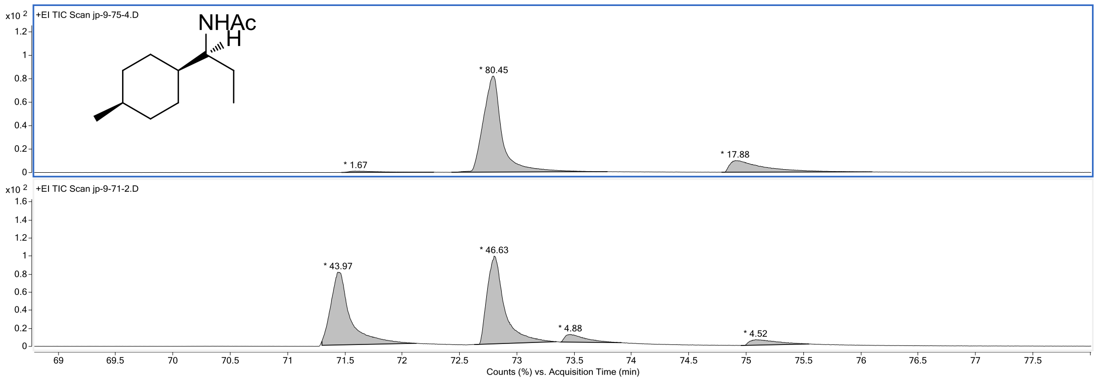
+EI TIC Scan jp-9-75-4.D
NHAc
* 1.67
* 80.45
* 17.88
+EI TIC Scan jp-9-71-2.D
* 43.97
* 46.63
* 4.88
* 4.52
Counts (%) vs. Acquisition Time (min)

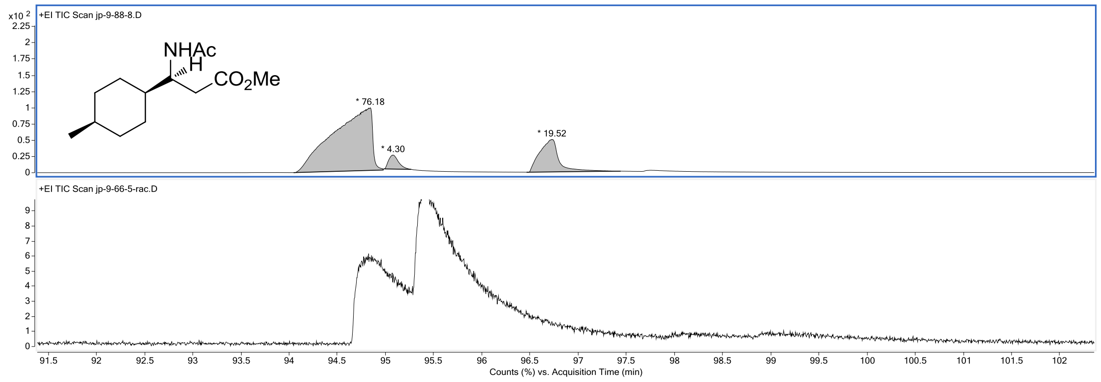
+EI TIC Scan jp-9-88-8.D
NHAc
H
CO2Me
* 76.18
* 4.30
* 19.52
+EI TIC Scan jp-9-66-5-rac.D
Counts (%) vs. Acquisition Time (min)

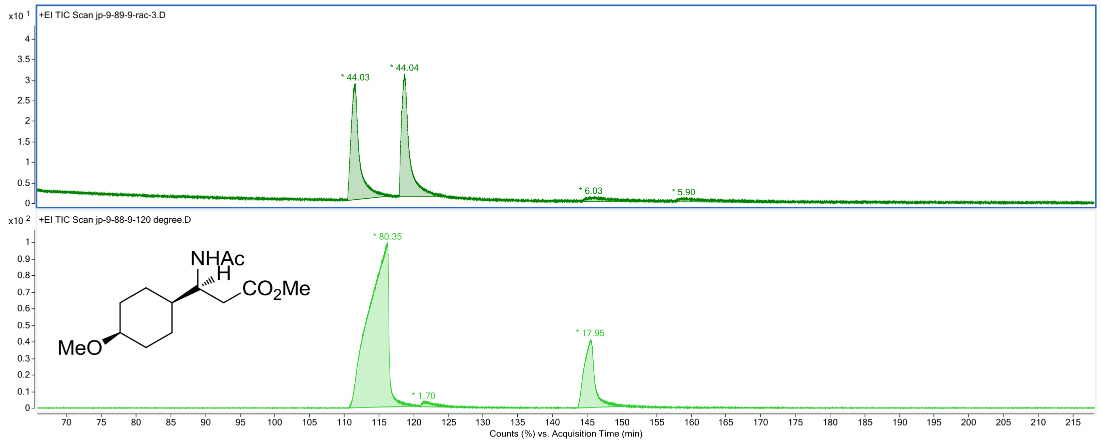
+EI TIC Scan jp-9-89-9-rac-3.D
* 44.03
* 44.04
* 6.03
* 5.90
+EI TIC Scan jp-9-88-9-120 degree.D
NHAc
H
CO2Me
MeO
* 80.35
* 1.70
* 17.95
Counts (%) vs. Acquisition Time (min)

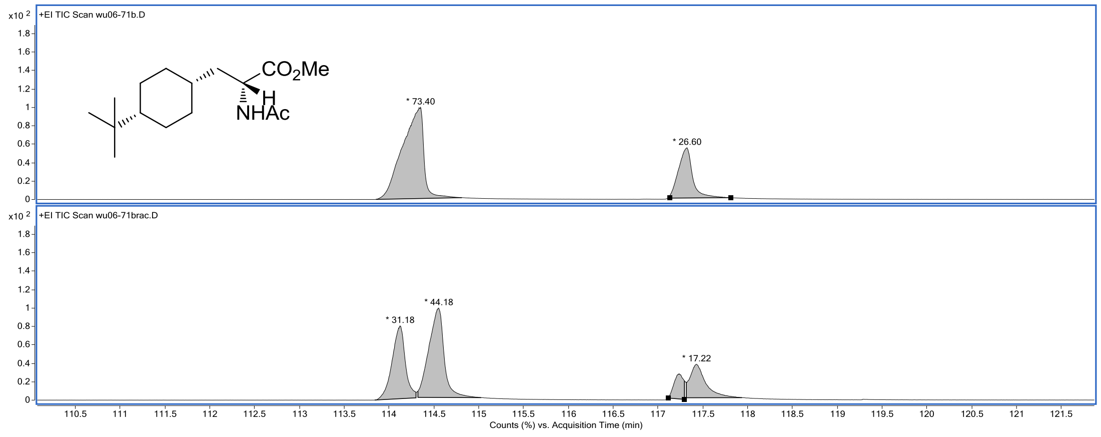
+EI TIC Scan wu06-71b.D
CO2Me
H
NHAc
* 73.40
* 26.60
+EI TIC Scan wu06-71brac.D
* 31.18
* 44.18
* 17.22
Counts (%) vs. Acquisition Time (min)

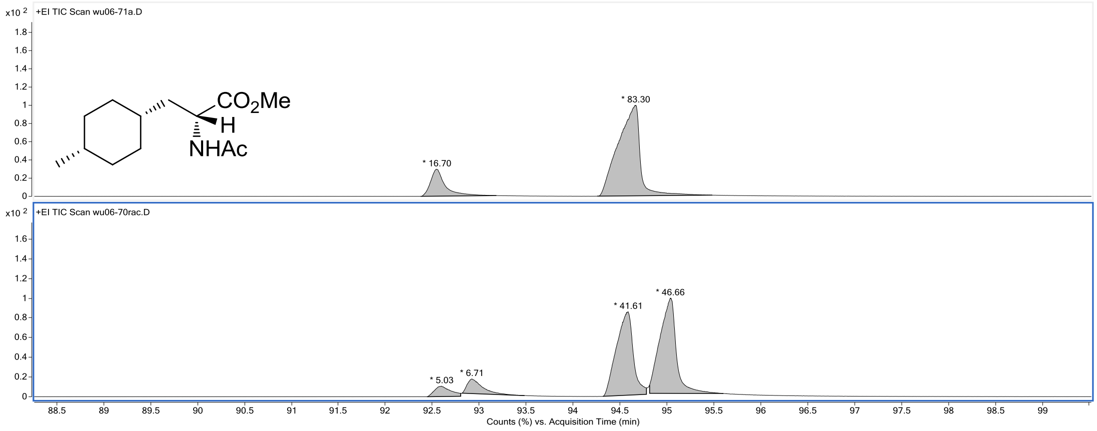

+EI TIC Scan wu06-71a.D
CO2Me
H
NHAc
* 16.70
* 83.30
+EI TIC Scan wu06-70rac.D
* 5.03
* 6.71
* 41.61
* 46.66
Counts (%) vs. Acquisition Time (min)


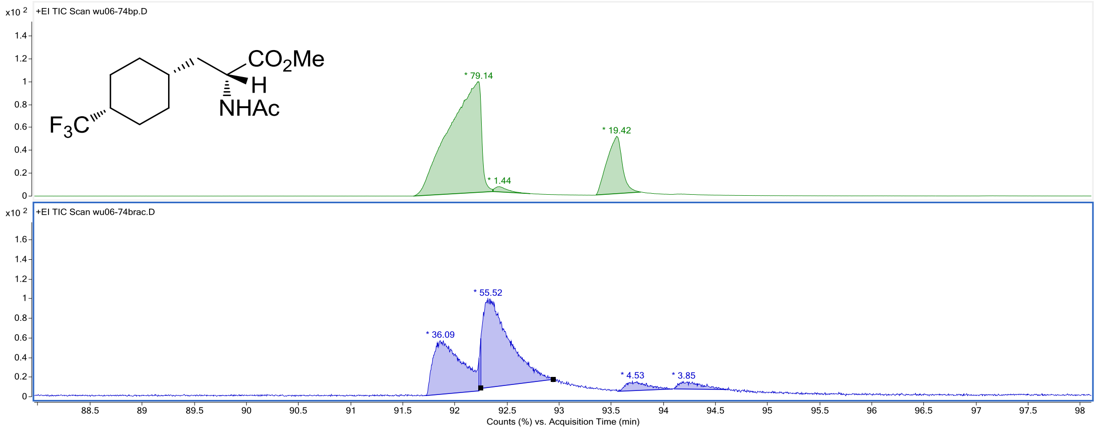

+EI TIC Scan wu06-74bp.D
CO2Me
H
NHAc
F3C
* 79.14
* 1.44
* 19.42
+EI TIC Scan wu06-74brac.D
* 36.09
* 55.52
* 4.53
* 3.85
Counts (%) vs. Acquisition Time (min)


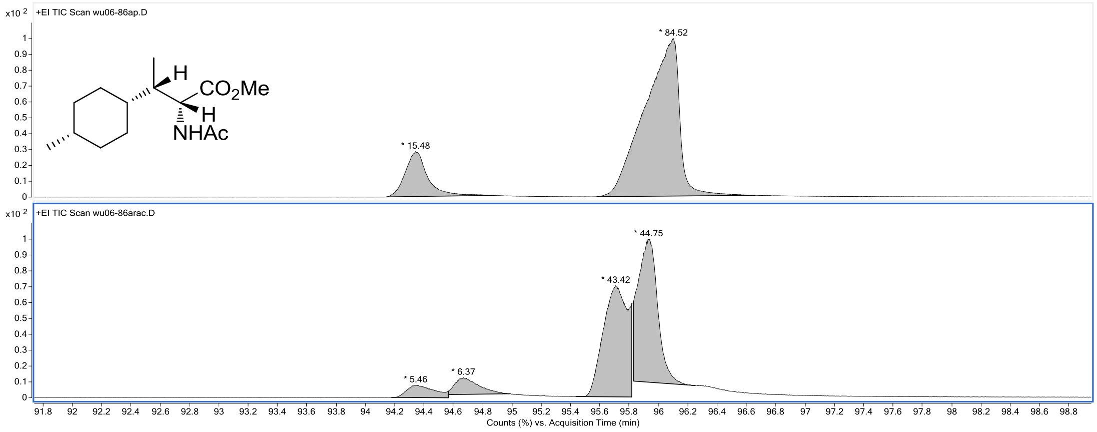

+EI TIC Scan wu06-86ap.D
H
CO2Me
H
NHAc
* 15.48
* 84.52
+EI TIC Scan wu06-86arac.D
* 5.46
* 6.37
* 43.42
* 44.75
Counts (%) vs. Acquisition Time (min)


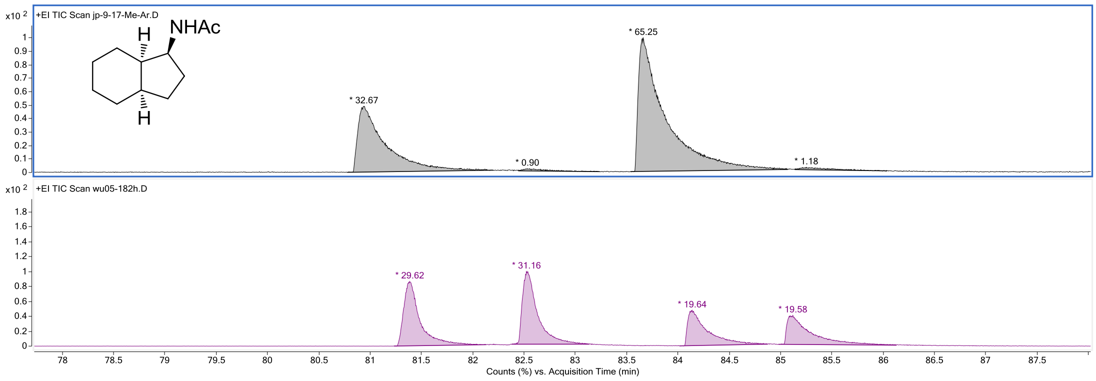

+EI TIC Scan jp-9-17-Me-Ar.D
NHAc
H
H
* 32.67
* 0.90
* 65.25
* 1.18
+EI TIC Scan wu05-182h.D
* 29.62
* 31.16
* 19.64
* 19.58
Counts (%) vs. Acquisition Time (min)

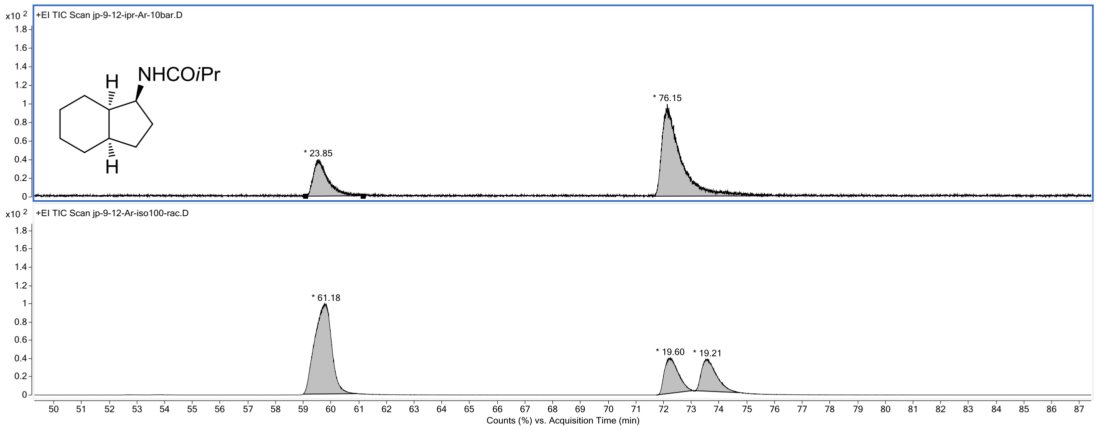
+EI TIC Scan jp-9-12-ipr-Ar-10bar.D
NHCOiPr
H
H
* 23.85
* 76.15
+EI TIC Scan jp-9-12-Ar-iso100-rac.D
* 61.18
* 19.60
* 19.21
Counts (%) vs. Acquisition Time (min)

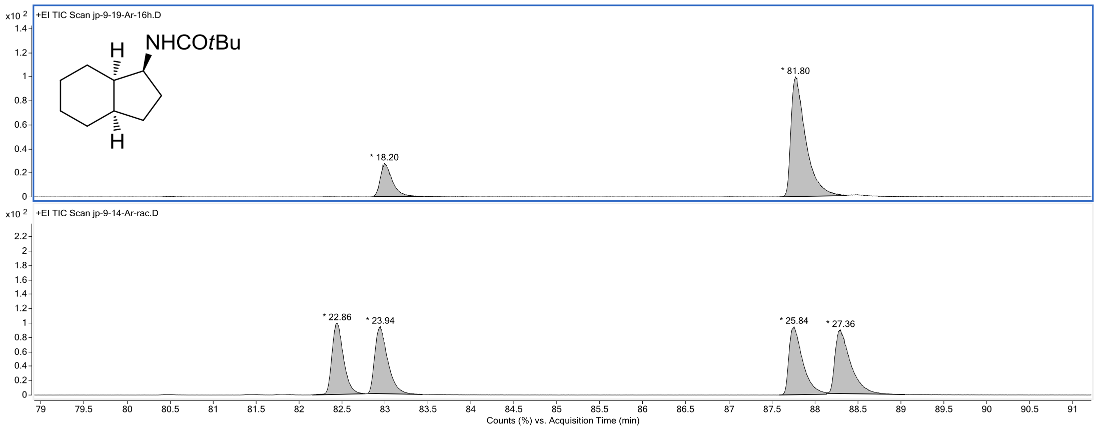
+EI TIC Scan jp-9-19-Ar-16h.D
NHCOtBu
H
H
* 18.20
* 81.80
+EI TIC Scan jp-9-14-Ar-rac.D
* 22.86
* 23.94
* 25.84
* 27.36
Counts (%) vs. Acquisition Time (min)

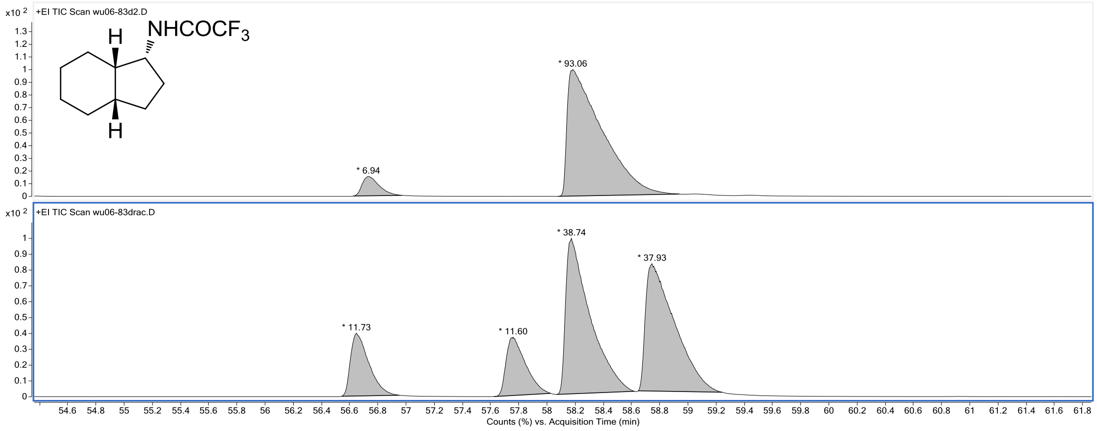
+EI TIC Scan wu06-83d2.D
NHCOCF3
H
H
* 6.94
* 93.06
+EI TIC Scan wu06-83drac.D
* 11.73
* 11.60
* 38.74
* 37.93
Counts (%) vs. Acquisition Time (min)

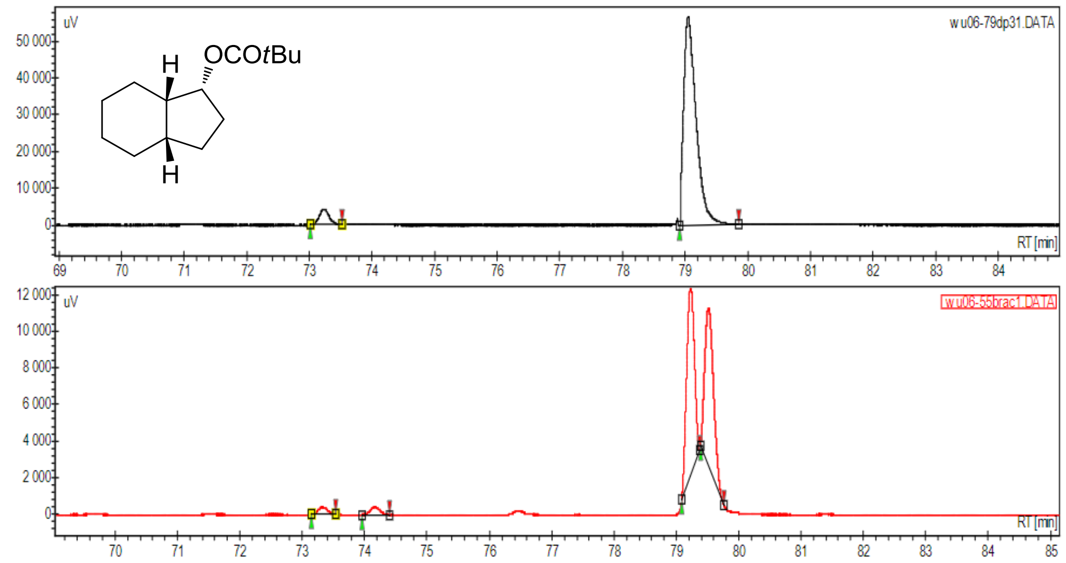


wu06-79dp31.DATA [FID]

| Index | Time [Min] | Quantity [% Area] | Height [uV] | Area [uV.Min] | Area % [%] |
|---|---|---|---|---|---|
| 1 | 73.23 | 5.58 | 4180.3 | 761.8 | 5.576 |
| 2 | 79.05 | 94.42 | 56552.4 | 12901.1 | 94.424 |
| | | | | | |
| Total | | 100.00 | 60732.7 | 13663.0 | 100.000 |

wu06-55brac1.DATA [FID]

| Index | Time [Min] | Quantity [% Area] | Height [uV] | Area [uV.Min] | Area % [%] |
|---|---|---|---|---|---|
| 1 | 73.32 | 2.21 | 390.7 | 67.1 | 2.213 |
| 2 | 74.17 | 2.43 | 411.5 | 73.5 | 2.425 |
| 3 | 79.23 | 48.98 | 10342.8 | 1484.9 | 48.982 |
| 4 | 79.52 | 46.38 | 8637.1 | 1405.9 | 46.379 |
| | | | | | |
| Total | | 100.00 | 19782.1 | 3031.4 | 100.000 |

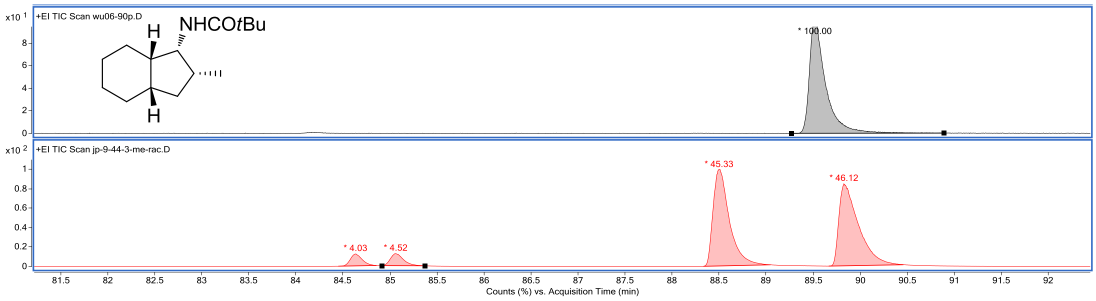


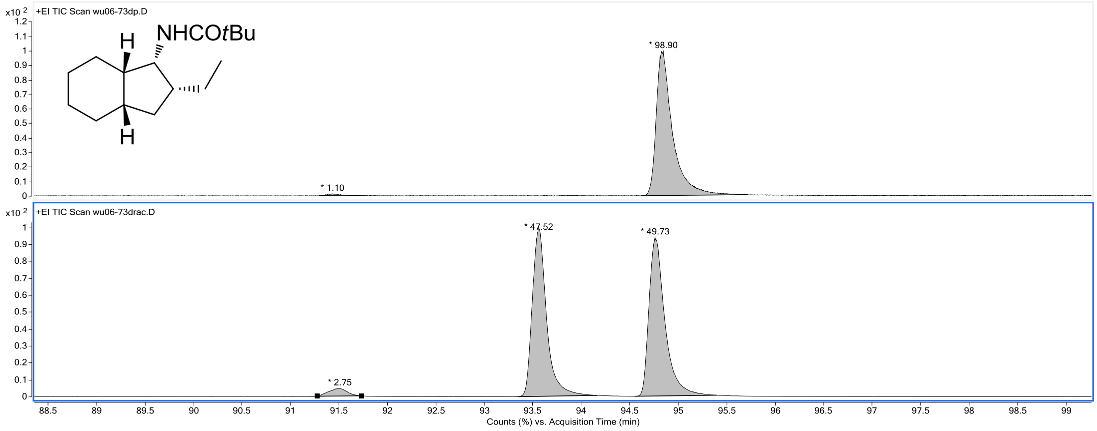

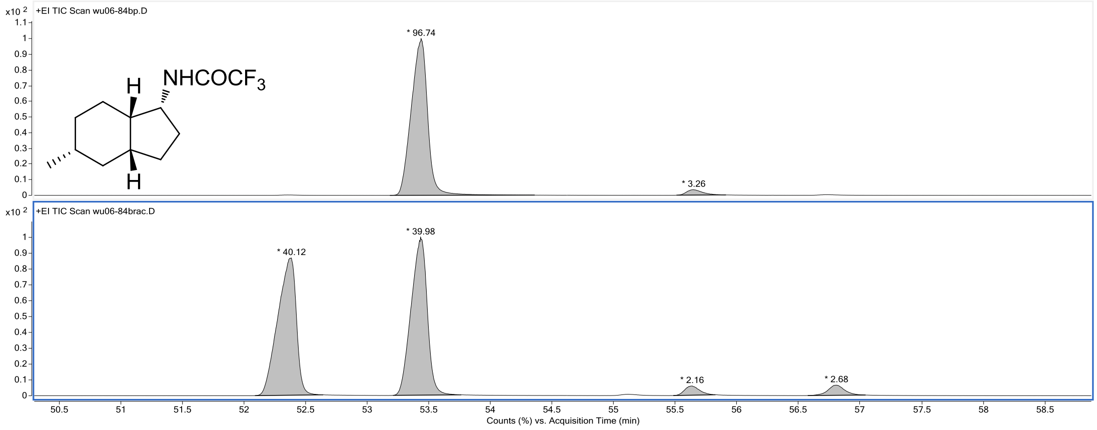
+EI TIC Scan wu06-84bp.D
NHCOCF3
H
H
* 96.74
* 3.26
+EI TIC Scan wu06-84brac.D
* 40.12
* 39.98
* 2.16
* 2.68
Counts (%) vs. Acquisition Time (min)

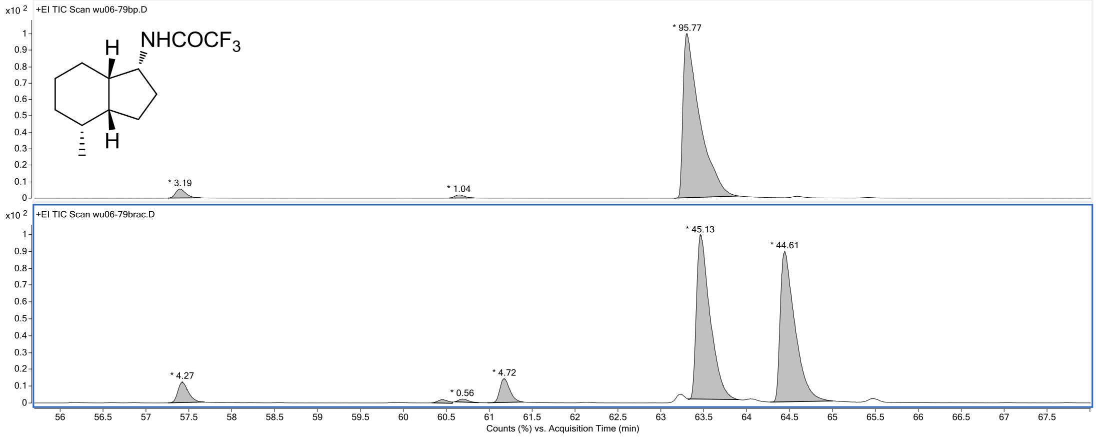
+EI TIC Scan wu06-79bp.D
NHCOCF3
H
H
* 3.19
* 1.04
* 95.77
+EI TIC Scan wu06-79brac.D
* 4.27
* 0.56
* 4.72
* 45.13
* 44.61
Counts (%) vs. Acquisition Time (min)

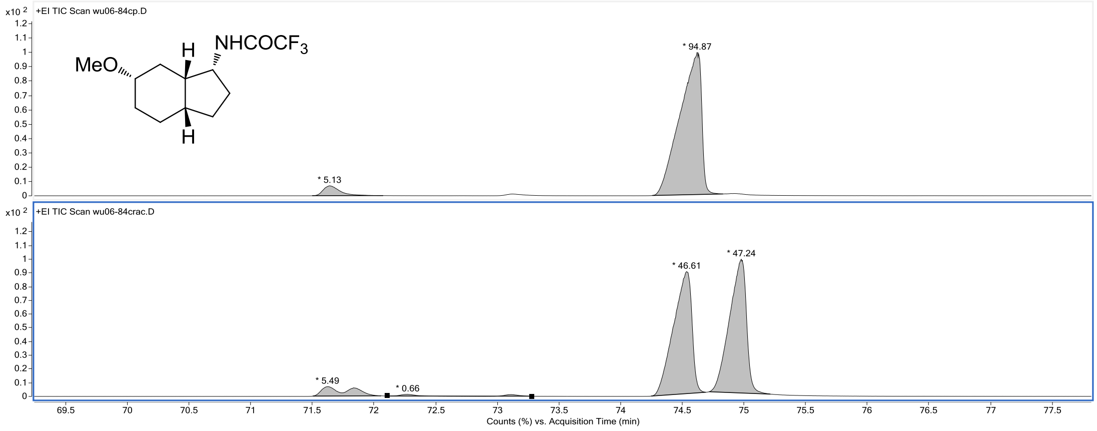
+EI TIC Scan wu06-84cp.D
MeO
NHCOCF3
H
H
* 5.13
* 94.87
+EI TIC Scan wu06-84crac.D
* 5.49
* 0.66
* 46.61
* 47.24
Counts (%) vs. Acquisition Time (min)

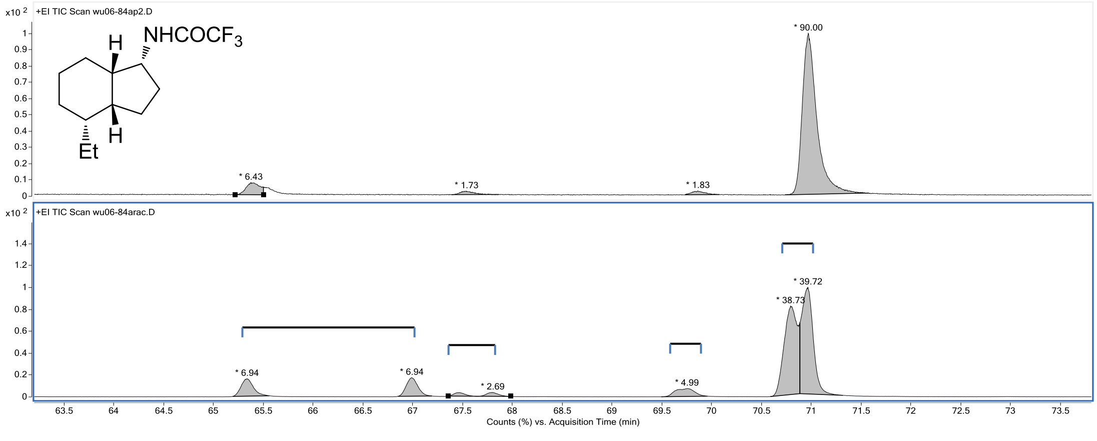
+EI TIC Scan wu06-84ap2.D
NHCOCF3
H
H
Et
* 6.43
* 1.73
* 1.83
* 90.00
+EI TIC Scan wu06-84arac.D
* 6.94
* 6.94
* 2.69
* 4.99
* 38.73
* 39.72
Counts (%) vs. Acquisition Time (min)

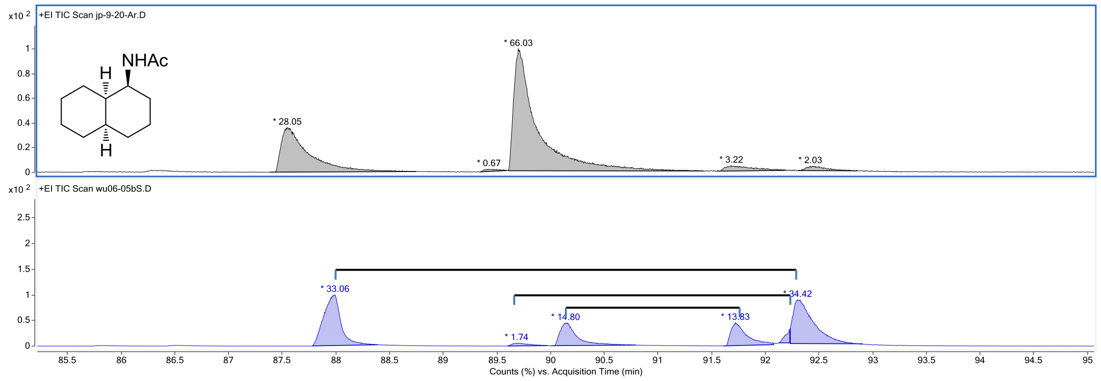
+EI TIC Scan jp-9-20-Ar.D
NHAc
H
H
* 28.05
* 0.67
* 66.03
* 3.22
* 2.03
+EI TIC Scan wu06-05bS.D
* 33.06
* 1.74
* 14.80
* 13.83
* 34.42
Counts (%) vs. Acquisition Time (min)

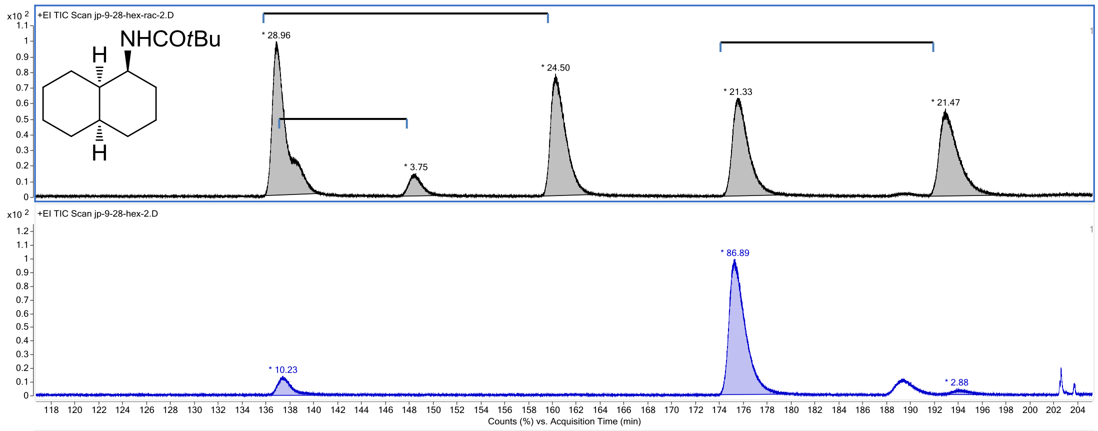
+EI TIC Scan jp-9-28-hex-rac-2.D
NHCOtBu
H
H
* 28.96
* 3.75
* 24.50
* 21.33
* 21.47
+EI TIC Scan jp-9-28-hex-2.D
* 10.23
* 86.89
* 2.88
Counts (%) vs. Acquisition Time (min)

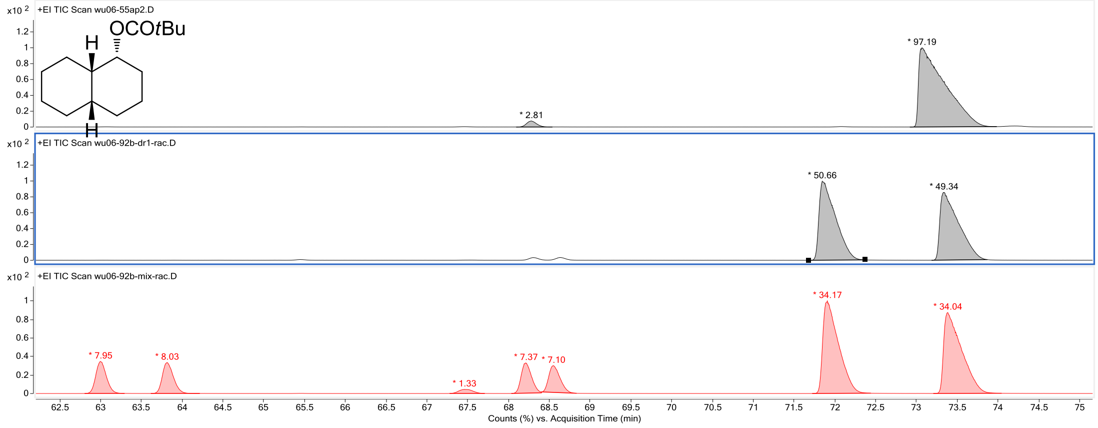
+EI TIC Scan wu06-55ap2.D
OCOtBu
H
H
* 2.81
* 97.19
+EI TIC Scan wu06-92b-dr1-rac.D
* 50.66
* 49.34
+EI TIC Scan wu06-92b-mix-rac.D
* 7.95
* 8.03
* 1.33
* 7.37
* 7.10
* 34.17
* 34.04
Counts (%) vs. Acquisition Time (min)

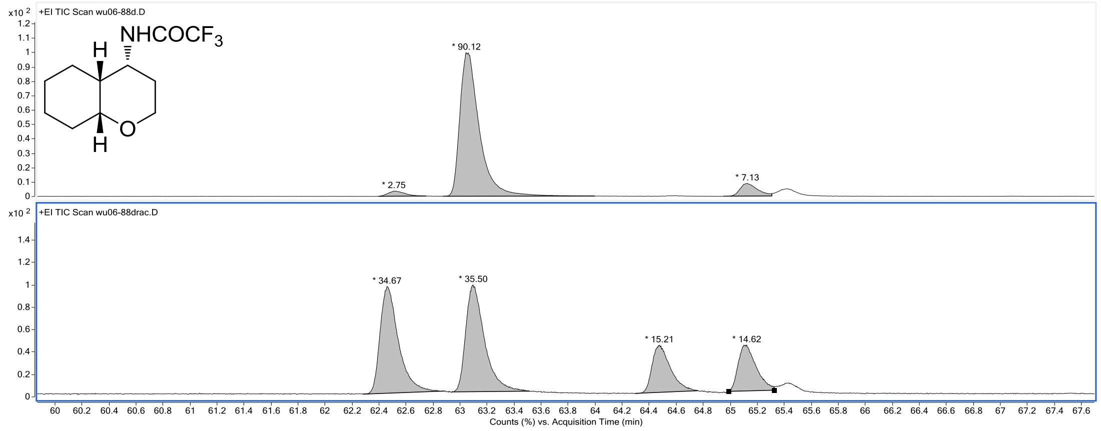
+EI TIC Scan wu06-88d.D
$NHCOCF_3$
H
H
O
* 2.75
* 90.12
* 7.13
+EI TIC Scan wu06-88drac.D
* 34.67
* 35.50
* 15.21
* 14.62
Counts (%) vs. Acquisition Time (min)

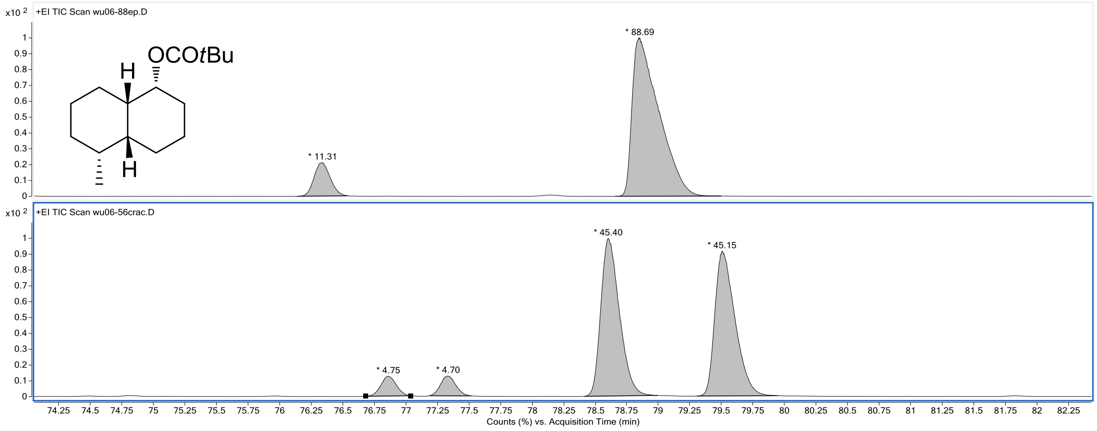

x10 2
+EI TIC Scan wu06-88ep.D
OCOtBu
H
H
* 11.31
* 88.69
x10 2
+EI TIC Scan wu06-56crac.D
* 4.75
* 4.70
* 45.40
* 45.15
Counts (%) vs. Acquisition Time (min)


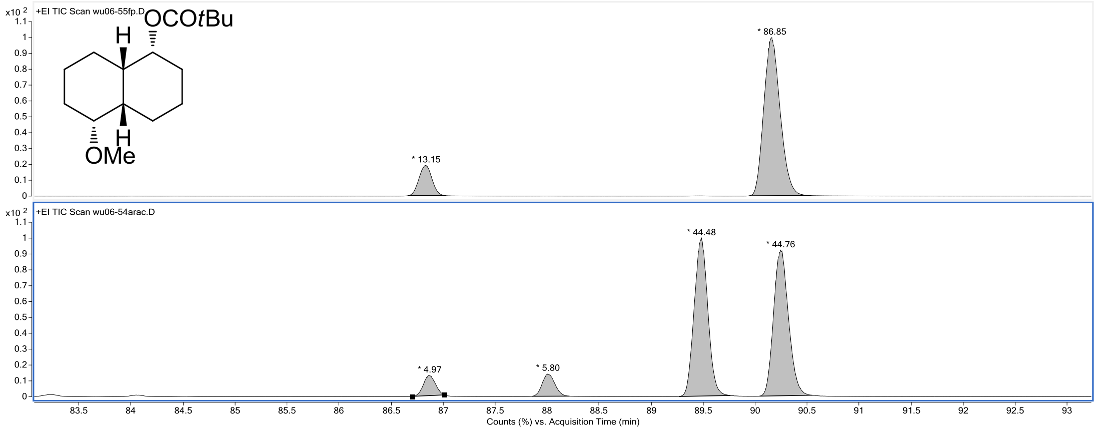

x10 2
+EI TIC Scan wu06-55fp.D
OCOtBu
H
H
OMe
* 13.15
* 86.85
x10 2
+EI TIC Scan wu06-54arac.D
* 4.97
* 5.80
* 44.48
* 44.76
Counts (%) vs. Acquisition Time (min)

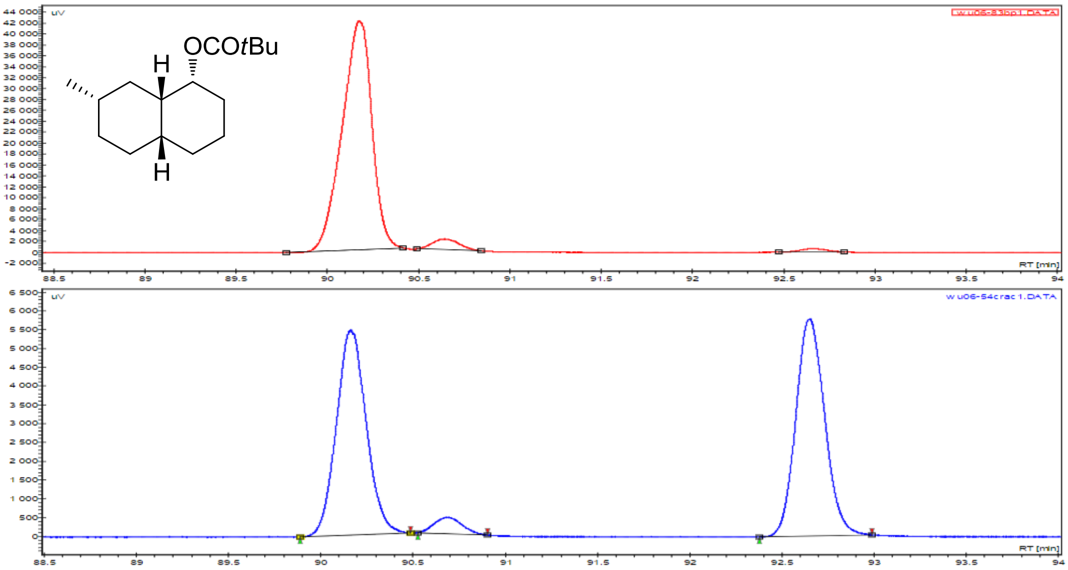


wu06-83bp1.DATA [FID]

| Index | Time [Min] | Quantity [% Area] | Height [uV] | Area [uV.Min] | Area % [%] |
|---|---|---|---|---|---|
| 1 | 90.17 | 94.73 | 41941.9 | 7914.8 | 94.731 |
| 3 | 90.65 | 4.04 | 1957.0 | 337.3 | 4.037 |
| 2 | 92.66 | 1.23 | 631.3 | 103.0 | 1.233 |
| | | | | | |
| Total | | 100.00 | 44530.2 | 8355.0 | 100.000 |

wu06-54crac1.DATA [FID]

| Index | Time [Min] | Quantity [% Area] | Height [uV] | Area [uV.Min] | Area % [%] |
|---|---|---|---|---|---|
| 1 | 90.16 | 47.65 | 5465.6 | 1024.1 | 47.648 |
| 2 | 90.70 | 3.59 | 437.7 | 77.1 | 3.588 |
| 3 | 92.65 | 48.76 | 5763.2 | 1048.1 | 48.764 |
| | | | | | |
| Total | | 100.00 | 11666.5 | 2149.3 | 100.000 |

## 11. References


1. (a) Jiang, Q., Xiao, D., Zhang, Z., Cao, P. & Zhang, X. Highly Enantioselective Hydrogenation of Cyclic Enol Acetates Catalyzed by a Rh–PennPhos Complex. Angew. Chem. Int. Ed. 1999, 38, 516-518. (b) Zhang, W.; Zhang, X., Synthesis of Triphosphorous Bidentate Phosphine–Phosphoramidite Ligands: Application in the Highly Enantioselective Hydrogenation of ortho-Substituted Aryl Enamides. Angew. Chem. Int. Ed. 2006, 45, 5515-5518.
2. Ensign, S. C., Vanable, E. P., Kortman, G. D., Weir, L. J. & Hull, K. L. Anti-Markovnikov Hydroamination of Homoallylic Amines. *J. Am. Chem. Soc.* **2015**, *137*, 13748-13751.
3. Oro, L. A., Valderrama, M., Cifuentes, P., Foces-Foces, C. & Cano, F. H. Azulene as a ligand in cationic rhodium and iridium complexes. Crystal structure of [Rh(TFB)(az)]PF6. *J. Organomet. Chem.* **1984**, *276*, 67-77.
4. Burk, M. J., Casy, G. & Johnson, N. B. A Three-Step Procedure for Asymmetric Catalytic Reductive Amidation of Ketones. *J. Org. Chem.* **1998**, *63*, 6084-6085.
5. Massaro, L. *et al.* Stereodivergent Synthesis of Trisubstituted Enamides: Direct Access to Both Pure Geometrical Isomers. *J. Org. Chem.* **2019**, *84*, 13540-13548.
6. Guan, Z.-H., Zhang, Z.-Y., Ren, Z.-H., Wang, Y.-Y. & Zhang, X. Synthesis of Enamides via CuI-Catalyzed Reductive Acylation of Ketoximes with NaHSO3. *J. Org. Chem.* **2011**, *76*, 339-341.
7. Yousuf, M. & Adhikari, S. One-Pot Synthesis of 3-Substituted 2-Arylpyrrole in Aqueous Media via Addition–Annulation of Arylboronic Acid and Substituted Aliphatic Nitriles. *Org. Lett.* **2017**, *19*, 2214-2217.
8. Guan, Z.-H., Huang, K., Yu, S. & Zhang, X. Synthesis of Enamides via Rh/C-Catalyzed Direct Hydroacylation of Ketoximes. *Org. Lett.* **2009**, *11*, 481-483.
9. Li, B.-J. *et al.* Cross-Coupling of Alkenyl/Aryl Carboxylates with Grignard Reagent via Fe-Catalyzed C−O Bond Activation. *J. Am. Chem. Soc.* **2009**, *131*, 14656-14657.
10. Yang, H., Wang, E., Yang, P., Lv, H. & Zhang, X. Pyridine-Directed Asymmetric Hydrogenation of 1,1-Diarylalkenes. *Org. Lett.* **2017**, *19*, 5062-5065.
11. Zhu, G., Chen, Z. & Zhang, X. Highly Efficient Asymmetric Synthesis of β-Amino Acid Derivatives via Rhodium-Catalyzed Hydrogenation of β-(Acylamino)acrylates. *J. Org. Chem.* **1999**, *64*, 6907-6910.
12. Storch, G. & Trapp, O. Temperature-Controlled Bidirectional Enantioselectivity in a Dynamic Catalyst for Asymmetric Hydrogenation. *Angew. Chem. Int. Ed.* **2015**, *54*, 3580-3586.
13. Zhang, Z., Zhu, G., Jiang, Q., Xiao, D. & Zhang, X. Highly Enantioselective Hydrogenation of Cyclic Enamides Catalyzed by a Rh-PennPhos Catalyst. *J. Org. Chem.* **1999**, *64*, 1774-1775.
14. Ranade, V. S., Consiglio, G. & Prins, R. Functional-Group-Directed Diastereoselective Hydrogenation of Aromatic Compounds. 21. *J. Org. Chem.* **2000**, *65*, 1132-1138.
15. Solladié-Cavallo, A., Ahmed, B., Schmitt, M. & Garin, F. Heterogeneous hydrogenation of 1-naphtol and 2-naphtol over $Ru/Al_2O_3$: a simple 1H NMR

method for determination of the diastereoselectivity. *C. R. Chimie*. **2005**, *8*, 1975-1980.

16. Reeves, J. T.; Tan, Z.; Han, Z. S.; Li, G.; Zhang, Y.; Xu, Y.; Reeves, D. C.; Gonnella, N. C.; Ma, S.; Lee, H.; Lu, B. Z.; Senanayake, C. H., Direct Titanium-Mediated Conversion of Ketones into Enamides with Ammonia and Acetic Anhydride. *Angew. Chem. Int. Ed.* **2012**, *51*, 1400-1404.
